# The reactivity and conformational control of cyclic tetrapeptides derived from aziridine-containing amino acids†
†Electronic supplementary information (ESI) available. See DOI: 10.1039/c6sc01687a



**DOI:** 10.1039/c6sc01687a

**Published:** 2016-06-30

**Authors:** Benjamin K. W. Chung, Christopher J. White, Conor C. G. Scully, Andrei K. Yudin

**Affiliations:** a Davenport Research Laboratories , Department of Chemistry , The University of Toronto , 80 St. George Street , Toronto , Ontario M5S 3H6 , Canada . Email: ayudin@chem.utoronto.ca

## Abstract

Among the smallest of the macrocyclic peptides, 12- and 13-membered cyclic tetrapeptides are particularly noteworthy because they exhibit a broad spectrum of biological activities due to their innate capacity to mimic β-turns in proteins. In this report, we demonstrate that aziridine-containing cyclic tetrapeptides offer a platform to interrogate the conformational properties of tetrapeptides. We show that aziridine ring-opening of 12-membered cyclic tetrapeptides yields exclusively 13-membered α_3_β macrocycles, regardless of peptide sequence, nucleophile, aziridine β-carbon substitution, or stereochemistry. NMR and computational studies on two related aziridine-containing cyclic tetrapeptides revealed that the amide conformations of their *N*-acyl aziridines are similar, and are likely the determinant of the observed ring-opening regioselectivity. Interestingly, some of the resulting 13-membered α_3_β macrocycles were found to be conformationally heterogeneous. This study on the reactivity and conformational control of aziridine-containing cyclic tetrapeptides provides useful insight on the design and development of macrocyclic therapeutics.

## Abstract 2

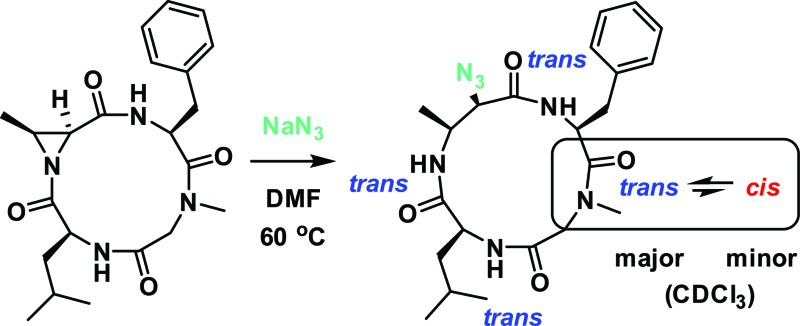
Regioselective ring-opening at a flexible *N*-acyl aziridine enables conformational control of α_3_β cyclic tetrapeptides through torsional strain.

Macrocycles are cyclic molecules that consist of 12 ring atoms or more, and there has recently been considerable interest in the use of these molecules in drug discovery.^1^ This surge has been driven by efforts to improve the stability, potency, and selectivity of peptide-based therapeutics and molecular probes.^2^ Macrocycle-protein interactions are complex and multifaceted,^3^ characterized by elaborate conformational rearrangements. In an analogy to chemical reactivity, just as the ground state conformation of a molecule is rarely the reactive one, the ground state conformation of a given macrocycle is not necessarily biologically relevant. For systems with high barriers to conformational interconversion, there is a possibility that a macrocycle might be “stuck” in an energy state that: (a) is not biologically active and (b) does not have a rapid escape route to the biologically relevant pose. While this is uncommon, cases that document slow on-rate kinetics point in this direction. For instance, obtaining a high quality co-crystal structure between a Grb2 SH2 domain and its macrocyclic inhibitor involved heating the sample at 50 °C for 10 minutes.^4^ Although not directly aimed at macrocycles, a study on conformational reorganization of ligands upon protein binding revealed that over 60% of ligands bind in a conformation that is at least 5 kcal mol^–1^ higher in energy than their unbound conformation in solution.^5^ In peptide macrocycles, energy barriers associated with conformational reorganization can become even larger when molecular rearrangements involve higher energy rotations such as *cis*–*trans* isomerization of amide bonds, which is characterized by a barrier of 16–22 kcal mol^–1^.^6^ Taken together, these findings suggest a need to not only develop new strategies for macrocyclization, but also to understand the influence of conformation on reactivity and behaviour of macrocycles.

Cyclic tetrapeptides are tremendously important tools to study β-turns and present a good opportunity to explore the conformational properties of macrocycles. Many are potent and selective molecules exhibiting a wide spectrum of biological activities. Examples include hirsutide, a cardiac calcium channel blocker,^7^ and HDAC inhibitors chlamydocin^8^ and the azumamides A–E.^9^ In addition, cyclic tetrapeptides often exhibit conformational dynamics in solution due to *cis*–*trans* isomerization of their amide bonds. In protein structures, few amides exist in the *cis* conformation: only 0.03% of non-proline amides and 5% of Xaa-Pro bonds (Xaa represents any amino acid) adopt *cis*-amide conformations.^10^ On the other hand, cyclic tetrapeptides contain a marked increase in *cis*-amide bond population because 12-membered ring strain leads to distortion of the amide geometries and lowers the barrier of *cis*–*trans* amide isomerization.^11^ Indeed, many 12-membered cyclic tetrapeptides display solvent-dependent conformational heterogeneity corresponding to *cis*–*trans* amide isomers, characterized either by broad NMR peaks or by additional amino acid spin systems.^12^ However, 13-membered α_3_β cyclic peptides have been found to be conformationally homogeneous in solution.^13^ Fairlie and co-workers have reported a design strategy in which 13-membered cyclic peptides containing a β-amino acid became conformationally homogeneous when a β residue was placed across the ring from a tertiary amide.^14,15^ Since then, other 13-membered α_3_β cyclic peptides have been characterized, including those by Arvidsson *et al.*,^13*c*^ documenting the effect of relative stereochemistry on structural heterogeneity.

We became interested in the structural fluctuations of cyclic tetrapeptides in different environments, and how conformations could be controlled by the atomic makeup of the macrocycle. In this report, we describe the conformational properties of 12-membered aziridine-containing cyclic tetrapeptides, and characterize the kinetics of structural interconversion in a conformationally heterogeneous 13-membered cyclic tetrapeptide. We propose that torsional strain in the β-residue of α_3_β cyclic peptides can yield conformationally heterogeneous 13-membered rings. This study of aziridine-containing cyclic tetrapeptides enables a further understanding of the conformational dynamics in medium-sized macrocyclic molecules.

In our initial report, we described the synthesis of the 12-membered cyclo-[Leu-Cma-Phe-Gly] (**1**, Cma = *cis*-methyl Azy, or (2*S*,3*S*)-3-methylaziridine-2-carboxylic acid) along with its azide and 7-mercapto-4-methyl coumarin ring-opened derivatives. We noted that ring-opening of the Cma residue occurred regioselectively with nucleophilic attack at the α-position to give a 13-membered ring, containing a β-amino acid. This was in contrast with linear systems incorporating Cma, which typically result in α-amino acid formation.^15^ To explore the effect of aziridine substitution and stereochemistry on the regioselectivity of ring-opening and to determine the extent to that these changes perturb the conformations of the resulting macrocycles, the Azy (**2**) and Tma (**3**, Tma = *trans*-methyl Azy, (2*S*,3*R*)-3-methylaziridine-2-carboxylic acid) variations were prepared (Fig. 1, see ESI† for synthesis).

**Fig. 1:**
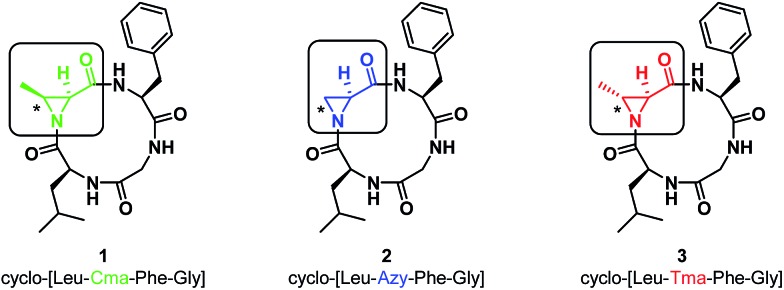
Structures of aziridine-containing 12-membered cyclic tetrapeptides with varying substitution and stereochemistry at the Azy residue (marked with an asterisk).

To evaluate if stereochemistry or substitution at C3 of the aziridine would influence its reactivity under nucleophilic ring-opening conditions, **2** and **3** were both subjected to aziridine ring-opening by sodium azide (**2a** and **3a**) and thiophenol (**2b** and **3b**). Additionally, compound **2** was treated with diethylamine^16^ (**2c**). In all cases, independent of nucleophile and aziridine substitution or stereochemistry, 13-membered α_3_β scaffolds were exclusively formed (Scheme 1).

**Scheme 1:**
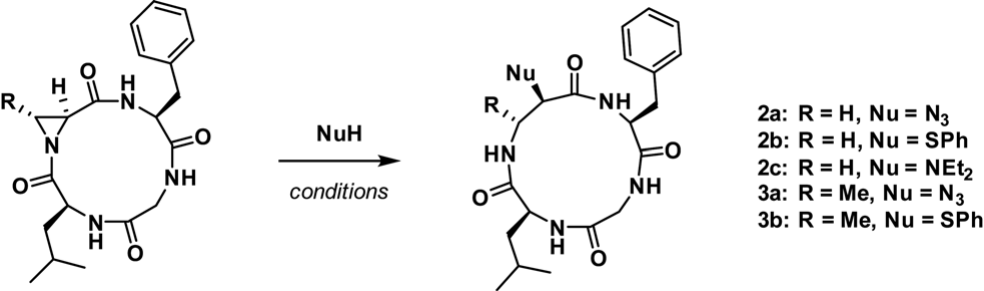
Aziridine ring-opening of **2** and **3** with various nucleophiles.

Next, residues on either side of the Azy amino acid were varied to determine if there was a sequence dependence on the regioselectivity of aziridine ring-opening. Linear peptides H-Ala-Cma-Phe-Gly-OH, H-Phe-Cma-Pro-Gly-OH, and H-Leu-Azy-Pro-Gly-OH were thus prepared. The rationale for an Leu to Ala mutation was to test if modulating the sterics around the aziridine would have an effect on its ring-opening. Incorporation of a neighbouring Pro residue was performed to evaluate if the presence of a secondary amino acid (yielding a tertiary amide bond) could alter the 12-membered ring conformation and ultimately affect aziridine ring-opening, since Xaa-Pro amide bonds are known to exhibit reduced barriers to *cis*–*trans* isomerization in macrocyclic systems.^17^ The three linear tetrapeptides were cyclized and subjected to aziridine ring-opening with sodium azide in a telescopic synthesis to yield compounds **4a**, **5a**, and **6a**, respectively (Fig. 2). As with previous examples, 13-membered α_3_β scaffolds were exclusively formed.

**Fig. 2:**
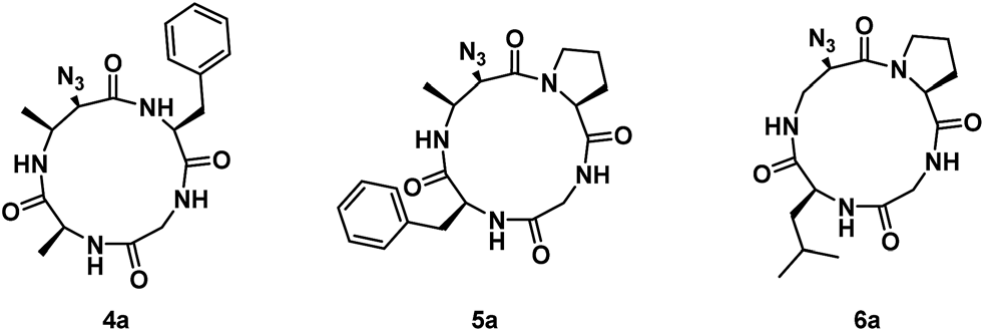
Structures of azide ring-opened 13-membered α_3_β rings with varying side chains around the Azy residue.

All examples of aziridine ring-opening of Azy-containing cyclic tetrapeptides have been shown to be highly regioselective (>20 : 1, by HPLC) towards nucleophilic attack at the α-carbon of Azy/Cma/Tma to generate the corresponding α-substituted β-amino acid residue, yielding 13-membered α_3_β rings. Taking all of this data into account, we speculate that this phenomenon is rooted in the conformation of the Azy-containing macrocycle. Indeed, the observed regioselectivity of aziridine ring-opening speaks to the adoption of different reactive conformations of the flexible *N*-acyl aziridine bond depending on whether it is within a 12-membered peptide macrocycle or a linear peptide.

To study the conformations of *N*-acyl aziridine-containing macrocycles, we decided to focus on the structures **1** and **3**. Broad singlet peaks observed in the ^1^H NMR spectra of the compounds suggested that they are conformationally flexible. We performed additional NMR experiments on compounds **1** and **3**. In the NOESY spectrum of **1**, cross peaks were observed between Phe-α/Gly-α and Leu-α/Cma-α protons, suggesting that the corresponding amides are in a *cis* conformation. The absence of α-to-α cross peaks in the remaining Cma-Phe and Gly-Leu amides suggest that they are in a *trans* conformation.^18^ The ROESY spectrum of **3** exhibited the same pattern of cross peaks, suggesting that the amide conformations of both Cma and Tma derivatives are the same. VT NMR experiments were also performed on **1** and **3**, and the data shows that compound **3** possesses one fewer hydrogen bonding interaction with its amide protons, suggesting that, even though they have similar amide conformations, the overall conformation of **3** is more flexible than **1** (Fig. 3).

**Fig. 3:**
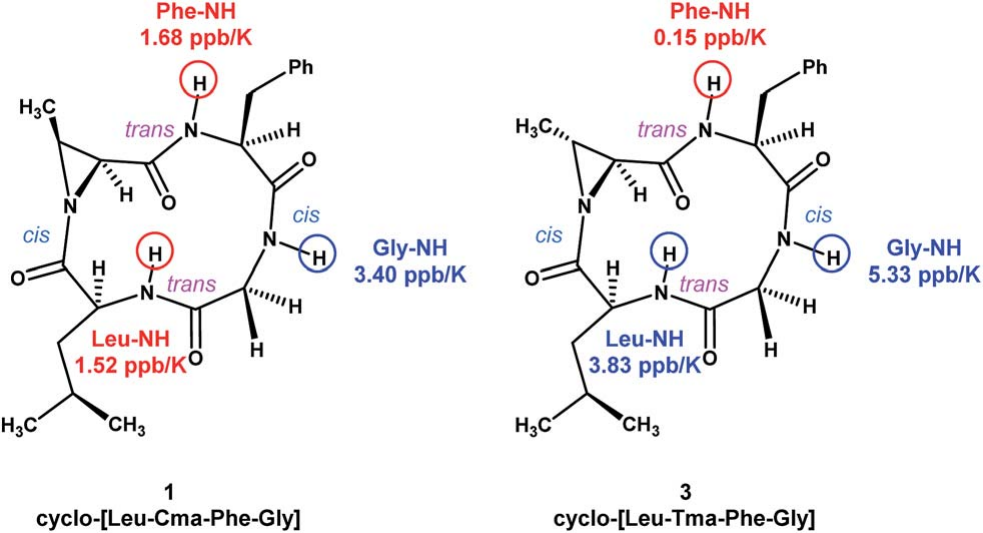
Global amide conformations and amide temperature coefficients of **1** and **3**.

To further investigate the structural differences between aziridine-containing macrocycles **1** and **3**, we modelled their structures using restrained conformational searches in molecular mechanics calculations with the NMR-derived amide constraints, followed by DFT-based optimization of the 20-member conformational ensemble to a single low energy conformer using B3LYP/6-31+G(d,p) (Fig. 4). This revealed that the nitrogen pyramid for Cma-containing **1**, at 35° (between the [C=O_Leu_, C_α,Cma_] plane and N_Cma_) is steeper than that of Tma-containing **3**, at 27° (between the [C=O_Leu_, C_α,Tma_] plane and N_Tma_). As seen in other *N*-acyl aziridine-containing peptides, the out-of-plane pyramidalization at the aziridine nitrogen of both **1** and **3** indicates the presence of a distorted amide.^19^ Furthermore, a steeper nitrogen pyramid in **1** corresponds with reduced nitrogen lone pair delocalization and therefore increased carbonyl character at the aziridine amide. This correlates with the experimentally determined ^13^C NMR shifts of the aziridine amide carbonyl, at 183.8 ppm for **1** and 180.4 ppm for **3**, where a more downfield shift in **1** is indicative of increased carbonyl character and a steeper nitrogen pyramid at the aziridine amide. While there is a slight difference in the steepness of the nitrogen pyramid in Cma *vs.* Tma-derived macrocycles, this does not translate to altered reactivity for aziridine ring-opening, as 13-membered α_3_β macrocycles are exclusively formed regardless of aziridine geometry. This is likely due to similar amide conformations (Fig. 3, in particular at the *N*-acyl aziridine amide) within 12-membered aziridine-containing macrocycles.

**Fig. 4:**
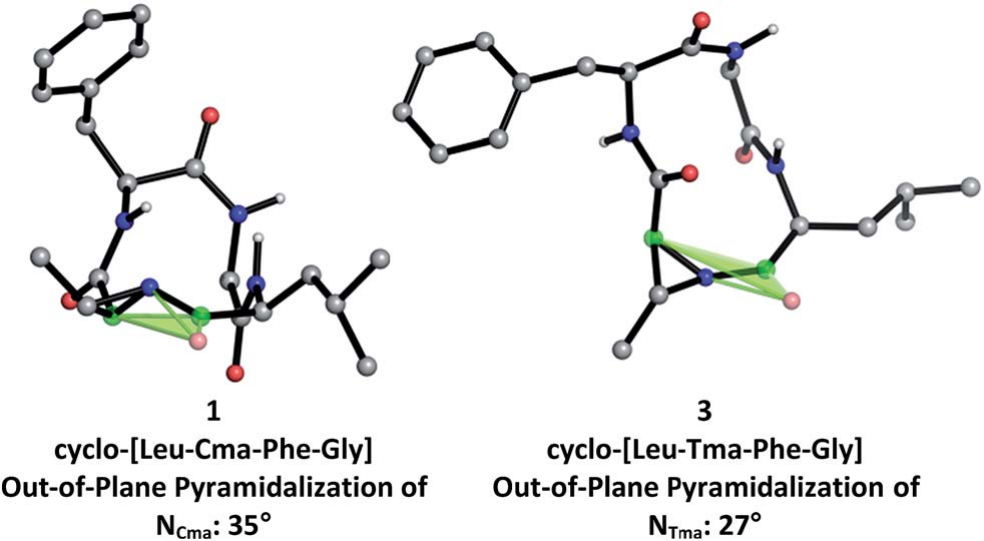
Computationally-derived angles of pyramidalization at aziridine amide for **1** and **3**.

Given the conformations of the 12-membered Azy-containing cyclic peptides and that their ring-opening yields 13-membered α_3_β macrocycles, we propose that the regioselectivity of nucleophilic ring-opening in Azy-containing systems is governed by the conformation of the aziridine amide. This proposal is based on several factors, beginning with the low barrier to rotation around the N–C(O) bond in *N*-acyl aziridines. The barrier to rotation about regular amides is substantially hindered due to partial double bond character, on the order of 16–22 kcal mol^–1^.^20^ Upon going from an sp^2^-like nitrogen of a regular amide, such as *N*,*N*-dimethylformamide, to the more sp^3^-like nitrogen of a pyramidal aziridine amide, the resulting reduction of resonance contribution from the nitrogen lone pair to the π* of the carbonyl leads to a lower rotational barrier. In a seminal study, Anet and Osyany reported that no such barrier to rotation can be measured on *N*-acetylaziridine, observing that internal rotation is fast relative to the NMR time scale even at –160 °C.^21^ Theoretical calculations on the electronic structure and conformational properties of *N*-acyl aziridines have revealed the N–C(O) rotational barrier in *N*-formylaziridine to be 2.84 kcal mol^–1^ by the *ab initio* (3-21G) method,^22^ 3.11 kcal mol^–1^ at the MP2/6-31G* level of theory,^23^ and 3.94 kcal mol^–1^ at the MP2/6-311++G** level of theory.^24^ Due to the facile rotation of this N–C(O) bond, the Azy residues are distinct from other amino acids in that they are conformationally adaptive toward a distorted conformation when placed in an extended peptide chain or within a constrained 12-membered ring.^19^ Even in cyclic peptides containing a tertiary amide (such as in **5a** and **6a**), the N–C(O) rotational barrier of the *N*-acyl aziridine is much lower than that of the tertiary amide (∼15–18 kcal mol^–1^)^25^ and consequently the *N*-acyl aziridine will always be the most flexible amide in the system. Thus, in the context of a strained 12-membered cyclic tetrapeptide, the conformation of the *N*-acyl aziridine is most likely to be *cis* in order to reduce ring strain. Indeed, 2D-NOESY NMR analysis of **1** and **3** revealed that the aziridine amide bond in both compounds exist in a *cis* conformation. As observed, aziridine ring-opening of Azy-containing cyclic tetrapeptides is independent of peptide sequence and is likely to be dependent solely on aziridine amide conformation.

The work of Rademacher and Würthwein has revealed that the so-called “perpendicular” conformation of the carbonyl group to the aziridine ring is stereoelectronically favoured for *N*-acyl aziridines as this conformation maximizes the n(N)/π*(CO) interaction.^26^ The requirement for the n(N)/π*(CO) interaction is thus expected to be a dominant contributor to the transition state for aziridine ring-opening. We therefore reason that the conformation of the aziridine amide plays a significant role in rationalizing our observed regioselectivity, as this factor is translated into the transition state for aziridine ring-opening. A reasonable way to explain the observed regioselectivity is to say that upon aziridine ring-opening, the transoid amide is favoured. Since the adaptive aziridine amide in 12-membered cyclic tetrapeptides adopts a *cis* conformation (Fig. 5, left), when the azide anion attacks the α-carbon of the Azy unit, a favourable *trans*-like amide would form in the transition state (Fig. 5, grey box). On the other hand, linear Azy-containing peptides possess significant flexibility over a constrained cyclic tetrapeptide and should thus be capable of accommodating an aziridine amide bond in its *trans*-conformation. In their study on the X-ray structural analysis of Azy-containing peptides, Goodman and co-workers showed that the distorted aziridine amide in several synthesized Azy-containing peptides adopts a *trans*-conformation.^27^

**Fig. 5:**
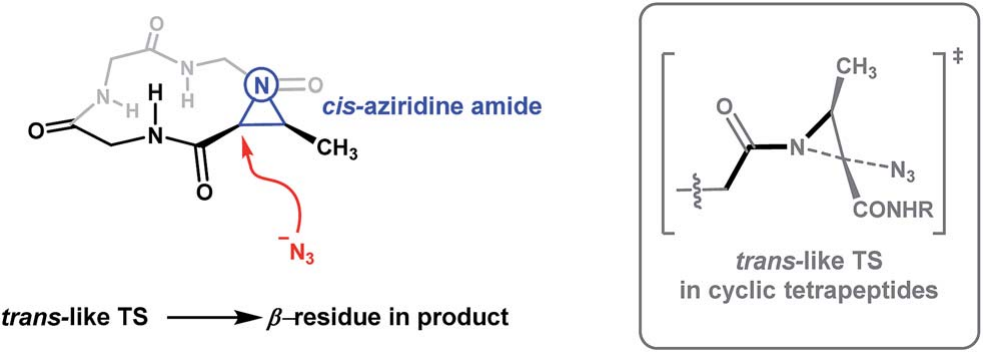
Proposed model to explain the observed regioselectivity in Azy ring-openings of cyclic tetrapeptides. The grey box on the right depicts the proposed energetically favourable *trans*-amide transition state (shown in black bonds) from a different perspective.

Next, we wanted to probe the possibility of modulating the conformation of Azy-containing cyclic tetrapeptides by considering the sequence H-Leu-Cma-Phe-Sar-OH. In this instance, the incorporation of a tertiary amide was across from the Azy residue. The linear peptide was cyclized to yield compound **7**, which was then subjected to aziridine ring-opening with sodium azide to yield compound **7a** (Scheme 2). While a 13-membered α_3_β ring was also formed exclusively in this instance, ^1^H NMR analysis of this compound indicated the existence of two conformations in slow exchange. That is, in the ^1^H NMR spectrum (DMSO-*d*_6_, 298 K) of the purified compound, there were two sets of peaks that corresponded to a major and a minor conformation (1 : 0.09, by integration). 2D NMR analysis permitted a full proton assignment of the major conformation. As the minor conformation was <10% of the mixture, only a partial assignment was possible. However only ^1^H nuclei involved in side chains were ambiguous, while those directly bonded to atoms in the 13-membered macrocycle were assigned, which still enabled conformational analysis of the macrocyclic core.

**Scheme 2:**
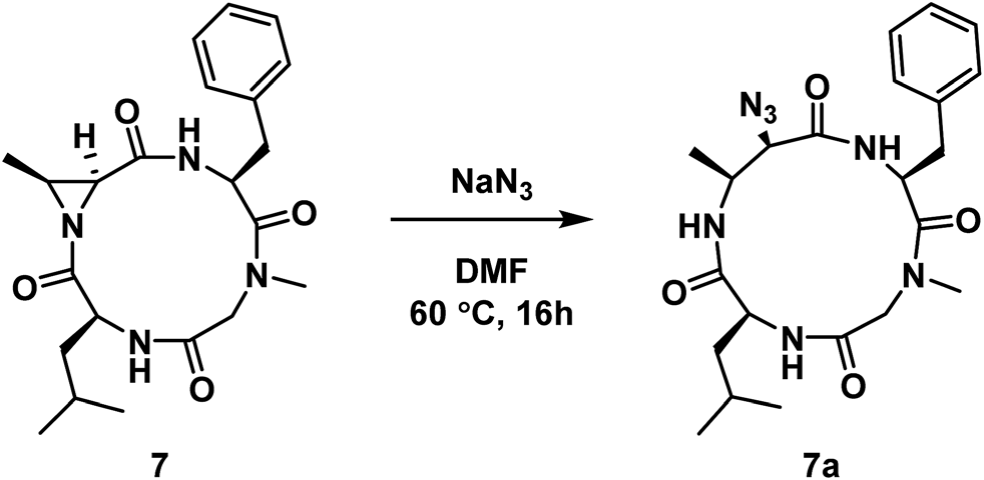
Aziridine ring-opening of **7** with sodium azide.

To verify that these pairs of peaks correspond to interconverting conformations as opposed to a mixture of two different compounds, a ROESY spectrum was obtained. For some pairs of identical ^1^H nuclei in the proposed major and minor conformations, ROESY crosspeaks corresponding to conformational exchange were observed and in opposite in phase to NOE crosspeaks (Fig. 6). In addition, ROESY analysis indicated that the major conformation contains two *cis*-amides at the Sar/Phe and β-amino acid/Leu residues, while the minor conformation contains all *trans*-amides. To quantify the extent of conformational exchange, 1D NOESY experiments were performed. A pair of ^1^H nuclei was chosen for their resolution from neighbouring signals, facilitating selective irradiation of the desired nuclei only. The resulting integrations obtained from the 1D NOESY experiments were analyzed using the EXSYCALC program to yield kinetic parameters *k*_tc_ = 0.029 s^–1^ and *k*_ct_ = 0.395 s^–1^, representing the rates of amide isomerization from *trans* to *cis*, and *cis* to *trans*, respectively (Fig. 7). These values were used to calculate Δ*G*‡tc and Δ*G*‡ct using the Eyring equation, and as a consistency check, the difference between them was used to calculate ΔΔ*G* and *K*, giving a ratio of 1 : 0.07 (major : minor conformation), which is consistent with ^1^H NMR integrations.

**Fig. 6:**
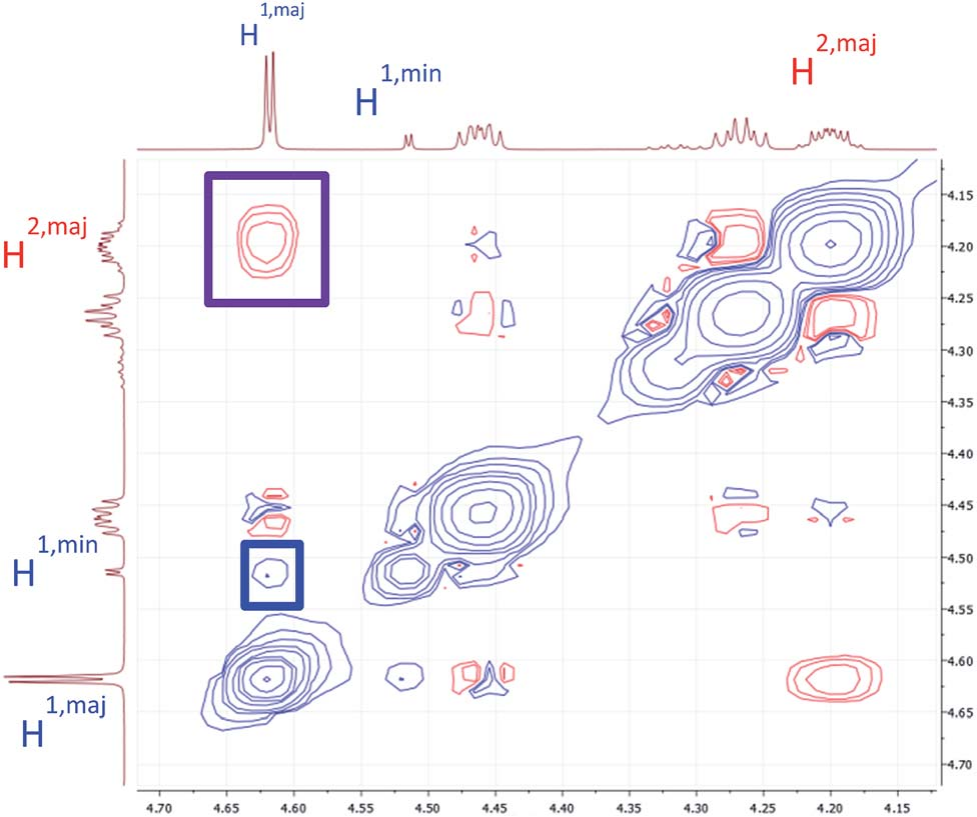
A portion of the ROESY spectrum of **7a** as observed in DMSO-*d*_6_. An NOE crosspeak is outlined in purple, while a conformational exchange (EXSY) crosspeak is outlined in blue.

**Fig. 7:**
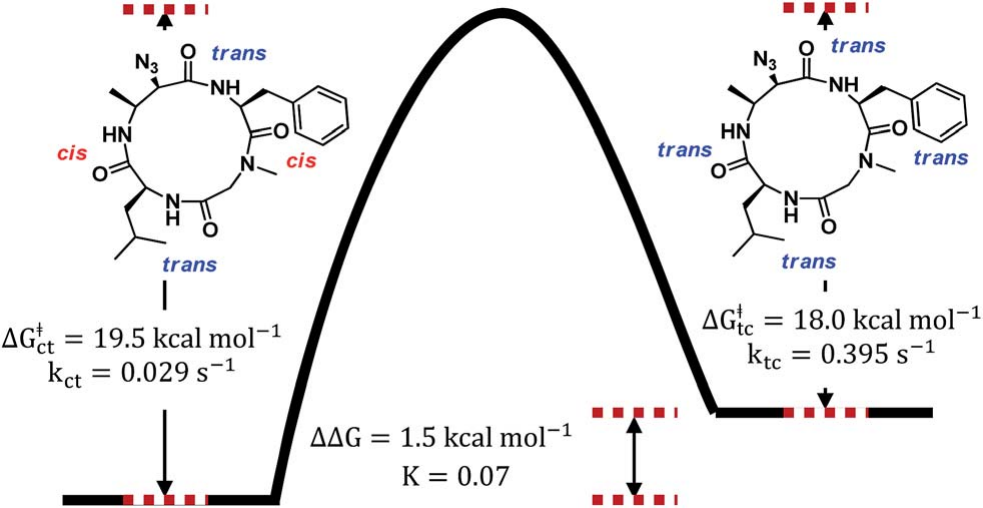
Energy diagram depicting the transition between two conformational states of **7a** as observed in DMSO-*d*_6_. The major conformer has two *cis*-amides at the Sar/Phe and β-amino acid/Leu residues, while the minor conformer is all *trans*.

To determine if there was solvent dependence on major : minor conformer distribution, the sample was lyophilized and re-dissolved in CDCl_3_. Integration of ^1^H NMR signals showed a major : minor conformation distribution of 1 : 0.8, and ROESY analysis indicated that the major conformation is all *trans* while the minor conformation contains one *cis*-amide at the Sar/Phe amide (Fig. 8). The difference in conformational distribution is possibly due to the hydrogen bond-disrupting properties of DMSO.^28^ It is important to note that these experiments were performed only at one temperature (298 K) and peptide concentration (approximately 0.1 M for both DMSO-*d*_6_ and CDCl_3_ samples). It could be that varying concentration and/or temperature could lead to conformational homogenization, but the extent of this phenomenon was not explored.

**Fig. 8:**
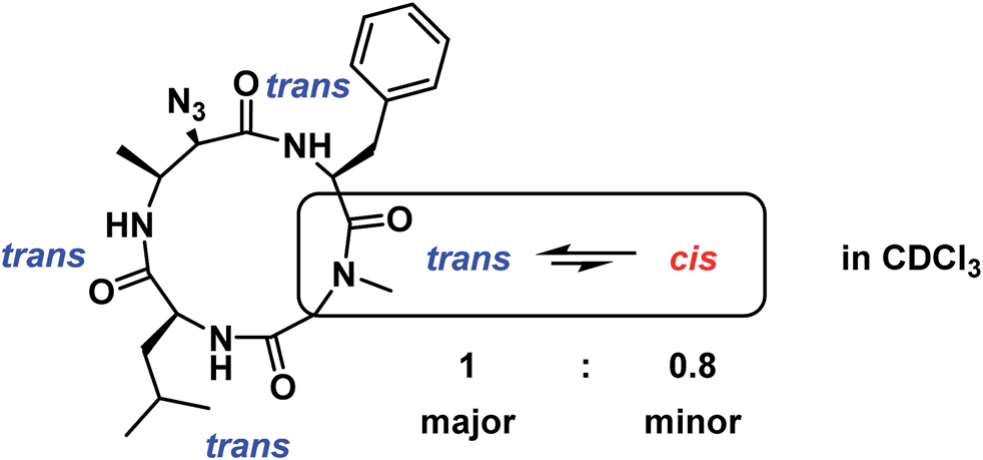
Conformational heterogeneity of **7a** as observed in CDCl_3_. The major conformer is all *trans* while the minor conformer has one *cis*-amide at the Sar/Phe residue, in a 1 : 0.8 ratio of major : minor conformers.

Unexpected conformational heterogeneity observed in some of the α_3_β scaffolds that were formed (namely **5a** and **7a**) suggest that 13-membered rings of this type can be more flexible than previously expected. Fairlie has reported that while 12-membered rings are conformationally flexible, 13-membered rings can be conformationally homogeneous if two turn-inducing constraints (such as Pro residue and a β-amino acid) are separated from each other by a single amino acid, or in other words, are positioned across from each other in the resulting cyclic tetrapeptide. However, compound **7a**, which contains a Phe residue between the tertiary amide and β-amino acid, still exists as a conformationally heterogeneous mixture. In addition, the distribution and conformation of the major and minor conformers differ in an unpredictable manner depending on solvent. In polar DMSO-*d*_6_, the favoured conformation contained two *cis*-amides, while in non-polar CDCl_3_, the major conformer is the all-*trans* structure. Thus, it appears that conformational heterogeneity in α_3_β macrocycles cannot be as straightforwardly predicted as originally thought.

A property that we observe is common between conformationally heterogeneous α_3_β macrocycles is the presence of a tertiary amide (such as Pro or Sar), but even their presence is insufficient to predict conformational heterogeneity, as Pro-containing **6a** exhibits only one conformation by ^1^H NMR analysis. However, this compound is derived from ring-opening of a cyclic Azy peptide, which yields an α-monosubstituted β-residue upon ring-opening. The other conformationally heterogeneous macrocycles are formed from ring-opening of a cyclic Cma peptide, which yields an α,β-disubstituted β-residue upon ring-opening, where the β substituent is derived from the *cis*-3-methyl group present in the starting macrocycle. The presence of α,β-disubstitution in the β-residue of the Cma-derived macrocycles likely leads to torsional strain that raises the barrier to *cis*–*trans* amide isomerization such that multiple conformations are energetically differentiated from each other, resulting in conformational heterogeneity. Indeed, it is the Leu-β-residue amide of N_3_-[Leu-Cma-Phe-Sar] that is undergoing *cis*–*trans* amide isomerization, as observed in the ROESY experiment performed in DMSO-*d*_6_. In conformationally homogeneous α_3_β macrocycles, the β-residue is monosubstituted, whether at the α- (**6a**) or β-position (Fairlie's macrocycle) (Fig. 9), which increases local flexibility at the β-residue and precludes the observation of multiple conformational species on the NMR timescale.

**Fig. 9:**
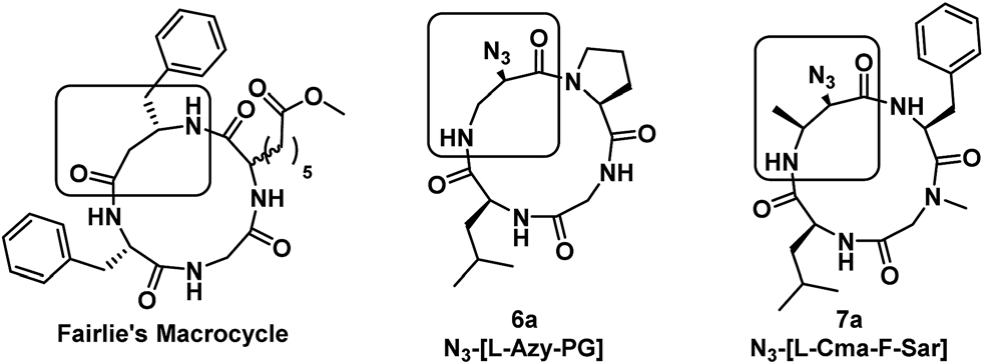
Structures of conformationally homogeneous (Fairlie's macrocycle and **6a**) and heterogeneous macrocycles (**7a**). A rectangle is used to indicate the β-amino acid residue.

Thus, α_3_β cyclic tetrapeptides that contain both a tertiary amide and also possess α,β-disubstitution at the β-residue appear to possess a varying degree of conformational heterogeneity. This can be rationalized by the fact that cyclic tetrapeptides contain 12/13-membered ring strain that results in an increase of *cis* conformers at tertiary amides, thereby bestowing the molecule with the flexibility to access multiple conformational states.^10,11^ The additional property of α,β-disubstitution at the β-residue possibly acts as a rigidifying element that raises the barrier of interconversion between these conformational states and leads to conformational heterogeneity. The origin of this rigidification is analogous to torsional strain observed in alkanes, where the barrier to rotation between C2 and C3 of butane (CH_3_–CH_3_ eclipsed interaction, analogous to α,β-disubstitution at the β-residue) is greater than that between C1 and C2 of propane (H–CH_3_ eclipsed interaction, analogous to a monosubstituted β-residue).^29^

## Conclusions

In summary, this study illuminates the behaviour of an important class of bioactive molecules and expands upon an understanding of the relationship between macrocycle structure and conformation. To this effect, we have shown that site-selective aziridine ring-opening of Azy-containing cyclic tetrapeptides exclusively yields 13-membered α_3_β cyclic peptides, independent of Azy substitution, stereochemistry, nucleophile, and peptide sequence. This suggests that Azy derivatives are generally applicable structural units to access an important class of macrocycles for drug discovery. NMR spectroscopy and modelling studies on 12-membered aziridine-containing cyclic peptides revealed that they possess similar *cis*-amide conformations at the *N*-acyl aziridine amide. According to our proposed model for aziridine ring-opening in these systems, this results in the same regioselectivity observed for Azy-containing macrocycles. However, the regioselectivity of this transformation is opposite to that of Azy-containing linear peptides, and we propose that the origin of this shift is caused by the flexibility of the *N*-acyl aziridine amide conformation. The preparation of conformationally heterogeneous α_3_β macrocycles also suggests that, while the presence of a tertiary amide can increase the flexibility of the molecule allowing access to multiple conformational states, torsional strain from an α,β-disubstituted β-residue operates in raising the barrier of *cis*–*trans* amide isomerization in cyclic tetrapeptides such that these conformations are distinct by NMR and observable on the NMR time scale. This is in contrast to conformationally homogeneous α_3_β macrocycles with tertiary amides but do not possess α,β-disubstitution at the β-residue. These findings are significant because they delineate the phenomenon of conformational switching in cyclic tetrapeptides and further provide a strategy to control their conformations, whether through a flexible *N*-acyl aziridine moiety or by torsional strain, which can be applicable to not just cyclic peptides but other macrocyclic molecules as well.

## Acknowledgements

We would like to acknowledge the Natural Sciences and Engineering Research Council of Canada (NSERC), Canadian Institute for Health Research (CIHR), and the Ontario Graduate Scholarship Program (B. C.) for their financial support. We would also like to thank Dr D. Burns and D. Pichugin for their assistance with NMR spectroscopic experiments.
