# Supplementary material for: The reactivity and conformational control of cyclic tetrapeptides derived from aziridine-containing amino acids
Source: Chem Sci. 2016 Jun 30;7(11):6662–8. doi: 10.1039/c6sc01687a (PMC5450523; doi:10.1039/c6sc01687a)

Electronic Supplementary Material (ESI) for Chemical Science.
This journal is © The Royal Society of Chemistry 2016

The reactivity and conformational control of cyclic tetrapeptides derived from aziridine-containing amino acids

*Benjamin K. W. Chung, Christopher J. White, Conor C. G. Scully, and Andrei K. Yudin**

Davenport Research Laboratories, Department of Chemistry,

University of Toronto, 80 St. George Street, Toronto, Ontario, Canada M5S 3H6

Supporting Information

Table of Contents

1. General Information 2
2. General Procedures 3
3. List of Compounds 5
4. NMR Spectra 35
5. Exchange Spectroscopy (EXSY) to Quantify Conformational Exchange 95

1. General Information

1.1. Reagents

2-chlorotrityl chloride resin, coupling reagents, and all Fmoc-protected amino acids were purchased from AAPPTec Inc. (Louisville, KY, U.S.A.). All other commercial reagents and solvents were purchased from commercial sources and used as received, unless otherwise noted.

1.2. Chromatography

Flash column chromatography was carried out using Silicycle Inc. (Quebec City, QC, Canada) 230-400 mesh silica gel, unless otherwise noted. Thin-layer chromatography (TLC) was performed on Macherey Nagel pre-coated glass backed TLC plates (SIL G/UV254, 0.25 mm) and visualized using a UV lamp (254 nm) or potassium permanganate. All reactions involving chemical transformations on peptides were analyzed using analytical high-performance liquid chromatography-mass spectrometry (HPLC/MS) with an Agilent Technologies 1200 Series liquid chromatography system equipped with G1312A Binary Pump, G1329A Thermostatted Autosampler, G1316A Column Thermostat, G1314D Variable Wavelength Detector (UV detection at 214 nm), and G6130B Single Quadrupole LC/MS. Analytical HPLC/MS separations were performed with an Agilent Poroshell 120 EC-C18 2.7 µm, 4.6 x 50 mm column. The method used eluents A (water with 0.1% formic acid) and B (acetonitrile with 0.1% formic acid). The method started at 5% B for 0 – 1 min, then a linear gradient of 5% - 95% B for 1 – 8 min, followed by a wash with 95% B for 8 – 9 min, and finally an equilibration at 5% B from 9 – 10.5 min.

Unless otherwise stated, purification of final peptide products were performed either by semi-preparative HPLC/MS or by reverse-phase using a CombiFlash® purification system. For semi-preparative HPLC/MS, an Agilent 1260 Infinity Series liquid chromatography system equipped with G1311A Quaternary Pump, G1310A Isocratic Pump, two G1364C Automatic Fraction Collectors, G2258A Dual Loop Autosampler PS, G1315C Diode Array Detector (UV detection at 214 nm), and G6130B Single Quadrupole LC/MS was used. Semi-preparative HPLC/MS separations were performed with a Phenomenex® (Torrance, CA, U.S.A.) Jupiter 4µ Proteo 90A 4 µm, 10.0 x 250 mm column. For reverse-phase CombiFlash® purifications, a RediSep Rf Gold® C18 30 g column was used on a Teledyne ISCO Combiflash® Rf 200 at room temperature with a flow rate of 30 mL/min. For both systems, the method used eluents A (water with 0.1% formic acid) and B (acetonitrile with 0.1% formic acid). The method started at xx% B followed by a linear gradient of xx – yy% B over 25 minutes. Finally, the column was washed with 95% B for 5 minutes followed by equilibration in 5% B for 2 minutes. (xx and yy are defined individually for each compound.) The fractions containing the desired product (as determined by analytical HPLC-MS) were combined, frozen at -78 °C, and lyophilized to yield the final product.

To determine xx and yy, a series of consecutive isocratic methods were performed according to the analytical HPLC-MS method described above, where instead of the linear gradient of 5 – 95% B, an isocratic flow of 10 to 90% B in 10% increments was used instead (nine analytical HPLC runs in total). The value for xx is equal to the isocratic flow in which the desired compound has a retention time of approximately 8 – 9 min, ie, when the compound is eluted from the column just prior to the 95% flush.

The value for yy is equal to the isocratic flow in which the desired compound has a retention time of 2 – 3 min, ie, when the compound is eluted from the column shortly after reaching the isocratic flow value.

1.3. Nuclear Magnetic Resonance Spectroscopy

Proton (^{1}H NMR), carbon (^{13}C NMR), gCOSY, zTOCSY, gc2HSQCse, and gcHMBC spectra were recorded on Varian Mercury 200, 400 MHz or Agilent 500, 600, or 700 MHz spectrometers. All pulse sequences were used as provided. All NMR spectra were Fourier transformed, manually phase corrected, and auto baseline corrected using a Whittaker Smoother method in MestReNova v. 8.0.1 (Santiago de Compostela, Spain). ^{1}H NMR spectra chemical shifts (δ) are reported in parts per million (ppm) and were referenced to $CDCl_3$ (δ 7.26 ppm), CD_3OD (δ 3.30 ppm), or DMSO-d_6 (δ 2.50 ppm). Spectral data is reported as follows: chemical shift, multiplicity (s = singlet, br s = broad singlet; d = doublet, t = triplet, q = quartet, dd = doublet of doublets, dt = doublet of triplets, ddt = doublet of doublet of triplets, dtd= doublet of triplet of doublets, m = multiplet, br = broad), coupling constant (*J*) in Hertz (Hz), and integration. ^{13}C NMR spectra chemical shifts (δ) are reported in parts per million (ppm) and were referenced to $CDCl_3$ (δ 77.2 ppm), CD_3OD (δ 49.0 ppm), or DMSO-d_6 (δ 39.52 ppm).

1.4. High-Resolution Mass Spectrometry

High resolution mass spectra were obtained on an ABI/Sciex™ (Framingham, MA, U.S.A.) Qstar mass spectrometer with ESI source, MS/MS and accurate mass capabilities.

2. General Procedures

2.1. Solid-Phase Peptide Synthesis

Unless otherwise noted, linear aziridine-containing peptides were prepared on 2-chlorotrityl chloride resin (theoretical loading of 0.84 mmol g^{-1}) using *N*-Fmoc-amino acids. 2-chlorotrityl chloride resin was loaded with the C-terminal *N*-Fmoc-amino acid according to a literature procedure[1]. Solid-phase peptide synthesis was performed manually using 20 mL solid-phase extraction tubes with frit and cap using a solid-phase extraction vacuum manifold. Fmoc-deprotection steps for amino acids and peptides without an Azy derivative on the peptide chain was performed by suspending the resin in ~10 mL of 20% piperidine in *N*,*N*-dimethylformamide (DMF) and agitation for 10 minutes, repeated one additional time. Fmoc-deprotection steps for peptides containing an Azy derivative on the peptide chain was performed by suspending the resin in ~10 mL of 1% 1,8-diazabicyclo[5.4.0]undec-7-ene (DBU) in DMF and agitation for 1 minute, repeated one additional time. Coupling steps were performed as single couplings by dissolving 3 eq. of *N*-Fmoc-amino acid and 3 eq. of 1-[Bis(dimethylamino)methylene]-1H-1,2,3-triazolo[4,5-b]pyridinium 3-oxid hexafluorophosphate (HATU) in ~10 mL DMF, followed by addition of 6 eq. of *N*,*N*-diisopropylethylamine (DIPEA) and immediate addition to the peptide on resin, followed by agitation for 1 hour. Following each coupling and deprotection step, the resin was washed alternately with

[1] Barlos, K.; et al., *Tetrahedron Lett.*, **1989**, *30*, 3943-3946.

dichloromethane (DCM) and DMF, repeated two additional times. A final wash step was performed at the end of the synthesis using the standard resin wash procedure with an additional rinse with DCM at the end. Peptides were cleaved from resin by addition of 5mL of 25% 1,1,1,3,3,3-hexafluoropropan-2-ol (HFIP) in dichloromethane (DCM) then agitated for 5 minutes, then drained into a 20 mL scintillation vial, repeated two additional times. This was followed by a rinse and drain with 5 mL 25% HFIP in DCM without shaking. The cleavage mixtures were concentrated *in vacuo* to approximately 3 – 5 mL in volume by rotary evaporation, and immediately precipitated by addition of 20 mL of *tert*-butyl methyl ether (TBME). The vial was then centrifuged at 1600 rpm, followed by decantation of the excess TBME. The residual solvent was removed with N_2 stream and the peptides were stored overnight under reduced pressure in a dessicator containing anhydrous calcium sulfate and 3% cobalt(II) chloride (Drierite™) and subsequently at -20 °C.

2.2. Cyclization of Aziridine-Containing Linear Tetrapeptides

HATU (1.2 eq.) and 1-hydroxy-7-azabenzotriazole (HOAt, 1 eq.) were dissolved in anhydrous DMF (2.5 mM) under an atmosphere of dry nitrogen with vigorous stirring. Linear aziridine-containing tetrapeptide (1 eq.) was dissolved separately in anhydrous DMF (0.02 M) in a scintillation vial (complete dissolution was achieved by sonication, if necessary). In another separate scintillation vial, a solution of DIPEA (2.5 eq.) in anhydrous DMF (0.05 M) was prepared. These two solutions were taken up separately into two syringes each and simultaneously added to the reaction solution with HATU and HOAt with the aid of a syringe pump (flow rate: 0.02mL/min). After complete addition, the solution was stirred vigorously for one hour and the solvents were removed *in vacuo*, not exceeding a water bath temperature of 50 °C. The cyclic peptide was isolated by one of two methods. For use in subsequent reactions, the peptide was isolated by dissolving the residue in a minimal amount of DMF and precipitating from ice-cold MeCN : H_2O (1 : 9, v/v), followed by isolation using vacuum filtration. To obtain an analytically pure sample of peptide, the residue was dissolved in a minimal amount of water, acetonitrile and propan-2-ol, then applied to either semi-preparative HPLC/MS or reverse-phase Combiflash® purification as described in section 1.2.

2.3. Aziridine Ring-Opening of Aziridine-Containing Cyclic Tetrapeptides

2.3.1. Aziridine Ring-Opening with Sodium Azide

Cyclic tetrapeptide (1 eq.) and sodium azide (5 eq.) were suspended in anhydrous DMF (0.05 M). The reaction was heated to 60 °C and stirred at this temperature under an atmosphere of dry nitrogen for 24 hours. The solution was then cooled to room temperature and the solvent was removed to afford a residue that was triturated with water. The product was then isolated by filtration and dried *in vacuo*. If the peptide is soluble in water, and/or an analytically pure sample is required, the peptide is dissolved in a minimal amount of water, acetonitrile, and propan-2-ol, then applied to either semi-preparative HPLC/MS or reverse-phase Combiflash® purification as described in section 1.2.

2.3.2. Aziridine Ring-Opening with Thiophenol

Cyclic tetrapeptide (1 eq.), thiophenol (20 eq.) and DIPEA (20 eq.) were suspended in anhydrous DMF (0.05 M). The reaction stirred at room temperature under an atmosphere of dry nitrogen for 24 hours. The solvent was removed to afford a residue that was triturated with 1:1 methanol:water to remove most of the excess thiophenol. The peptide was then dissolved in a minimal amount of water, acetonitrile, and propan-2-ol, then applied to reverse-phase Combiflash® purification as described in section 1.2.

3. List of Compounds

3.1. Fmoc-Azy-OH and Derivatives

3.1.1. Preparation of Fmoc-Azy-OH

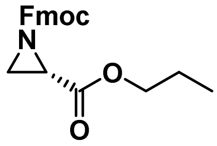

1-((9*H*-fluoren-9-yl)methyl) 2-propyl (*S*)-aziridine-1,2-dicarboxylate

Starting aziridine *n*-propyl ester (2.30 g, 17.8 mmol, 1 eq. prepared using reported procedures) was dissolved in 1,4-dioxane (89 mL, 0.2 M) and cooled with stirring on ice. *N*,*N*-diisopropylethylamine (3.1 mL, 17.8 mmol, 1 eq.) was added. Fmoc-OSu (6.00 g, 17.8 mmol, 1 eq.) was separately dissolved in 1,4-dioxane (14.8 mL, 1.2 M) and added dropwise to the mixture containing the aziridine ester. The resulting mixture was warmed to room temperature with stirring overnight. The reaction was then transferred to a separatory funnel and diluted with approximately 200 mL of ethyl acetate. The mixture was washed with saturated citric acid (10 mL), followed by brine (100 mL). The organic extract was dried with $Na_2SO_{4(s)}$, filtered, and concentrated onto celite by rotary evaporation. The celite mixture was then subjected to Combiflash purification using an 80g gold silica gel column with a flow rate of 60 mL/min and total run time of 40 minutes, using 100% hexanes from 0 – 5 min, then a gradient of 0 – 20% ethyl acetate in hexanes from 5 – 12.5 min, and finally 20% ethyl acetate in hexanes from 12.5 min – the end of the purification. This yielded 5.36 g of 1-((9*H*-fluoren-9-yl)methyl) 2-propyl (*S*)-aziridine-1,2-dicarboxylate as a white solid in 86% yield. ^{1}H NMR (400 MHz, $CDCl_3$) δ 7.77 (d, *J* = 7.5 Hz, 2H), 7.61 (dd, *J* = 7.4, 1.6 Hz, 2H), 7.46 – 7.36 (m, 2H), 7.32 (tt, *J* = 7.4, 1.3 Hz, 2H), 4.46 (dd, *J* = 10.5, 7.3 Hz, 1H), 4.37 (dd, *J* = 10.5, 7.2 Hz, 1H), 4.25 (t, *J* = 7.2 Hz, 1H), 4.18 – 4.03 (m, 2H), 3.10 (dd, *J* = 5.4, 3.2 Hz, 1H), 2.62 (dd, *J* = 3.2, 1.3 Hz, 1H), 2.46 (dd, *J* = 5.4, 1.3 Hz, 1H), 1.67 (h, *J* = 7.2 Hz, 2H), 0.95 (t, *J* = 7.4 Hz, 3H).

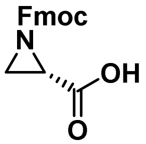

(*S*)-1-(((9*H*-fluoren-9-yl)methoxy)carbonyl)aziridine-2-carboxylic acid

1-((9*H*-fluoren-9-yl)methyl) 2-propyl (*S*)-aziridine-1,2-dicarboxylate (1.00 g, 2.85 mmol, 1 eq.) was dissolved in THF:water (25 mL each, 0.057 M) and cooled with stirring on ice. LiOH•H_2O (0.24 g, 5.70 mmol, 2 eq.) was added and the reaction was stirred for 20 minutes. The reaction was then transferred to an Erlenmeyer flask, and 1M $HCl_{(aq)}$ was added until pH = 1. The organic layer was separated and washed once with brine (100 mL). The organic extract was dried with $Na_2SO_{4(s)}$, filtered, and concentrated to a volume of 50 mL. The reaction was diluted with DCM (50 mL) and the entire volume was loaded onto a silica column preconditioned with 1:1 DCM:EtOAc. The column was washed with three column volumes of DCM:EtOAc (1:1), and eluted by eight column volumes of DCM:MeOH (4:1). The fractions containing the desired product were combined and evaporated to ~70 mL. At this point, THF (100 mL) was added, and concentrated until the volume was once again ~70 mL. This THF addition/evaporation sequence was repeated once more. Water (70 mL) was then added, and the mixture was frozen on dry ice/acetone and lyophilized. This yielded 628 mg of (*S*)-1-(((9*H*-fluoren-9-yl)methoxy)carbonyl)aziridine-2-carboxylic acid as a white solid in 79% yield. ^{1}H NMR (500 MHz, CD_3OD) δ 7.78 (dt, *J* = 7.7, 0.9 Hz, 2H), 7.66 – 7.61 (m, 2H), 7.38 (tt, *J* = 7.6, 0.8 Hz, 2H), 7.33 – 7.28 (m, 2H), 4.46 – 4.30 (m, 2H), 4.23 (t, *J* = 6.9 Hz, 1H), 2.99 (dd, *J* = 5.5, 3.1 Hz, 1H), 2.47 – 2.39 (m, 1H), 2.36 (dd, *J* = 5.4, 1.5 Hz, 1H).

3.1.2. Preparation of Fmoc-Cma-OH

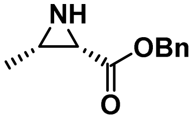

Benzyl (2*S*,3*S*)-3-methylaziridine-2-carboxylate

N-Trt-*cis*-3-Me-Azy-OBn was obtained through previously reported methods. *N*-Trt-*cis*-3-Me-Azy-OBn (20 g, 46.1 mmol, 1 eq.) and triethylsilane (14.7 mL, 92.3 mmol, 2 eq.) were dissolved in DCM (230.5 mL, 0.2 M). The resulting mixture was cooled on ice/brine. Trifluoroacetic acid (14.1 mL, 184.5 mmol, 4 eq.) was then added dropwise by addition funnel over 30 minutes. Following TFA addition, the mixture was stirred for an additional 30 minutes. The mixture was concentrated under reduced pressure, and redissolved in diethyl ether (250 mL). The product was extracted into deionized water (twice with 50 mL each, twice more with 25 mL each). The aqueous layers were combined and washed with diethyl ether (25 mL) and collected in a 500 mL Erlenmeyer flask. Solid sodium bicarbonate was added until pH ≈ 9 as indicated by pH paper. The aqueous portion was then added into a continuous extractor, followed by 150 mL of chloroform. The continuous extractor was attached to a 500 mL round bottom flask containing 250 mL of chloroform. The round bottom flask was then heated in an oil bath at 70 °C overnight. The organic extracts were then combined, dried with anhydrous Na_2SO_4, filtered, and concentrated under reduced

pressure to yield benzyl (2*S*,3*S*)-3-methylaziridine-2-carboxylate as a white solid in 61% yield. ^{1}H NMR (200 MHz, $CDCl_3$) δ 7.46 – 7.28 (m, 5H), 5.21 (s, 2H), 2.68 (d, *J* = 6.1 Hz, 1H), 2.31 (p, *J* = 5.8 Hz, 1H), 1.29 (d, *J* = 5.7 Hz, 3H).

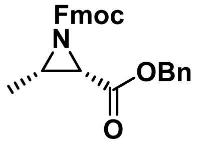

1-((9*H*-fluoren-9-yl)methyl) 2-benzyl (2*S*,3*S*)-3-methylaziridine-1,2-dicarboxylate

Benzyl (2*S*,3*S*)-3-methylaziridine-2-carboxylate was converted into an Fmoc carbamate using Fmoc-OSu via an identical procedure for Fmoc-Azy-O^{n}Pr (section 3.1.1) to yield 1-((9*H*-fluoren-9-yl)methyl) 2-benzyl (2*S*,3*S*)-3-methylaziridine-1,2-dicarboxylate as a white solid in 88% yield. ^{1}H NMR (400 MHz, $CDCl_3$) δ 7.75 (ddt, *J* = 7.7, 2.6, 0.8 Hz, 2H), 7.58 (ddt, *J* = 7.6, 2.8, 0.9 Hz, 2H), 7.44 – 7.33 (m, 7H), 7.30 (ddd, *J* = 8.6, 7.5, 1.2 Hz, 2H), 5.33 – 5.15 (m, 2H), 4.42 (d, *J* = 7.0 Hz, 2H), 4.23 (t, *J* = 7.0 Hz, 1H), 3.11 (d, *J* = 6.7 Hz, 1H), 2.69 (dq, *J* = 6.8, 5.6 Hz, 1H), 1.34 (d, *J* = 5.6 Hz, 3H).

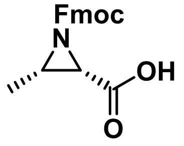

(2*S*,3*S*)-1-(((9*H*-fluoren-9-yl)methoxy)carbonyl)-3-methylaziridine-2-carboxylic acid

1-((9*H*-fluoren-9-yl)methyl) 2-benzyl (2*S*,3*S*)-3-methylaziridine-1,2-dicarboxylate (1 g, 2.4 mmol, 1 eq.) and 10% Pd on C (242 mg, 0.1 g/mmol ester) were placed into an oven-dried round bottom flask that has been purged with N_2. 1,4-dioxane (48 mL, 0.05 M) was added, followed by attachment of a H_2 balloon attached to a long needle that is submerged into the reaction solvent. With stirring, the flask was purged with a H_2 balloon while attached to a bubbler, purging at approximately 1 bubble/s. When the balloon had emptied, it was replaced with another full H_2 balloon. The resulting reaction was stirred for two hours at room temperature. The mixture was then filtered through a fritted funnel containing celite. The filter cake was washed with ethyl acetate until the filtrate showed no more UV active spot on TLC (~300 mL). The filtrate was then concentrated to approximately 50 mL, at which point it was diluted with 50 mL of hexanes and applied onto a silica gel column equilibrated with 1:1 EtOAc:hexanes. The column was then washed with 1:1 EtOAc:hexanes,, followed by 9:1 EtOAc:hexanes, and finally the product was eluted with 4:1 EtOAc:MeOH. The fractions containing the desired compound were combined and concentrated to approximately 50 mL. 100 mL of THF was added, and concentrated to approximately 50 mL once again. This process was repeated one more time. Following this, 50 mL of THF, 50 mL of MeCN, and 100 mL of deionized water was added. The solution was frozen on dry ice/acetone, and lyophilized to yield (2*S*,3*S*)-1-(((9*H*-fluoren-9-yl)methoxy)carbonyl)-3-methylaziridine-2-carboxylic acid as a white powder in 78% yield. ^{1}H NMR (400 MHz, DMSO-d_6) δ 7.90 (d, *J* = 7.4 Hz, 2H), 7.65 (dd, *J* = 7.5, 4.0 Hz, 2H), 7.43 (t, *J* = 7.5 Hz, 2H), 7.34 (tt, *J* = 7.3, 1.4 Hz, 2H), 4.41 – 4.29 (m, 2H), 4.26 (dd, *J* = 14.3, 7.4 Hz, 1H), 2.80 (d, *J* = 7.0 Hz, 1H), 2.47 (dt, *J* = 11.3, 5.8 Hz, 1H), 1.21 (d, *J* = 5.5 Hz, 3H). ^{13}C NMR (101 MHz, DMSO-d_6) δ 162.1, 151.5, 143.6, 140.7, 139.4, 137.4, 128.9, 127.7, 127.3, 127.1, 125.1, 124.9, 121.4, 120.0, 109.7, 66.9, 46.5, 30.4, 12.9.

3.1.3. Preparation of Fmoc-Tma-OH

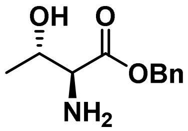

Benzyl L-allothreoninate

Allo-L-threonine (26.9 g, 226 mmol, 1 eq.) was suspended in BnOH:benzene (4:1, 180 mL, 1M), followed by the addition of *p*-TsOH monohydrate (47.2 g, 248 mmol, 1.1 eq.). The round-bottomed flask was fitted with a Dean-Stark apparatus and reflux condenser, and the reaction was refluxed at 110 °C for 24 h. The flask was then cooled to room temperature and concentrated by rotary evaporation. Water (~180 mL) was added, and the aqueous layer was washed twice with ethyl acetate (~180 mL each). The organic layers were combined and back extracted with water (4 x 90 mL). The aqueous layers were combined and adjusted to pH > 9 using KOH pellets. The aqueous layers were then extracted with ethyl acetate (5 x 180 mL). The organic layers were combined, dried over $Na_2SO_{4(s)}$, filtered, and concentrated under reduced pressure to give 10.4 g of benzyl L-allothreoninate as a clear, colourless oil in 22% yield. ^{1}H NMR (400 MHz, $CDCl_3$) δ 7.43 – 7.25 (m, 5H), 5.18 (d, *J* = 2.7 Hz, 2H), 4.70 (s, 1H), 4.03 (qd, *J* = 6.4, 4.7 Hz, 1H), 3.59 (d, *J* = 4.8 Hz, 1H), 1.92 (br s, 2H), 1.06 (d, *J* = 6.4 Hz, 3H). ^{13}C NMR (100 MHz, $CDCl_3$) δ 173.7, 140.9, 135.4, 128.7, 128.6, 128.5, 128.4, 127.7, 127.0, 68.1, 67.0, 65.4, 59.1, 18.0.

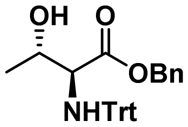

Benzyl trityl-L-allothreoninate

In a round-bottomed flask, benzyl L-allothreoninate (10.4 g, 49.8 mmol, 1 eq.) was dissolved in DCM (40 mL) and cooled in an ice bath. Triethylamine (13.9 mL, 99.7 mmol, 2 eq.) was added dropwise to the reaction by syringe pump. After trimethylamine addition, trityl chloride (13.9 g, 49.8 mmol, 1 eq.) was dissolved in DCM (43 mL, 0.6 M final concentration wrt benzyl L-allothreoninate) and added dropwise to the reaction by syringe pump. The reaction was then stirred at 4 °C overnight. Following this, the reaction was filtered, and the filtrate concentrated under reduced pressure. The residue was resuspended in EtOAc (100 mL), and washed twice with aqueous ice-cold citric acid (0.5 M), twice with water, and once with brine (50 mL each). The organic layer was dried over $Na_2SO_{4(s)}$, filtered, and concentrated under reduced pressure to give 22.5 g of benzyl trityl-L-allothreoninate as a white solid in quantitative yield. ^{1}H NMR (400 MHz, $CDCl_3$) δ 7.55 – 7.47 (m, 5H), 7.44 – 7.13 (m, 15H), 4.73 (d, *J* = 12.3 Hz, 1H), 4.49 (d, *J* = 12.2 Hz, 1H), 3.94 (pd, *J* = 6.6, 3.9 Hz, 1H), 3.45 (dd, *J* = 10.0, 3.8 Hz, 1H), 3.06 (d, *J* = 10.0 Hz, 1H), 2.36 (d, *J* = 6.8 Hz, 1H), 1.08 (d, *J* = 6.6 Hz, 3H). ^{13}C NMR (100 MHz, Chloroform-*d*) δ 172.6, 145.7, 144.1, 135.3, 128.9, 128.7, 128.5, 128.3, 128.3, 128.3, 127.9, 127.8, 127.0, 127.0, 127.0, 126.6, 71.0, 70.0, 66.7, 61.1, 19.6.

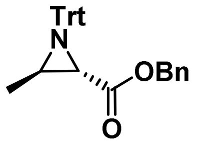

Benzyl (2*S*,3*R*)-3-methyl-1-tritylaziridine-2-carboxylate

Benzyl trityl-L-allothreoninate (22.5 g, 49.8 mmol, 1 eq.) was dissolved in anhydrous dichloromethane (100 mL, 0.5 M), placed under an atmosphere of dry nitrogen and cooled to 0 °C in an ice bath. Methanesulfonyl chloride (4.6 mL, 59.8 mmol, 1.2 eq.) was then added followed by the drop-wise addition of triethylamine (10.4 mL, 74.7 mmol, 1.5 eq.). The resulting orange suspension was stirred at 0 °C for 30 minutes. The ice bath was removed and the solution was diluted with dichloromethane (100 mL). The solution was subsequently washed with ice-cold 10% aqueous citric acid (100 mL) and water (100 mL). The organic layer was dried over Na_2SO_4, filtered and concentrated *in vacuo* to give the crude meslyate as a yellow-orange oil. This residue was dissolved in anhydrous tetrahydrofuran (180 mL) and triethylamine (15.3 mL, 110 mmol, 2.2 eq.) was then added. The resulting solution was heated to 70 °C for 3 days. The solution was then cooled and concentrated *in vacuo*. The resulting residue was dissolved in ethyl acetate (200 mL) and washed with ice-cold 10% aqueous citric acid (2 x 100 mL), water (2 x 100 mL) and saturated aqueous sodium chloride (1 x 100 mL). The organic layer was then dried over Na_2SO_4, filtered and concentrated to ~100 mL and left to stand open to the air in an Erlenmeyer flask overnight with a gentle stream of dry nitrogen. The resulting crystals were isolated by filtration and dried *in vacuo*, yielding 14.7 g of benzyl (2*S*,3*R*)-3-methyl-1-tritylaziridine-2-carboxylate as a crystalline white solid in 68% yield. ^{1}H NMR (400 MHz, $CDCl_3$) δ 7.51 – 7.47 (m, 5H), 7.35 – 7.17 (m, 15H), 5.23 – 5.07 (m, 2H), 3.01 (qd, J = 6.2, 2.4 Hz, 1H), 2.40 (d, J = 2.3 Hz, 1H), 0.59 (d, J = 6.1 Hz, 3H). ^{13}C NMR (101 MHz, $CDCl_3$) δ 171.8, 146.9, 145.6, 135.9, 129.7, 129.0, 128.8, 128.8, 128.6, 128.6, 128.4, 128.2, 128.0, 127.6, 127.3, 126.8, 73.1, 66.6, 42.0, 38.4, 13.8.

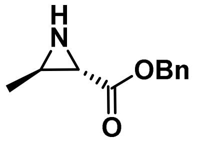

Benzyl (2*S*,3*R*)-3-methylaziridine-2-carboxylate

Benzyl (2*S*,3*R*)-3-methyl-1-tritylaziridine-2-carboxylate (14.7 g, 33.9 mmol) was subjected to trityl deprotection using TFA according to an identical procedure for Trt-Cma-OBn (Section 3.1.2). This yielded 4.8 g of H-Tma-OBn benzyl (2*S*,3*R*)-3-methylaziridine-2-carboxylate as a crystalline yellow-white solid in 74% yield. ^{1}H NMR (400 MHz, $CDCl_3$) δ 7.40 – 7.33 (m, 5H), 5.24 – 5.11 (m, 2H), 2.36 – 2.28 (m, 2H), 1.23 (d, J = 5.3 Hz, 3H). ^{13}C NMR (100 MHz, $CDCl_3$) δ 143.9, 129.5, 128.7, 128.3, 126.3, 67.3, 56.9, 36.2, 35.0, 18.1.

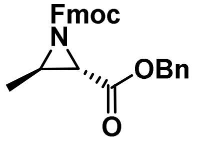

1-((9*H*-fluoren-9-yl)methyl) 2-benzyl (2*S*,3*R*)-3-methylaziridine-1,2-dicarboxylate

Fmoc-protection of benzyl (2*S*,3*R*)-3-methylaziridine-2-carboxylate (4.8 g, 25.2 mmol) was performed using FmocOSu according to an identical procedure for H-Cma-OBn (Section 3.1.2), to give 6.3 g of 1-((9*H*-fluoren-9-yl)methyl) 2-benzyl (2*S*,3*R*)-3-methylaziridine-1,2-dicarboxylate as a white solid in 60% yield. ^{1}H NMR (400 MHz, $CDCl_3$) δ 7.76 (ddt, *J* = 7.6, 2.1, 0.9 Hz, 2H), 7.58 (ddd, *J* = 7.6, 3.4, 1.1 Hz, 2H), 7.44 – 7.34 (m, 4H), 7.33 – 7.26 (m, 5H), 5.16 – 5.07 (m, 2H), 4.34 (ddd, *J* = 77.0, 10.4, 7.1 Hz, 2H), 2.93 (dd, *J* = 2.6, 1.3 Hz, 2H), 1.32 (dd, *J* = 4.9, 1.8 Hz, 3H). ^{13}C NMR (100 MHz, $CDCl_3$) δ 171.1, 168.1, 160.1, 143.7, 141.3, 134.9, 128.7, 128.6, 128.6, 128.5, 127.8, 127.8, 127.1, 125.2, 125.1, 120.0, 120.0, 68.4, 68.0, 67.6, 67.0, 65.4, 61.1, 60.4, 47.2, 46.9, 41.6, 40.0, 21.1, 16.5, 16.0, 14.2.

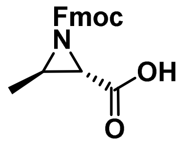

(2*S*,3*R*)-1-(((9*H*-fluoren-9-yl)methoxy)carbonyl)-3-methylaziridine-2-carboxylic acid

1-((9*H*-fluoren-9-yl)methyl) 2-benzyl (2*S*,3*R*)-3-methylaziridine-1,2-dicarboxylate (1.0 g, 2.4 mmol) was subjected to hydrogenation using H_2, 10% Pd/C using a previously reported analogous procedure for Fmoc-Cma-OBn (Section 3.1.2) to give 383 mg of (2*S*,3*R*)-1-(((9*H*-fluoren-9-yl)methoxy)carbonyl)-3-methylaziridine-2-carboxylic acid as a white solid in 49% yield after purification by silica gel column chromatography. ^{1}H NMR (500 MHz, MeOD) δ 7.78 (dt, *J* = 7.5, 0.9 Hz, 2H), 7.67 – 7.62 (m, 2H), 7.38 (dddd, *J* = 8.2, 7.5, 1.2, 0.6 Hz, 2H), 7.30 (tdd, *J* = 7.4, 2.3, 1.1 Hz, 2H), 4.41 – 4.32 (m, 2H), 2.78 – 2.67 (m, 1H), 2.21 (m, 1H), 1.25 – 1.21 (m, 3H). ^{13}C NMR (126 MHz, MeOD) δ 143.9, 143.7, 141.1, 127.4, 126.7, 124.9, 124.9, 124.7, 119.5, 67.9, 38.4, 34.0, 29.5, 15.3.

3.2. Precursors to and Derivatives of Linear Azy-containing Tetrapeptides Prepared In Solution

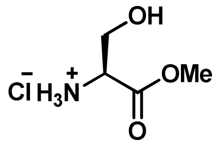

(*S*)-3-Hydroxy-1-methoxy-1-oxopropan-2-aminium chloride

A 1000 mL, flame-dried, one-necked, round-bottomed flask was equipped with a magnetic stirring bar and charged with anhydrous methanol (170 mL, 1.18 M). The flask was then cooled to 0 °C in an ice bath for 15 minutes with stirring. Thionyl chloride (44 mL, 600 mmol) was then added drop-wise from an addition funnel over 30 minutes. The solution was stirred for an additional 30 minutes at 0 °C. The addition funnel was then removed and, to the reaction flask, was added L-serine **2.30** (21.0 g, 200 mmol) as a solid in one portion. The round-bottomed flask was then fitted with a flame-dried reflux condenser protected from moisture by a calcium chloride-filled drying tube and the ice bath was replaced with an oil bath. The stirred suspension was then heated to 60 °C and stirred at this temperature for 8 hours. The resulting clear solution was then cooled to room temperature and concentrated *in vacuo*. Here, the relatively large 1000 mL flask is necessary to prevent the product from bumping into the rotary

evaporator. The resulting crystalline solid was triturated with diethyl ether (400 mL) and the product was isolated by vacuum filtration. The product was then washed with addition diethyl ether (3 x 200 mL) and dried overnight in a vacuum desiccator over a bed of Drierite. This yielded 30.4 – 31.1 g of (*S*)-methyl serinate hydrochloride ((*S*)-3-Hydroxy-1-methoxy-1-oxopropan-2-aminium chloride, 195 – 200 mmol, 98 - 99% yield) as a white, crystalline solid that is used without further purification. mp = 159 – 161 °C (MeOH); $[\alpha]^{27}$ +13.3° (MeOH, *c* 1.0); IR (neat) cm^{-1}: 3345, 2922, 1745, 1592, 1509, 1443, 1240, 1093, 1038, 966, 900; ^{1}H NMR (400 MHz, d_4-MeOH) δ: 3.85 (s, 3H), 3.95 (ddd, *J* = 11.8, 3.5, 0.5 Hz, 1H), 4.01 (ddd, *J* = 11.8, 4.4, 0.5 Hz, 1H), 4.15 (dd, *J* = 4.4, 3.5 Hz, 1H); ^{13}C NMR (100 MHz, d_4-MeOH) δ: 53.7, 56.1, 60.7, 169.3; HR-MS: *m/z* = 120.0655 $[M+H]^+$, calcd. 120.0656 for $C_4H_{10}NO_3$.

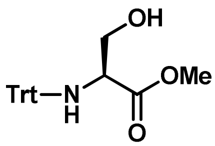

(*S*)-Methyl 3-hydroxy-2-(tritylamino)propanoate

A 1000 mL, flame-dried, 2-necked, round-bottomed flask was equipped with a magnetic stirring bar and charged with (*S*)-methyl serinate hydrochloride (25.0 g, 160 mmol) and anhydrous dichloromethane (100 mL, 1.6 M). The flask was then fitted with a flame-dried, 60 mL and a 250 mL pressure-equalizing addition funnel, which were sealed with rubber septa. The apparatus was maintained under an atmosphere of dry nitrogen during the course of the reaction. The resulting white suspension was stirred and cooled to 0 °C in an ice bath. The 60 mL addition funnel was charged with triethylamine (44.6 mL, 320 mmol) and then added drop-wise over 30 minutes. During this time, a flame-dried, 250 mL, one-necked, round-bottomed flask, equipped with an egg-shaped magnetic stirring bar was charged with trityl chloride (44.6 g, 160 mmol), dissolved in anhydrous dichloromethane (100 mL, 1.6 M), sealed with a rubber septum and placed under an atmosphere of dry nitrogen. This resulting yellow solution was then added to the 250 mL addition funnel of the reaction flask through the use of a cannula. To ensure a quantitative transfer, the 250 mL round-bottomed flask was washed with additional dichloromethane (3 x 15 mL), which was subsequently transferred to the addition funnel with the cannula. Once the triethylamine was completely added to the reaction flask, the trityl chloride solution was then immediately added drop-wise to the reaction over 1 hour. Once the addition is complete, both addition funnels were removed from the 1000 mL round-bottomed flask and replaced with rubber septa. The flask was then placed in a refrigerator and stirred at 4 °C for 20 hours. The flask was then warmed to room temperature and the resulting white suspension was vacuum filtered over a fritted funnel. The white precipitate was washed with additional dichloromethane (150 mL). The combined filtrate was concentrated *in vacuo* and then re-suspended in ethyl acetate (500 mL). This suspension was washed sequentially with ice-cold 10% aqueous citric acid (2 x 200 mL), water (2 x 200 mL) and saturated aqueous sodium chloride (1 x 200 mL). The organic layer was then collected and dried with anhydrous Na_2SO_4. The solution was then filtered into a flame-dried, 1000 mL, one-necked, round-bottomed flask by vacuum filtration, concentrated *in vacuo* and then dried under high vacuum to give 56.0 – 57.7 g of *N*-trityl-L-serine methyl ester ((*S*)-Methyl 3-hydroxy-2-(tritylamino)propanoate, 155 – 160 mmol, 97 - 99 % yield) as an off-white, crystalline solid that was used without further purification. mp = 140 – 149 °C (Ethyl acetate); $[\alpha]^{27}$ +3.4° ($CHCl_3$, *c* 1.0); IR (neat) cm^{-1}: 3453, 1701, 1596, 1491, 1445, 1424, 1207, 1171, 1056, 1011, 964, 893, 754, 697; ^{1}H NMR (400 MHz,

$CDCl_3$, solvent referenced to TMS at 0.00 ppm) δ: 3.29 (s, 3H), 3.60 – 3.50 (m, 2H), 3.70 (dd, *J* = 10.1, 4.0 Hz, 1H), 7.16 – 7.22 (m, 3H), 7.23 – 7.31 (m, 7H), 7.45 – 7.51 (m, 6H) (A minor trityl-based impurity overlaps in the aromatic region that offsets the integration); ^{13}C NMR (100 MHz, $CDCl_3$) δ: 52.1, 57.9, 65.1, 71.1, 126.7, 128.0, 128.8, 145.7, 174.1; HR-MS: *m/z* = : 384.1580 $[M+Na]^+$, calcd. 384.1570 for $C_{23}H_{25}NNaO_3$.

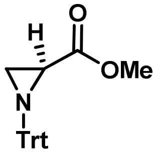

(*S*)-Methyl 1-tritylaziridine-2-carboxylate

N-trityl-L-serine methyl ester (56.0 g, 155 mmol) was placed in a round-bottomed flask with magnetic stirring bar was added anhydrous dichloromethane (310 mL, 0.5 M), sealed with a rubber septum, placed under an atmosphere of dry nitrogen and cooled to 0 °C in an ice bath. Methanesulfonyl chloride (14.4 mL, 186 mmol, 1.2 eq.) was then added followed by the drop-wise addition of triethylamine (32.4 mL, 233 mmol, 1.5 eq.). The resulting orange suspension was stirred at 0 °C for 30 minutes. The ice bath was removed and the solution was diluted with dichloromethane (300 mL). The solution was subsequently washed with ice-cold 10% aqueous citric acid (300 mL) and water (300 mL). The organic layer was dried over Na_2SO_4, filtered and concentrated *in vacuo* to give the crude meslyate as a yellow-orange oil. This residue was dissolved in anhydrous tetrahydrofuran (570 mL) and triethylamine (48.3 mL, 346 mmol, 2.2 eq.) was then added. The resulting solution was heated to 70 °C for 90 hours, at which point TLC analysis (solvent system = 10% ethyl acetate in hexanes) indicated complete consumption of the mesylate. The solution was then cooled back to room temperature and concentrated *in vacuo*. The resulting residue was dissolved in ethyl acetate (600 mL) and washed with ice-cold 10% aqueous citric acid (2 x 300 mL), water (2 x 300 mL) and saturated aqueous sodium chloride (1 x 300 mL). The organic layer was then dried over Na_2SO_4, filtered and concentrated *in vacuo* to yield an orange solid. The product was recrystallized from hot ethanol to give 48.4 g of (*S*)-Methyl 1-tritylaziridine-2-carboxylate (136 mmol, 88% yield) as off-white crystals. mp = 127 -130 °C (EtOH); $[\alpha]^{27}$ -88.1° ($CHCl_3$, *c* 1.0); IR (neat) cm^{-1}: 1742, 1595, 1490, 1448, 1394, 1233, 1198, 1179, 1087, 1018, 971, 783, 770, 751, 710, 697; ^{1}H NMR (400 MHz, $CDCl_3$, solvent referenced to TMS at 0.00 ppm) δ: 1.41 (dd, *J* = 6.2, 1.6 Hz, 1H), 1.89 (dd, *J* = 6.2, 2.7 Hz, 1H), 2.25 (dd, *J* = 2.8, 1.6 Hz, 1H), 3.75 (s, 3H), 7.18 – 7.31 (m, 9H), 7.42 – 7.58 (m, 6H); ^{13}C NMR (100 MHz, $CDCl_3$) δ: 28.8, 31.8, 52.2, 74.5, 127.1, 127.8, 129.4, 143.7, 172.1; HR-MS: *m/z* = : 366.1477 $[M+Na]^+$, calcd. 366.1464 for $C_{23}H_{21}NNaO_2$.

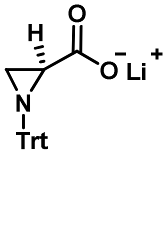

Lithium (*S*)-1-tritylaziridine-2-carboxylate

To a one-necked, round-bottomed flask with a magnetic stirring bar was added (*S*)-Methyl 1-tritylaziridine-2-carboxylate (6.87 g, 20 mmol) and was dissolved in acetonitrile (50 mL) with stirring in an ice bath at 0 °C. A solution of lithium hydroxide monohydrate (0.92 g, 22 mmol) was dissolved separately in water (20 mL) and this solution was subsequently added to the solution. The reaction mixture was capped and stirred at 0 °C for 5 hours, at which point TLC analysis (solvent system = 10% ethyl acetate in hexanes) indicated complete consumption of the methyl ester. The solution was then lyophilized for 48 hours to give lithium (*S*)-1-tritylaziridine-2-carboxylate as a fluffy white solid (6.71 g, 20 mmol) in a quantitative yield. This product may be used without further purification. ^{1}H NMR (400 MHz, d_4-MeOH) δ 7.71 – 7.02 (m, 15H), 2.04 (dd, *J* = 3.0, 1.8 Hz, 1H), 1.75 (dd, *J* = 6.3, 3.0 Hz, 1H), 1.23 (dd, *J* = 6.3, 1.8 Hz, 1H); ^{13}C NMR (101 MHz, d_4-MeOH) δ 179.8, 145.8, 130.8, 128.4, 127.7, 75.7, 35.2, 28.6; HR-MS: *m/z* = : 328.1340 [M-H]$^-$, calcd. 328.1343 for $C_{22}H_{18}NO_2$.

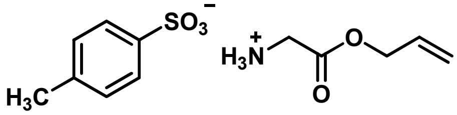

2-(Allyloxy)-2-oxoethanaminium 4-methylbenzenesulfonate

The reaction was performed on a 65 mmol (4.87 g) scale of glycine. Into a flame-dried, one-necked, round bottom flask equipped with a magnetic stirring bar was added glycine (1.0 equiv.) and TsOH · H_2O (1.1 equiv.). Benzene was was then added and the resulting suspension (130 mM) was stirred vigorously. Allyl alcohol (10 equiv.) was then added and the reaction flask was fitted with a Dean-Stark trap. The system was heated to 85 °C and stirred at this temperature with concomitant azeotropic removal of water. After 12 hours, TLC analysis (solvent system = 4 : 1, MeCN : H_2O, staining with ninhydrin) revealed complete consumption of glycine. The system was then cooled down to room temperature and the Dean-Stark trap removed. The Solvent was then removed *in vacuo* to yield a pale yellow viscous oil, from which the precipitated with cold diethyl ether. The product was isolated by vacuum filtration and after drying overnight under vacuum, yielded 2-(allyloxy)-2-oxoethanaminium 4-methylbenzenesulfonate (98%) as a white solid. The spectroscopic information of 2-(allyloxy)-2-oxoethanaminium 4-methylbenzenesulfonate was found to match that of what has previously been reported (see ref. 78). ^{1}H NMR (400 MHz, DMSO-d_6) δ 8.22 (s, 3H), 7.48 (d, *J* = 6.4 Hz, 2H), 7.12 (d, *J* = 7.7 Hz, 2H), 5.93 (ddt, *J* = 17.5, 10.7, 5.4 Hz, 1H), 5.38 (dd, *J* = 17.3, 1.6 Hz, 1H), 5.27 (dd, *J* = 10.7, 1.5 Hz, 1H), 4.72 – 4.66 (m, 2H), 3.88 (s, 2H), 2.29 (s, 3H); ^{13}C NMR (100 MHz, DMSO) δ 167.4, 145.4, 137.9, 131.8, 128.1, 125.5, 118.5, 65.7, 20.8.

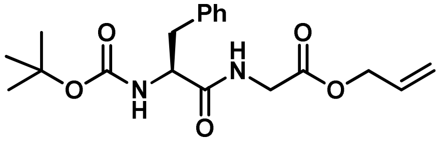

(*S*)-Allyl 2-(2-((tert-butoxycarbonyl)amino)-3-phenylpropanamido)acetate

This reaction was performed on a 32 mmol scale. *N*-Boc-phenylalanine (1.0 equiv.), glycine allyl ester tosylate (2-(allyloxy)-2-oxoethanaminium 4-methylbenzenesulfonate, 1.0 equiv.) and PyBOP (1.2 equiv.) were suspended in anhydrous $CHCl_3$ (1000 mM) under an atmosphere of dry nitrogen with stirring in a flame-dried, one-necked, round-bottomed flask. The solution was then cooled in an ice bath and to this was added *N,N*-diisopropylethylamine dropwise (3.0 equiv.). Once the addition was complete, the ice bath was removed and the solution was stirred at 23 °C for 2 hours. The chloroform was then removed *in vacuo* and the resulting residue was dissolved in ethyl acetate. The solution was then washed subsequently with 10% aqueous citric acid, saturated aqueous sodium bicarbonate, water and saturated aqueous sodium chloride. The organic layer was then dried with sodium sulfate, filtered and concentrated *in vacuo*. The residue was purified by silica-gel flash chromatography (43% ethyl acetate in hexanes to 75% ethyl acetate in hexanes) to afford the dipeptide (*S*)-allyl 2-(2-((tert-butoxycarbonyl)amino)-3-phenylpropanamido)acetate (97.5%) as a white, crystalline solid. R_f= 0.58 (75% ethyl acetate in hexanes). ^{1}H (400MHz, $CDCl_3$): δ = 7.36 – 7.14 (m, 5H), 6.40 (t, *J* = 4.2 Hz, 1H), 5.95 – 5.84 (m, 1H), 5.32 (ddd, *J* = 17.2, 2.9, 1.5 Hz, 1H), 5.28 – 5.24 (m, 1H), 4.98 (bs, 1H), 4.63 (dt, *J* = 5.8, 1.3 Hz, 2H), 4.40 (bs, 1H), 4.07 (dd, *J* = 18.3, 5.5 Hz, 1H), 3.96 (dd, *J* = 18.4, 5.0 Hz, 1H), 3.12 (dd, *J* = 13.9, 6.7 Hz, 1H), 3.05 (dd, *J* = 13.8, 7.2 Hz, 1H), 1.40 (s, 9H); ^{13}C NMR (100 MHz, $CDCl_3$): δ = 171.7, 169.1, 155.4, 136.6, 131.4, 129.3, 128.6, 126.8, 118.9, 80.2, 65.9, 55.5, 41.2, 38.4, 28.2; HR-MS: *m/z* = 363.1922 $[M+H]^+$, 363.1920 calcd. for $C_{19}H_{27}N_2O_5$.

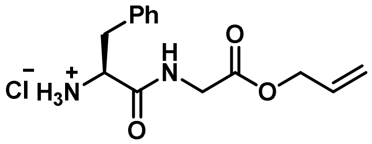

(*S*)-1-((2-(Allyloxy)-2-oxoethyl)amino)-1-oxo-3-phenylpropan-2-aminium chloride

This reaction was performed on a 30 mmol scale. Dipeptide (*S*)-Allyl 2-(2-((tert-butoxycarbonyl)amino)-3-phenylpropanamido)acetate (1.0 equiv.) was added to a flask-dried, one-necked, round-bottomed flask equipped with a magnetic stirring bar, sealed with a rubber septum and placed under an atmosphere of dry nitrogen. 1,4-Dioxane was added (2000 mM) and the solution was stirred until homogeneous. HCl (4.0 M in dioxane) was then added (11 equiv.) and the resulting solution was stirred at room temperature. After one hour, TLC analysis (solvent system: 43% ethyl acetate in hexanes) indicated that the reaction had gone to completion. At this point the septum was removed and nitrogen gas was carefully bubbled through the reaction solution with stirring, using a glass pipette for 3 hours. The dioxane was then removed *in vacuo* and the resulting pale yellow syrup was dissolved in dichloromethane (250 mM) and the solvent was subsequently removed *in vacuo*. This process with dichloromethane was repeated twice more. After the third concentration, the pale yellow syrup was dissolved in acetone: heptane (1:1, *v*:*v*) (250 mM) and the solvent was subsequently removed *in vacuo*. This process was also repeated twice more. The residue was then dried under high vacuum overnight to give a pale-yellow solid. This solid was then triturated with hexanes, and upon filtration and further drying under high vacuum, afforded dipeptide (*S*)-1-((2-(allyloxy)-2-oxoethyl)amino)-1-oxo-3-phenylpropan-2-aminium chloride as an off-white solid in quantitative yield that was used without further purification. The product was found to very hygroscopic. ^{1}H NMR (400 MHz, DMSO-d_6) δ 9.31 (t, *J* = 5.8 Hz, 1H), 8.42 (s, 3H), 7.39 – 7.20 (m, 5H), 5.91 (ddt, *J* = 17.3, 10.5, 5.4 Hz, 1H), 5.33 (dq, *J* = 17.3, 1.7 Hz, 1H), 5.22 (ddt, *J* = 10.5, 1.8, 1.4 Hz, 1H),

4.60 (dt, J = 5.4, 1.5 Hz, 2H), 4.12 (dd, J = 7.1, 5.8 Hz, 1H), 3.99 (dd, J = 17.4, 5.9 Hz, 1H), 3.91 (dd, J = 17.4, 5.7 Hz, 1H), 3.20 (dd, J = 14.0, 5.8 Hz, 1H), 3.06 (dd, J = 14.0, 7.1 Hz, 1H); ^{13}C NMR (101 MHz, DMSO) δ 168.9, 168.6, 134.9, 132.3, 129.7, 128.4, 127.0, 118.0, 64.9, 53.3, 40.6, 36.7; HR-MS: *m/z* = 263.1391 $[M+H]^+$, calcd. 263.1396 for $C_{14}H_{19}N_2O_3$.

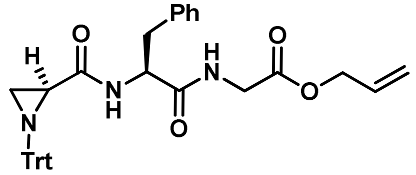

Allyl 2-((*S*)-3-phenyl-2-((*S*)-1-tritylaziridine-2-carboxamido)propanamido)acetate

Prepared *via* a PyBOP-mediated coupling between lithium (*S*)-1-tritylaziridine-2-carboxylate (2.68 g, 8.0 mmol) and dipeptide (*S*)-1-((2-(allyloxy)-2-oxoethyl)amino)-1-oxo-3-phenylpropan-2-aminium chloride (2.39 g, 8.0 mmol) using the same general procedure for the synthesis of (*S*)-allyl 2-(2-((tert-butoxycarbonyl)amino)-3-phenylpropanamido)acetate. The product was purified by silica-gel flash chromatography (25% to 50% ethyl acetate in hexanes) to give 4.09g (7.1 mmol, 89% yield) of tripeptide allyl 2-((*S*)-3-phenyl-2-((*S*)-1-tritylaziridine-2-carboxamido)propanamido)acetate as a white foam. ^{1}H NMR 400 MHz, $CDCl_3$) δ 7.46 – 7.08 (m, 21H), 6.73 (t, J = 5.5 Hz, 1H), 5.91 (ddt, J = 16.5, 10.9, 5.8 Hz, 1H), 5.34 (dd, J = 16.8, 2.0 Hz, 1H), 5.27 (d, J = 10.4 Hz, 1H), 4.73 (q, J = 7.4 Hz, 1H), 4.65 (d, J = 5.7 Hz, 2H), 4.13 – 3.97 (m, 2H), 3.30 – 3.15 (m, 2H), 1.96 (dd, J = 6.6, 2.7 Hz, 1H), 1.47 (d, J = 2.7 Hz, 1H), 1.30 (d, J = 6.6 Hz, 1H); ^{13}C NMR (100 MHz, $CDCl_3$) δ 171.4, 171.2, 169.1, 143.1, 136.5, 131.6, 129.4, 129.4, 128.9, 127.9, 127.3, 127.3, 127.2, 119.0, 74.5, 66.1, 53.4, 41.4, 37.4, 33.8, 29.8; HR-MS: *m/z* = : 596.2506 $[M+Na]^+$, calcd. 596.2504 for $C_{33}H_{34}N_3NaO_4$.

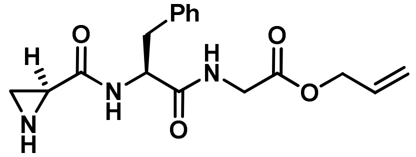

Allyl 2-((*S*)-2-((*S*)-aziridine-2-carboxamido)-3-phenylpropanamido)acetate

Allyl 2-((*S*)-3-phenyl-2-((*S*)-1-tritylaziridine-2-carboxamido)propanamido)acetate (4.10 g, 7.0 mmol) was dissolved in anhydrous dichloromethane (140 mL, 50 mM) under an atmosphere of dry nitrogen. Triethylsilane (4.5 mL, 28 mmol, 4 eq.) was then added and the solution was cooled to -10 °C. Once cooled, trifluoroacetic acid (2.1 mL, 28 mmol, 4 eq.) was added and the solution stirred at this temperature for 30 minutes, at which point TLC analysis (solvent system = 50% ethyl acetate in hexanes) indicated complete consumption of the starting peptide. *N*,*N*-diisopropylethylamine (6.1 mL, 35 mmol, 5 eq.) was then added dropwise at this temperature and after the addition, the solution was allowed to warm to room temperature over 20 minutes. The reaction was then concentrated *in vacuo* and the resulting residue was purified by silica-gel flash chromatography (2.5% to 5% methanol in chloroform) to afford the deprotected tripeptide allyl 2-((*S*)-2-((*S*)-aziridine-2-carboxamido)-3-phenylpropanamido)acetate in 91% yield (2.10 g, 6.3 mmol) as a white solid. By ^{1}H NMR, the compound appears as two conformers $CDCl_3$ at 25 °C. ^{1}H NMR (400 MHz, $CDCl_3$) δ 7.26 (s, 12H), 6.90 (d, J = 8.2 Hz, 1H), 6.68 (s, 1H), 6.58

(s, 1H), 6.16 (s, 1H), 5.99 – 5.81 (m, 2H), 5.33 (d, *J* = 16.9 Hz, 2H), 5.27 (d, *J* = 10.4 Hz, 2H), 4.73 (q, *J* = 7.2 Hz, 1H), 4.67 – 4.56 (m, 4H), 4.12 – 3.88 (m, 4H), 3.25 – 3.04 (m, 3H), 2.93 (dd, *J* = 14.2, 9.0 Hz, 1H), 2.66 – 2.58 (m, 1H), 2.29 (dt, *J* = 7.8, 3.4 Hz, 1H), 2.03 – 1.91 (m, 2H), 1.78 – 1.70 (m, 1H), 1.61 (s, 1H), 1.15 (d, *J* = 3.1 Hz, 1H), 1.13 (d, *J* = 4.1 Hz, 1H), 0.78 (q, *J* = 8.8 Hz, 1H); ^{13}C NMR (100 MHz, $CDCl_3$) δ 171.2, 169.2, 136.4, 131.5, 129.3, 128.7, 127.1, 119.1, 66.1, 54.8, 53.4, 41.4, 38.6, 37.5, 30.1, 26.6; HR-MS: *m/z* = : 332.1599 $[M+H]^+$, calcd. 332.1605 for $C_{17}H_{22}N_3O_4$.

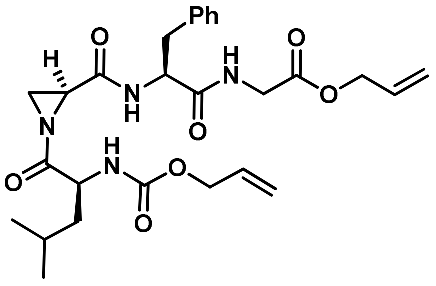

Allyl-2-((*S*)-2-((*S*)-1-((*S*)-2-(((allyloxy)carbonyl)amino)-4-methylpentanoyl)aziridine-2-carboxamido)-3-phenylpropanamido)acetate

N-Alloc-leucine (1.48 g, 6.9 mmol) was dissolved in anhydrous chloroform (35 mL), capped with a rubber septum and placed under a dry nitrogen atmosphere with stirring. The solution was then cooled to 0 °C in an ice bath and TOTU (2.7 g, 8.3 mmol) was added by quickly removing the septum and adding the solid in one portion. The reaction vessel was then resealed with a septum, and placed back under a dry nitrogen atmosphere. *N,N*-Diisopropylethylamine (1.2 mL, 6.9 mmol) was then added drop-wise and the resulting solution was stirred at 0 °C for 20 minutes. Tripeptide allyl 2-((*S*)-2-((*S*)-aziridine-2-carboxamido)-3-phenylpropanamido)acetate (2.05 g, 6.2 mmol) was dissolved separately in anhydrous chloroform (35 mL) and this solution was then added to the reaction mixture drop-wise followed by the addition of additional *N,N*-diisopropylethylamine (1.2 mL, 6.9 mmol) drop-wise. The reaction was stirred at 0 °C for 30 minutes and then at room temperature for 4 hours. The reaction mixture was subsequently diluted with ethyl acetate (350 mL). The solution was then washed with 10% aqueous citric acid (150 mL), water (150 mL) and saturated aqueous sodium chloride (150 mL). The organic layer was then dried over Na_2SO_4, filtered and concentrated *in vacuo*. The resulting residue was then purified by silica-gel flash chromatography (40% to 70% ethyl acetate in hexanes) to give the protected tetrapeptide allyl-2-((*S*)-2-((*S*)-1-((*S*)-2-(((allyloxy)carbonyl)amino)-4-methylpentanoyl)aziridine-2-carboxamido)-3-phenylpropanamido)acetate (2.85 g, 5.4 mmol) in 87% yield as an orange foam. ^{1}H NMR (500 MHz, $CDCl_3$) δ 7.27 – 7.09 (m, 5H), 7.04 – 6.94 (m, 2H), 5.91 – 5.75 (m, 3H), 5.31 – 5.25 (m, 2H), 5.25 – 5.22 (m, 2H), 5.22 – 5.20 (m, 2H), 5.18 – 5.11 (m, 2H), 4.79 (td, *J* = 8.3, 6.2 Hz, 1H), 4.58 (dt, *J* = 5.8, 1.4 Hz, 2H), 4.53 – 4.45 (m, 1H), 4.45 – 4.37 (m, 1H), 4.23 (ddd, *J* = 9.8, 7.9, 4.4 Hz, 1H), 4.09 (dd, *J* = 18.0, 6.0 Hz, 1H), 3.87 (dd, *J* = 18.1, 5.1 Hz, 1H), 3.17 (dd, *J* = 14.0, 6.2 Hz, 1H), 3.06 (dd, *J* = 6.3, 3.2 Hz, 1H), 2.95 (dd, *J* = 14.0, 8.1 Hz, 1H), 2.56 (d, *J* = 6.5 Hz, 1H), 1.95 (d, *J* = 2.6 Hz, 1H), 1.76 – 1.63 (m, 2H), 1.63 – 1.54 (m, 1H), 0.92 (dd, *J* = 9.1, 6.1 Hz, 6H). ^{13}C NMR (126 MHz, $CDCl_3$) δ 184.9, 171.1, 169.7, 167.5, 156.0, 136.4, 132.4, 131.4, 129.2, 128.5, 128.5, 127.0, 118.9, 118.3, 66.0, 66.0, 54.8, 53.3, 41.4, 41.2, 37.7, 36.1, 30.5, 24.8, 23.0, 21.5; HR-MS: *m/z* = 529.2635 $[M+H]^+$, calcd. 529.2657 for $C_{27}H_{37}N_4O_7$.

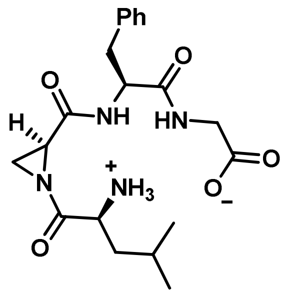

2-((*S*)-2-((*S*)-1-((*S*)-2-Ammonio-4-methylpentanoyl)aziridine-2-carboxamido)-3-phenylpropanamido)acetate

Protected tetrapeptide allyl-2-((*S*)-2-((*S*)-1-((*S*)-2-(((allyloxy)carbonyl)amino)-4-methylpentanoyl)aziridine-2-carboxamido)-3-phenylpropanamido)acetate (1.06 g, 2.0 mmol) and $Pd(PPh_3)_4$ (0.116 g, 0.1 mmol) were dissolved in anhydrous dichloromethane (40 mL. 50 mM) and placed under an atmosphere of dry nitrogen. *N,N*-dimethylbarbituric acid (0.62 g, 4.0 mmol) was dissolved separately in anhydrous dichloromethane (8 mL) and added drop-wise to the reaction system. After stirring for 1 hour, the deprotected tetrapeptide had precipitated out and the solution was filtered. The precipitate was washed repeatedly with dichloromethane and also acetonitrile and was dried *in vacuo* to yield the desired tetrapeptide 2-((*S*)-2-((*S*)-1-((*S*)-2-Ammonio-4-methylpentanoyl)aziridine-2-carboxamido)-3-phenylpropanamido)acetate (0.76 g, 1.9 mmol) in 95% yield as a white solid. The product was used with further purification. ^{1}H NMR (500 MHz, DMSO-d_6) δ 8.95 (d, J = 8.5 Hz, 1H), 8.24 (t, J = 5.4 Hz, 1H), 7.31 – 7.16 (m, 5H), 4.63 (td, J = 9.1, 4.3 Hz, 1H), 3.66 (d, J = 5.4 Hz, 2H), 3.27 (dd, J = 5.1, 3.1 Hz, 1H), 3.17 – 3.03 (m, 2H), 2.82 (dd, J = 14.0, 9.9 Hz, 1H), 2.46 (d, J = 3.5 Hz, 1H), 2.19 (s, 1H), 1.70 (dq, J = 13.6, 6.8 Hz, 1H), 1.49 – 1.39 (m, 1H), 1.39 – 1.29 (m, 1H), 0.84 (d, J = 6.6 Hz, 3H), 0.79 (d, J = 6.6 Hz, 3H); ^{13}C NMR (126 MHz, DMSO) δ 184.4, 171.3, 170.5, 166.7, 137.6, 129.1, 128.1, 126.3, 54.0, 53.6, 42.6, 41.7, 37.7, 35.9, 29.0, 24.0, 22.7, 22.0; HR-MS: *m/z* = 405.2122 $[M+H]^+$, calcd. 405.2132 for $C_{20}H_{29}N_4O_5$.

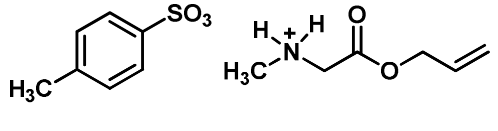

2-(Allyloxy)-*N*-methyl-2-oxoethanaminium 4-methylbenzenesulfonate

Prepared *via* the same procedure to yield tosylate salt 2-(allyloxy)-2-oxoethanaminium 4-methylbenzenesulfonate, starting with sarcosine (8.91 g, 100 mmol). After precipitation from ice-cold diethyl ether, vacuum filtration, and drying *in vacuo*, the product 2-(allyloxy)-*N*-methyl-2-oxoethanaminium 4-methylbenzenesulfonate was isolated (29.3 g, 97.0 mmol) in 97% yield as a white solid. ^{1}H NMR (399 MHz, DMSO-d_6) δ 8.90 (s, 3H), 7.48 (d, J = 8.1 Hz, 2H), 7.23 – 6.95 (m, 2H), 5.94 (ddt, J = 17.3, 10.7, 5.5 Hz, 1H), 5.38 (dq, J = 17.2, 1.6 Hz, 1H), 5.28 (dq, J = 10.5, 1.4 Hz, 1H), 4.71 (dt,

J = 5.5, 1.5 Hz, 3H), 4.05 (s, 2H), 2.60 (s, 3H), 2.29 (s, 3H); ^{13}C NMR (100 MHz, DMSO) δ 166.5, 145.6, 137.7, 131.7, 128.1, 125.5, 118.7, 65.8, 48.1, 32.7, 20.8; HR-MS: m/z = 130.0861 [M+H]$^+$, calcd. 130.0863 for $C_6H_{12}N_2O$.

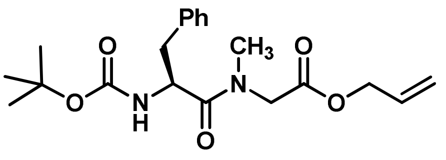

(*S*)-Allyl 2-(2-((tert-butoxycarbonyl)amino)-*N*-methyl-3-phenylpropanamido)acetate

Prepared *via* a PyBOP-mediated coupling between Boc-Phe-OH (10.61 g, 40.0 mmol) and 2-(allyloxy)-*N*-methyl-2-oxoethanaminium 4-methylbenzenesulfonate (12.05 g, 40.0 mmol) using the same general procedure for the synthesis of (*S*)-allyl 2-(2-((tert-butoxycarbonyl)amino)-3-phenylpropanamido)acetate. The product was purified by silica-gel chromatography (20% to 30% ethyl acetate in hexanes) to give 14.75 g (39.2 mmol, 97% yield) of (*S*)-allyl 2-(2-((tert-butoxycarbonyl)amino)-*N*-methyl-3-phenylpropanamido)acetate as a colourless syrup. R_f= 0.43 (30% ethyl acetate in hexanes). The product exists in two distinct conformations on the NMR time scale in $CDCl_3$ at 25 °C in a ratio of 1.00 : 0.36. The major conformer is reported: ^{1}H NMR (400 MHz, $CDCl_3$) δ 7.32 – 7.19 (m, 5H), 5.90 (ddt, J = 17.3, 10.5, 5.9 Hz, 1H), 5.37 – 5.28 (m, 2H), 5.26 (dq, J = 10.4, 1.3 Hz, 1H), 4.89 (dt, J = 8.9, 6.9 Hz, 1H), 4.63 (dt, J = 5.8, 1.4 Hz, 2H), 4.15 (d, J = 17.3 Hz, 1H), 4.02 (d, J = 17.3 Hz, 1H), 3.04 (dd, J = 13.6, 7.2 Hz, 1H), 2.97 – 2.93 (m, 1H), 2.89 (s, 3H), 1.40 (s, 9H); ^{13}C NMR (101 MHz, $CDCl_3$) δ 172.4, 168.5, 155.1, 136.4, 131.7, 129.7, 128.5, 126.9, 118.9, 65.9, 51.5, 49.7, 39.7, 36.3, 28.4, 28.4; HR-MS: m/z = 377.2068 [M+H]$^+$, calcd. 377.2071 for $C_{20}H_{29}N_2O_5$.

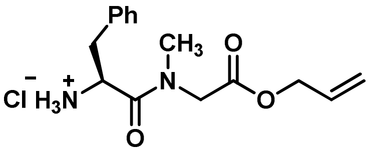

(*S*)-1-((2-(Allyloxy)-2-oxoethyl)(methyl)amino)-1-oxo-3-phenylpropan-2-aminium-chloride

Prepared *via* the same procedure to yield dipeptide (*S*)-1-((2-(allyloxy)-2-oxoethyl)amino)-1-oxo-3-phenylpropan-2-aminium chloride, using Boc-protected dipeptide (*S*)-allyl 2-(2-((tert-butoxycarbonyl)amino)-*N*-methyl-3-phenylpropanamido)acetat (14.68 g, 39.0 mmol). After a final trituration with hexanes, the product (*S*)-1-((2-(allyloxy)-2-oxoethyl)(methyl)amino)-1-oxo-3-phenylpropan-2-aminium-chloride was isolated in 96% yield (11.73 g, 37.5 mmol) as an off-white sticky foam that was extremely hygroscopic. The product exists in two distinct conformations on the NMR time scale in DMSO-d_6 at 25 °C in a ratio of 1 : 0.35. The major conformer is reported: ^{1}H NMR (400 MHz, DMSO-d_6) δ 8.53 (s, 3H), 7.37 – 7.20 (m, 5H), 5.91 (ddt, J = 17.2, 10.7, 5.4 Hz, 1H), 5.36 – 5.29 (m, 1H), 5.25 – 5.20 (m, 1H), 4.63 – 4.58 (m, 3H), 4.19 (d, J = 17.1 Hz, 1H), 4.05 (d, J = 17.1 Hz, 1H), 3.16 (dd, J = 13.8, 5.6 Hz, 1H), 3.09 – 3.01 (m, 1H), 2.82 (s, 3H); ^{13}C NMR (101 MHz, DMSO) δ 168.8, 168.3,

134.5, 132.3, 129.8, 128.4, 127.2, 118.0, 65.0, 50.4, 49.3, 36.1, 35.9; HR-MS: *m/z* = 277.1539 $[M+H]^+$, calcd. 277.1547 for $C_{15}H_{21}N_2O_5$.

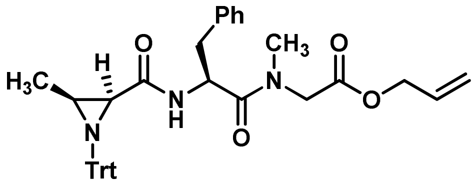

Allyl 2-((*S*)-*N*-methyl-2-((2*S*,3*S*)-3-methyl-1-tritylaziridine-2-carboxamido)-3-phenylprop-anamido)acetate

Prepared *via* a PyBOP-mediated coupling between aziridine acid (2*S*,3*S*)-3-methyl-1-tritylaziridine-2-carboxylic acid[2] (10.30 g, 30.0 mmol) and dipeptide (*S*)-1-((2-(allyloxy)-2-oxoethyl)(methyl)amino)-1-oxo-3-phenylpropan-2-aminium-chloride (9.38 g, 30.0 mmol) using the same general procedure for the synthesis of allyl 2-((*S*)-3-phenyl-2-((*S*)-1-tritylaziridine-2-carboxamido)propanamido)acetate. The product was purified by silica-gel flash chromatography (20% to 50% ethyl acetate in hexanes) to give 14.42 g (24.0 mmol, 82% yield) of tripeptide allyl 2-((*S*)-*N*-methyl-2-((2*S*,3*S*)-3-methyl-1-tritylaziridine-2-carboxamido)-3-phenylprop-anamido)acetate as a white foam. The product exists in two distinct conformations on the NMR time scale in $CDCl_3$ at 25 °C in a ratio of 1.00 : 0.30. The major conformer is reported: R_f= 0.34 (40% ethyl acetate in hexanes). ^1H NMR (400 MHz, $CDCl_3$) δ 7.38 (d, *J* = 8.7 Hz, 1H), 7.37 – 7.08 (m, 20H), 5.84 (ddt, *J* = 16.4, 10.4, 5.8 Hz, 1H), 5.30 – 5.22 (m, 2H), 5.18 (dd, *J* = 10.3, 1.3 Hz, 1H), 4.57 (ddd, *J* = 5.8, 3.0, 1.4 Hz, 2H), 4.19 (d, *J* = 17.2 Hz, 1H), 3.96 (d, *J* = 17.2 Hz, 1H), 3.11 (dd, *J* = 13.7, 7.9 Hz, 1H), 3.00 (dd, *J* = 13.7, 6.6 Hz, 1H), 2.82 (s, 3H), 1.86 (d, *J* = 6.9 Hz, 1H), 1.48 – 1.40 (m, 1H), 1.06 (d, *J* = 5.6 Hz, 3H); ^{13}C NMR (100 MHz, $CDCl_3$) δ 171.9, 168.7, 168.3, 143.5, 136.0, 131.6, 129.5, 129.4, 128.4, 127.6, 127.0, 126.8, 118.8, 75.3, 65.7, 49.7, 49.7, 39.3, 38.4, 36.2, 35.3, 34.5, 13.0; HR-MS: *m/z* = 624.2814 $[M+Na]^+$, calcd. 624.2833 for $C_{38}H_{39}N_3NaO_4$.

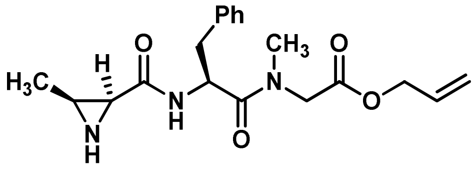

Allyl 2-((*S*)-*N*-methyl-2-((2*S*,3*S*)-3-methylaziridine-2-carboxamido)-3-phenylpropanamido)-acetate

Prepared *via* the same trityl deprotection procedure to give allyl 2-((*S*)-2-((*S*)-aziridine-2-carboxamido)-3-phenylpropanamido)acetate, using trityl-protected tripepitde allyl 2-((*S*)-*N*-methyl-2-((2*S*,3*S*)-3-methyl-1-tritylaziridine-2-carboxamido)-3-phenylprop-anamido)acetate (14.42 g, 24.0 mmol). After purification by silica-gel flash column chromatography (2.5% to 5% methanol in chloroform) the product allyl 2-((*S*)-*N*-methyl-2-((2*S*,3*S*)-3-methylaziridine-2-carboxamido)-3-phenylpropanamido)-acetate was isolated in 94% yield (8.12 g, 22.6 mmol) as a pale yellow syrup. R_f= 0.46 (10% methanol in chloroform). The product exists in two distinct conformations on the NMR time scale in $CDCl_3$ at 25 °C in a ratio of 1.00 : 0.40. The major conformer is reported: ^1H NMR (400 MHz, $CDCl_3$) δ 7.33 – 7.15 (m, 6H), 5.90 (ddt, *J* = 17.0, 10.3, 5.7 Hz, 1H), 5.33 (dd, *J* = 17.2, 1.5 Hz, 1H), 5.29 – 5.22 (m, 2H), 4.63 (d, *J* = 6.1 Hz, 2H), 4.18 (d, *J*

[2] White, C. J.; Yudin, A. K. *Org. Lett.* **2012**, *14* (11), 2898.

= 17.3 Hz, 1H), 4.00 (d, J = 17.3 Hz, 1H), 3.13 – 3.05 (m, 1H), 2.92 (s, 3H), 2.71 – 2.65 (m, 1H), 2.38 – 2.30 (m, 1H), 1.26 (s, 1H), 1.05 – 0.98 (m, 1H), 0.90 (d, J = 5.7 Hz, 3H); ^{13}C NMR (100 MHz, $CDCl_3$) δ 172.1, 168.9, 168.5, 136.3, 131.7, 129.4, 128.6, 127.1, 118.9, 66.3, 65.9, 49.8, 39.2, 36.5, 35.4, 32.0, 13.6; HR-MS: *m/z* = 360.1927 $[M+H]^+$, calcd. 360.1918 for $C_{19}H_{26}N_3O_4$.

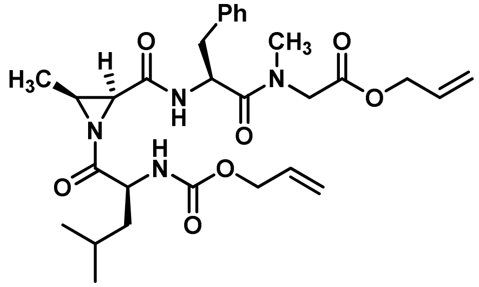

Allyl 2-((*S*)-2-((2*S*,3*S*)-1-((*S*)-2-(((allyloxy)carbonyl)amino)-4-methylpentanoyl)-3-methyl-aziridine-2-carboxamido)-*N*-methyl-3-phenylpropanamido)acetate

Prepared *via* a TOTU-mediated coupling between Allo-Leu-OH (5.31 g, 24.7 mmol) and tripeptide allyl 2-((*S*)-*N*-methyl-2-((2*S*,3*S*)-3-methylaziridine-2-carboxamido)-3-phenylpropanamido)-acetate (8.22 g, 22.9 mmol) using the same procedure for the synthesis of allyl-2-((*S*)-2-((*S*)-1-((*S*)-2-(((allyloxy)carbonyl)amino)-4-methylpentanoyl)aziridine-2-carboxamido)-3-phenylpropanamido)acetate. The product was purified by silica-gel flash chromatography (50% to 70% ethyl acetate in hexanes) to give 11.1 g (19.9 mmol, 87% yield) of tetrapeptide allyl 2-((*S*)-2-((2*S*,3*S*)-1-((*S*)-2-(((allyloxy)carbonyl)amino)-4-methylpentanoyl)-3-methyl-aziridine-2-carboxamido)-*N*-methyl-3-phenylpropanamido)acetate as a red syrup. The product exists in two distinct conformations on the NMR time scale in $CDCl_3$ at 25 °C in a ratio of 1.00 : 0.45. The major conformer is reported: ^{1}H NMR (399 MHz, $CDCl_3$) δ 7.28 – 7.20 (m, 5H), 6.93 (d, J = 8.7 Hz, 1H), 5.97 – 5.80 (m, 2H), 5.38 – 5.31 (m, 1H), 5.30 – 5.24 (m, 3H), 5.23 – 5.18 (m, 1H), 4.65 (dt, J = 5.8, 1.4 Hz, 2H), 4.54 – 4.46 (m, 2H), 4.30 (d, J = 17.3 Hz, 1H), 4.27 – 4.21 (m, 1H), 3.91 (d, J = 17.2 Hz, 1H), 3.13 (dd, J = 6.9, 1.9 Hz, 1H), 2.98 (d, J = 6.9 Hz, 1H), 2.95 (s, 3H), 2.91 – 2.88 (m, 1H), 1.81 – 1.72 (m, 2H), 1.69 – 1.59 (m, 1H), 1.10 (d, J = 5.7 Hz, 3H), 0.99 (d, J = 6.4 Hz, 3H), 0.97 (d, J = 6.1 Hz, 3H).^{13}C NMR (100 MHz, $CDCl_3$) δ 185.7, 172.0, 168.4, 166.0, 156.2, 135.9, 132.8, 131.6, 129.5, 128.7, 127.2, 119.1, 118.0, 66.0, 55.1, 49.9, 42.1, 39.0, 37.9, 36.5, 31.7, 25.0, 23.1, 22.8, 21.7, 14.2, 13.0; HR-MS: *m/z* = 557.2953 $[M+H]^+$, calcd. 557.2970 for $C_{29}H_{41}N_4O_7$.

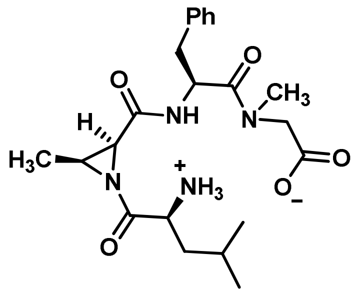

2-((*S*)-2-((2*S*,3*S*)-1-((*S*)-2-ammonio-4-methylpentanoyl)-3-methylaziridine-2-carboxamido)-*N*-methyl-3-phenylpropanamido)acetate

Prepared *via* the same procedure to yield tetrapeptide 2-((*S*)-2-((*S*)-1-((*S*)-2-Ammonio-4-methylpentanoyl)aziridine-2-carboxamido)-3-phenylpropanamido)acetate, using protected tetrapeptide allyl 2-((*S*)-2-((2*S*,3*S*)-1-((*S*)-2-(((allyloxy)carbonyl)amino)-4-methylpentanoyl)-3-methyl-aziridine-2-carboxamido)-*N*-methyl-3-phenylpropanamido)acetate (1.11 g, 2.0 mmol). After precipitation with *tert*-butyl methyl ether, vacuum filtration, extensive washing with dichloromethane and acetonitrile, and drying *in vacuo*, the product 2-((*S*)-2-((2*S*,3*S*)-1-((*S*)-2-ammonio-4-methylpentanoyl)-3-methylaziridine-2-carboxamido)-*N*-methyl-3-phenylpropanamido)acetate was isolated in 79% yield (0.685 g, 1.58 mmol) as a pale-orange solid. The crude product was shown to be approximately 95% pure by analytical HPLC (Elution Method A, Retention time = 4.121 min.). The product exists as two distinct conformations on the NMR time scale in DMSO-d_6 at 25 °C in a ratio of approximately 1.00 : 1.00. Both conformers are reported: ^{1}H NMR (400 MHz, DMSO-d_6) δ 8.34 (d, *J* = 8.4 Hz, 1H), 8.28 (d, *J* = 8.2 Hz, 1H), 7.32 – 7.14 (m, 10H), 4.97 (td, *J* = 8.8, 5.0 Hz, 1H), 4.76 – 4.68 (m, 1H), 4.23 (d, *J* = 17.9 Hz, 1H), 3.95 (d, *J* = 16.8 Hz, 1H), 3.83 (d, *J* = 17.0 Hz, 1H), 3.72 (d, *J* = 17.9 Hz, 1H), 3.63 – 3.52 (m, 2H), 3.11 (d, *J* = 6.5 Hz, 1H), 2.52 – 2.48 (m, 1H), 3.07 (d, *J* = 6.5 Hz, 1H), 3.03 (s, 3H), 2.99 – 2.96 (m, 1H), 2.95 – 2.89 (m, 1H), 2.89 – 2.84 (m, 1H), 2.83 (s, 3H), 1.89 – 1.78 (m, 2H), 1.63 – 1.52 (m, 2H), 1.50 – 1.41 (m, 2H), 0.97 – 0.82 (m, 18H); ^{13}C NMR (101 MHz, DMSO) δ 184.5, 184.3, 171.3, 171.1, 170.8, 170.7, 165.5, 165.4, 138.0, 137.4, 129.3, 129.3, 128.1, 128.0, 126.4, 126.2, 53.0, 52.9, 50.4, 50.1, 50.0, 42.6, 42.5, 39.5, 37.0, 36.8, 35.9, 34.9, 26.8, 23.9, 22.8, 22.7, 21.9, 21.9, 12.2, 12.1; HR-MS: *m/z* = 433.2433 $[M+H]^+$, calcd. 433.2445 for $C_{22}H_{33}N_4O_5$.

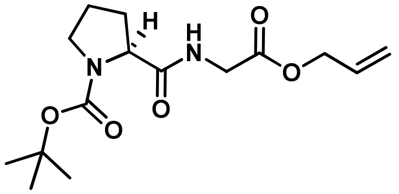

(*S*)-*Tert*-butyl 2-((2-(allyloxy)-2-oxoethyl)carbamoyl)pyrrolidine-1-carboxylate

Prepared *via* a PyBOP-mediated coupling between Boc-Pro-OH (6.46 g, 30.0 mmol) and tosylate 2-(allyloxy)-2-oxoethanaminium 4-methylbenzenesulfonate (8.62 g, 30.0 mmol) using the same general procedure for the synthesis of (*S*)-allyl 2-(2-((tert-butoxycarbonyl)amino)-3-phenylpropanamido)acetate. The product was purified by silica-gel chromatography (40% to 50% ethyl acetate in hexanes) to give 8.87 g (28.5 mmol, 95% yield) of (*S*)-*tert*-butyl 2-((2-(allyloxy)-2-oxoethyl)carbamoyl)pyrrolidine-1-carboxylate as a colourless syrup that slowly crystallized into white needles. R_f = 0.35 (50% ethyl acetate in hexanes). ^{1}H NMR (399 MHz, $CDCl_3$) δ 6.00 – 5.83 (m, 1H), 5.38 – 5.23 (m, 2H), 4.64 (d, *J* = 4.5 Hz, 2H), 4.42 – 4.22 (m, 1H), 4.12 (dd, *J* = 18.3, 5.7 Hz, 1H), 4.02 (d, *J* = 17.8 Hz, 1H), 3.41 (d, *J* = 42.1 Hz, 2H), 2.34 (s, 1H), 2.16 (s, 1H), 1.99 – 1.82 (m, 3H), 1.47 (s, 9H);

^{13}C NMR (100 MHz, $CDCl_3$) δ 172.5, 169.4, 155.8, 131.6, 118.9, 80.6, 65.9, 60.0, 47.2, 41.3, 31.0, 28.4, 24.5.

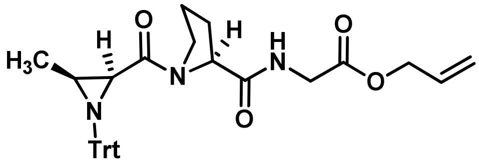

Allyl 2-((*S*)-1-((2*S*,3*S*)-3-methyl-1-tritylaziridine-2-carbonyl)pyrrolidine-2-carboxamido)-acetate

Dipeptide (*S*)-*tert*-butyl 2-((2-(allyloxy)-2-oxoethyl)carbamoyl)pyrrolidine-1-carboxylate (9.40 g, 30.0 mmol) was added to a flask-dried round-bottomed flask equipped with a magnetic stirring bar, sealed with a rubber septum and placed under an atmosphere of dry nitrogen. 1,4-Dioaxane was added (30 mL, 1.0 M) and the solution was stirred until homogeneous. HCl (4.0 M in dioxane) was then added (80 mL, 320 mmol) and the resulting solution was stirred at room temperature. After one hour, TLC analysis (solvent system: 50% ethyl acetate in hexanes) indicated that the reaction had gone to completion. At this point the solvent was then removed *in vacuo* and the resulting pale yellow syrup was dissolved in dichloromethane (50 mL) and the solvent was subsequently removed *in vacuo*. This process with dichloromethane was repeated twice more. The residue was then dried under high vacuum overnight to give deprotected dipeptide as a yellow syrup that was used directly in the next step. Into a separate flame-dried, round-bottomed flask equipped with a magnetic stirring bar, was added aziridine acid (2*S*,3*S*)-3-methyl-1-tritylaziridine-2-carboxylic acid[2] (10.03 g, 30.0 mmol) and PyBOP (18.73 g, 36.0 mmol). The flask was sealed with a rubber septum and placed under an atmosphere of dry nitrogen. Chloroform was then added (20 mL, 1.5 M) and the flask was cooled to 0 °C in a ice bath with stirring. *N,N*-diisopropylethylamine (15.7 mL, 90.0 mmol) was then added drop-wise, followed by the addition of the deprotected dipeptide in DMF (40 mL). The reaction flask was then warmed to room temperature and stirred for 11 hours. The reaction was then subjected to standard work-up conditions (see synthesis of allyl 2-((*S*)-*N*-methyl-2-((2*S*,3*S*)-3-methyl-1-tritylaziridine-2-carboxamido)-3-phenylprop-anamido)acetate). The product was purified by silica-gel chromatography (30% to 60% ethyl acetate in hexanes) to give 14.52 g of tripeptide allyl 2-((*S*)-1-((2*S*,3*S*)-3-methyl-1-tritylaziridine-2-carbonyl)pyrrolidine-2-carboxamido)-acetate (27.0 mmol, 90% over two steps) as a white foam. R_f= 0.44 (60% ethyl acetate in hexanes). The product exists in two distinct conformations on the NMR time scale in $CDCl_3$ at 25 °C in a ratio of 1.00 : 0.34. The major conformer is reported: ^1H NMR (399 MHz, Chloroform-*d*) δ 7.76 (t, *J* = 5.7 Hz, 1H), 7.65 – 7.14 (m, 15H), 5.89 (ddt, *J* = 17.1, 10.3, 5.7 Hz, 1H), 5.31 (dd, *J* = 17.2, 1.5 Hz, 1H), 5.23 (dd, *J* = 10.4, 1.3 Hz, 1H), 4.71 (d, *J* = 7.3 Hz, 1H), 4.64 – 4.58 (m, 2H), 4.01 (dd, *J* = 7.2, 5.7 Hz, 2H), 3.25 (ddd, *J* = 10.3, 8.5, 2.3 Hz, 1H), 3.10 (td, *J* = 9.9, 7.0 Hz, 1H), 2.42 (dd, *J* = 12.4, 6.3 Hz, 1H), 2.02 – 1.92 (m, 1H), 1.90 (d, *J* = 6.6 Hz, 1H), 1.88 – 1.73 (m, 2H), 1.73 – 1.65 (m, 1H), 1.34 (d, *J* = 5.4 Hz, 3H); ^{13}C NMR (100 MHz, $CDCl_3$) δ 171.5, 168.9, 168.7, 143.8, 131.5, 129.2, 127.3, 126.6, 118.3, 74.7, 65.4, 59.6, 46.3, 41.0, 36.6, 33.3, 26.8, 24.5, 13.2; HR-MS: *m/z* = 538.2683 $[M+H]_+$, calcd. 538.2700 for $C_{33}H_{36}N_3O_4$.

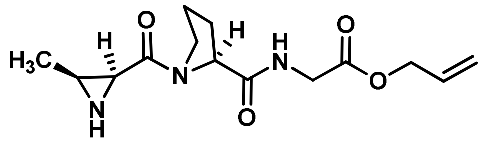

Allyl 2-((2*S*)-1-((2*S*,3*S*)-3-methylaziridine-2-carbonyl)pyrrolidine-2-carboxamido)acetate

Prepared *via* the same trityl deprotection procedure to give allyl-2-((*S*)-2-((2*S*,3*S*)-3-methylaziridine-2-carboxamido)-3-phenylpropanamido)acetate, using trityl-protected tripeptide allyl 2-((*S*)-1-((2*S*,3*S*)-3-methyl-1-tritylaziridine-2-carbonyl)pyrrolidine-2-carboxamido)-acetate (1.63 g, 3.0 mmol). After purification by silica-gel flash column chromatography (5% methanol in chloroform) the product allyl 2-((2*S*)-1-((2*S*,3*S*)-3-methylaziridine-2-carbonyl)pyrrolidine-2-carboxamido)acetate was isolated in 88% yield (0.78 g, 2.65 mmol) as a pale-yellow syrup. R_f= 0.45 (10% methanol in chloroform). ^{1}H NMR (399 MHz, $CDCl_3$) δ 7.49 (s, 1H), 5.94 – 5.78 (m, 1H), 5.30 (ddd, *J* = 17.2, 2.4, 1.2 Hz, 1H), 5.26 – 5.21 (m, 1H), 4.68 – 4.55 (m, 3H), 4.04 – 3.92 (m, 2H), 3.69 – 3.58 (m, 2H), 2.65 (d, *J* = 5.9 Hz, 1H), 2.45 (dtd, *J* = 10.4, 4.1, 1.9 Hz, 1H), 2.35 – 2.26 (m, 1H), 2.19 – 2.00 (m, 2H), 1.94 – 1.82 (m, 1H), 1.23 (d, *J* = 4.9 Hz, 3H), 1.13 (s, 1H); ^{13}C NMR (100 MHz, $CDCl_3$) δ 171.4, 169.5, 169.3, 131.6, 119.0, 66.0, 60.3, 47.0, 41.5, 35.9, 33.9, 27.3, 25.0, 13.2; HR-MS: *m/z* = 296.1601 $[M+H]^+$, calcd. 296.1605 for $C_{14}H_{22}N_3O_4$.

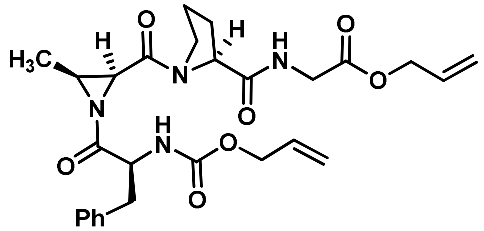

Allyl 2-((*S*)-1-((2*S*,3*S*)-1-((*S*)-2-(((allyloxy)carbonyl)amino)-3-phenylpropanoyl)-3-methyl-aziridine-2-carbonyl)pyrrolidine-2-carboxamido)acetate

Prepared *via* a TOTU-mediated coupling between Allo-Phe-OH (0.50 g, 2.0 mmol) and tripeptide allyl 2-((2*S*)-1-((2*S*,3*S*)-3-methylaziridine-2-carbonyl)pyrrolidine-2-carboxamido)acetate (0.59 g, 2.0 mmol) using the same procedure for the synthesis of allyl-2-((*S*)-2-((2*S*,3*S*)-1-((*S*)-2-(((allyloxy)carbonyl)amino)-4-methylpentanoyl)-3-methyl-aziridine-2-carboxamido)-3-phenylpropanamido)acetate, except the coupling reagent COMU (0.94 g, 2.2 mmol) was used in place of TOTU. The product was purified by silica-gel flash chromatography (60% to 100% ethyl acetate in hexanes) to give 0.70 g (1.3 mmol, 67% yield) of tetrapeptide allyl 2-((*S*)-1-((2*S*,3*S*)-1-((*S*)-2-(((allyloxy)carbonyl)amino)-3-phenylpropanoyl)-3-methyl-aziridine-2-carbonyl)pyrrolidine-2-carboxamido)acetate as yellow foam. ^{1}H NMR (400 MHz, $CDCl_3$) δ 7.43 (t, *J* = 5.7 Hz, 1H), 7.32 – 7.13 (m, 5H), 5.93 – 5.76 (m, 2H), 5.38 (d, *J* = 7.8 Hz, 1H), 5.33 – 5.12 (m, 5H), 4.65 – 4.49 (m, 5H), 4.49 – 4.43 (m, 2H), 4.28 – 4.18 (m, 1H), 3.99 – 3.94 (m, 2H), 3.61 – 3.49 (m, 2H), 3.26 – 3.22 (m, 1H), 3.11 – 2.96 (m, 2H), 2.43 – 2.35 (m, 1H), 2.16 – 2.06 (m, 1H), 2.02 – 1.94 (m, 1H), 1.93 – 1.78 (m, 2H), 1.34 (d, *J* = 5.5 Hz, 3H); ^{13}C NMR (101 MHz, $CDCl_3$) δ 183.6, 171.2, 169.3, 165.8, 155.9, 136.2, 132.5, 131.6, 129.4, 128.7, 127.2, 118.8, 117.8, 65.9, 65.8, 60.1, 57.0, 47.1, 41.4, 40.3, 38.8, 38.6, 27.3, 25.1, 13.5. HR-MS: *m/z* = 527.2484 $[M+H]^+$, calcd. 527.2500 for $C_{27}H_{35}N_4O_7$.

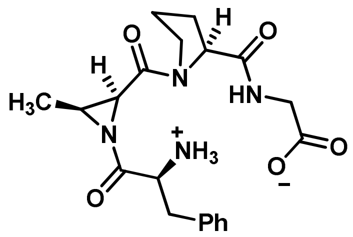

2-((*S*)-1-((2*S*,3*S*)-1-((*S*)-2-ammonio-3-phenylpropanoyl)-3-methylaziridine-2-carbonyl)-pyrrolidine-2-carboxamido)acetate

Prepared *via* the same procedure to yield tetrapeptide 2-((*S*)-2-((2*S*,3*S*)-1-((*S*)-2-ammonio-4-methylpentanoyl)-3-methylaziridine-2-carboxamido)-3-phenylpropanamido)acetate, using protected tetrapeptide allyl 2-((*S*)-1-((2*S*,3*S*)-1-((*S*)-2-(((allyloxy)carbonyl)amino)-3-phenylpropanoyl)-3-methyl-aziridine-2-carbonyl)pyrrolidine-2-carboxamido)acetate (0.695 g, 1.32 mmol). After vacuum filtration, extensive washing with dichloromethane and acetonitrile, and drying *in vacuo*, the product 2-((*S*)-1-((2*S*,3*S*)-1-((*S*)-2-ammonio-3-phenylpropanoyl)-3-methylaziridine-2-carbonyl)-pyrrolidine-2-carboxamido)acetate was isolated in 86% yield (0.455 g, 1.13 mmol) as an off-white solid. The crude product was shown to be approximately 94% pure by analytical HPLC (Elution Method A, Retention time = 2.903 min.). Although pure by HPLC, the deprotected tetrapeptide was not very soluble in DMSO-d_6. The ^{1}H and ^{13}C NMR spectra showed multiple conformations with many overlapping peaks. As a result, the NMR spectra of compound could not accurately be reported (see section 4 for the NMR spectra). HR-MS: m/z = 403.1958 $[M+H]^+$, calcd. 403.1976 for $C_{20}H_{27}N_4O_5$.

3.3. Cyclic Azy-containing Tetrapeptides and Ring-Opened Derivatives

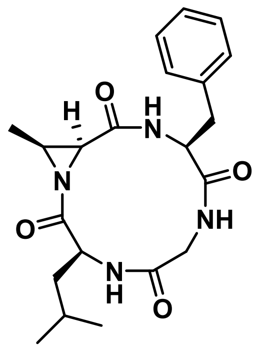

cyclo-[L-Cma-FG]

(3*S*,9*S*,12*S*,13*S*)-9-Benzyl-3-isobutyl-13-methyl-1,4,7,10-tetraazabicyclo[10.1.0]tridecane-2,5,8,11-tetraone (1)

Prepared as previously described.[2] IR (neat) cm^{-1}: 3282, 2954, 1691, 1662, 1533, 1417, 1388, 1321, 1284, 1132, 1031, 956, 847, 752, 699; ^{1}H NMR (400 MHz, DMSO-d_6): δ = 8.22 (br s, 1H), 7.82 (br s, 1H), 7.63 (t, *J* = 7.5 Hz, 1H), 7.28 – 6.89 (m, 5H), 4.87 (br s, 1H), 4.43 (br s, 1H), 3.56 (dd, *J* = 18.2, 8.6 Hz, 1H), 3.46 (br d, *J* = 13.4 Hz, 1H), 3.19 – 3.07 (m, 1H), 2.81 (br s, 1H), 2.67 – 2.50 (m, 2H), 1.76 – 1.61 (m, 1H), 1.58 – 1.40 (m, 2H), 0.84 (d, *J* = 6.5 Hz, 3H), 0.81 (d, *J* = 4.8 Hz, 3H), 0.74 (d, *J* = 6.4 Hz, 3H); ^{13}C NMR (150 MHz, DMSO-d_6): δ = 183.8, 173.0, 169.5, 135.3, 137.9, 129.5, 127.7, 125.9, 51.1, 50.2, 45.7, 40.9, 40.4, 37.2, 36.9, 24.0, 23.5, 20.7; HR-MS: *m/z* = 401.2204 [M+H]$^+$, calcd. 401.2189 for $C_{21}H_{29}N_4O_4$.

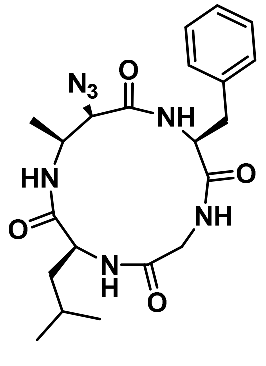

N_3-[L-Cma-FG]

(3*S*,9*S*,12*R*,13*S*)-12-Azido-9-benzyl-3-isobutyl-13-methyl-1,4,7,10-tetraazacyclotridecane-2,5,8,11-tetraone

Prepared as previously described.[2] ^{1}H NMR (400 MHz, DMSO-d_6): δ = 8.59 (bs, 1H), 8.16 (bs, 1H), 7.91 (bs, 1H), 7.37 – 7.04 (m, 6H), 4.55 (d, *J* = 2.9 Hz, 1H), 4.54 – 4.45 (m, 1H), 4.19 (td, *J* = 9.7, 6.2 Hz, 1H),

4.10 – 4.04 (m, 1H), 3.77 (dd, *J* = 14.2, 5.9 Hz, 1H), 3.51 (dd, *J* = 14.1, 6.5 Hz, 1H), 3.03 (ddd, *J* = 23.0, 13.8, 7.8 Hz, 2H), 1.64 – 1.47 (m, 2H), 1.44 – 1.32 (m, 1H), 1.13 (d, *J* = 6.7 Hz, 3H), 0.86 (dd, *J* = 20.1, 6.3 Hz, 6H); 13C NMR (100 MHz, DMSO-d_6): δ = 171.9, 170.1, 168.4, 166.8, 137.4, 129.0, 128.3, 126.5, 67.4, 55.1, 53.3, 48.5, 47.5, 43.3, 36.4, 30.1, 24.4, 22.6, 21.6, 17.6; HR-MS: *m/z* = 444.2359 $[M+H]^+$, calcd. 444.2359 for $C_{21}H_{30}N_7O_4$.

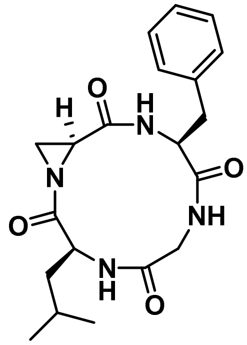

cyclo-[L-Azy-FG]

(3*S*,9*S*,12*S*)-9-Benzyl-3-isobutyl-1,4,7,10-tetraazabicyclo[10.1.0]tridecane-2,5,8,11-tetraone (2)

Prepared *via* the general procedure described in section 2.2 using linear tetrapeptide 2-((*S*)-2-((*S*)-1-((*S*)-2-Ammonio-4-methylpentanoyl)aziridine-2-carboxamido)-3-phenylpropanamido)acetate (100 mg, 0.25 mmol). After concentration *in vacuo* and precipitation/trituration from acetonitrile : water (5:95, *v/v*), the cyclic tetrapeptide (3*S*,9*S*,12*S*)-9-Benzyl-3-isobutyl-1,4,7,10-tetraazabicyclo[10.1.0]tridecane-2,5,8,11-tetraone (**2**) was isolated in 67% yield (65 mg, 0.17 mmol) as a pale-orange solid. ^{1}H NMR (500 MHz, DMSO-d_6) δ 8.33 (s, 1H), 7.92 (s, 1H), 7.64 (s, 1H), 7.18 (dd, *J* = 28.4, 7.9 Hz, 5H), 4.68 (s, 1H), 4.57 (s, 1H), 3.60 (s, 1H), 3.52 (d, *J* = 17.6 Hz, 1H), 3.32 (d, *J* = 40.0 Hz, 1H), 3.12 (s, 1H), 2.88 (s, 1H), 2.50 (d, *J* = 39.3 Hz, 1H), 2.14 (s, 1H), 1.73 (s, 1H), 1.58 (s, 2H), 0.90 (d, *J* = 6.4 Hz, 3H), 0.82 (d, *J* = 6.4 Hz, 3H); ^{13}C NMR (126 MHz, DMSO-d_6) δ 183.2, 173.1, 169.8, 166.5, 138.5, 130.0, 128.2, 126.4, 51.6, 46.2, 40.9, 37.1, 36.1, 30.1, 24.4, 23.8, 21.9, 21.3; HR-MS: *m/z* = 387.2013 $[M+H]^+$, calcd. 387.2027 for $C_{20}H_{27}N_4O_4$.

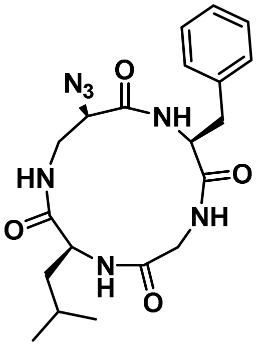

N_3-[L-Azy-FG]

(3*S*,9*S*,12*R*)-12-Azido-9-benzyl-3-isobutyl-1,4,7,10-tetraazacyclotridecane-2,5,8,11-tetraone (2a)

Prepared *via* the general procedure described in section 2.3.1, using Azy-containing cyclic tetrapeptide **2** (77 mg, 0.20 mmol). After precipitation, the product **2a** was isolated by vacuum filtration as a beige-brown solid (65 mg, 0.15 mmol) in 76% yield. ^{1}H NMR (400 MHz, DMSO-d_6) δ 8.35 (s, 1H), 8.23 (d, *J* = 7.4 Hz, 1H), 7.64 (d, *J* = 6.7 Hz, 1H), 7.39 – 7.08 (m, 5H), 6.87 (s, 1H), 4.58 (s, 1H), 4.36 (s, 1H), 4.28 (s, 1H), 4.01 (s, 1H), 3.80 (s, 1H), 3.36 (d, *J* = 12.6 Hz, 1H), 3.27 – 3.17 (m, 3H), 3.17 – 3.06 (m, 1H), 3.00 – 2.89 (m, 1H), 1.70 (s, 1H), 1.55 (s, 1H), 1.40 (s, 1H), 0.98 – 0.75 (m, 6H); ^{13}C NMR (126 MHz, DMSO-d_6) δ 171.4, 170.8, 169.0, 167.4, 137.7, 129.0, 128.2, 126.3, 63.0, 54.3, 50.6, 43.9, 40.4, 35.1, 24.1, 23.2, 21.4; HR-MS: *m/z* = 430.2182 $[M+H]^+$, calcd. 430.2197 for $C_{20}H_{28}N_7O_4$.

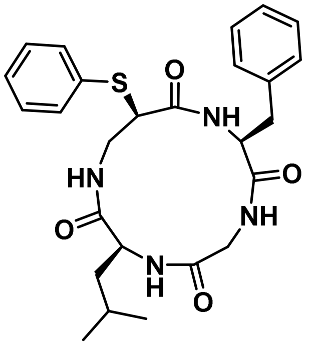

PhS-[L-Azy-FG]

(3*S*,9*S*,12*R*)-9-Benzyl-3-isobutyl-12-(phenylthio)-1,4,7,10-tetraazacyclotridecane-2,5,8,11-tetraone (2b)

Prepared *via* the general procedure described in section 2.3.2, using Azy-containing cyclic tetrapeptide **2** (19.3 mg, 0.05 mmol). The product **2b** was isolated by reverse-phase Combiflash® as described in section 1.2 using a gradient of 30 – 80% B to yield a white solid (10.5 mg, 0.02 mmol) in 42% yield. ^{1}H NMR (500 MHz, DMSO-d_6) δ 8.56 (t, *J* = 7.2 Hz, 1H), 8.54 (d, *J* = 8.4 Hz, 1H), 8.17 (d, *J* = 8.8 Hz, 1H), 7.36 – 7.06 (m, 10H), 7.23 (m, 1H), 4.28 (td, *J* = 9.0, 5.7 Hz, 1H), 4.14 (td, *J* = 10.1, 4.3 Hz, 1H), 3.93 (dd, *J* = 13.8, 7.3 Hz, 1H), 3.76 (dd, *J* = 8.5, 4.6 Hz, 1H), 3.60 (m, 1H), 3.46 (m, 1H), 3.33 (dd, *J* = 9.2, 3.7 Hz, 1H), 3.02 (d, *J* = 7.7 Hz, 2H), 1.53 (ddd, *J* = 17.4, 10.2, 5.6 Hz, 2H), 1.44 (m, 1H), 0.86 (d, *J* = 6.1 Hz, 3H), 0.81 (d, *J* = 5.9 Hz, 3H); ^{13}C NMR (126 MHz, DMSO-d_6) δ 172.1,

171.4, 170.1, 169.4, 138.6, 134.1, 130.6, 129.6, 129.5, 128.7, 128.6, 127.6, 67.8, 56.2, 52.1, 50.9, 44.3, 34.9, 24.7, 23.4, 21.8, 14.3; HR-MS: *m/z* = 497.2215 $[M+H]^+$, calcd. 497.2217 for $C_{26}H_{33}N_4O_4S$.

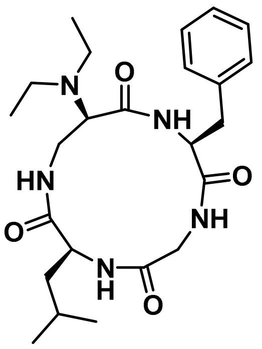

Et₂N-[L-Azy-FG]

(3*S*,9*S*,12*R*)-9-Benzyl-12-(diethylamino)-3-isobutyl-1,4,7,10-tetraazacyclotridecane-2,5,8,11-tetraone (2c)

Cyclic tetrapeptide **2** (19.3 mg, 0.05 mmol, 1 eq.) was dissolved in 1:1 DCM:MeCN (5 mL each, 0.02 M) followed by the addition of diethylamine (31 µL, 0.30 mmol, 6 eq.) The reaction was stirred at room temperature for 20h, followed by stirring at 40 °C for 20 h. The solvent and volatiles were removed *in vacuo* to yield the product **2c** a white solid (14.1 mg, 0.03 mmol) in 61% yield. ^{1}H NMR (500 MHz, DMSO-d_6) δ 8.32 (s, 1H), 7.96 (d, *J* = 9.3 Hz, 2H), 7.31 – 7.09 (m, 5H), 6.65 (t, *J* = 5.6 Hz, 1H), 4.51 (td, *J* = 9.7, 5.6 Hz, 1H), 4.17 (ddd, *J* = 10.7, 8.7, 3.7 Hz, 1H), 4.02 (dd, *J* = 14.2, 7.7 Hz, 1H), 3.51 (dt, *J* = 13.1, 6.3 Hz, 1H), 3.14 – 3.07 (m, 2H), 3.02 – 2.93 (m, 2H), 2.38 (q, *J* = 6.8 Hz, 2H), 2.28 (q, *J* = 7.0 Hz, 2H), 1.61 – 1.49 (m, 2H), 1.43 (dd, *J* = 10.7, 8.8 Hz, 1H), 0.88 (d, *J* = 5.9 Hz, 3H), 0.84 (d, *J* = 7.1 Hz, 6H), 0.82 (d, *J* = 6.0 Hz, 3H); ^{13}C NMR (126 MHz, DMSO-d_6) δ 172.1, 171.9, 171.8, 169.6, 138.4, 129.4, 128.6, 126.7, 65.5, 54.6, 52.0, 44.3, 42.4, 41.9, 38.1, 35.6, 24.7, 23.5, 21.6, 11.4; HR-MS: *m/z* = 460.2912 $[M+H]^+$, calcd. 460.2918 for $C_{24}H_{38}N_5O_4$.

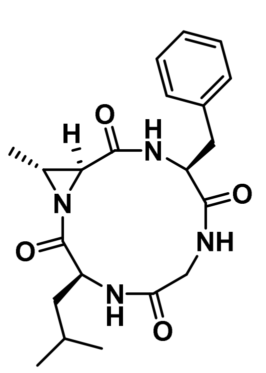

cyclo-[L-Tma-FG]

(3*S*,9*S*,12*S*,13*R*)-9-Benzyl-3-isobutyl-13-methyl-1,4,7,10-tetraazabicyclo[10.1.0]tridecane-2,5,8,11-tetraone (3)

Linear peptide H-Leu-Tma-Phe-Gly-OH was prepared according to the general procedure described in section 2.1 using Fmoc-Tma-OH and other Fmoc-protected amino acids. Linear peptide H-Leu-Tma-Phe-Gly-OH (65.6 mg, 0.16 mmol) was cyclized according to the general procedure described in section 2.2. The peptide was triturated with methanol (20 mL) to yield cyclo-[L-Tma-F-G] as a white solid (31.6 mg, 0.079 mmol) in 49% yield. ^{1}H NMR (500 MHz, DMSO-d_6) δ 8.24 (br s, 1H), 7.72 (br s, 1H), 7.63 (br s, 1H), 7.26 – 7.09 (m, 5H), 4.68 (br s, 2H), 3.50 (br s, 2H), 3.10 (br s, 1H), 2.74 (s, 1H), 2.54 (br s, 2H), 1.75 (br s, 1H), 1.47 (br s, 2H), 1.24 (s, 3H), 0.84 (d, J = 6.3 Hz, 6H); ^{13}C NMR (126 MHz, DMSO-d_6) δ 180.4, 173.5, 169.7, 166.6, 138.5, 130.0, 128.3, 126.4, 51.5, 50.0, 46.1, 41.8, 37.3, 24.7, 24.3, 23.9, 21.5, 14.8; HR-MS: m/z = 401.2192 [M+H]$^+$, calcd. 401.2189 for $C_{21}H_{29}N_4O_4$.

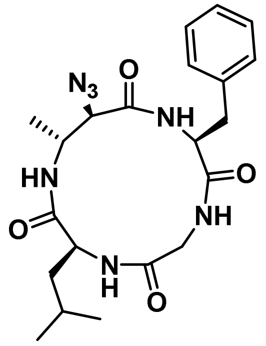

N_3-[L-Tma-FG]

(3*S*,9*S*,12*R*,13*R*)-12-Azido-9-benzyl-3-isobutyl-13-methyl-1,4,7,10-tetraazacyclotridecane-2,5,8,11-tetraone (3a)

Cyclo-[L-Tma-F-G] (10 mg, 0.025 mmol) was subjected to aziridine ring-opening by sodium azide as described in section 2.3.1. The product was isolated by reverse-phase Combiflash® as described in section 1.2 using a gradient of 20 – 55% B to yield a white solid (5.2 mg, 0.012 mmol) in 47% yield. ^{1}H NMR (700 MHz, DMSO-d_6) δ 8.55 (s, 1H), 8.37 (d, J = 8.8 Hz, 1H), 7.59 (dd, J = 7.9, 4.2 Hz, 1H), 7.27 – 7.13 (m, 5H), 6.74 (d, J = 9.7 Hz, 1H), 4.54 (td, J = 9.7, 5.4 Hz, 1H), 4.30 – 4.24 (m, 1H), 4.24 – 4.20 (m, 1H), 4.10 (d, J = 4.5 Hz, 1H), 3.92 (dd, J = 15.2, 7.3 Hz, 1H), 3.38 (dd, J = 15.2, 3.6 Hz, 1H), 3.12 (dd, J = 13.9, 5.4 Hz, 1H), 2.98 (dd, J = 13.9, 10.2 Hz, 1H), 1.78 – 1.72 (m, 1H), 1.37 – 1.27 (m, 2H), 1.09 (d, J = 6.9 Hz, 3H), 0.87 (d, J = 6.7 Hz, 3H), 0.85 (d, J = 6.5 Hz, 3H); ^{13}C NMR (126 MHz, DMSO-d_6) δ 171.8, 170.9, 169.6, 167.0, 138.3, 129.4, 128.7, 126.7, 67.6, 54.9, 50.5, 46.3, 44.5, 36.2, 34.9, 24.6, 23.8, 21.9, 18.6; HR-MS: m/z = 444.2353 [M+H]$^+$, calcd. 444.2354 for $C_{21}H_{30}N_7O_4$.

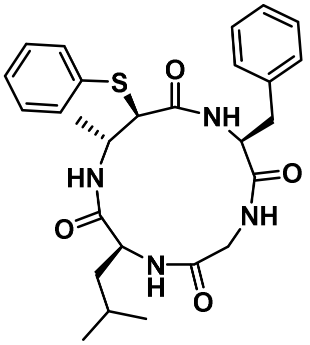

PhS-[L-Tma-FG]

(3*S*,9*S*,12*R*,13*R*)-9-Benzyl-3-isobutyl-13-methyl-12-(phenylthio)-1,4,7,10-tetraazacyclotridecane-2,5,8,11-tetraone (3b)

Cyclo-[L-Tma-F-G] (10 mg, 0.025 mmol) was subjected to aziridine ring-opening by thiophenol as described in section 2.3.2. The product was isolated by reverse-phase Combiflash® as described in section 1.2 using a gradient of 30 – 80% B to yield a white solid (4.4 mg, 0.009 mmol) in 34% yield. ^{1}H NMR (500 MHz, DMSO-d_6) δ 8.52 (d, J = 9.1 Hz, 1H), 8.46 (br s, 1H), 7.51 (br s, 1H), 7.50 – 7.24 (m, 10H), 7.16 (br s, 1H), 4.41 (br s, 1H), 4.34 (d, J = 6.6 Hz, 1H), 4.26 (td, J = 9.3, 5.0 Hz, 1H), 4.00 (dd, J = 15.0, 7.7 Hz, 1H), 3.68 (d, J = 6.4 Hz, 1H), 3.36 (dd, J = 14.9, 3.5 Hz, 1H), 3.18 – 3.07 (m, 1H), 3.05 – 2.94 (m, 1H), 1.74 (ddd, J = 13.9, 9.1, 5.1 Hz, 1H), 1.58 – 1.47 (m, 1H), 1.37 (ddd, J = 14.0, 9.6, 4.8 Hz, 1H), 1.19 (d, J = 6.8 Hz, 3H), 0.89 (d, J = 6.6 Hz, 3H), 0.86 (d, J = 6.5 Hz, 3H); ^{13}C NMR (126 MHz, DMSO-d_6) δ 172.1, 172.0, 170.7, 169.7, 134.5, 130.2, 129.5, 129.4, 128.6, 127.3, 126.5, 125.8, 62.6, 55.6, 50.8, 46.8, 44.4, 34.6, 27.2, 24.7, 23.6, 22.0, 20.4; HR-MS: m/z = 511.2370 $[M+H]^+$, calcd. 511.2374 for $C_{27}H_{35}N_4O_4S$.

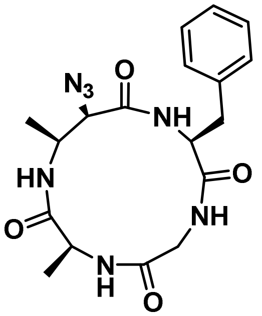

N_3-[A-Cma-FG]

(3*S*,9*S*,12*R*,13*S*)-12-Azido-9-benzyl-3,13-dimethyl-1,4,7,10-tetraazacyclotridecane-2,5,8,11-tetraone (4a)

Linear peptide H-Ala-Cma-Phe-Gly-OH was prepared according to the general procedure described in section 2.1 using Fmoc-Cma-OH and other Fmoc-protected amino acids. Linear peptide H-Ala-Cma-Phe-Gly-OH (21.6 mg, 0.057 mmol) was cyclized according to the general procedure described in section 2.2. Without further purification, the peptide was subjected to aziridine ring-opening with sodium azide as described in section 2.3.1. The product was isolated by reverse-phase Combiflash® as described in

section 1.2 using a gradient of 10 – 40% B to yield a white solid (10.9 mg, 0.027 mmol) in 48% yield over two steps. ^{1}H NMR (500 MHz, DMSO-d_6) δ 9.38 (br s, 1H), 8.93 (br s, 1H), 8.69 (br s, 1H), 7.45 (d, *J* = 7.5 Hz, 1H), 7.31 – 7.13 (m, 5H), 4.45 (d, *J* = 2.8 Hz, 1H), 4.36 (td, *J* = 9.7, 5.7 Hz, 1H), 4.15 (dq, *J* = 9.8, 7.3 Hz, 1H), 3.98 (pd, *J* = 6.7, 3.0 Hz, 1H), 3.73 (ddd, *J* = 14.1, 8.5, 1.8 Hz, 1H), 3.49 (dt, *J* = 11.6, 3.3 Hz, 1H), 3.09 – 2.98 (m, 2H), 1.20 (d, *J* = 7.3 Hz, 3H), 1.09 (d, *J* = 6.6 Hz, 3H); ^{13}C NMR (126 MHz, DMSO-d_6) δ 173.3, 171.3, 168.6, 167.5, 138.1, 129.5, 128.7, 126.9, 67.9, 57.0, 50.7, 48.0, 43.7, 37.0, 18.2, 18.0; HR-MS: *m/z* = 402.1892 $[M+H]^+$, calcd. 402.1884 for $C_{18}H_{24}N_7O_4$.

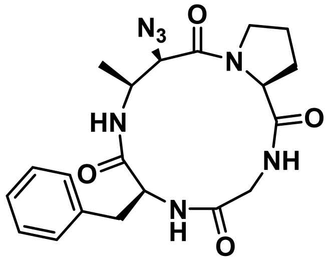

N_3-[F-Cma-PG]

(6*S*,9*S*,10*R*,15a*S*)-10-Azido-6-benzyl-9-methyldecahydro-1H-pyrrolo[1,2-a][1,4,7,10]tetra-azacyclotridecine-1,4,7,11(8H)-tetraone (5a)

Linear tetrapeptide 2-((*S*)-1-((2*S*,3*S*)-1-((*S*)-2-ammonio-3-phenylpropanoyl)-3-methylaziridine-2-carbonyl)-pyrrolidine-2-carboxamido)acetate (0.080 g, 0.20 mmol) was cyclized according to the general procedure described in section 2.2. Without working up the cyclization reaction mixture, sodium azide (0.13 g, 2.00 mmol) was added directly to the reaction mixture. After concentrating *in vacuo*, the crude residue was transferred to a small reaction vessel in which DMF (4 mL) and excess NaN_3 (0.13 g, 2.00 mmol) were added. The system was then stirred at 60 °C for 24 hours. The suspension was then concentrated *in vacuo* and the product was purified by preparative HPLC as described in section 2.1 using a gradient elution of 15% to 35% B. This yielded **5a** (0.040 g, 0.094 mmol) as a fluffy white solid (after lyophilization) in 45% yield over 2 steps. The product was shown to exist in two distinct conformations on the NMR time scale in DMSO-d_6 at 25 °C in a ratio of 1.00 : 0.29. The major conformer is reported: ^{1}H NMR (500 MHz, DMSO-*d*6) δ 9.19 (s, 1H), 8.70 (d, *J* = 8.6 Hz, 1H), 7.57 (s, 1H), 7.31 – 7.17 (m, 5H), 4.65 (dd, *J* = 8.6, 2.3 Hz, 1H), 4.26 (ddd, *J* = 10.8, 8.2, 4.3 Hz, 1H), 4.18 – 4.10 (m, 1H), 3.94 – 3.85 (m, 1H), 3.53 – 3.47 (m, 1H), 3.47 – 3.41 (m, 1H), 3.39 – 3.36 (m, 1H), 2.98 (dd, *J* = 14.0, 4.3 Hz, 1H), 2.88 (dd, *J* = 14.0, 10.8 Hz, 1H), 2.24 (dddd, *J* = 12.7, 11.0, 8.4, 7.0 Hz, 1H), 2.01 (ddt, *J* = 9.4, 6.4, 3.1 Hz, 1H), 1.88 – 1.81 (m, 1H), 1.74 – 1.62 (m, 1H), 1.23 (d, *J* = 7.0 Hz, 3H); ^{13}C NMR (126 MHz, DMSO) δ 174.1, 170.1, 169.6, 166.0, 138.0, 128.9, 128.2, 126.4, 61.3, 60.1, 56.8, 47.6, 46.9, 44.1, 35.9, 31.8, 21.9, 17.4; HR-MS: *m/z* = 428.2040 $[M+H]^+$, calcd. 428.2041 for $C_{20}H_{26}N_7O_4$.

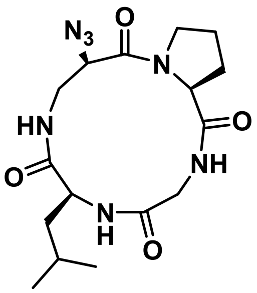

N_3-[L-Azy-PG]

(6*S*,10*R*,15a*S*)-10-Azido-6-isobutyldecahydro-1H-pyrrolo[1,2-a][1,4,7,10]tetraazacyclotridecine-1,4,7,11(8H)-tetraone (6a)

Linear peptide H-Leu-Azy-Pro-Gly-OH was prepared according to the general procedure described in section 2.1 using Fmoc-Cma-OH and other Fmoc-protected amino acids. Linear peptide H-Ala-Cma-Phe-Gly-OH (36.1 mg, 0.10 mmol) was cyclized according to the general procedure described in section 2.2. Without further purification, the peptide was subjected to aziridine ring-opening with sodium azide as described in section 2.3.1. The product was isolated by reverse-phase Combiflash® as described in section 1.2 using a gradient of 5 – 20% B to yield a white solid (25.2 mg, 0.066 mmol) in 66% yield over two steps. ^{1}H NMR (700 MHz, DMSO-d_6) δ 9.12 (t, J = 6.2 Hz, 1H), 8.65 (d, J = 7.8 Hz, 1H), 7.36 (d, J = 9.5 Hz, 1H), 4.50 (dd, J = 8.7, 2.6 Hz, 1H), 4.00 (ddd, J = 10.5, 7.7, 5.2 Hz, 1H), 3.83 – 3.80 (m, 1H), 3.80 – 3.78 (m, 1H), 3.59 – 3.53 (m, 1H), 3.50 (dd, J = 13.3, 6.7 Hz, 1H), 3.41 (ddd, J = 11.8, 8.7, 7.3 Hz, 1H), 3.34 (ddd, J = 13.1, 9.1, 2.0 Hz, 1H), 3.16 (dd, J = 9.2, 2.0 Hz, 1H), 2.25 (dddd, J = 12.8, 10.6, 8.5, 7.0 Hz, 1H), 1.96 (ddt, J = 12.9, 6.5, 3.1 Hz, 1H), 1.84 (dtd, J = 8.7, 7.2, 3.6 Hz, 1H), 1.71 (ddt, J = 12.8, 10.8, 7.8 Hz, 1H), 1.67 – 1.59 (m, 1H), 1.51 (ddd, J = 13.7, 10.5, 5.3 Hz, 1H), 1.38 (ddd, J = 13.9, 8.9, 5.3 Hz, 1H), 0.86 (d, J = 6.6 Hz, 3H), 0.80 (d, J = 6.6 Hz, 3H); ^{13}C NMR (126 MHz, DMSO-d_6) δ 174.0, 172.5, 170.2, 168.0, 60.5, 59.3, 54.3, 47.4, 44.4, 31.9, 24.8, 23.2, 22.6, 21.6; HR-MS: m/z = 380.2047 [M+H]$^+$, calcd. 380.2041 for $C_{16}H_{26}N_7O_4$.

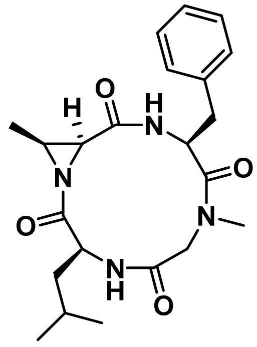

cyclo-[L-Cma-F-Sar]

(3*S*,9*S*,12*S*,13*S*)-9-Benzyl-3-isobutyl-7,13-dimethyl-1,4,7,10-tetraazabicyclo[10.1.0]tri-decane-2,5,8,11-tetraone (7)

Prepared *via* the general procedure described in section 2.2 using linear tetrapeptide 2-((*S*)-2-((2*S*,3*S*)-1-((*S*)-2-ammonio-4-methylpentanoyl)-3-methylaziridine-2-carboxamido)-*N*-methyl-3-phenylpropanamido)acetate (0.108 g, 0.25 mmol). After concentration *in vacuo* and precipitation/trituration from acetonitrile : water (5:95, *v/v*), cyclic tetrapeptide **7** was isolated in 83% yield (0.086 g, 0.21 mmol) as a pale-orange solid. The crude product was shown to be approximately 92% pure by analytical HPLC (Elution Method B, Retention time = 12.609 min.). An analytically pure sample of the cyclic peptide **7** was obtained by preparative HPLC as described in section 2.1 using a gradient elution of 15% to 35% B. After lyophilization, the desired product was isolated as a white fluffy solid. The product exists in two distinct conformations on the NMR time scale in DMSO-d_6 at 25 °C in a ratio of 1.00 : 0.20. The major conformer is reported: ^{1}H NMR (500 MHz, DMSO-d_6) δ 8.33 (d, *J* = 9.7 Hz, 1H), 7.84 (d, *J* = 9.6 Hz, 1H), 7.33 – 7.10 (m, 5H), 5.03 (td, *J* = 9.1, 5.1 Hz, 1H), 4.56 – 4.51 (m, 1H), 3.87 (d, *J* = 18.6 Hz, 1H), 3.67 (d, *J* = 18.7 Hz, 1H), 3.22 (dd, *J* = 14.1, 5.5 Hz, 1H), 2.88 (d, *J* = 6.5 Hz, 1H), 2.83 (s, 3H), 2.72 – 2.68 (m, 1H), 2.69 – 2.61 (m, 1H), 1.85 – 1.73 (m, 1H), 1.63 – 1.45 (m, 2H), 0.94 – 0.89 (m, 6H), 0.83 (d, *J* = 6.5 Hz, 3H); ^{13}C NMR (126 MHz, DMSO) δ 183.6, 171.3, 168.2, 165.2, 137.9, 129.7, 127.7, 126.0, 57.5, 53.3, 51.2, 50.9, 40.9, 37.9, 37.1, 35.6, 24.3, 23.4, 20.9, 13.1. HR-MS: *m/z* = 415.2325 [M+H]$^+$, calcd. 415.2340 for $C_{22}H_{31}N_4O_4$.

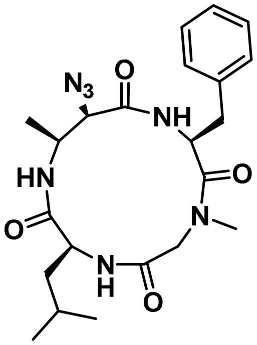

N3-[L-Cma-F-Sar]

(3*S*,9*S*,12*R*,13*S*)-12-Azido-9-benzyl-3-isobutyl-7,13-dimethyl-1,4,7,10-tetraazacyclotri-decane-2,5,8,11-tetraone (7a)

Cyclic peptide **7** (0.080 mg, 0.20 mmol) was subjected to aziridine ring-opening by sodium azide as described in section 2.3.1. The product was isolated by trituration with water followed by filtration and dried *in vacuo* to afford cyclic peptide **7a** (0.075 mg, 85%) as a beige powder. The product exists in two distinct conformations on the NMR time scale in DMSO-d_6 at 25 °C in a ratio of 1.00 : 0.09. Major conformation in DMSO-d_6: ^{1}H NMR (700 MHz, DMSO-d_6) δ 7.79 (d, *J* = 9.7 Hz, 1H), 7.41 (d, *J* = 8.3 Hz, 1H), 7.30 (t, *J* = 7.6 Hz, 2H), 7.28 – 7.21 (m, 2H), 7.18 – 7.14 (m, 2H), 4.62 (d, *J* = 3.2 Hz, 0H), 4.49 – 4.43 (m, 1H), 4.27 (td, *J* = 10.0, 6.2 Hz, 1H), 4.20 (ddd, *J* = 8.4, 6.7, 3.6 Hz, 1H), 3.52 (d, *J* = 18.6 Hz, 1H), 3.38 (d, *J* = 18.9 Hz, 1H), 3.02 – 2.88 (m, 2H), 2.84 (s, 1H), 1.56 (ddd, *J* = 13.3, 10.2, 5.6 Hz, 1H), 1.45 (dt, *J* = 13.2, 6.7 Hz, 1H), 1.38 (ddd, *J* = 13.8, 8.1, 6.2 Hz, 1H), 1.16 (d, *J* = 6.4 Hz, 2H), 0.86 (d, *J* = 6.6 Hz, 3H), 0.79 (d, *J* = 6.3 Hz, 2H); Minor conformation (partial assignment) in DMSO-d_6: ^{1}H NMR (700 MHz, DMSO-d_6) δ 8.03 (d, *J* = 9.8 Hz, 1H), 7.32 – 7.31 (m, 4H), 7.24 – 7.24 (m, 13H), 5.17 (ddd, *J* = 9.8, 8.4, 6.5 Hz, 1H), 4.86 (d, *J* = 13.7 Hz, 1H), 4.51 (d, *J* = 2.4 Hz, 1H), 4.32 (td, *J* = 10.0, 6.5 Hz, 1H), 4.02 – 3.96 (m, 1H), 3.11 (dd, *J* = 13.7, 6.5 Hz, 1H), 2.96 (d, *J* = 5.0 Hz, 5H), 2.91 (s, 1H), 1.12

(d, J = 6.5 Hz, 2H); Major conformation in $CDCl_3$: ^{1}H NMR (700 MHz, $CDCl_3$) δ 7.27 (d, J = 6.5 Hz, 1H), 6.71 (d, J = 10.1 Hz, 1H), 5.65 (d, J = 7.8 Hz, 1H), 5.26 – 5.19 (m, 1H), 4.77 (d, J = 13.4 Hz, 1H), 4.56 – 4.50 (m, 2H), 4.12 (dd, J = 2.9, 0.8 Hz, 1H), 3.15 (dd, J = 13.2, 9.5 Hz, 1H), 3.02 (dd, J = 13.2, 6.1 Hz, 1H), 2.95 (dd, J = 13.5, 0.8 Hz, 1H), 2.92 (d, J = 0.8 Hz, 3H), 1.67 – 1.57 (m, 2H), 1.50 – 1.45 (m, 2H), 1.36 (dd, J = 6.7, 0.9 Hz, 3H), 0.95 (dd, J = 6.6, 0.8 Hz, 3H), 0.92 (dd, J = 6.6, 0.8 Hz, 3H); Minor conformation in $CDCl_3$: ^{1}H NMR (700 MHz, $CDCl_3$) δ 7.48 (d, J = 9.4 Hz, 1H), 7.15 (d, J = 7.2 Hz, 1H), 7.08 (d, J = 8.2 Hz, 1H), 4.79 (q, J = 7.1 Hz, 1H), 4.64 (dqd, J = 13.7, 6.9, 3.3 Hz, 1H), 4.36 (td, J = 9.1, 6.4 Hz, 1H), 4.13 (d, J = 3.4 Hz, 1H), 3.92 (d, J = 18.5 Hz, 1H), 3.32 (d, J = 18.6 Hz, 1H), 3.12 – 3.10 (m, 2H), 2.92 (d, J = 0.8 Hz, 3H), 1.70 (ddd, J = 13.6, 9.7, 6.0 Hz, 1H), 1.66 – 1.59 (m, 2H), 1.54 (dt, J = 20.2, 6.5 Hz, 1H), 1.33 (d, J = 6.8 Hz, 3H), 0.92 (dd, J = 6.6, 0.8 Hz, 3H), 0.88 (dd, J = 6.6, 0.8 Hz, 3H); Major conformation only in DMSO-d_6: ^{13}C NMR (126 MHz, DMSO) δ 171.4, 170.6, 166.8, 166.5, 136.3, 128.9, 128.5, 126.9, 67.5, 53.3, 52.6, 50.9, 47.2, 35.3, 24.5, 22.6, 21.6, 17.6; HR-MS: m/z = 458.2514 $[M+H]^+$, calcd. 458.2510 for $C_{22}H_{32}N_7O_4$.

4. NMR Spectra

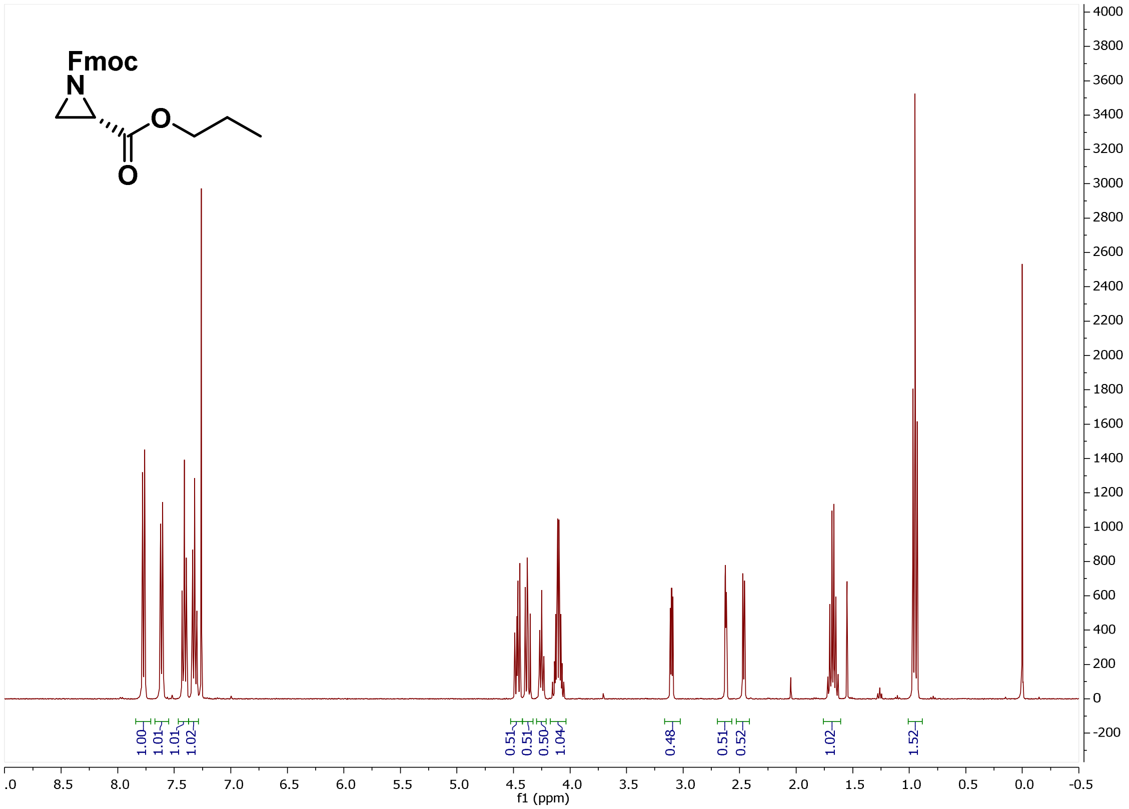

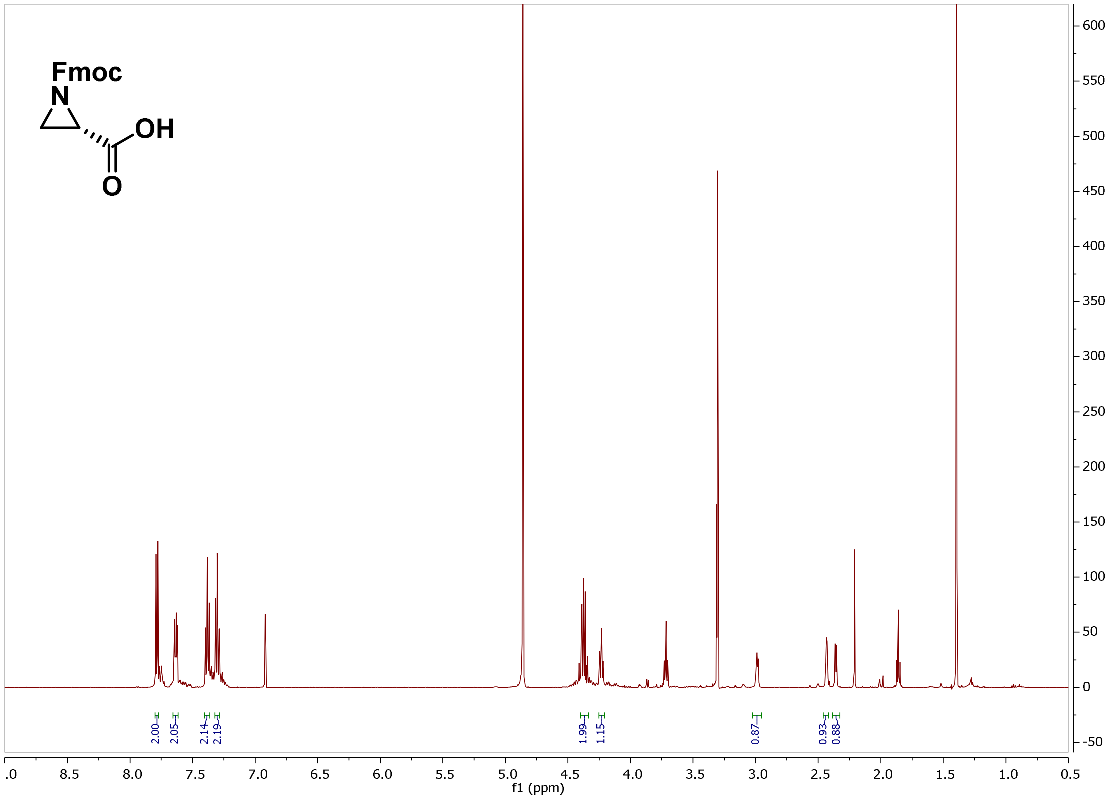
Fmoc
N
OH
O
f1 (ppm)

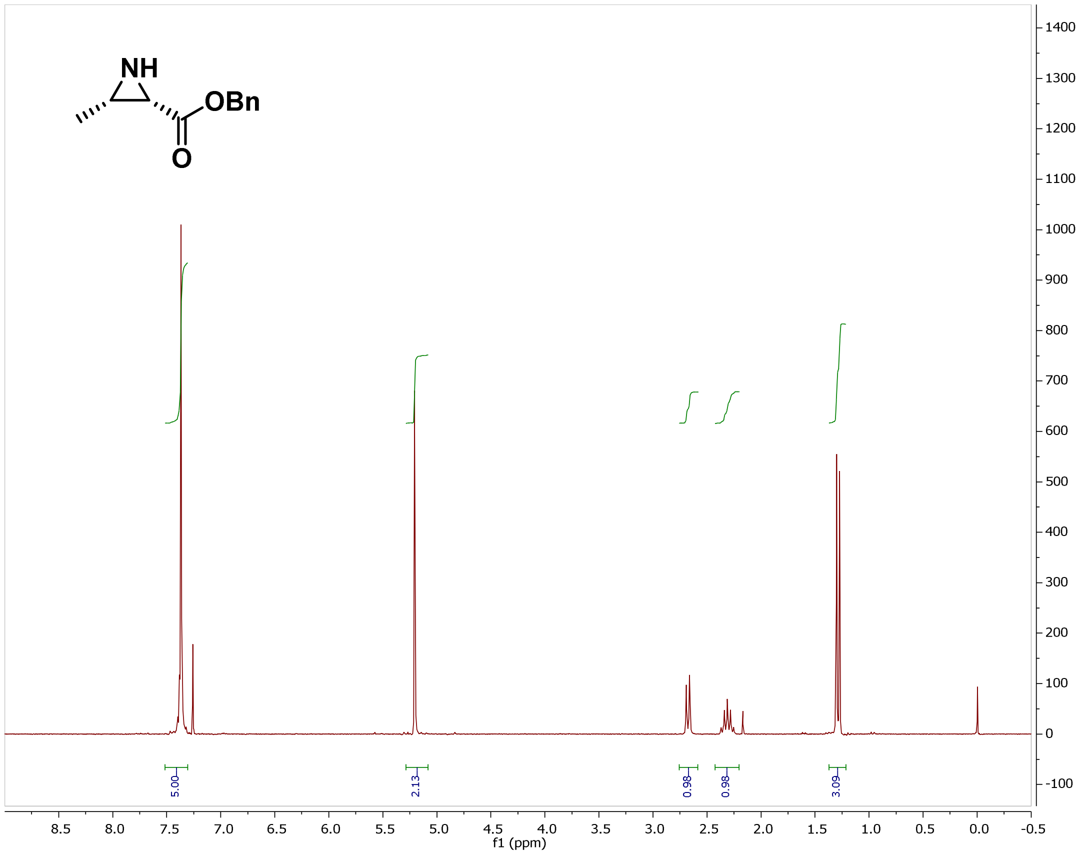
NH
OBn
O
f1 (ppm)

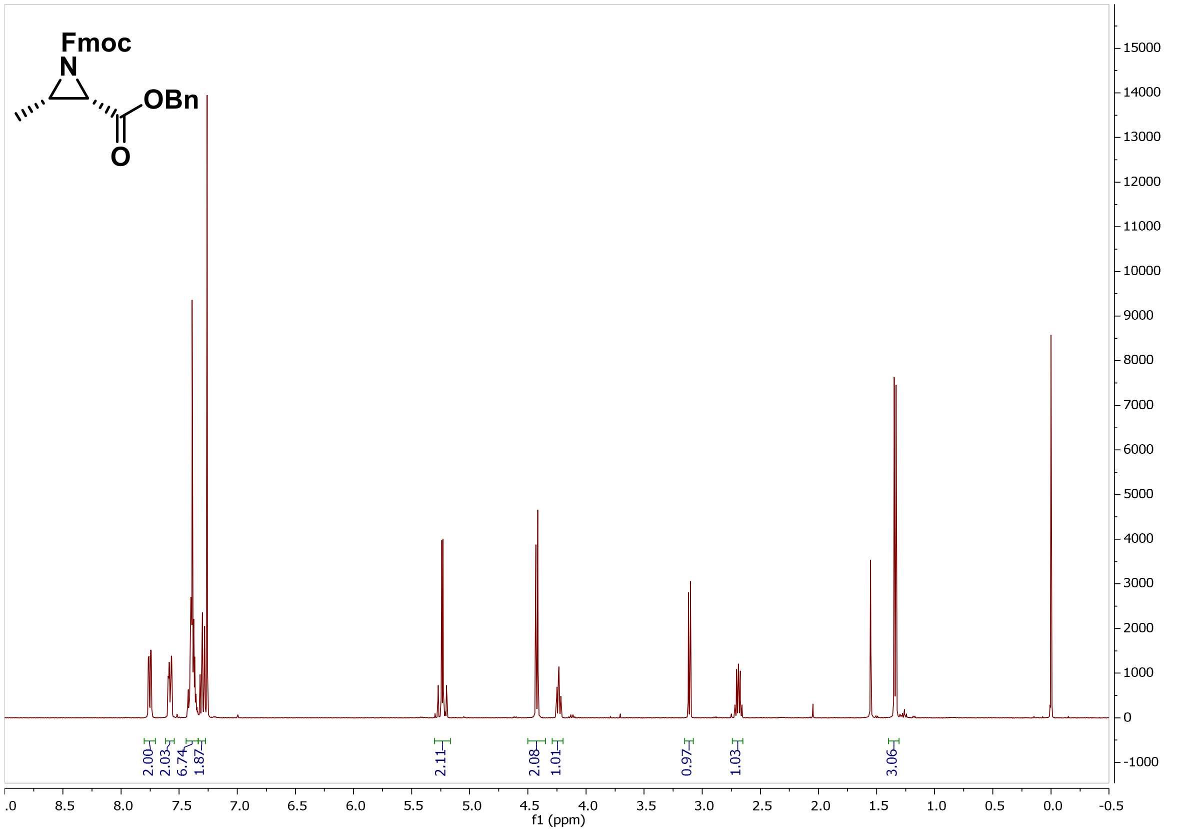

Fmoc
N
OBn
O
2.00
2.03
6.74
1.87
2.11
2.08
1.01
0.97
1.03
3.06
f1 (ppm)

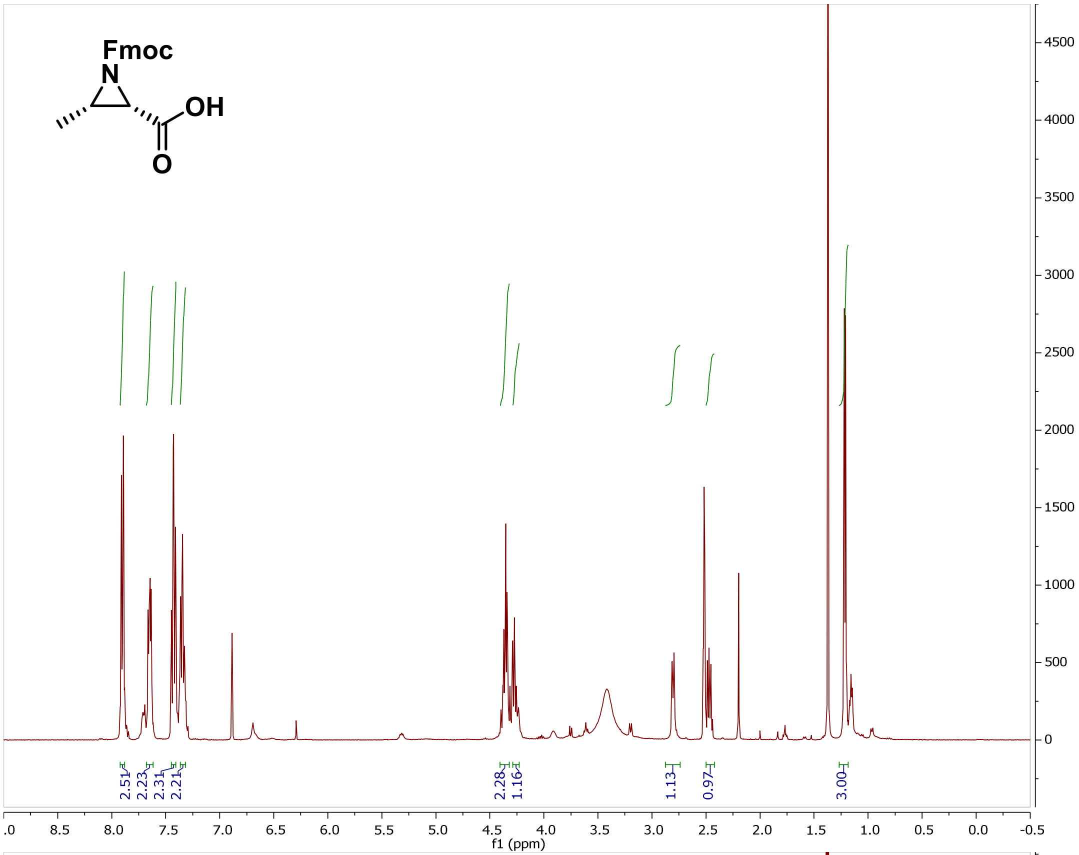
Fmoc
N
OH
O
2.51
2.23
2.31
2.21
2.28
1.16
1.13
0.97
3.00
4500
4000
3500
3000
2500
2000
1500
1000
500
0
.0 8.5 8.0 7.5 7.0 6.5 6.0 5.5 5.0 4.5 4.0 3.5 3.0 2.5 2.0 1.5 1.0 0.5 0.0 -0.5
f1 (ppm)

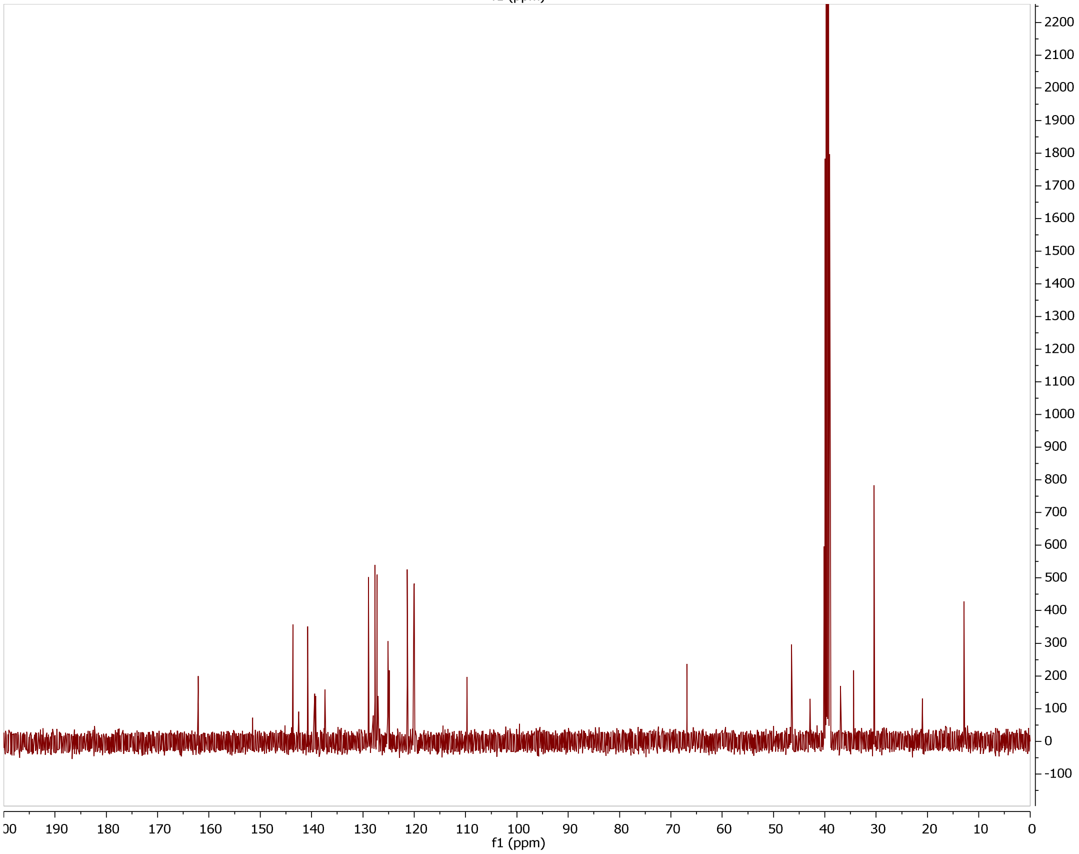
2200
2100
2000
1900
1800
1700
1600
1500
1400
1300
1200
1100
1000
900
800
700
600
500
400
300
200
100
0
-100
00 190 180 170 160 150 140 130 120 110 100 90 80 70 60 50 40 30 20 10 0
f1 (ppm)

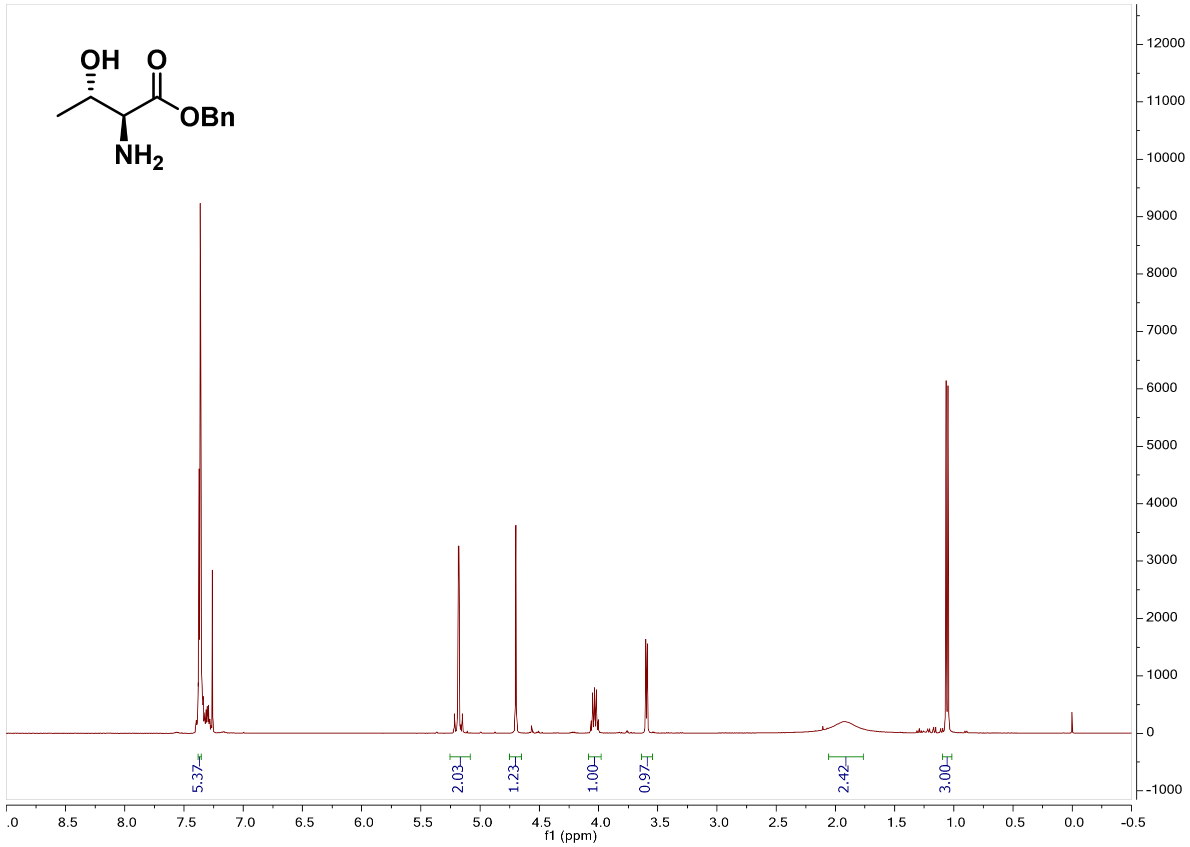
OH
O
OBn
NH2
5.37
2.03
1.23
1.00
0.97
2.42
3.00
f1 (ppm)

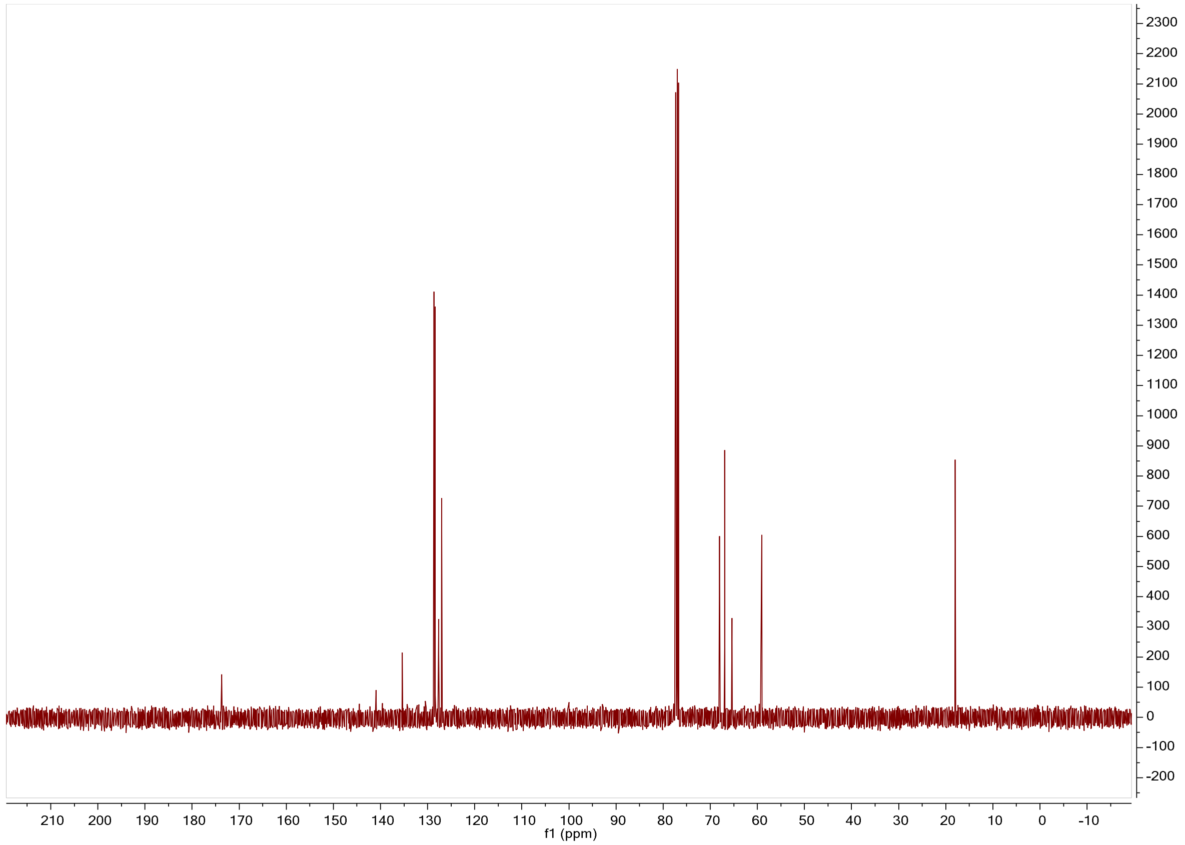
f1 (ppm)

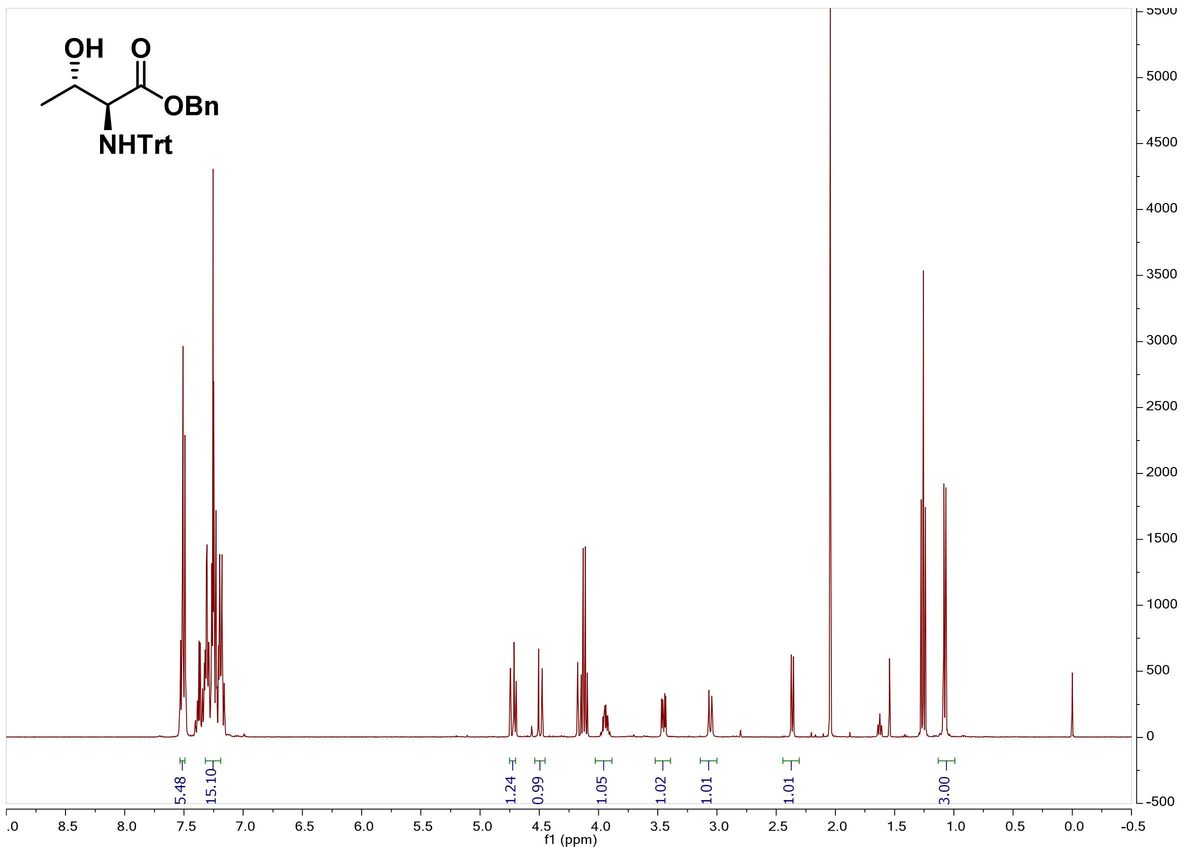
OH
O
OBn
NHTrt
5.48
15.10
1.24
0.99
1.05
1.02
1.01
1.01
3.00
f1 (ppm)

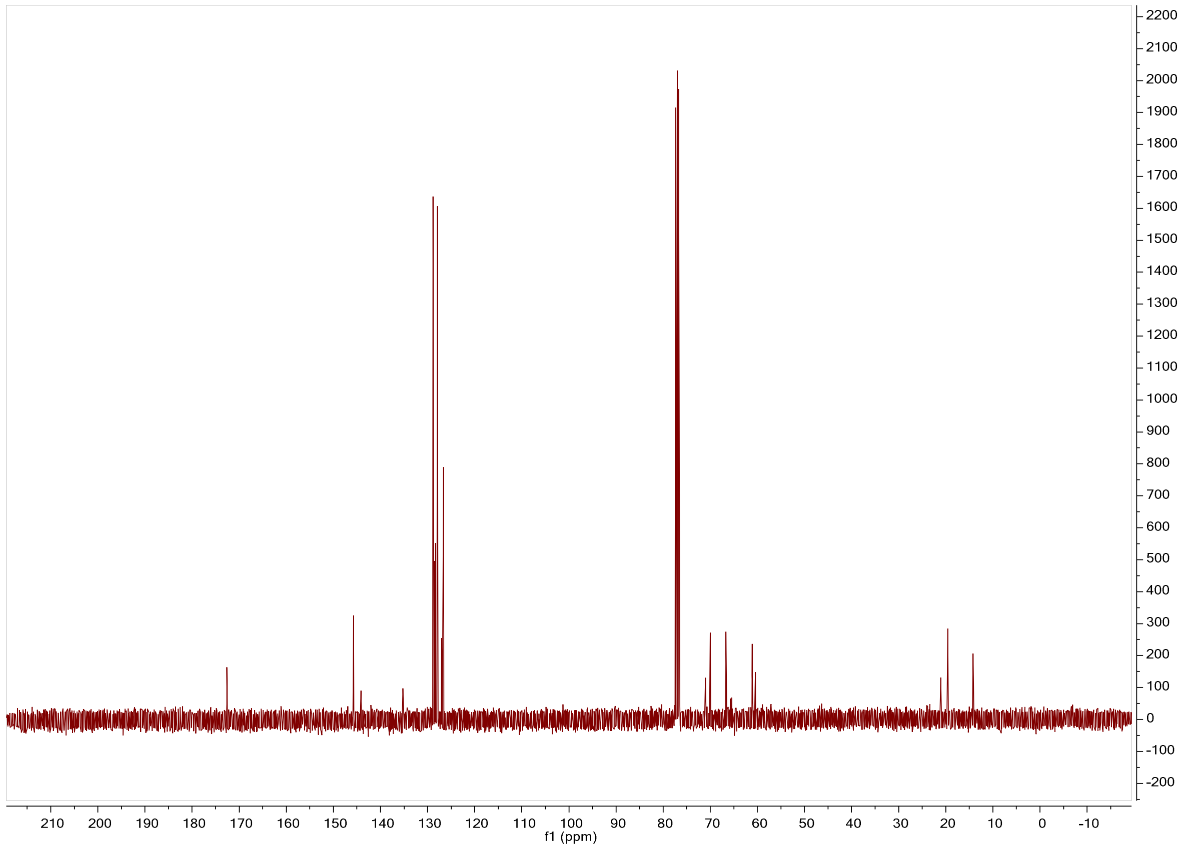
f1 (ppm)

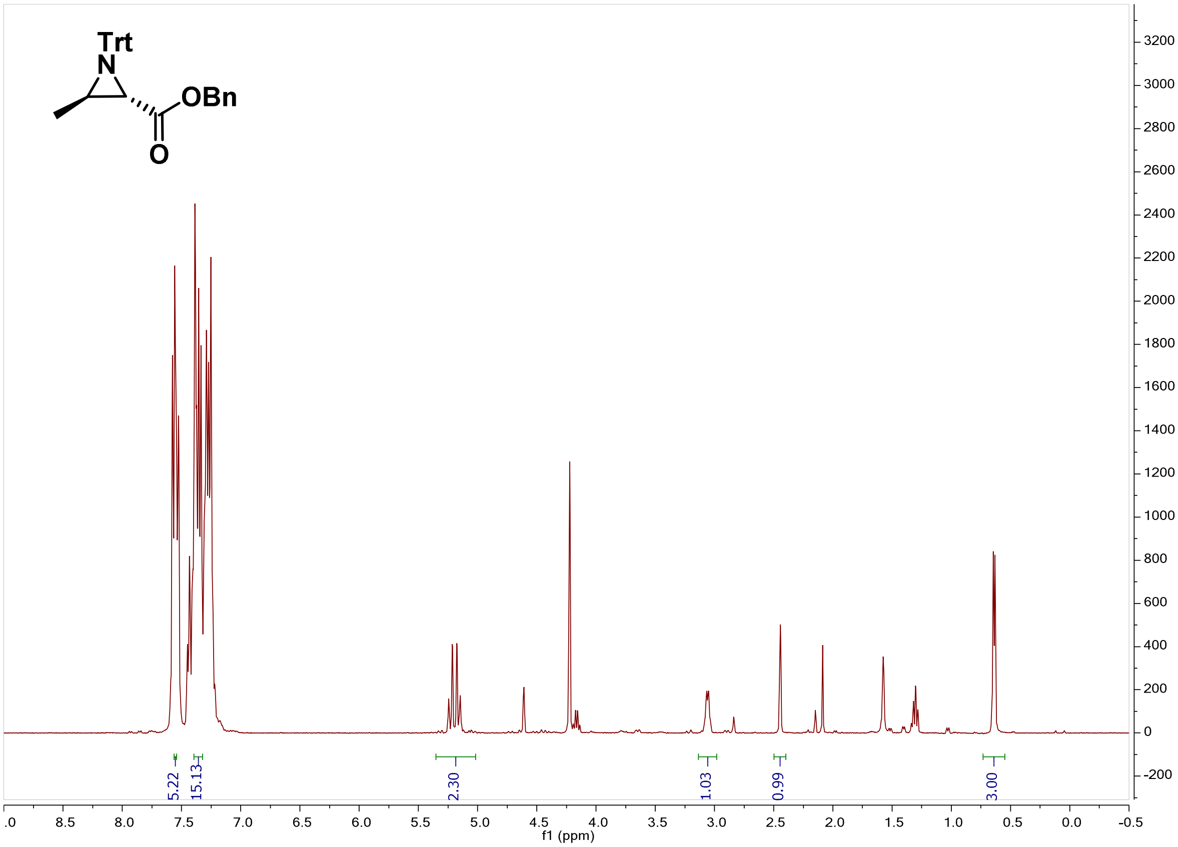
Trt
N
OBn
O
5.22
15.13
2.30
1.03
0.99
3.00
f1 (ppm)

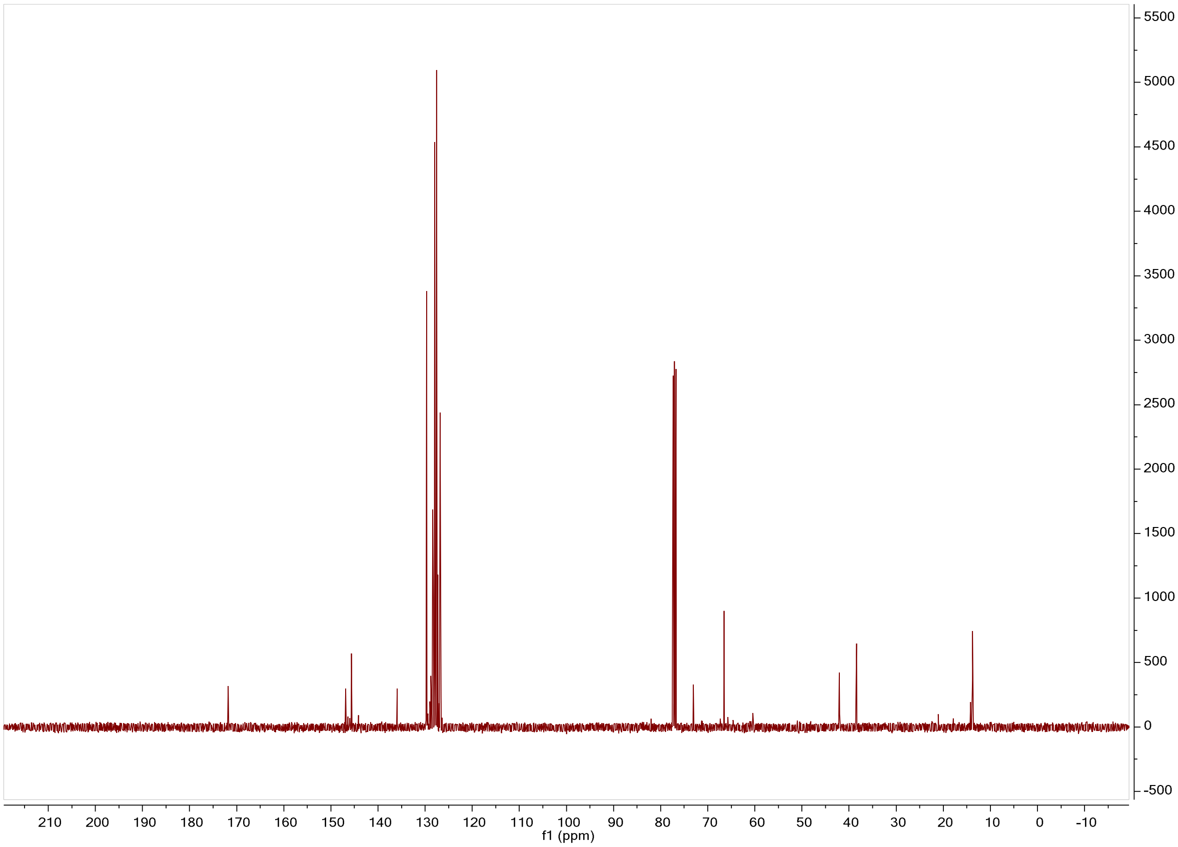
f1 (ppm)

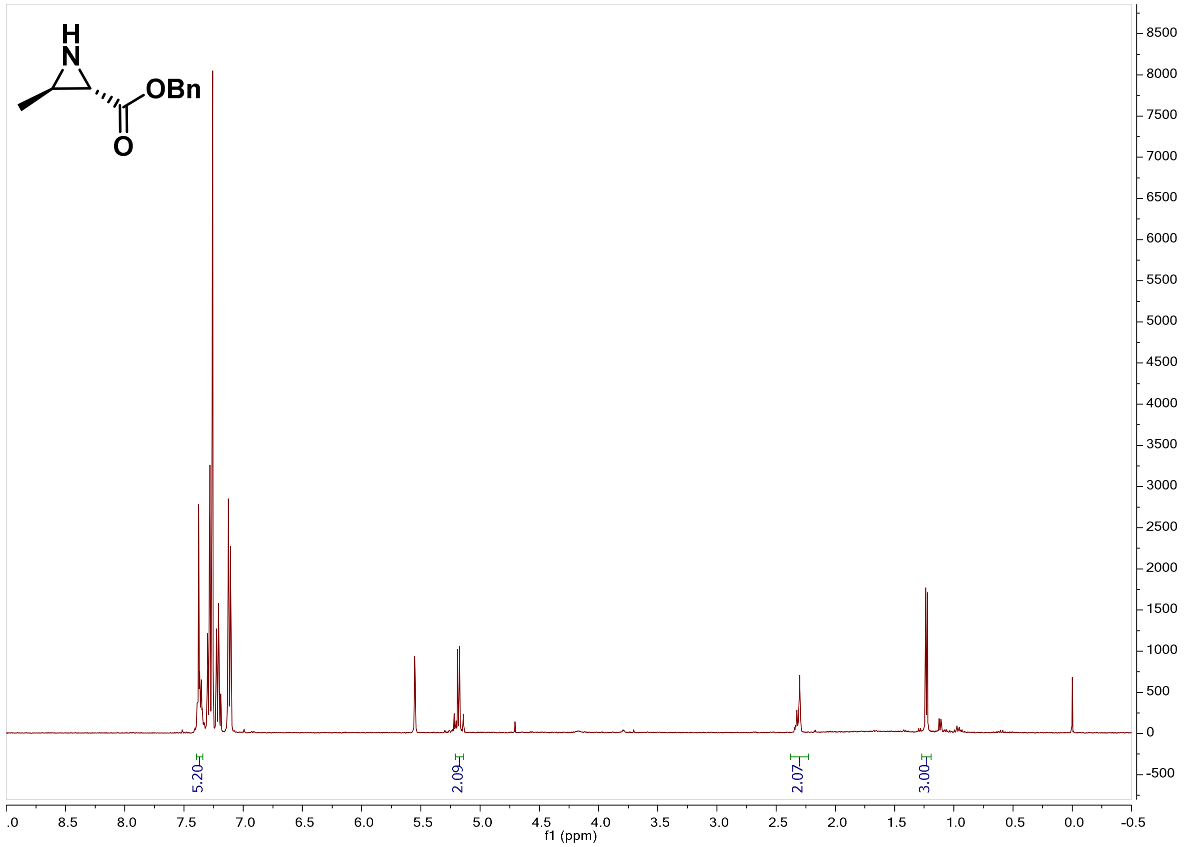
H
N
OBn
O
5.20
2.09
2.07
3.00
f1 (ppm)

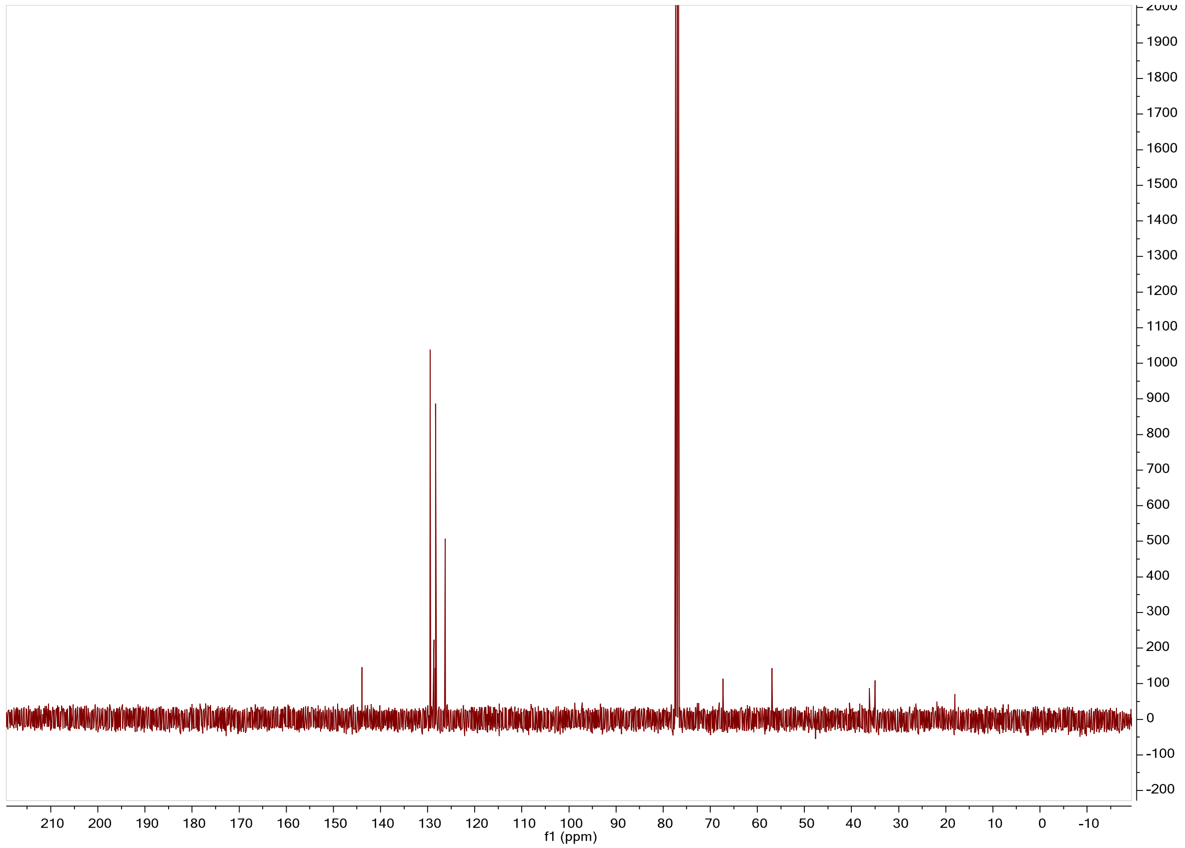
f1 (ppm)

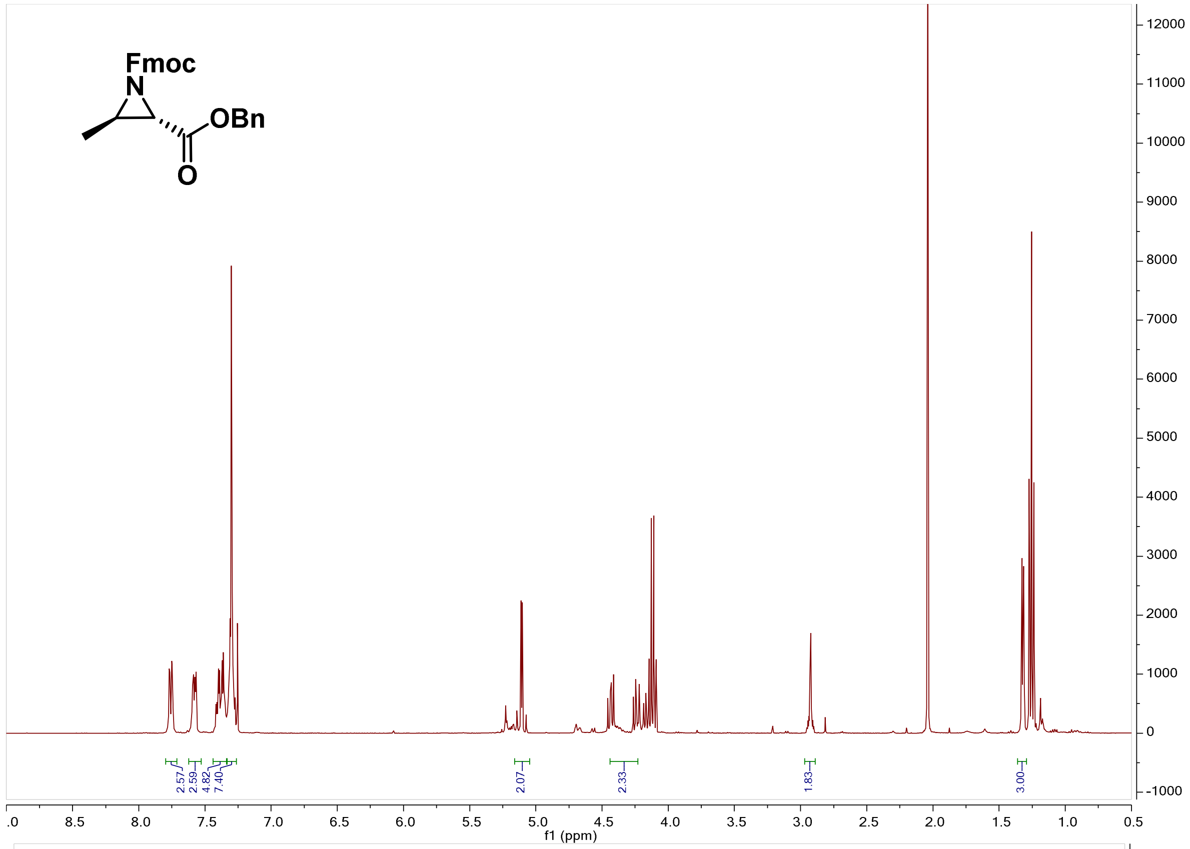
Fmoc
N
OBn
O
2.57
2.59
4.82
7.40
2.07
2.33
1.83
3.00
f1 (ppm)

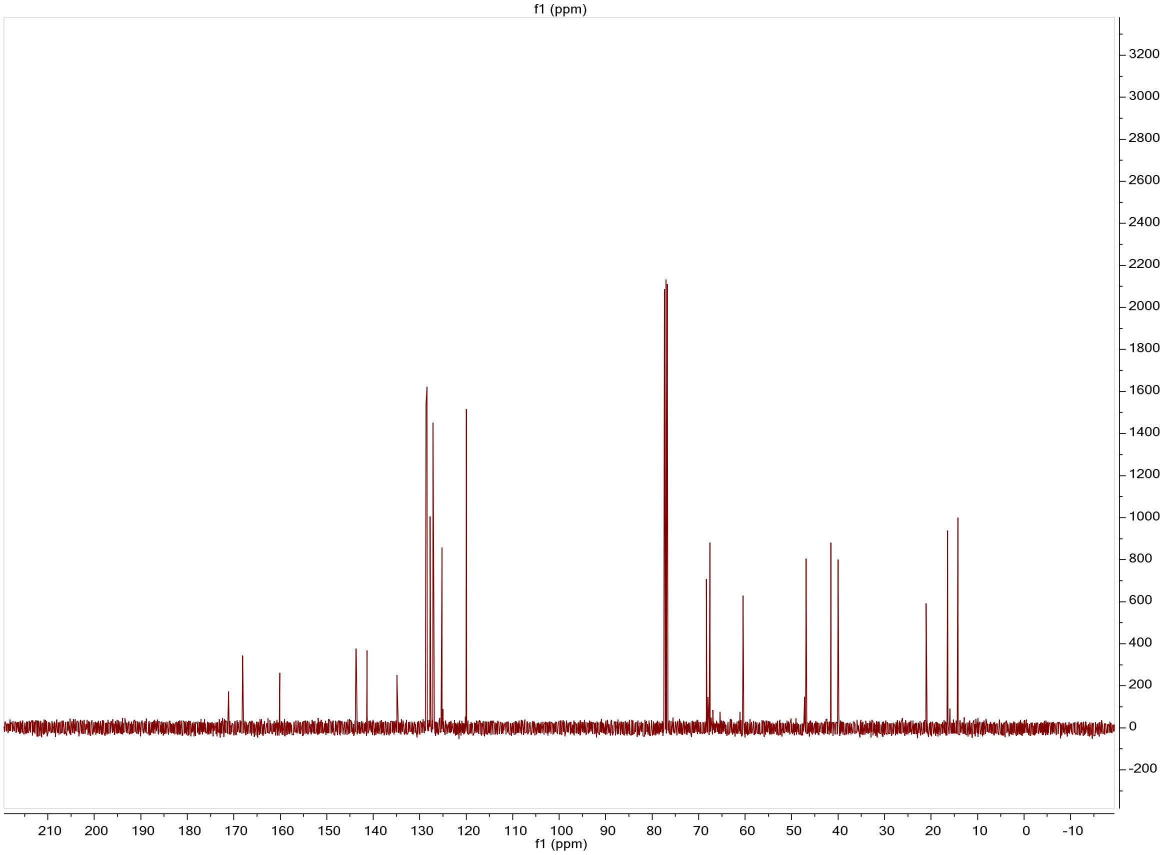
f1 (ppm)

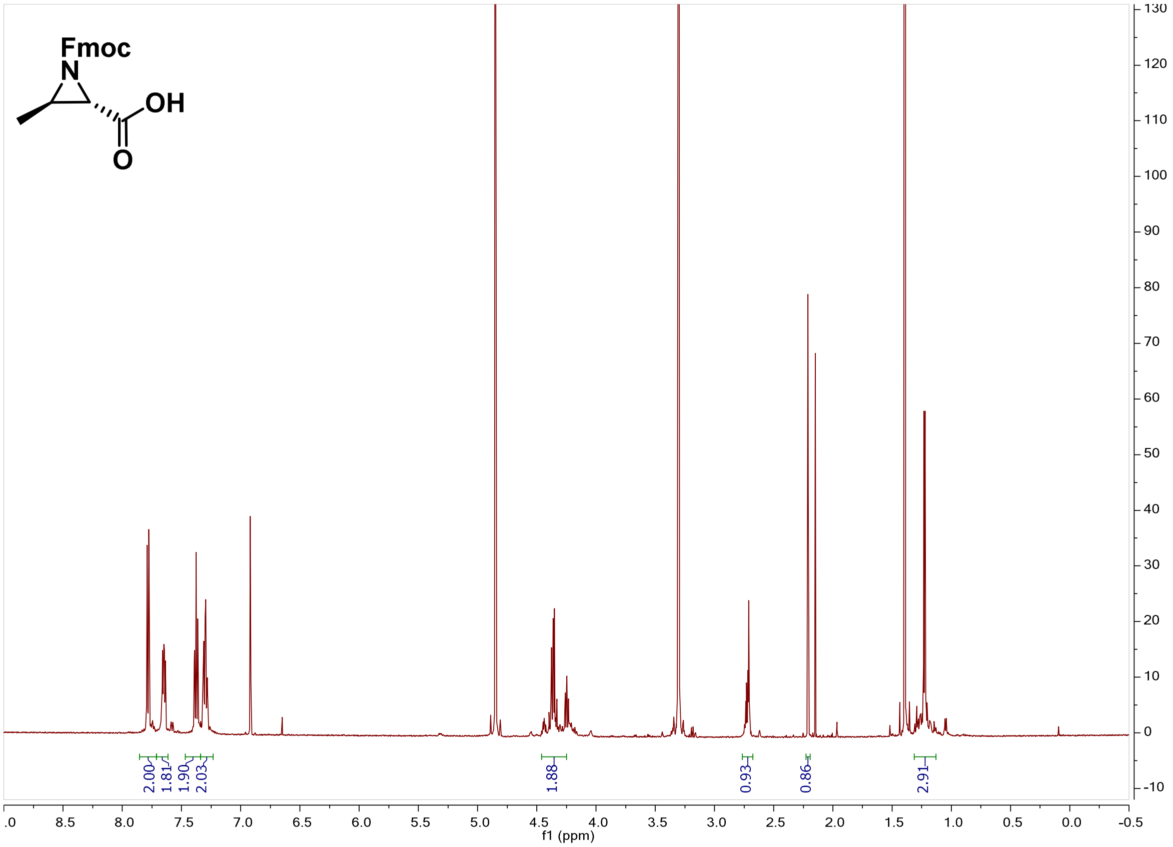
Fmoc
N
OH
O
2.00
1.81
1.90
2.03
1.88
0.93
0.86
2.91
f1 (ppm)

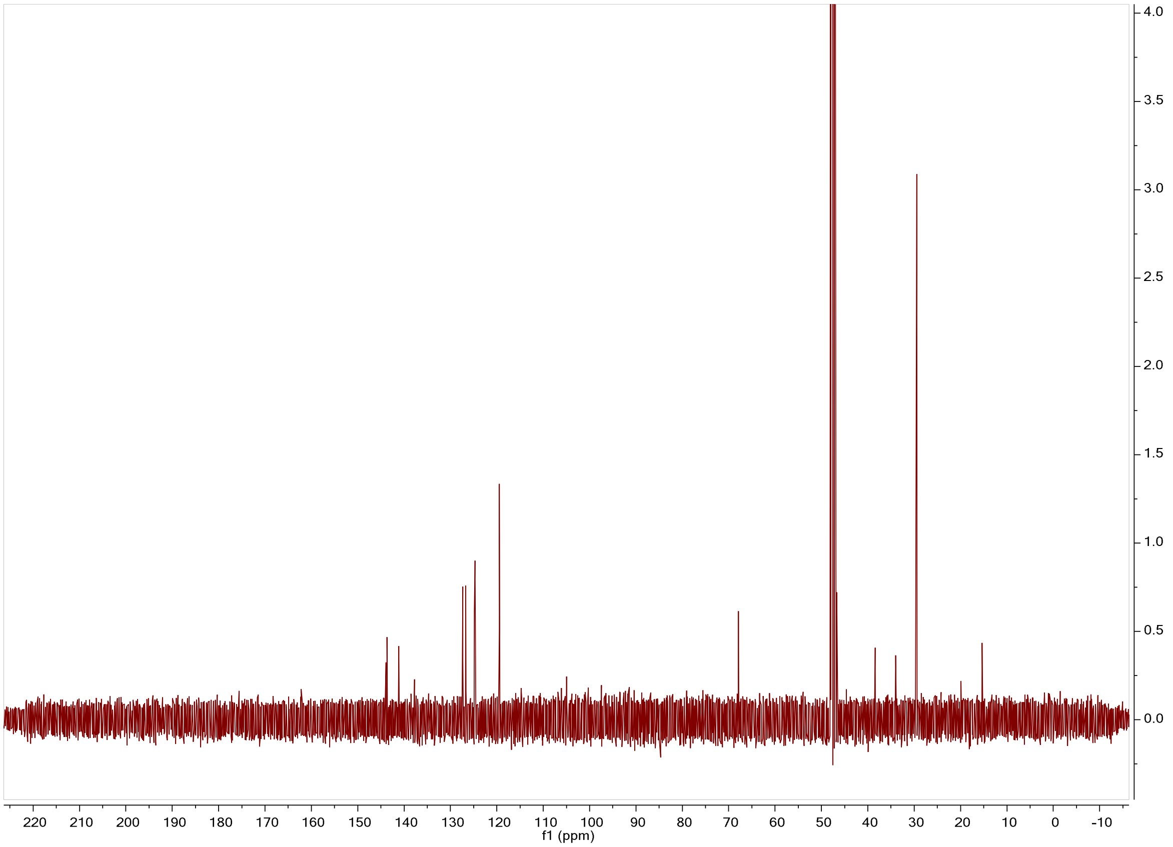
f1 (ppm)

20121213_mercury_400_CW05135-MeOH-PROTON_01

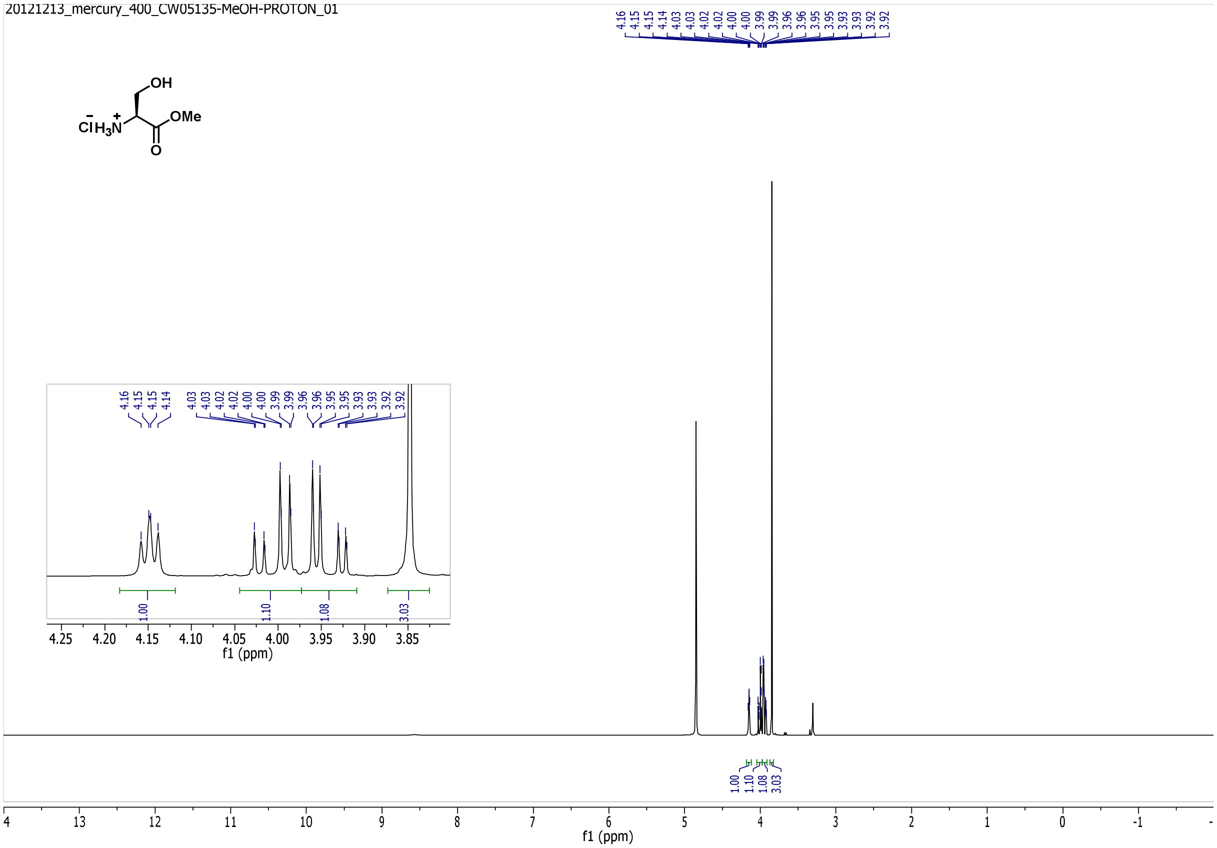

20121216_mercury_400_CW05135-MeOHC-CARBON_01

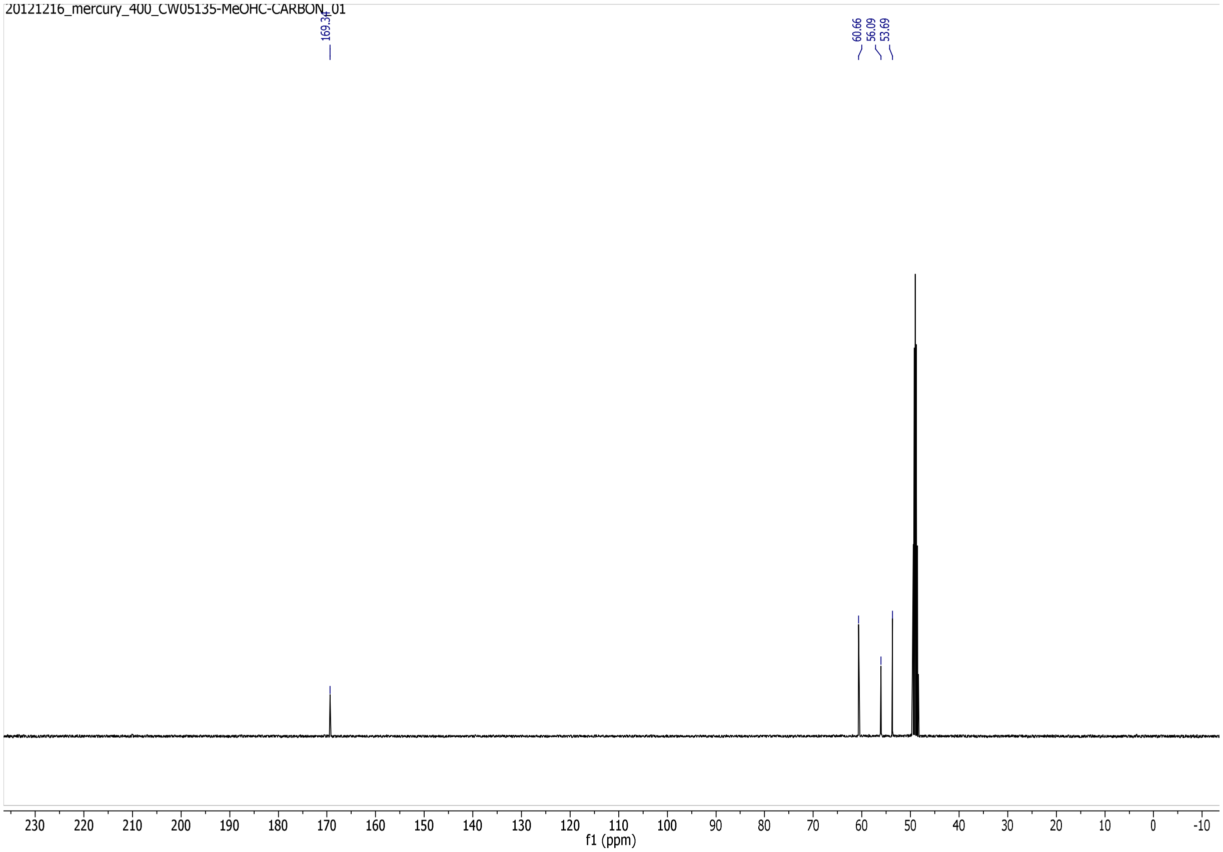

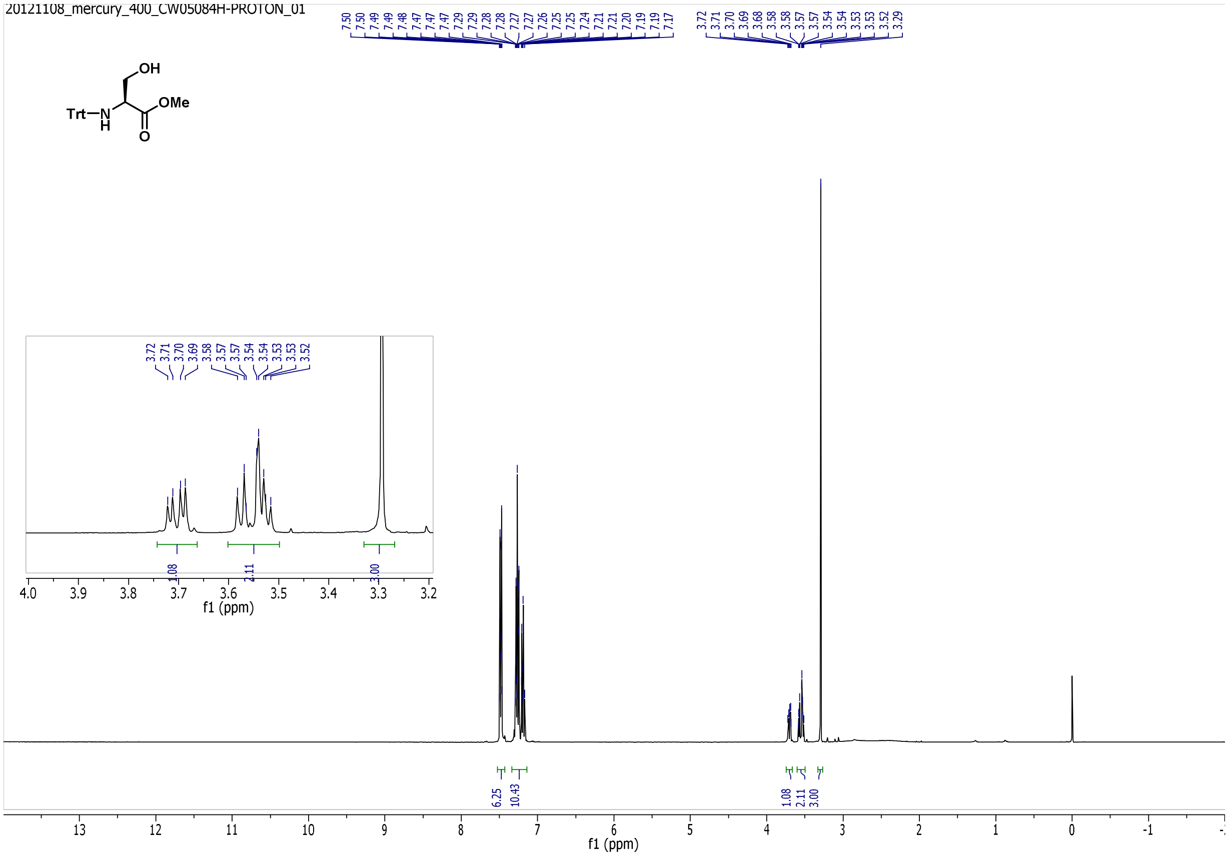

20121108_mercury_400_CW05084H-PROTON_01
7.50 7.50 7.49 7.49 7.48 7.47 7.47 7.47 7.29 7.29 7.28 7.28 7.27 7.27 7.26 7.25 7.25 7.24 7.21 7.21 7.20 7.19 7.19 7.17
3.72 3.71 3.70 3.69 3.68 3.58 3.58 3.57 3.57 3.54 3.54 3.53 3.53 3.52 3.29
OH
OMe
Trt
N
H
O
3.72 3.71 3.70 3.69 3.58 3.57 3.57 3.54 3.54 3.53 3.53 3.52
1.08
2.11
3.00
6.25
10.43
f1 (ppm)

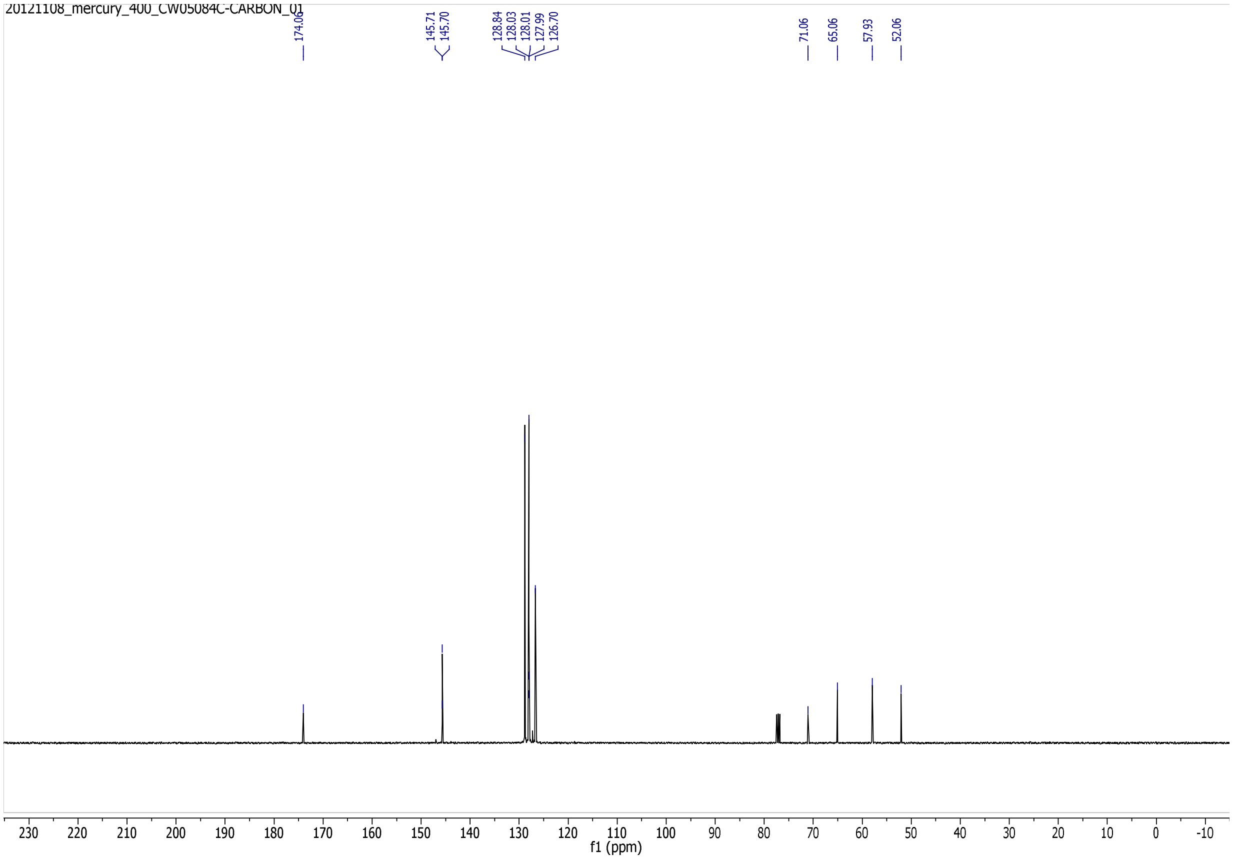

20121108_mercury_400_CW05084C-CARBON_01
174.06
145.71 145.70
128.84 128.03 128.01 127.99 126.70
71.06
65.06
57.93
52.06
f1 (ppm)

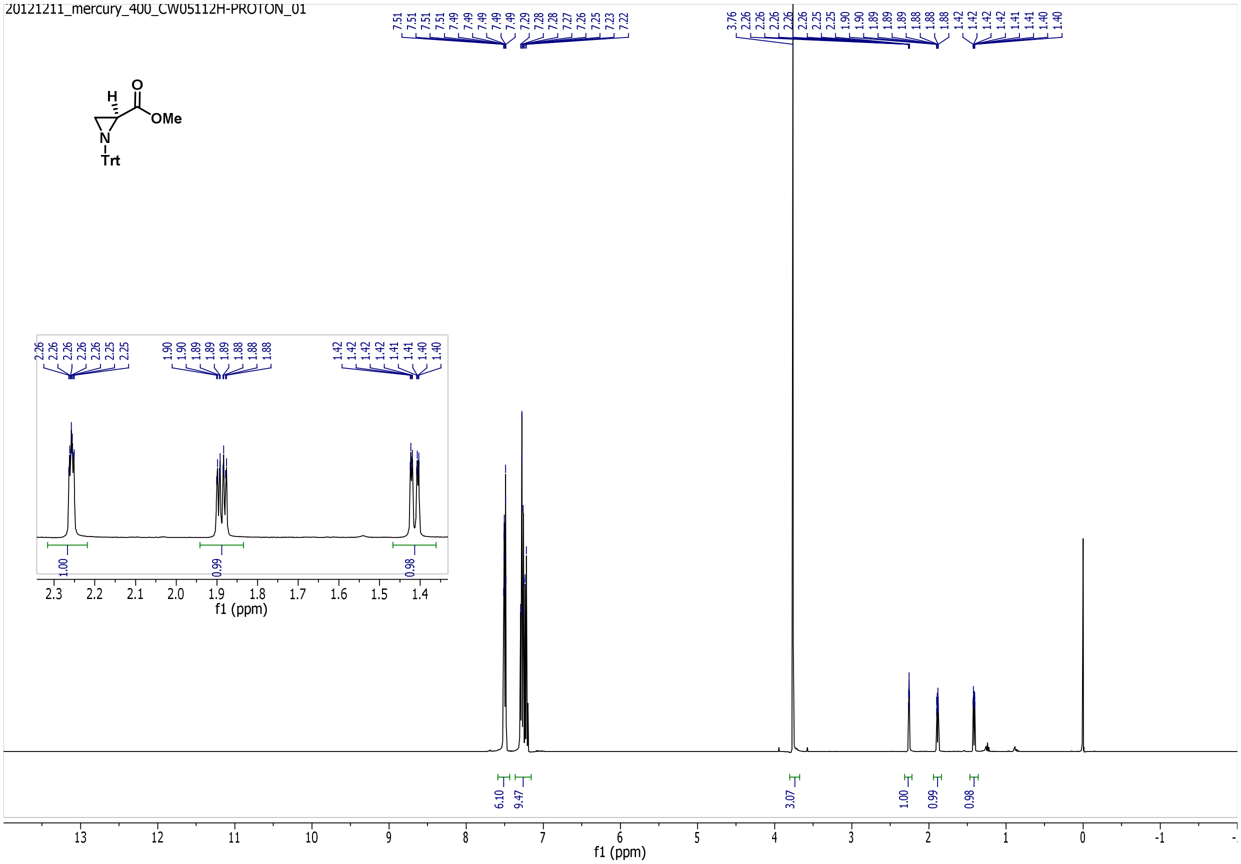
20121211_mercury_400_CW05112H-PROTON_01
OMe
Trt
f1 (ppm)

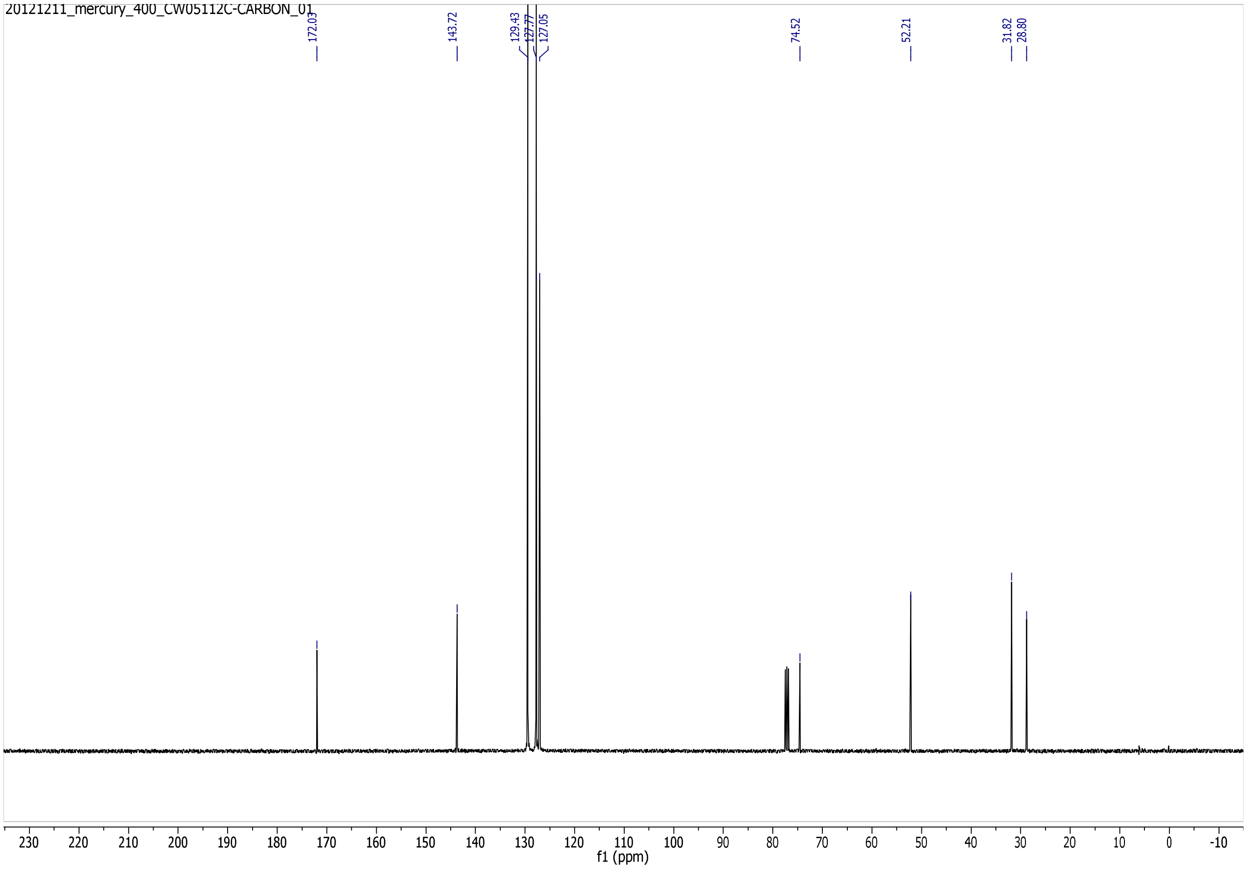
20121211_mercury_400_CW05112C-CARBON_01
172.03
143.72
129.43
127.77
127.05
74.52
52.21
31.82
28.80
f1 (ppm)

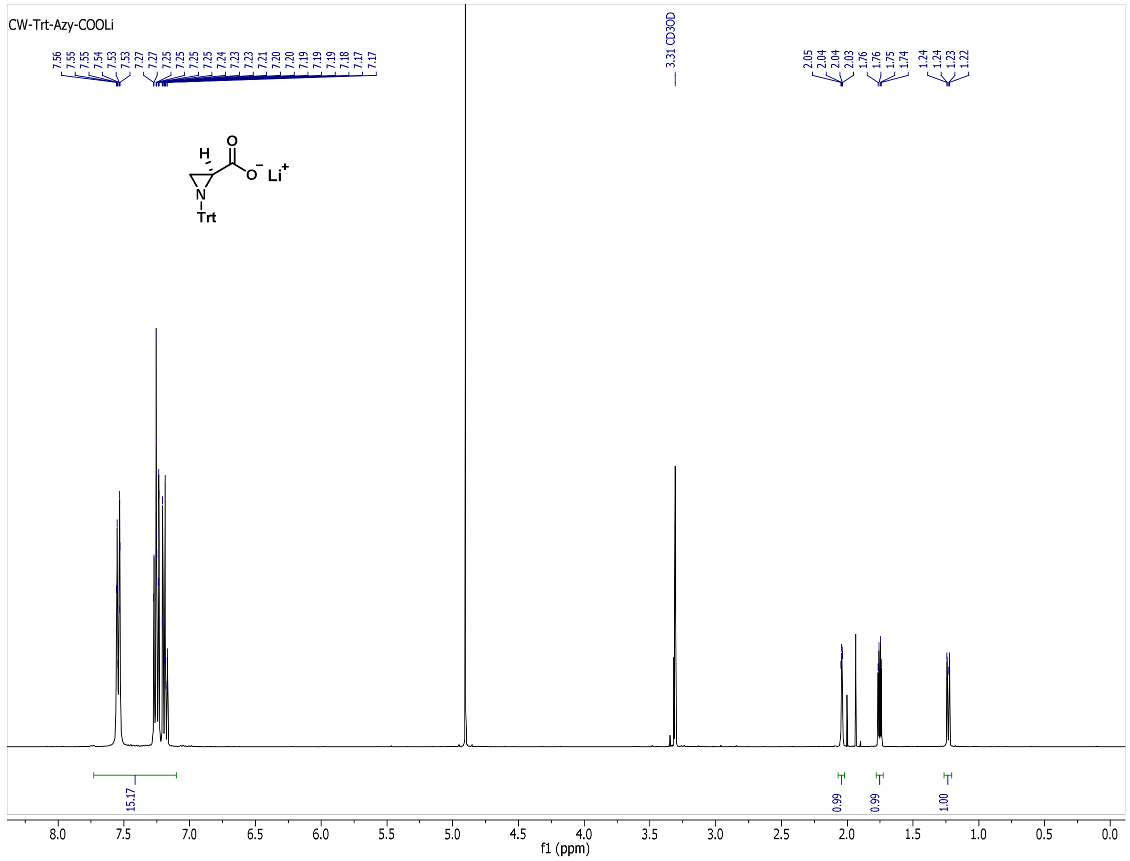
CW-Trt-Azy-COOLi
7.56 7.55 7.55 7.54 7.53 7.53 7.27 7.27 7.25 7.25 7.25 7.25 7.24 7.23 7.23 7.21 7.20 7.20 7.19 7.19 7.18 7.17 7.17
3.31 CD3OD
2.05 2.04 2.04 2.03 1.76 1.76 1.75 1.74 1.24 1.24 1.23 1.22
H
O
O
Li+
N
Trt
15.17
0.99
0.99
1.00
f1 (ppm)

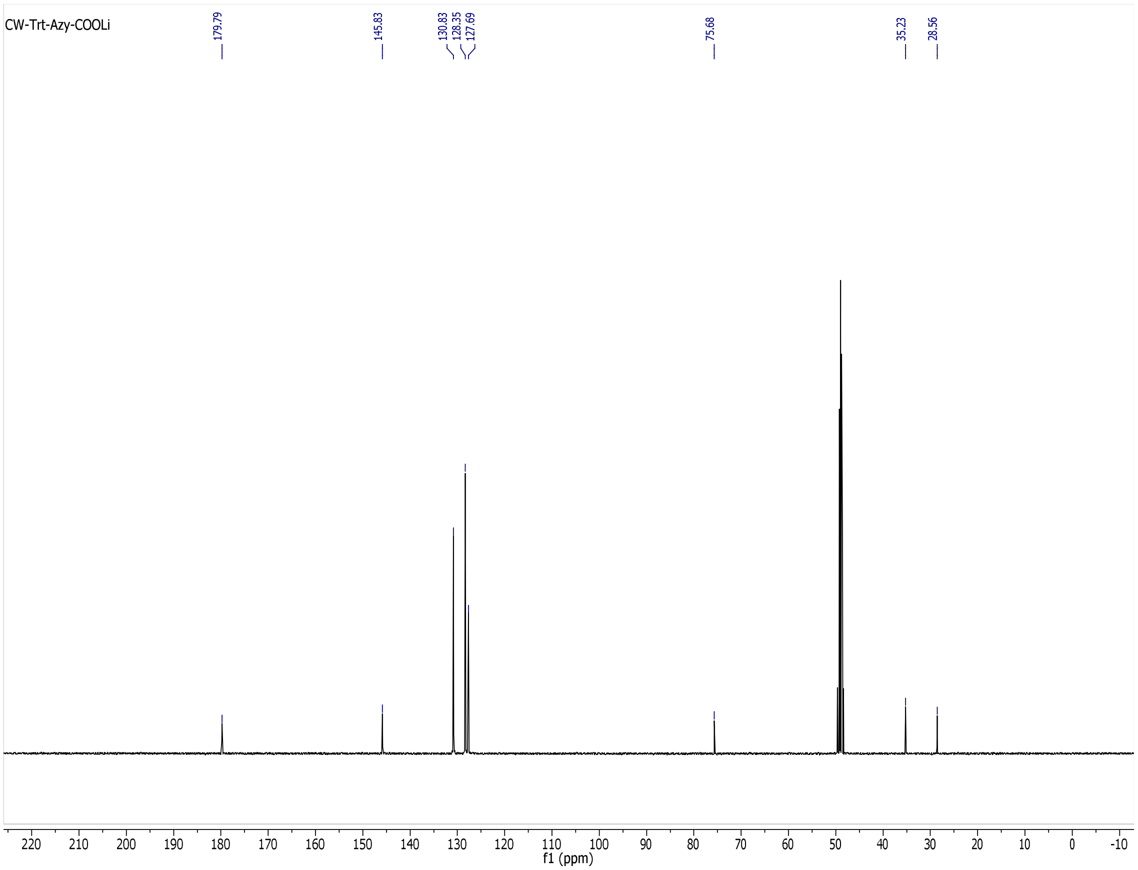
CW-Trt-Azy-COOLi
179.79
145.83
130.83 128.35 127.69
75.68
35.23
28.56
f1 (ppm)

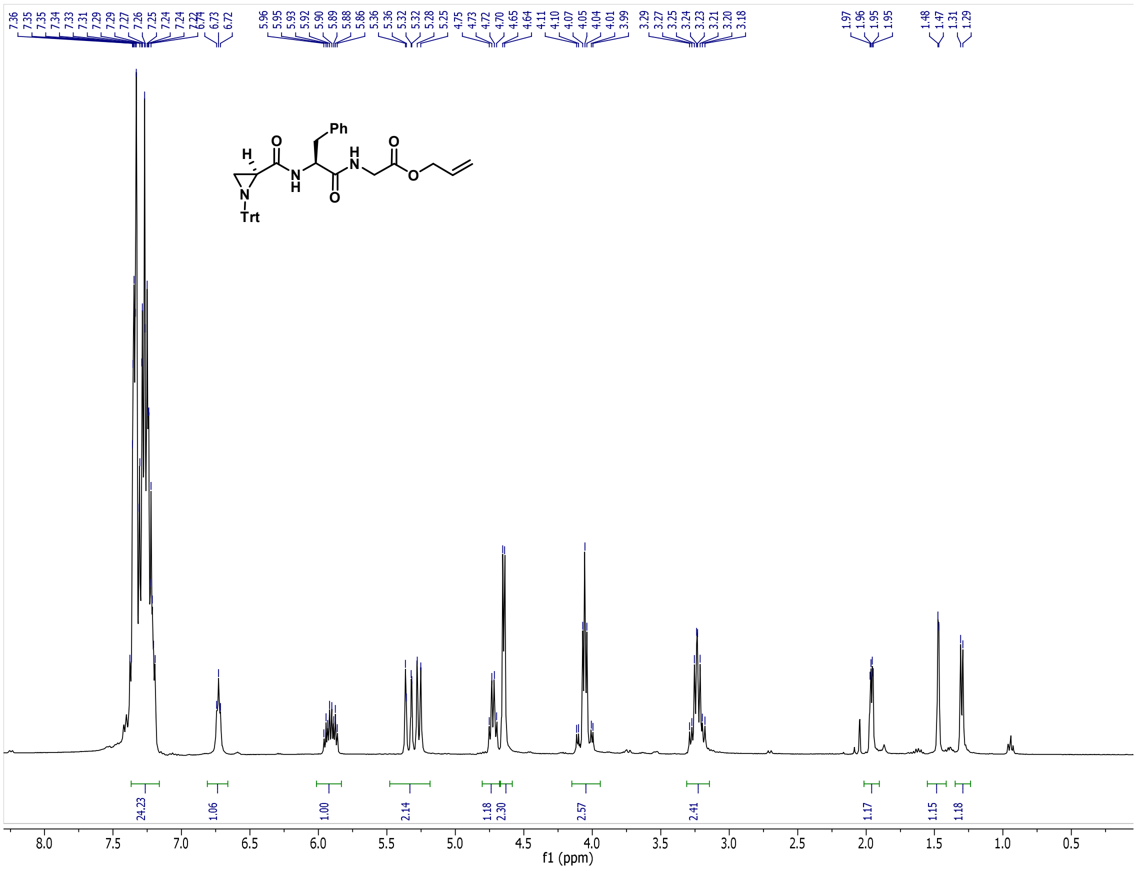
Ph
H
N
H
O
O
N
H
O
O
N
Trt
f1 (ppm)

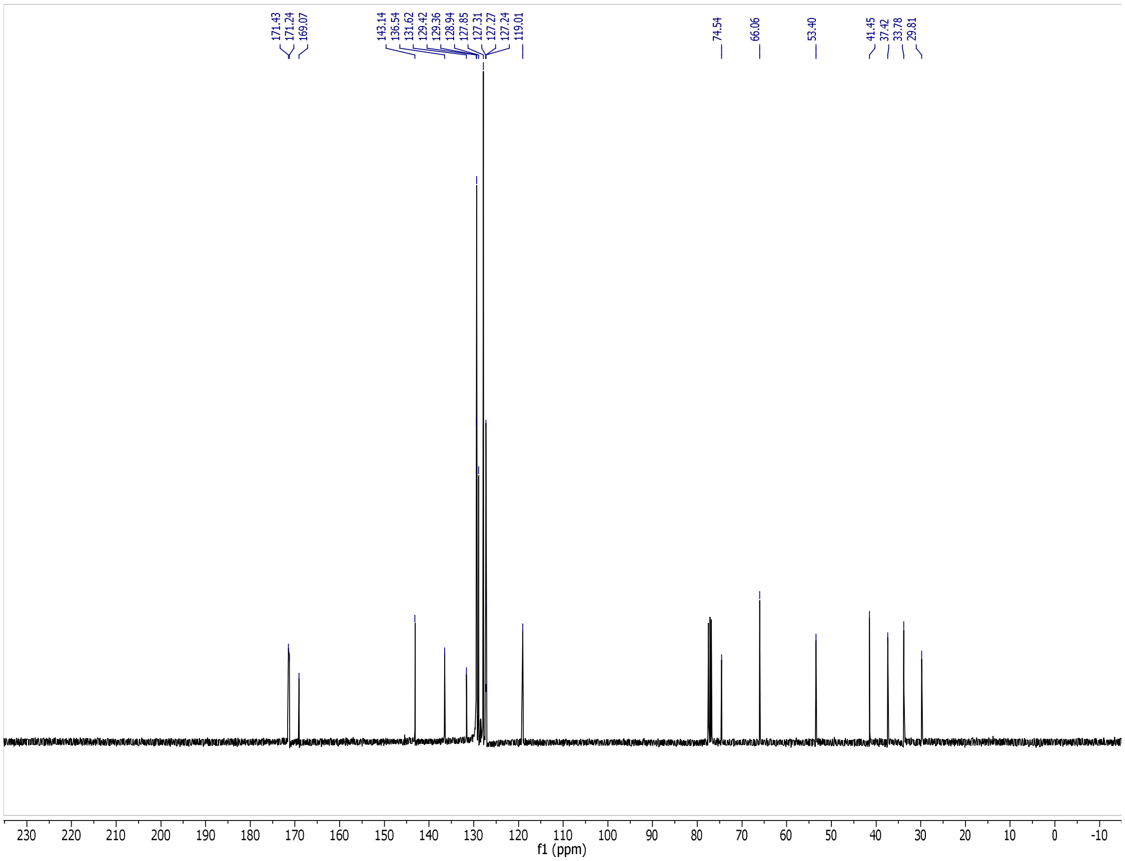
171.43
171.24
169.07
143.14
136.54
131.62
129.42
129.36
128.94
127.85
127.31
127.27
127.24
119.01
74.54
66.06
53.40
41.45
37.42
33.78
29.81
f1 (ppm)

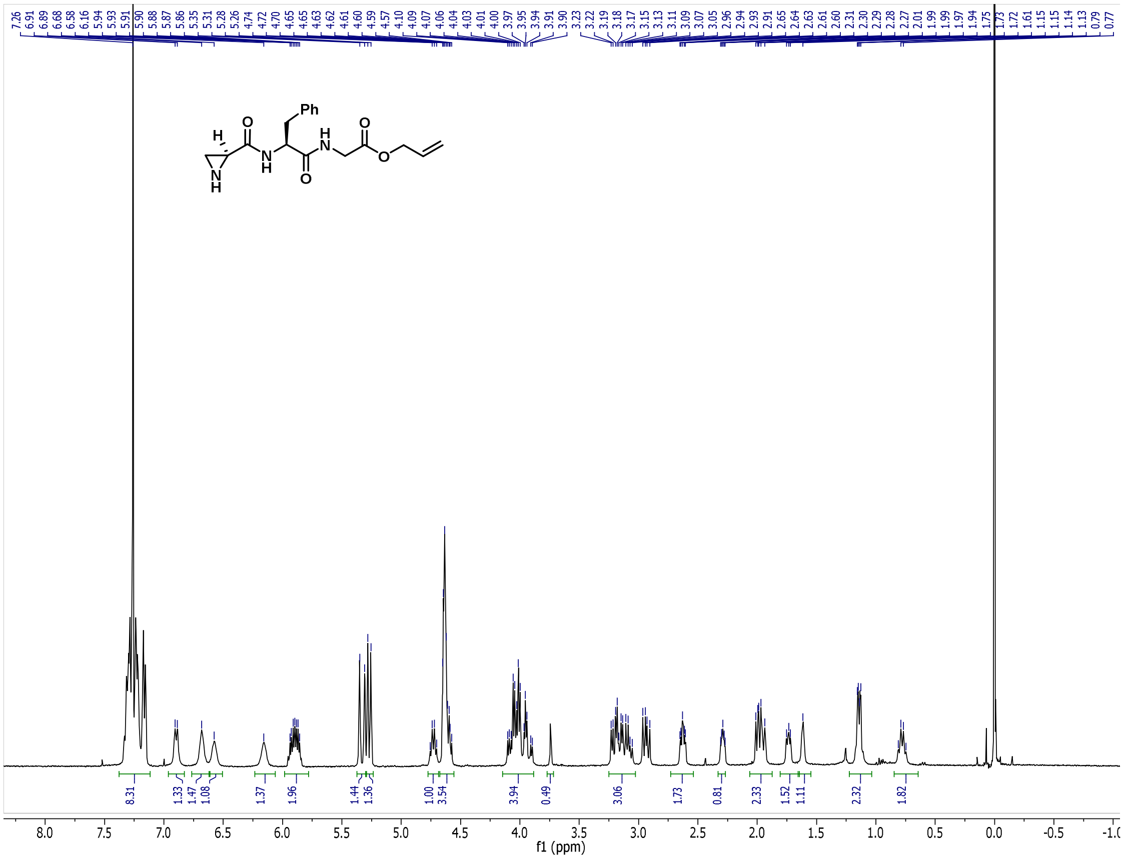
7.26 6.91 6.89 6.68 6.58 6.16 5.94 5.93 5.91 5.90 5.88 5.87 5.86 5.35 5.31 5.28 5.26 4.74 4.72 4.70 4.65 4.65 4.63 4.62 4.61 4.60 4.59 4.57 4.10 4.09 4.07 4.06 4.04 4.03 4.01 4.00 3.97 3.95 3.94 3.91 3.90 3.23 3.22 3.19 3.18 3.17 3.15 3.13 3.11 3.09 3.07 3.05 2.96 2.94 2.93 2.91 2.65 2.64 2.63 2.61 2.60 2.31 2.30 2.29 2.28 2.27 2.01 1.99 1.99 1.97 1.94 1.75 1.73 1.72 1.61 1.15 1.15 1.14 1.13 0.79 0.77
Ph
8.31 1.33 1.47 1.08 1.37 1.96 1.44 1.36 1.00 3.54 3.94 0.49 3.06 1.73 0.81 2.33 1.52 1.11 2.32 1.82
f1 (ppm)

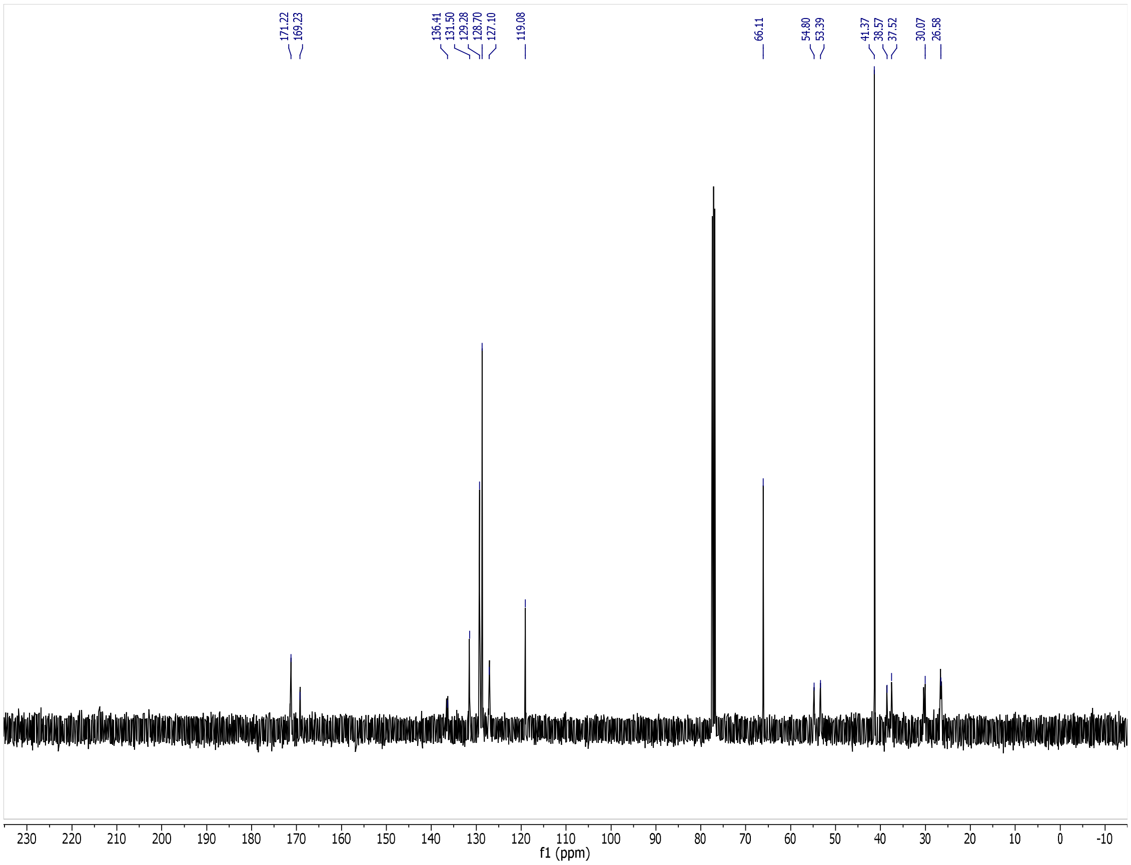
171.22 169.23 136.41 131.50 129.28 128.70 127.10 119.08 66.11 54.80 53.39 41.37 38.57 37.52 30.07 26.58
f1 (ppm)

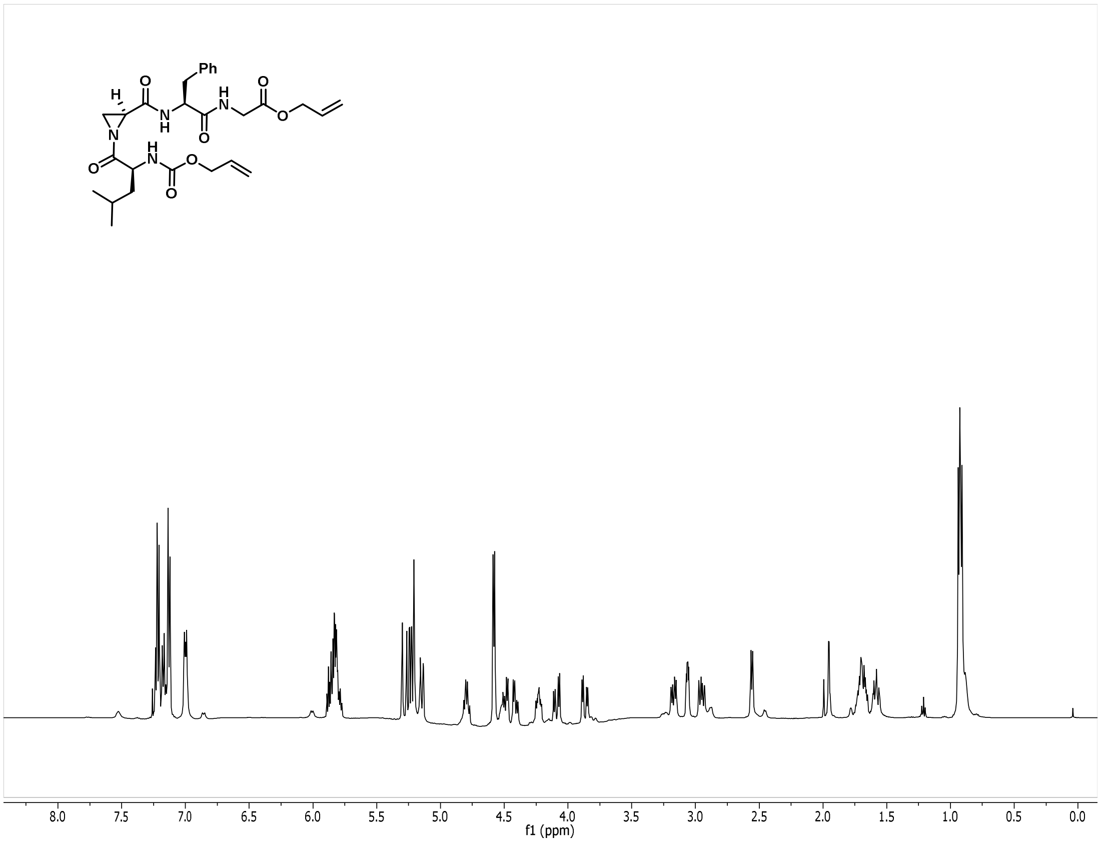
Ph
f1 (ppm)

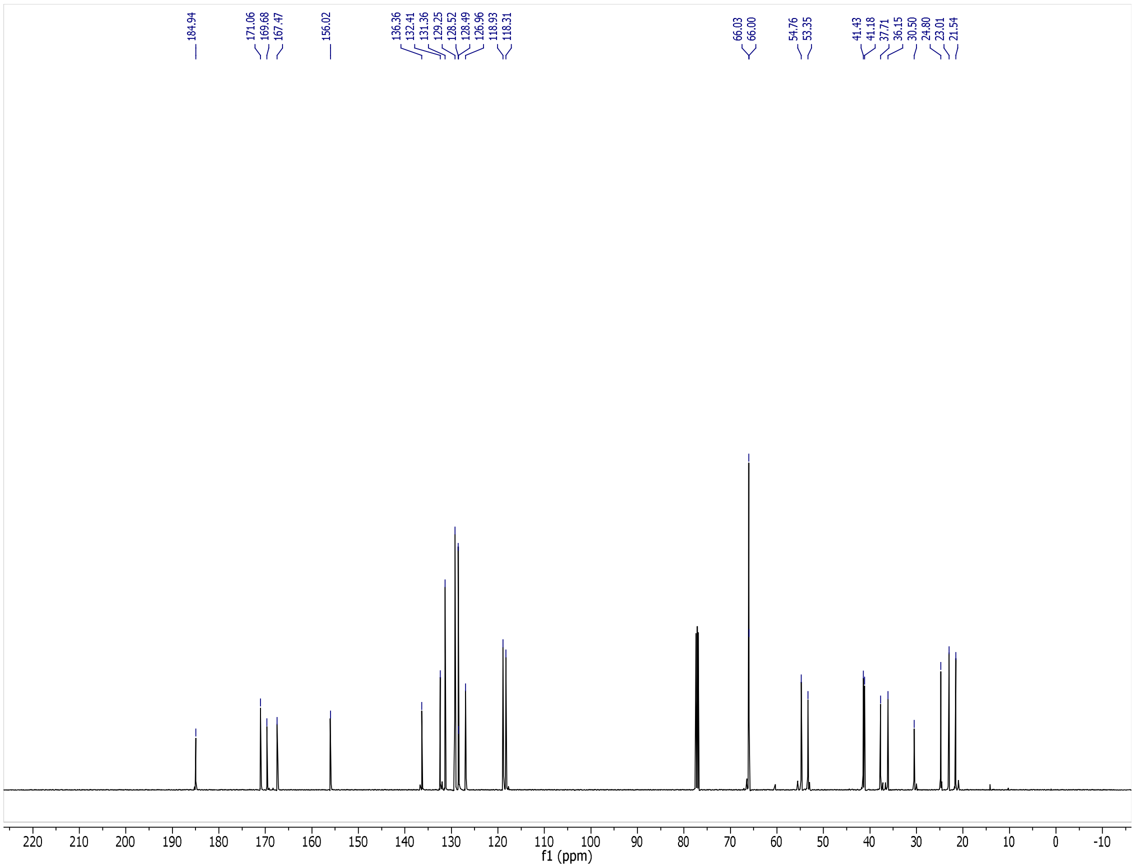
184.94
171.06
169.68
167.47
156.02
136.36
132.41
131.36
129.25
128.52
128.49
126.96
118.93
118.31
66.03
66.00
54.76
53.35
41.43
41.18
37.71
36.15
30.50
24.80
23.01
21.54
f1 (ppm)

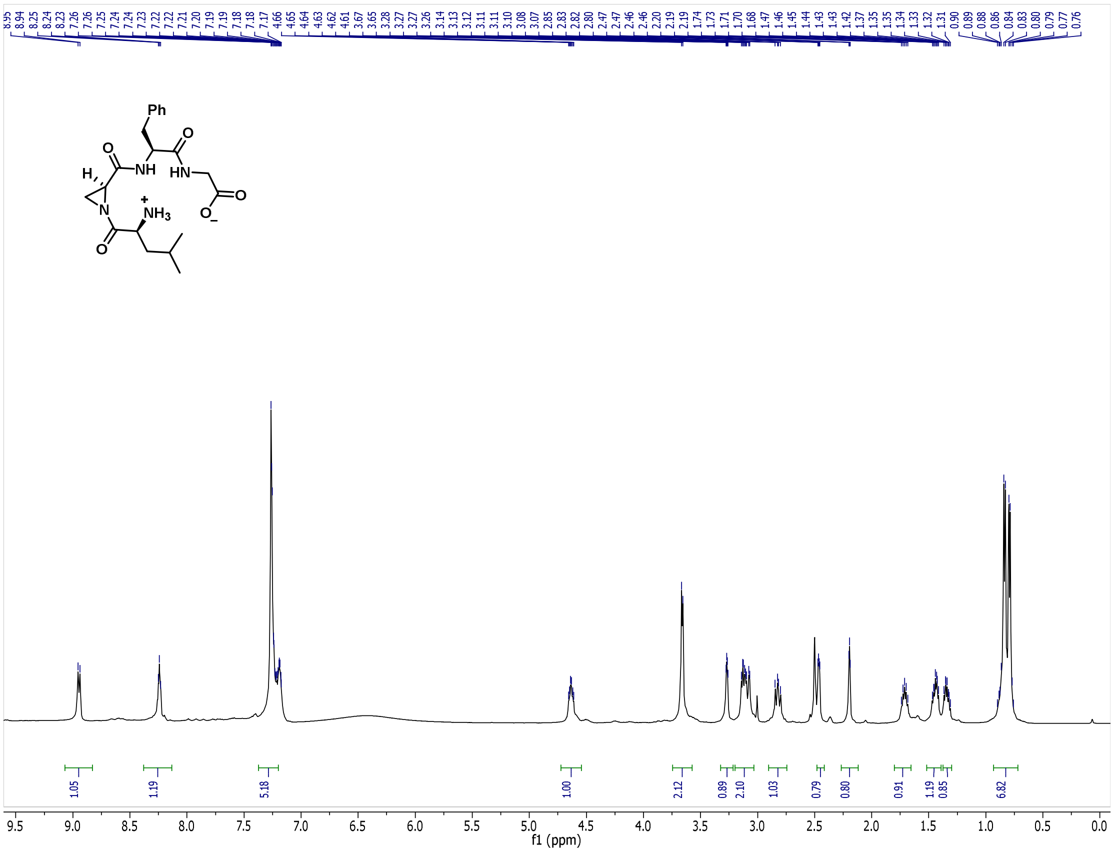

8.95 8.94 8.25 8.24 8.23 7.26 7.25 7.24 7.24 7.23 7.22 7.22 7.21 7.20 7.19 7.19 7.18 7.17 4.66 4.65 4.64 4.63 4.62 4.61 3.67 3.65 3.28 3.27 3.26 3.14 3.13 3.12 3.11 3.11 3.10 3.08 3.07 2.85 2.83 2.82 2.80 2.47 2.47 2.46 2.46 2.20 2.19 2.19 1.74 1.73 1.71 1.70 1.68 1.47 1.46 1.45 1.44 1.43 1.43 1.42 1.37 1.35 1.35 1.34 1.33 1.32 1.31 0.90 0.89 0.88 0.86 0.84 0.83 0.80 0.79 0.77 0.76
Ph
O
O
H
NH
HN
N
NH_3^+
O
O
O⁻
1.05
1.19
5.18
1.00
2.12
0.89
2.10
1.03
0.79
0.80
0.91
1.19
0.85
6.82
9.5 9.0 8.5 8.0 7.5 7.0 6.5 6.0 5.5 5.0 4.5 4.0 3.5 3.0 2.5 2.0 1.5 1.0 0.5 0.0
f1 (ppm)

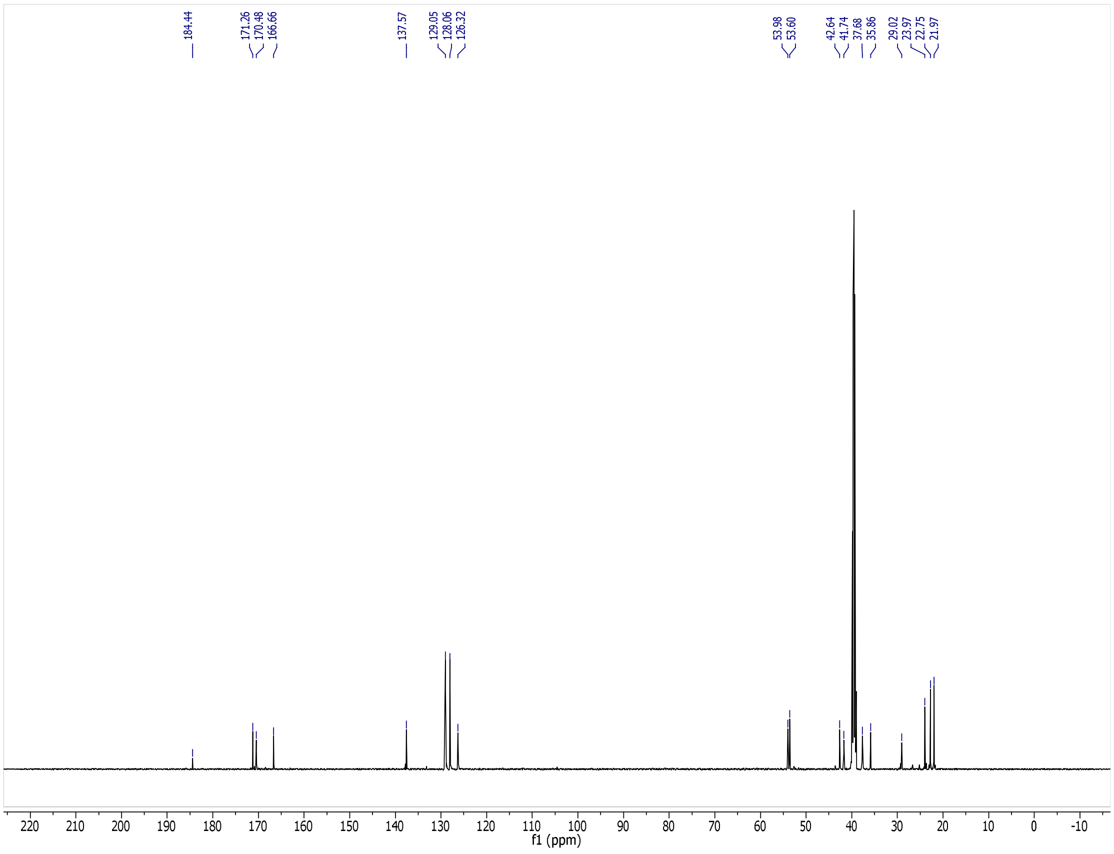

184.44
171.26 170.48 166.66
137.57
129.05 128.06 126.32
53.98 53.60
42.64 41.74 37.68 35.86
29.02 23.97 22.75 21.97
220 210 200 190 180 170 160 150 140 130 120 110 100 90 80 70 60 50 40 30 20 10 0 -10
f1 (ppm)

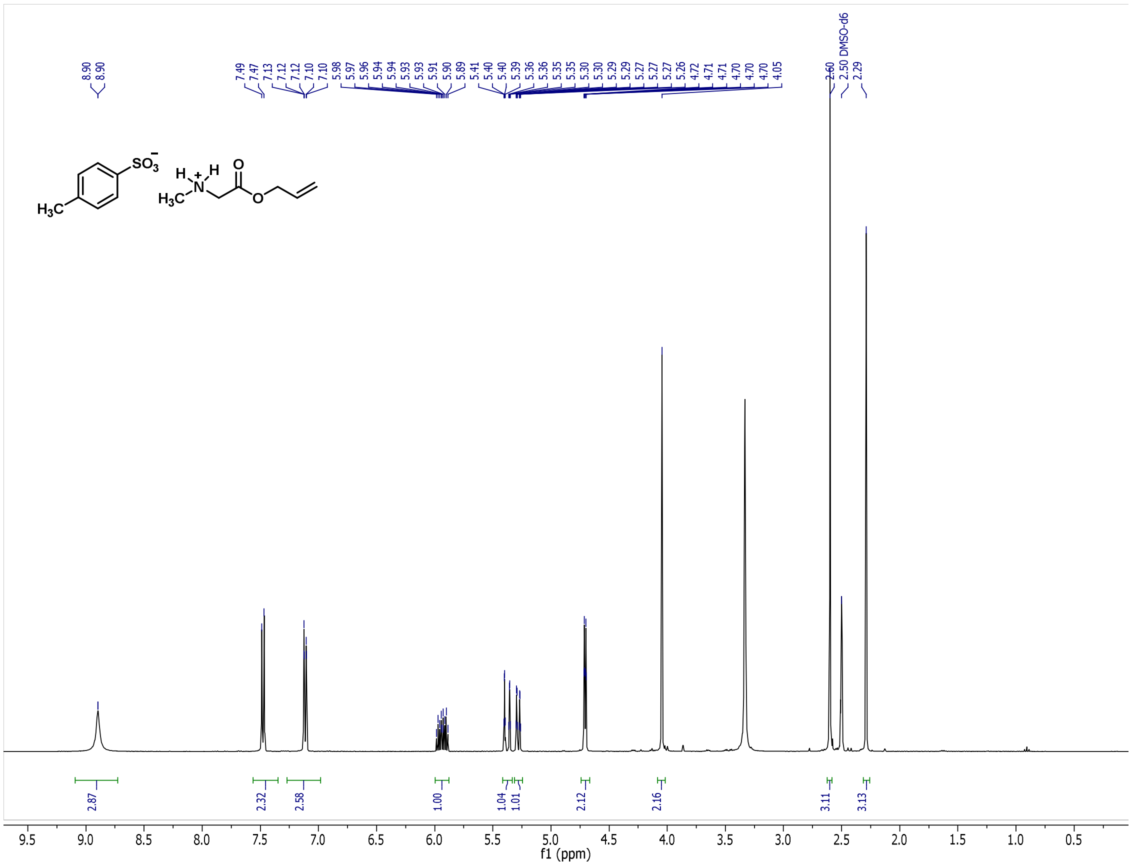
8.90
8.90
7.49
7.47
7.13
7.12
7.12
7.10
7.10
5.98
5.97
5.96
5.94
5.94
5.93
5.93
5.91
5.90
5.89
5.41
5.40
5.40
5.39
5.36
5.36
5.35
5.35
5.30
5.30
5.29
5.29
5.27
5.27
5.27
5.26
4.72
4.71
4.71
4.70
4.70
4.70
4.05
2.60
2.50 DMSO-d6
2.29
2.87
2.32
2.58
1.00
1.04
1.01
2.12
2.16
3.11
3.13
f1 (ppm)

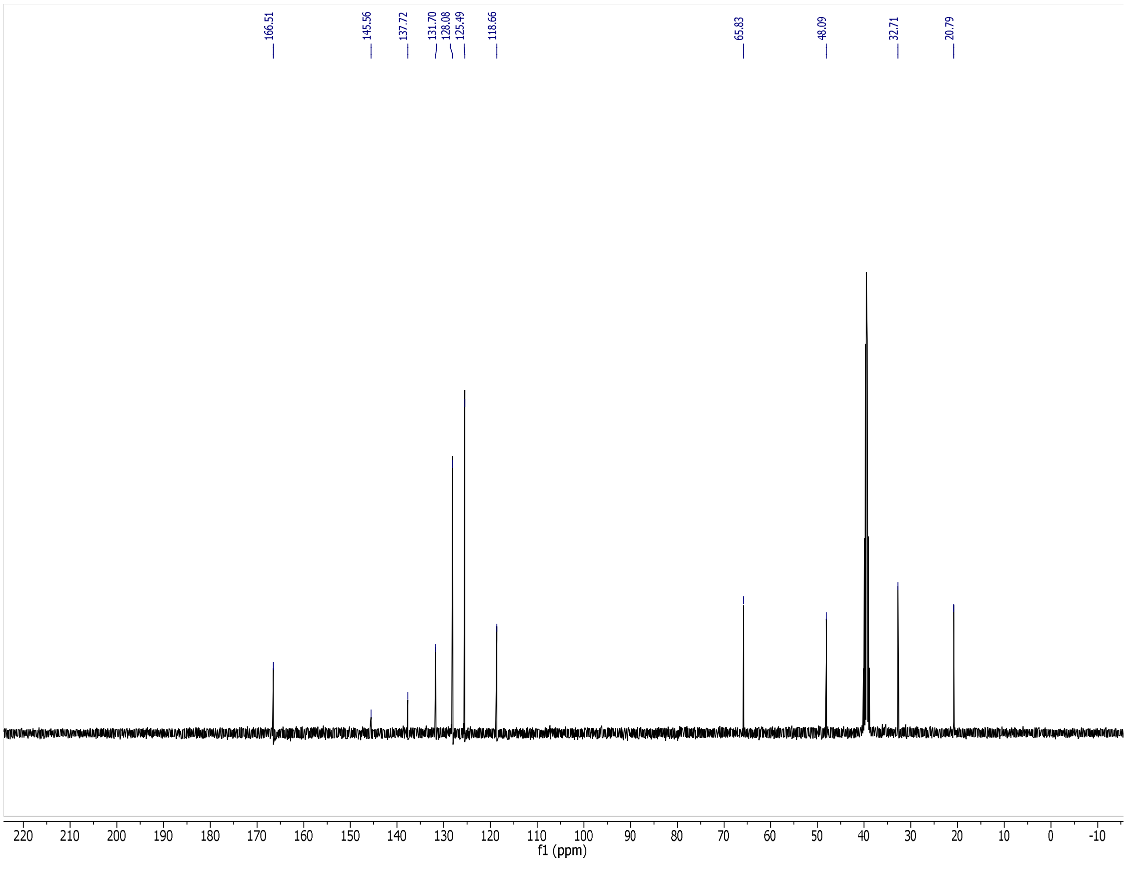
166.51
145.56
137.72
131.70
128.08
125.49
118.66
65.83
48.09
32.71
20.79
f1 (ppm)

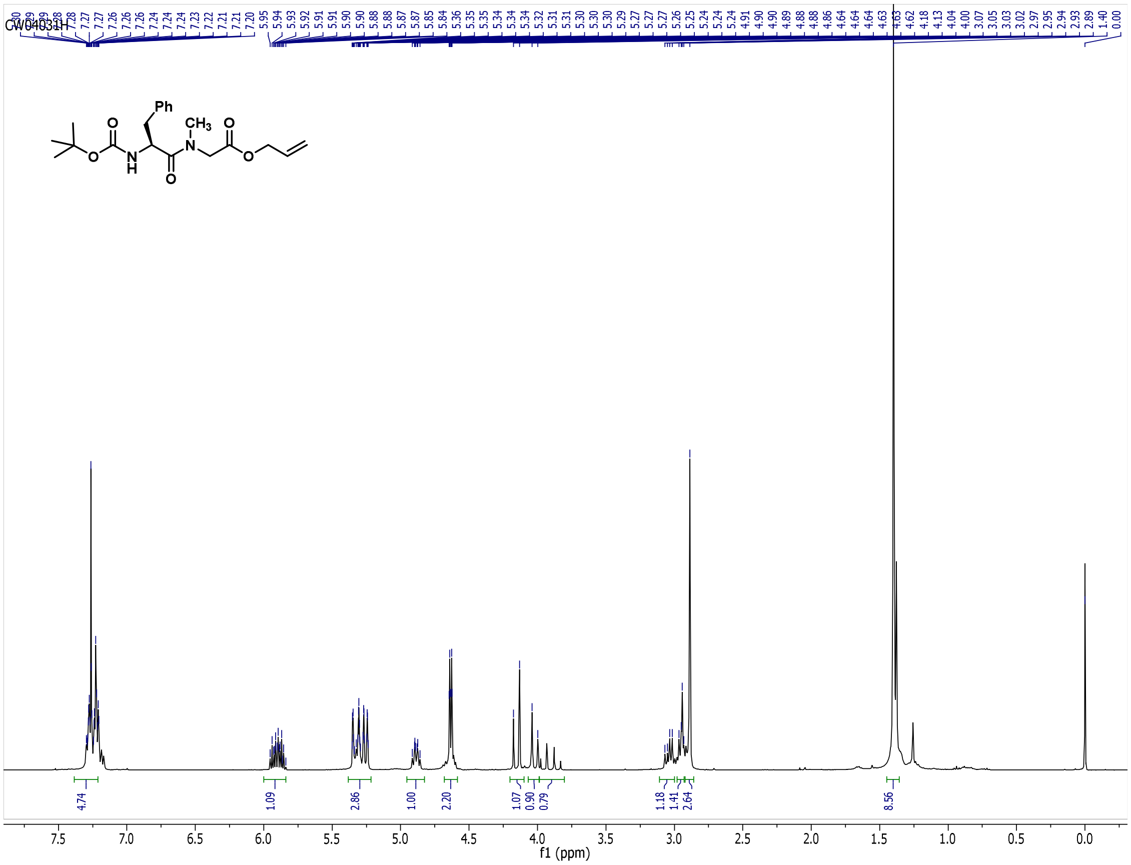
CW04031H
Ph
CH_3
4.74
1.09
2.86
1.00
2.20
1.07
0.90
0.79
1.18
1.41
2.64
8.56
f1 (ppm)

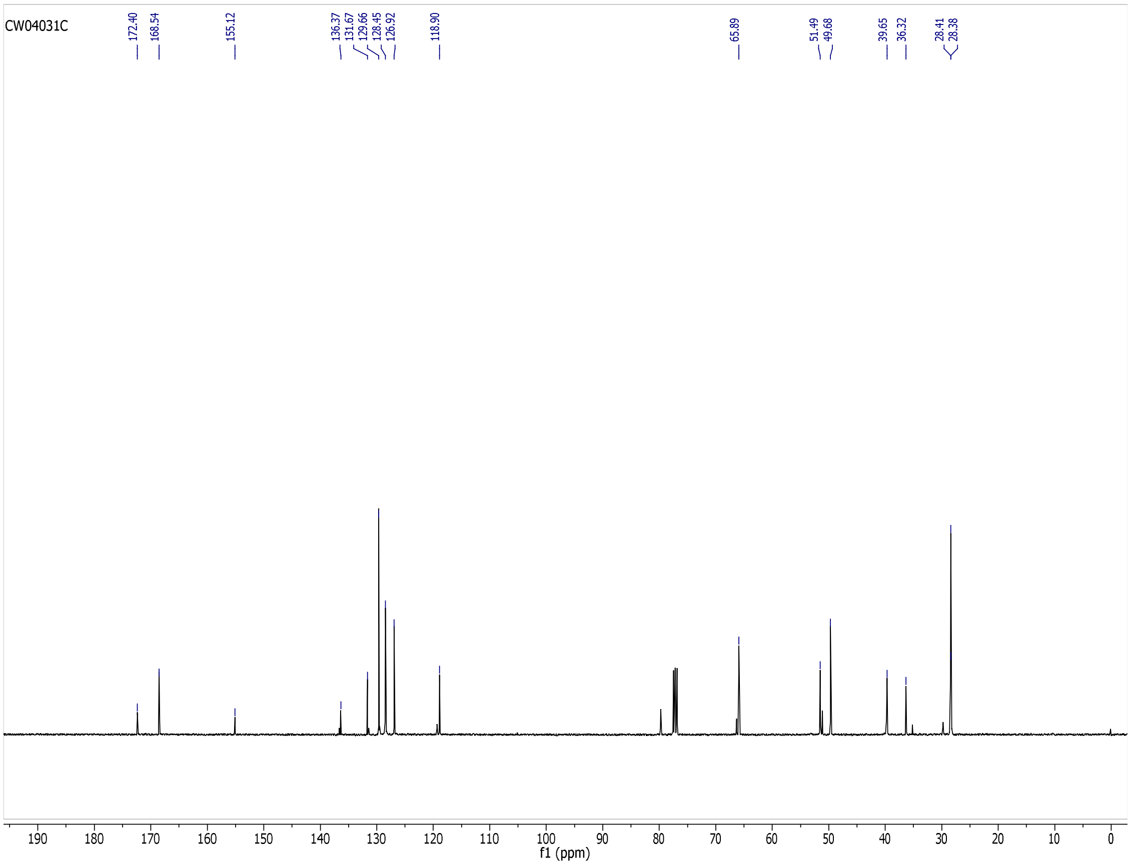
CW04031C
172.40
168.54
155.12
136.37
131.67
129.66
128.45
126.92
118.90
65.89
51.49
49.68
39.65
36.32
28.41
28.38
f1 (ppm)

HCl-Phe-Sar-OAllyl

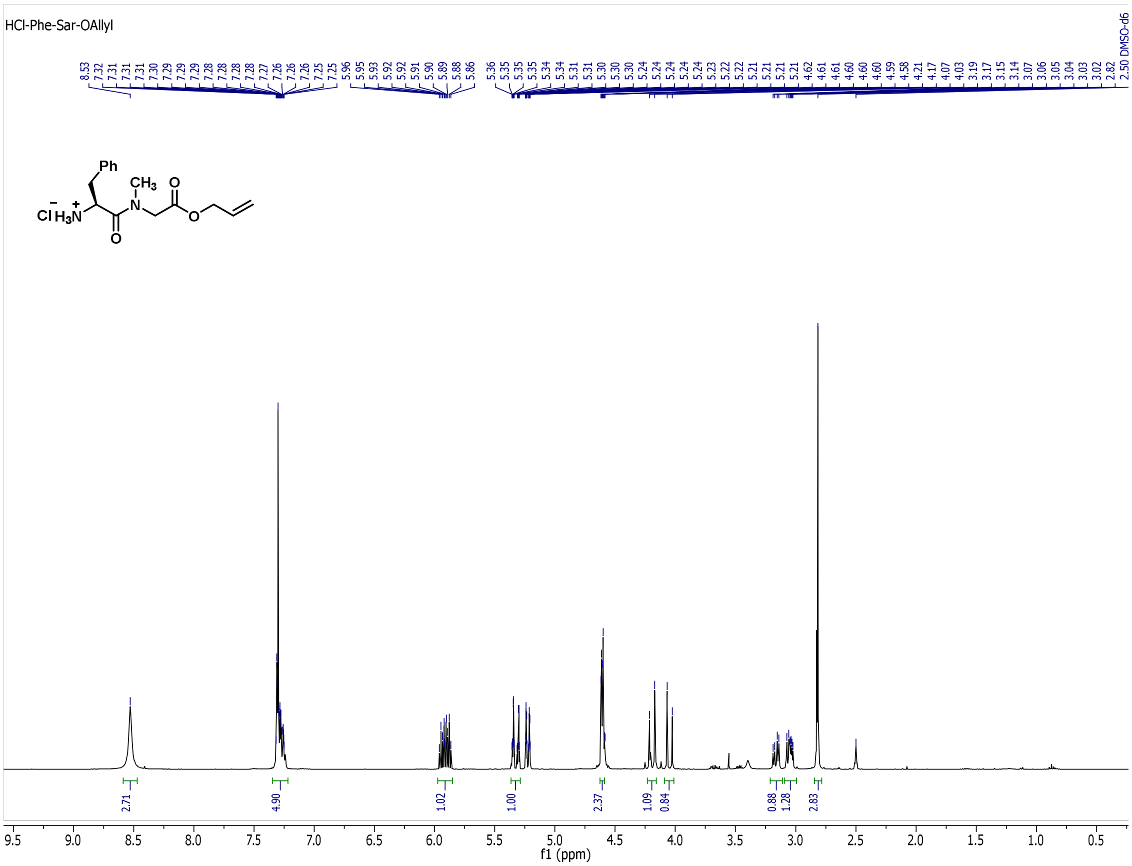

HCl-Phe-Sar-OAllyl

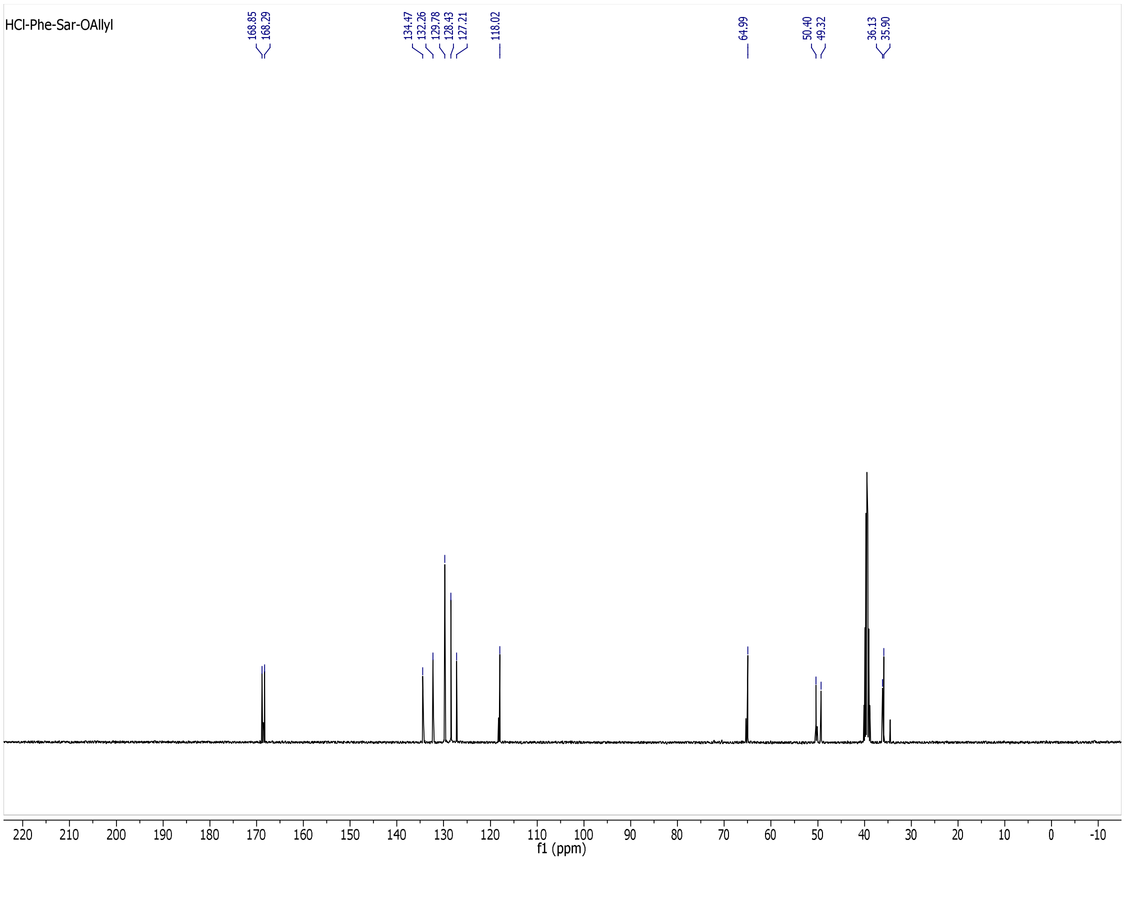

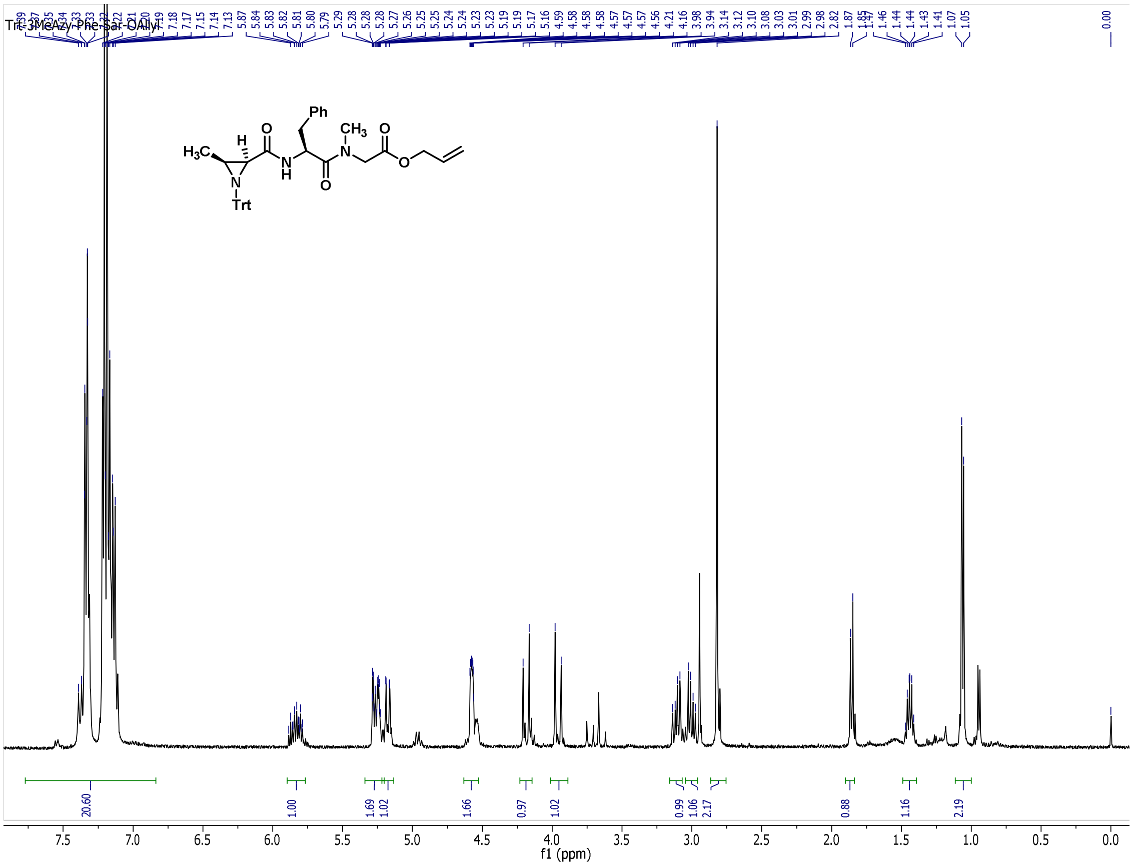
Trt-3MeAzy-Phe-Sar-OAllyl
f1 (ppm)

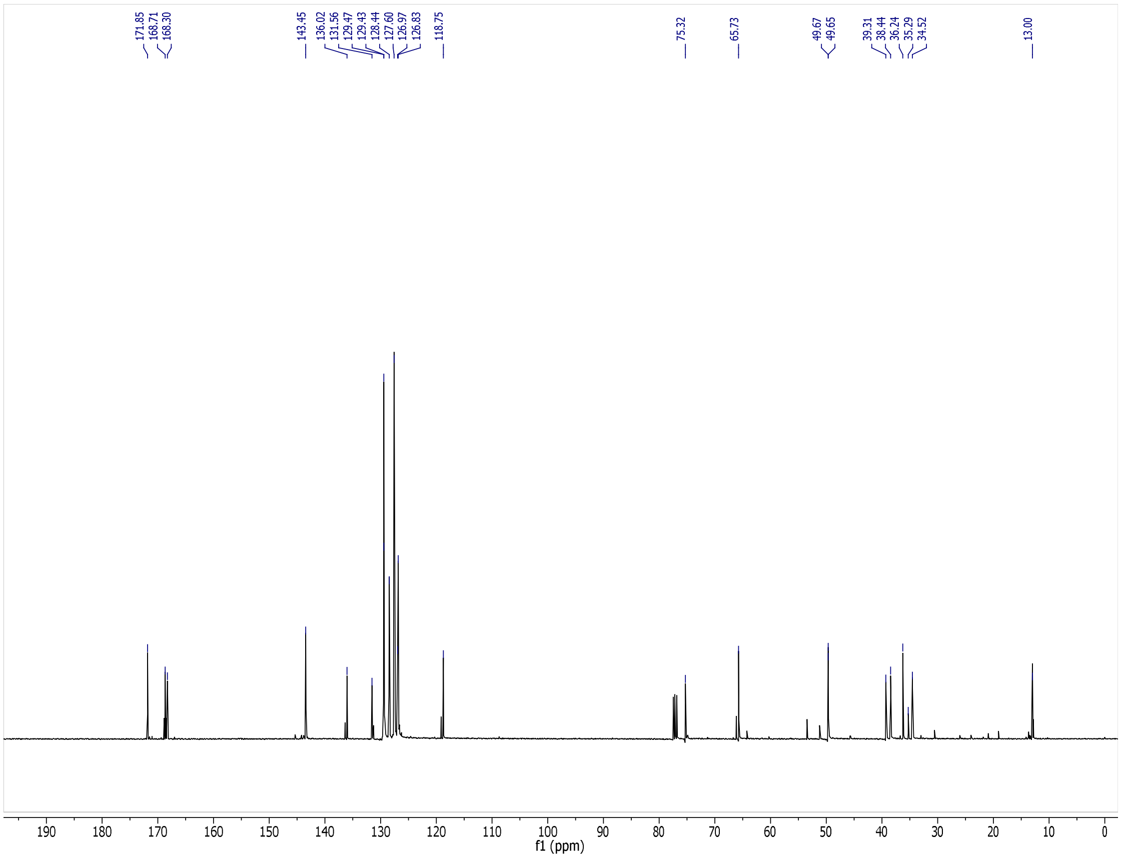
f1 (ppm)

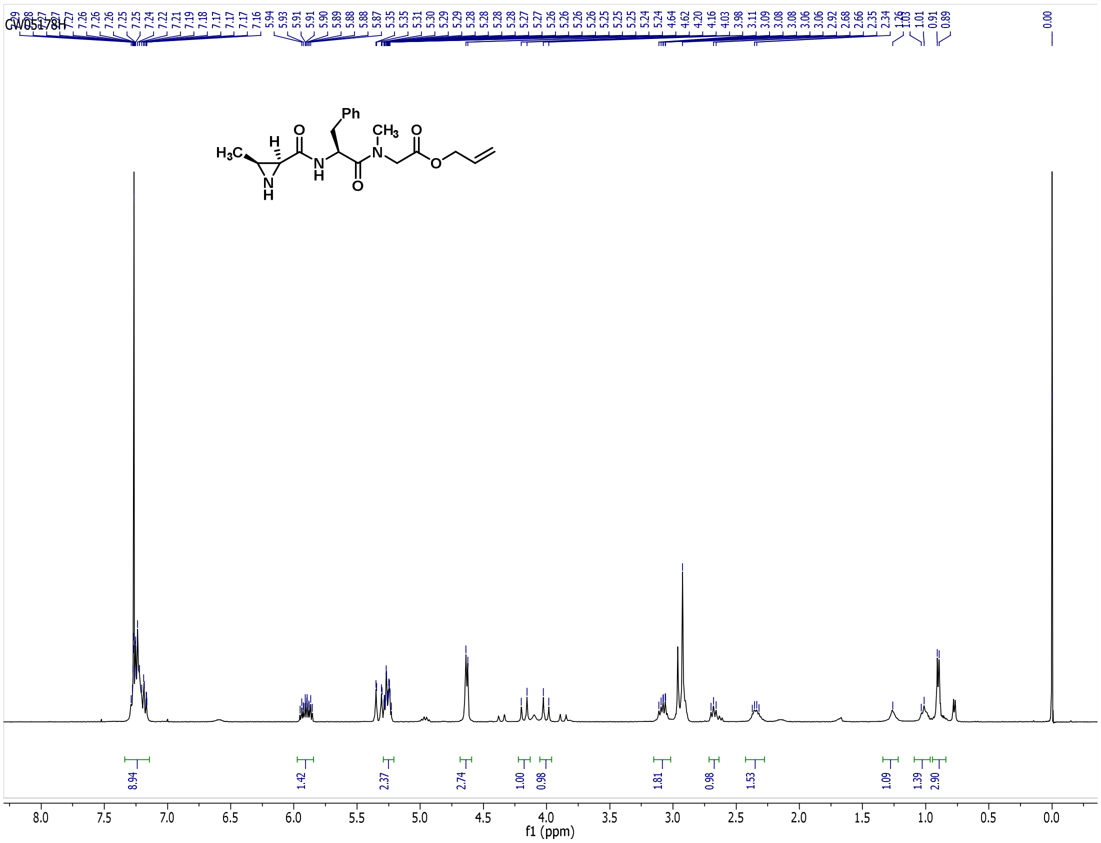
GW05178H
Ph
CH3
H3C
8.94
1.42
2.37
2.74
1.00
0.98
1.81
0.98
1.53
1.09
1.39
2.90
f1 (ppm)

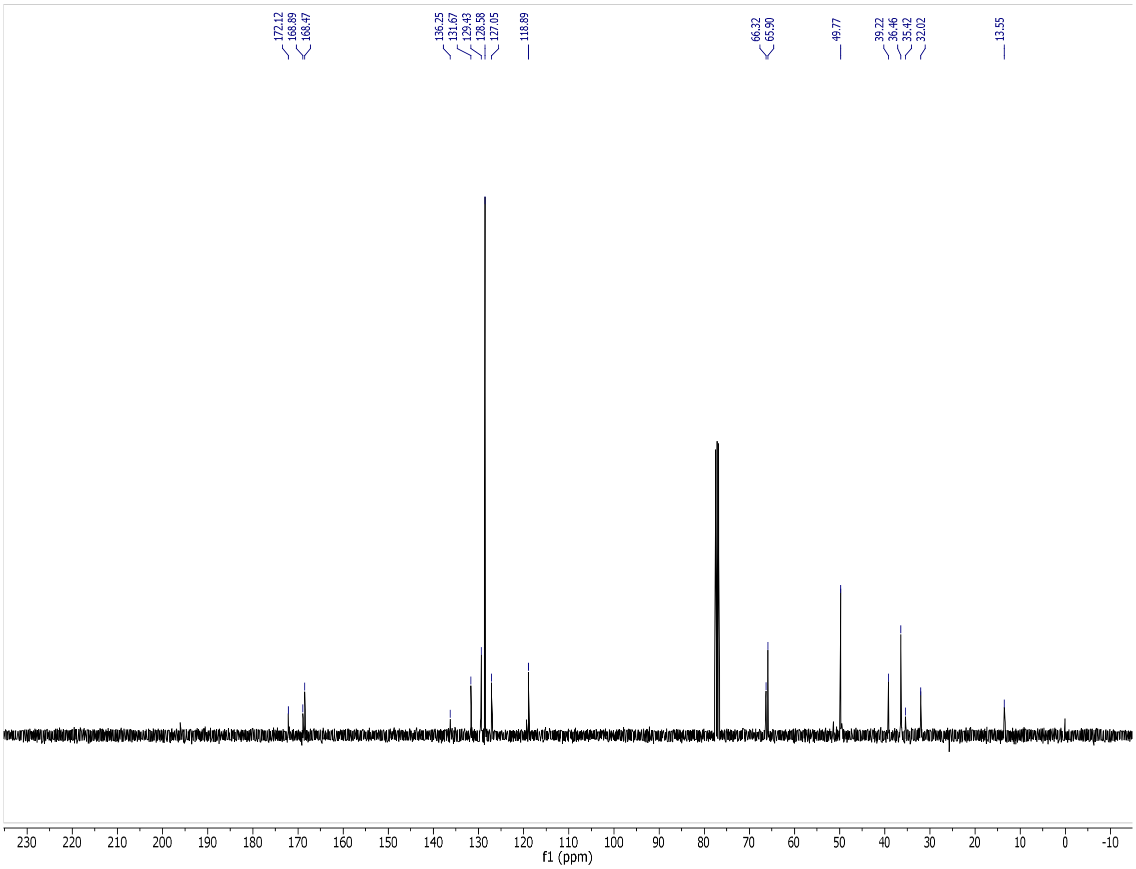
172.12
168.89
168.47
136.25
131.67
129.43
128.58
127.05
118.89
66.32
65.90
49.77
39.22
36.46
35.42
32.02
13.55
f1 (ppm)

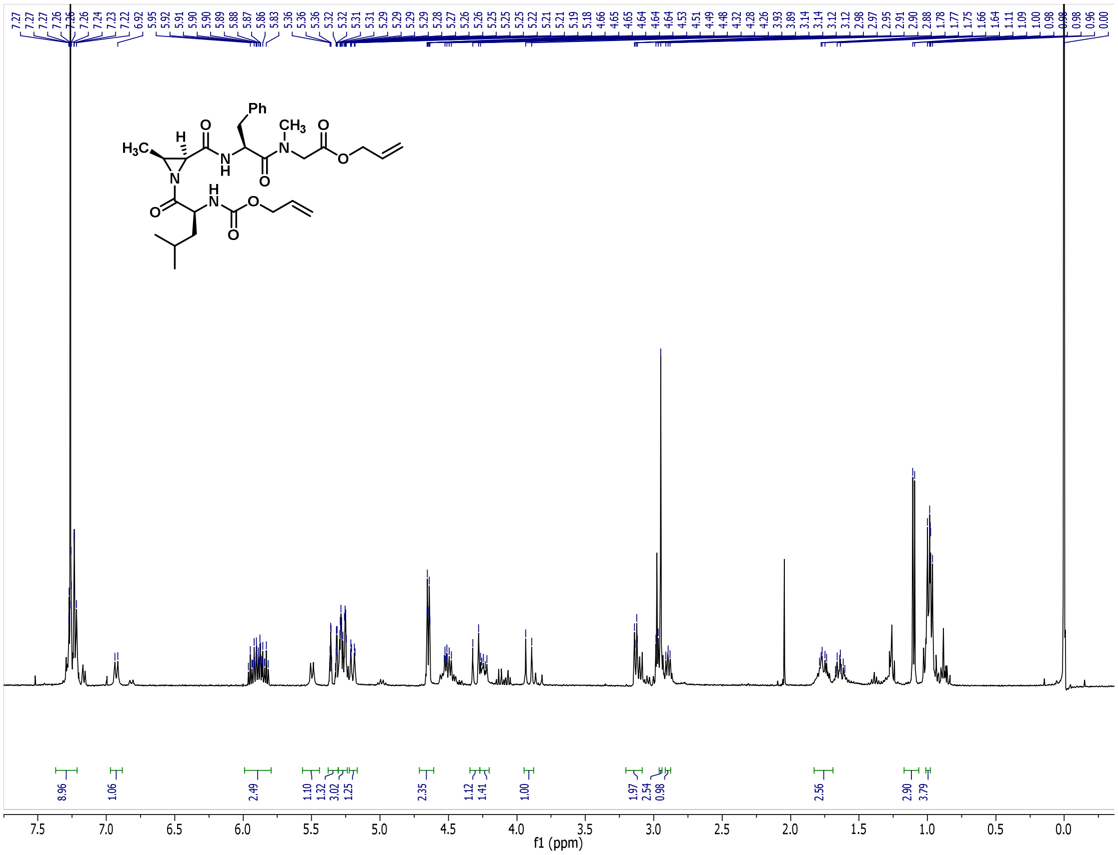
Ph
CH3
H
H3C
N
O
H
N
O
N
O
O
O
N
H
O
O
8.96
1.06
2.49
1.10
1.32
3.02
1.25
2.35
1.12
1.41
1.00
1.97
2.54
0.98
2.56
2.90
3.79
f1 (ppm)

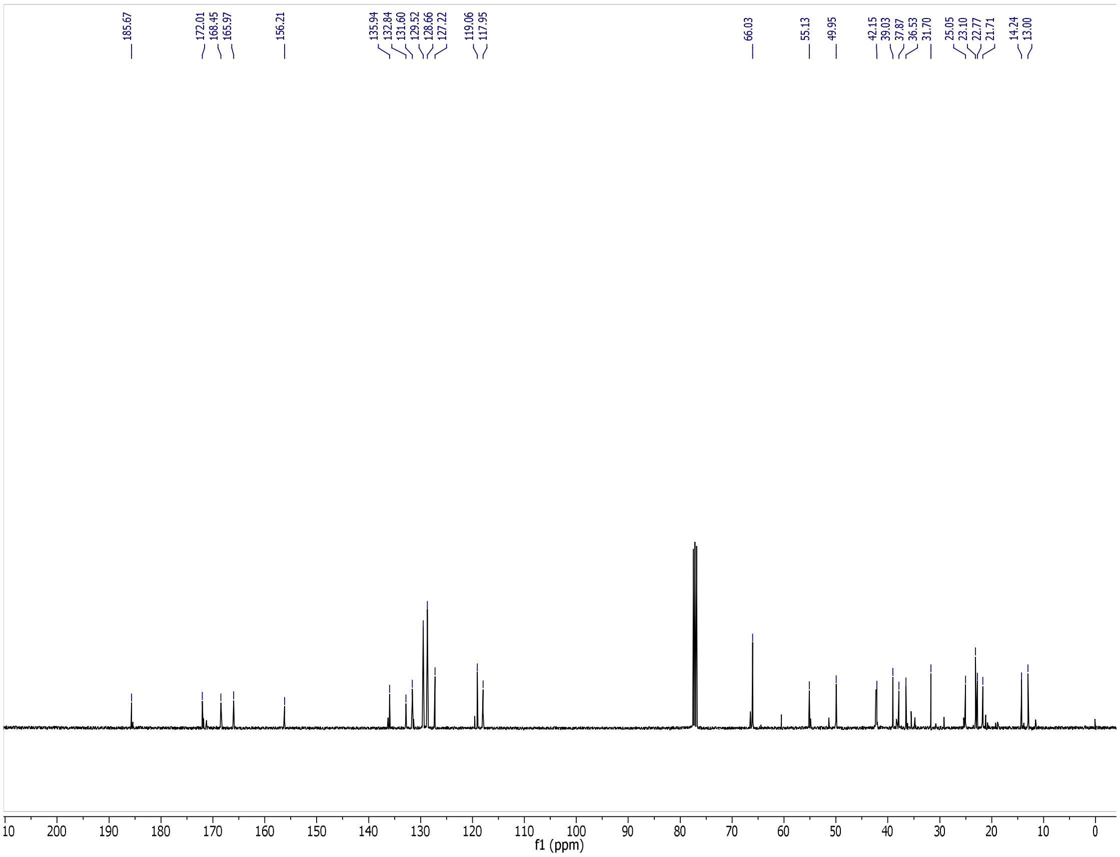
185.67
172.01
168.45
165.97
156.21
135.94
132.84
131.60
129.52
128.66
127.22
119.06
117.95
66.03
55.13
49.95
42.15
39.03
37.87
36.53
31.70
25.05
23.10
22.77
21.71
14.24
13.00
f1 (ppm)

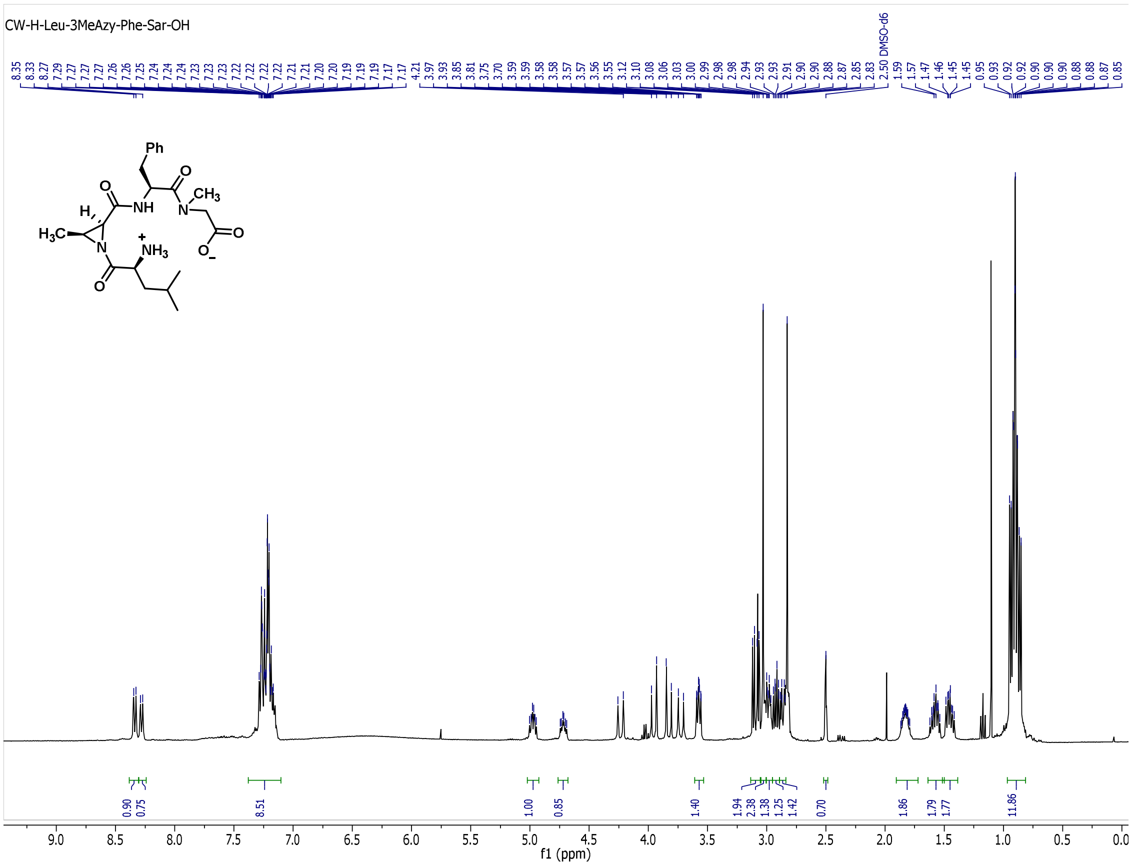
CW-H-Leu-3MeAzy-Phe-Sar-OH
8.35 8.33 8.27 7.29 7.27 7.27 7.27 7.26 7.25 7.24 7.24 7.24 7.23 7.23 7.23 7.22 7.22 7.22 7.22 7.21 7.21 7.20 7.20 7.19 7.19 7.19 7.17 7.17 4.21 3.97 3.93 3.85 3.81 3.75 3.70 3.59 3.59 3.58 3.58 3.57 3.57 3.56 3.55 3.12 3.10 3.08 3.06 3.03 3.00 2.99 2.98 2.98 2.94 2.93 2.93 2.91 2.90 2.90 2.88 2.87 2.85 2.83 2.50 DMSO-d6 1.59 1.57 1.47 1.46 1.45 1.45 0.95 0.93 0.92 0.92 0.90 0.90 0.90 0.90 0.88 0.88 0.87 0.85
0.90 0.75 8.51 1.00 0.85 1.40 1.94 2.38 1.38 1.25 1.42 0.70 1.86 1.79 1.77 11.86
f1 (ppm)

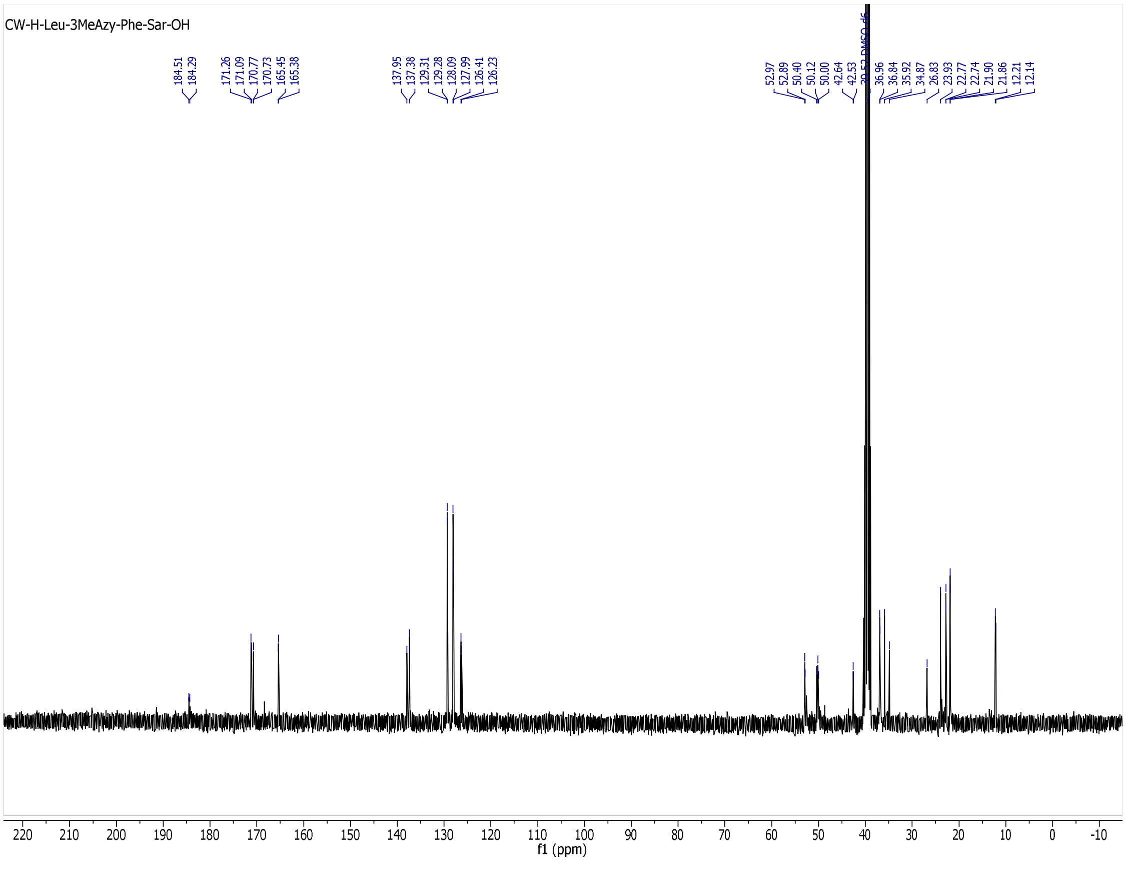
CW-H-Leu-3MeAzy-Phe-Sar-OH
184.51 184.29 171.26 171.09 170.77 170.73 165.45 165.38 137.95 137.38 129.31 129.28 128.09 127.99 126.41 126.23 52.97 52.89 50.40 50.12 50.00 42.64 42.53 [illegible] DMSO-d6 36.96 36.84 35.92 34.87 26.83 23.93 22.77 22.74 21.90 21.86 12.21 12.14
220 210 200 190 180 170 160 150 140 130 120 110 100 90 80 70 60 50 40 30 20 10 0 -10
f1 (ppm)

COSY spectrum

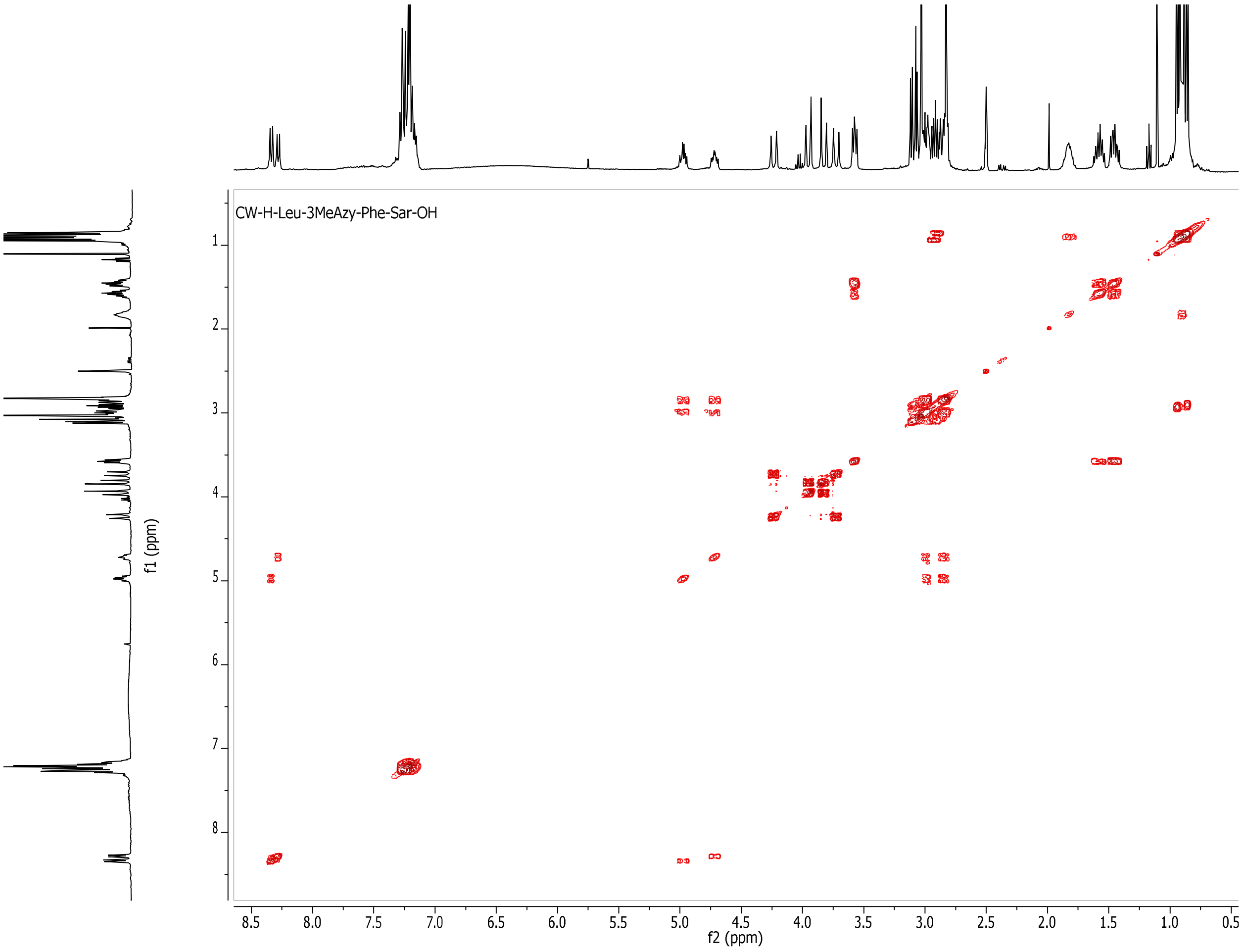

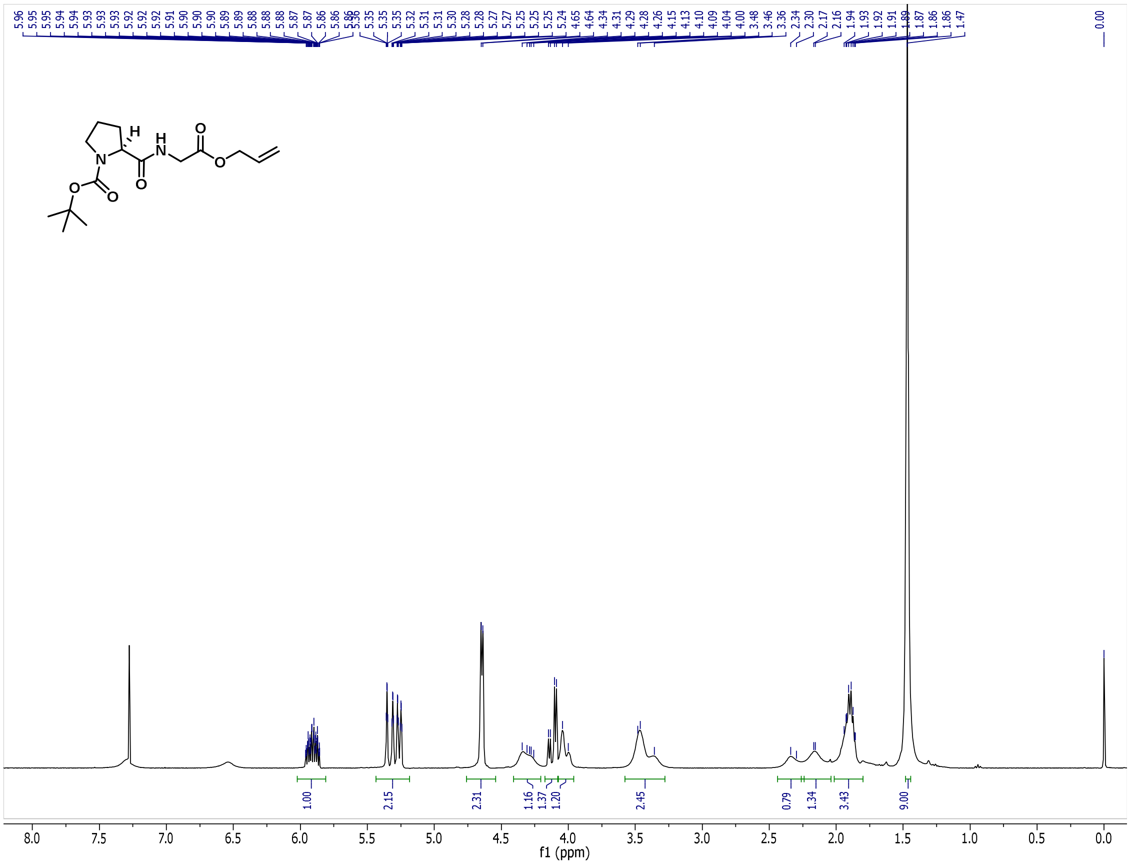
1.00
2.15
2.31
1.16
1.37
1.20
2.45
0.79
1.34
3.43
9.00
0.00
8.0 7.5 7.0 6.5 6.0 5.5 5.0 4.5 4.0 3.5 3.0 2.5 2.0 1.5 1.0 0.5 0.0
f1 (ppm)

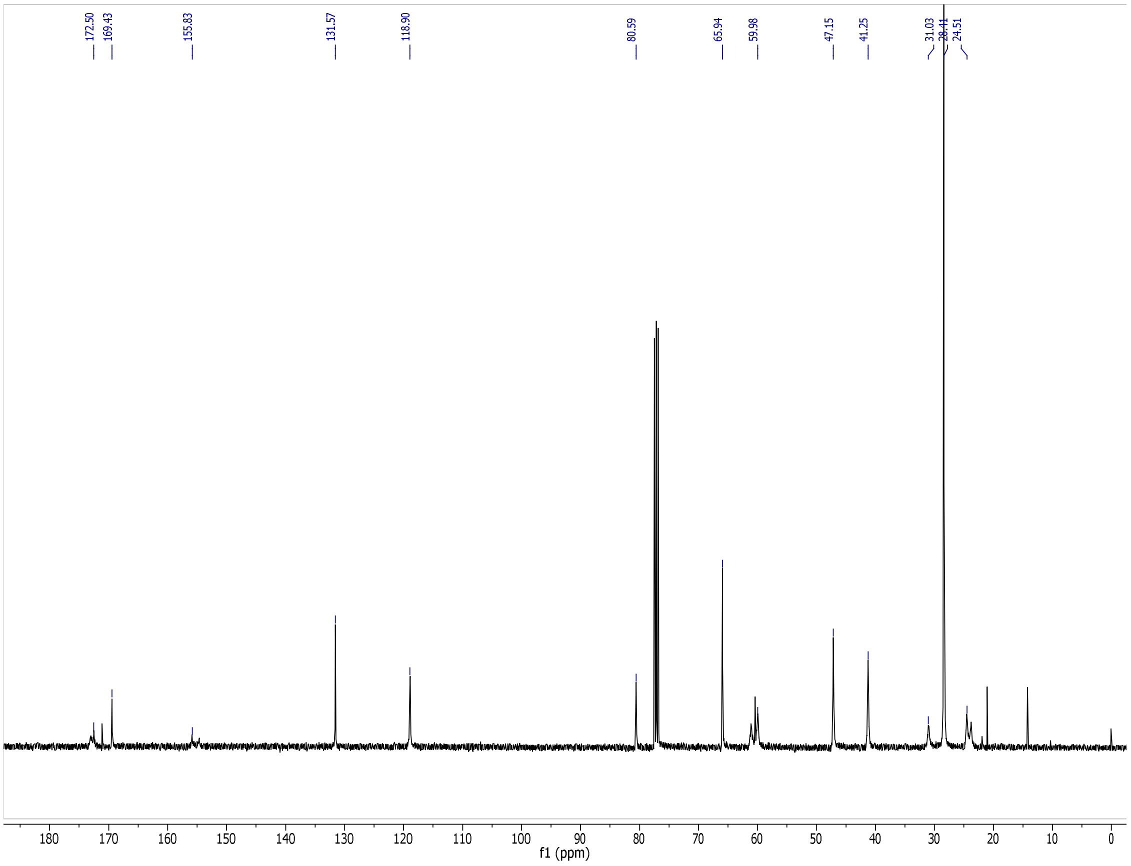
172.50
169.43
155.83
131.57
118.90
80.59
65.94
59.98
47.15
41.25
31.03
28.41
24.51
180 170 160 150 140 130 120 110 100 90 80 70 60 50 40 30 20 10 0
f1 (ppm)

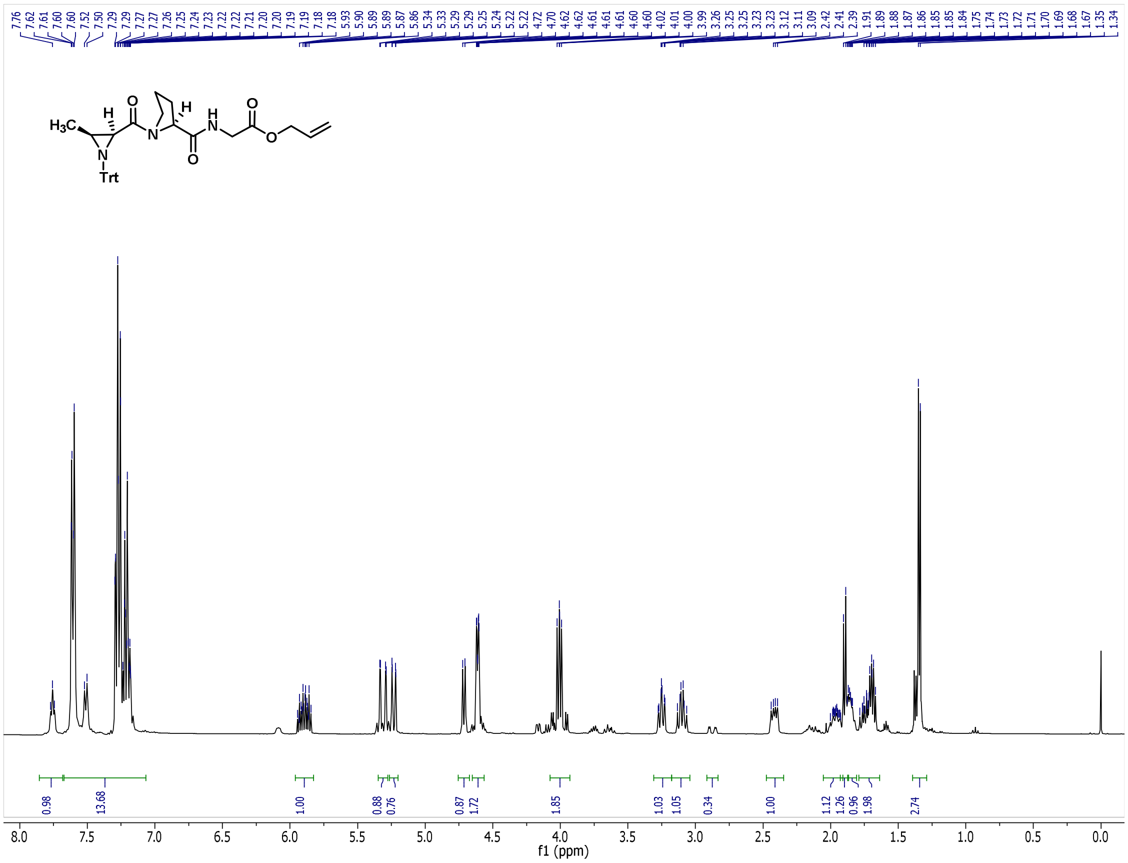
7.76 7.62 7.61 7.60 7.60 7.52 7.50 7.29 7.29 7.27 7.27 7.26 7.25 7.24 7.23 7.22 7.22 7.21 7.20 7.20 7.19 7.19 7.18 7.18 5.93 5.90 5.89 5.89 5.87 5.86 5.34 5.33 5.29 5.29 5.25 5.24 5.22 5.22 4.72 4.70 4.62 4.62 4.61 4.61 4.61 4.60 4.60 4.02 4.01 4.00 3.99 3.26 3.25 3.25 3.23 3.12 3.11 3.09 2.42 2.41 2.39 1.91 1.89 1.88 1.87 1.86 1.85 1.85 1.84 1.75 1.74 1.74 1.73 1.72 1.71 1.70 1.69 1.68 1.67 1.35 1.34
H3C
H
O
N
H
Trt
0.98 13.68 1.00 0.88 0.76 0.87 1.72 1.85 1.03 1.05 0.34 1.00 1.12 1.26 0.96 1.98 2.74
8.0 7.5 7.0 6.5 6.0 5.5 5.0 4.5 4.0 3.5 3.0 2.5 2.0 1.5 1.0 0.5 0.0
f1 (ppm)

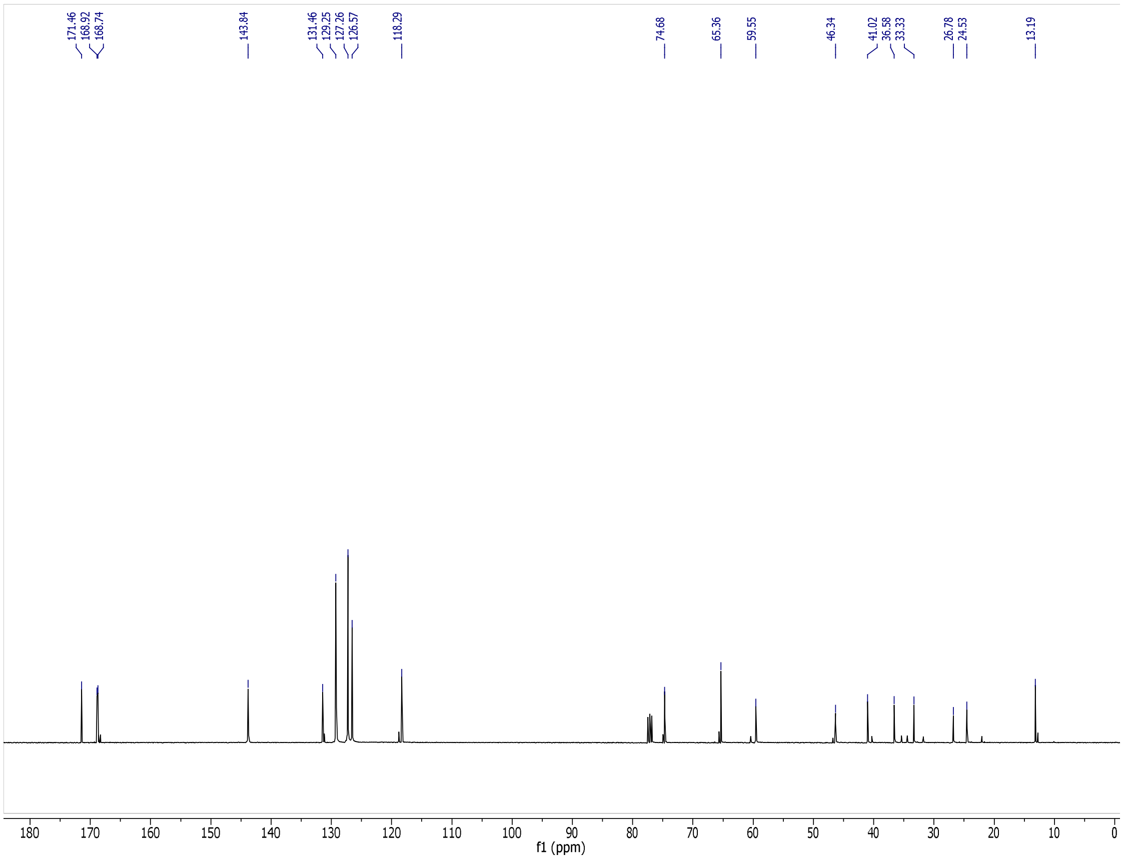
171.46 168.92 168.74 143.84 131.46 129.25 127.26 126.57 118.29 74.68 65.36 59.55 46.34 41.02 36.58 33.33 26.78 24.53 13.19
180 170 160 150 140 130 120 110 100 90 80 70 60 50 40 30 20 10 0
f1 (ppm)

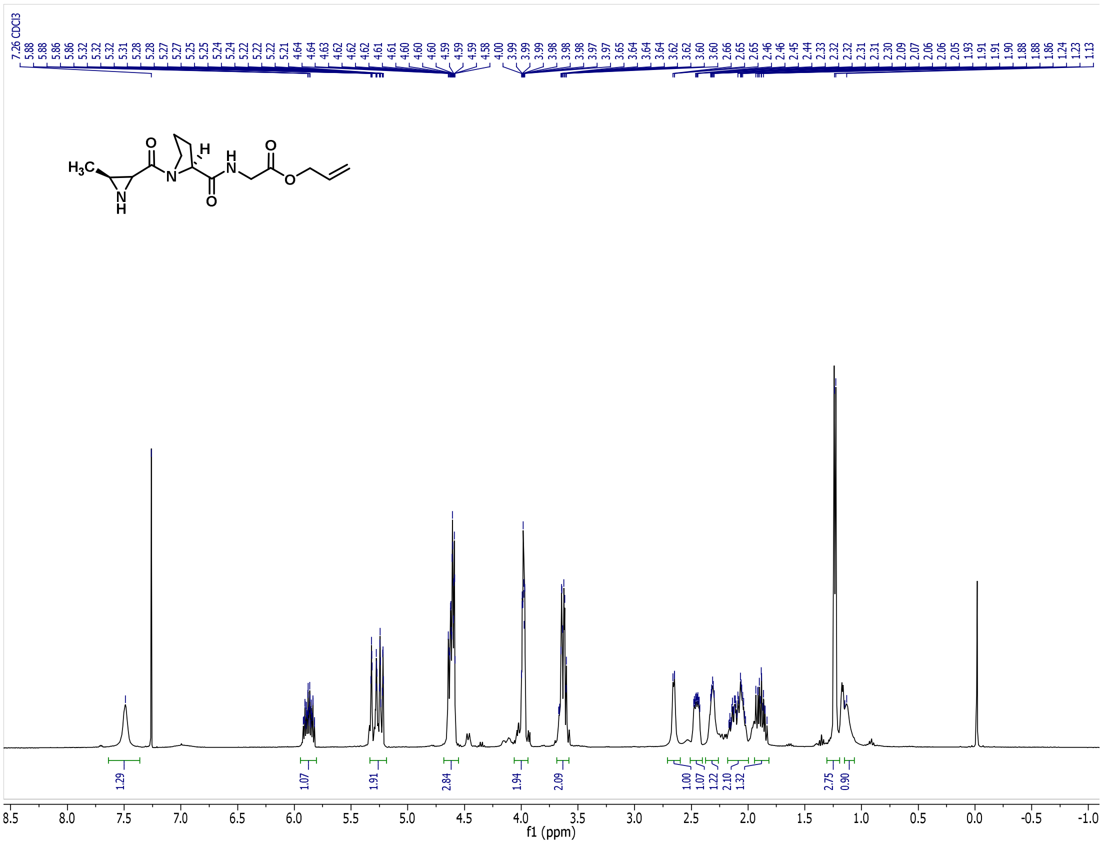
7.26 CDCl3
5.88
5.88
5.86
5.86
5.32
5.32
5.32
5.31
5.28
5.28
5.27
5.27
5.25
5.25
5.24
5.24
5.22
5.22
5.22
5.21
4.64
4.64
4.63
4.62
4.62
4.62
4.61
4.61
4.60
4.60
4.59
4.59
4.59
4.59
4.58
4.00
3.99
3.99
3.99
3.98
3.98
3.98
3.97
3.97
3.65
3.64
3.64
3.62
3.62
3.60
3.60
2.66
2.65
2.65
2.46
2.46
2.45
2.44
2.33
2.32
2.32
2.31
2.30
2.09
2.07
2.06
2.06
2.05
1.93
1.91
1.91
1.90
1.88
1.86
1.24
1.23
1.13
H3C
N
H
O
N
H
H
N
O
O
O
1.29
1.07
1.91
2.84
1.94
2.09
1.00
1.07
1.22
2.10
1.32
2.75
0.90
8.5
8.0
7.5
7.0
6.5
6.0
5.5
5.0
4.5
4.0
3.5
3.0
2.5
2.0
1.5
1.0
0.5
0.0
-0.5
-1.0
f1 (ppm)

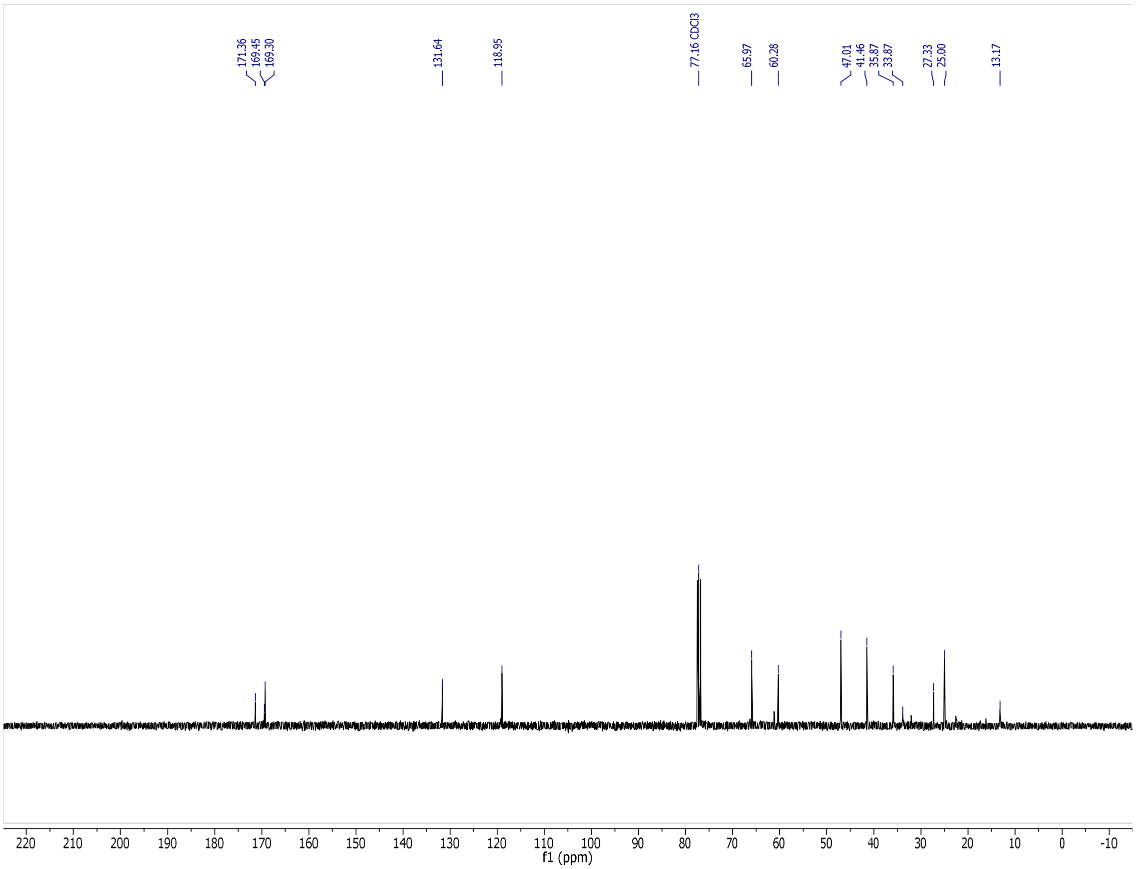
171.36
169.45
169.30
131.64
118.95
77.16 CDCl3
65.97
60.28
47.01
41.46
35.87
33.87
27.33
25.00
13.17
220
210
200
190
180
170
160
150
140
130
120
110
100
90
80
70
60
50
40
30
20
10
0
-10
f1 (ppm)

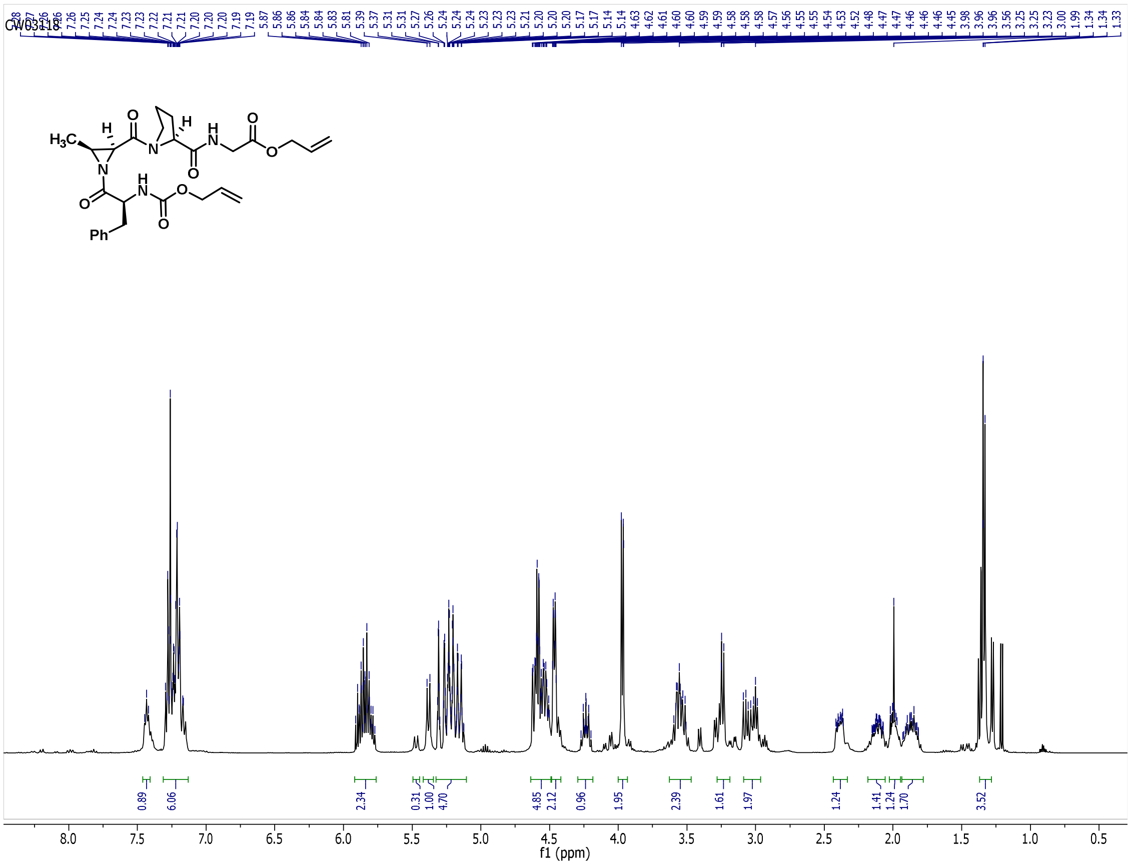
CW03118
7.26 7.26 7.25 7.24 7.24 7.23 7.23 7.23 7.22 7.21 7.21 7.20 7.20 7.20 7.19 7.19 5.87 5.86 5.86 5.84 5.84 5.83 5.81 5.39 5.37 5.31 5.31 5.27 5.26 5.24 5.24 5.24 5.24 5.23 5.23 5.23 5.21 5.20 5.20 5.20 5.17 5.17 5.14 5.14 4.63 4.62 4.61 4.60 4.60 4.59 4.59 4.58 4.58 4.58 4.57 4.56 4.55 4.55 4.54 4.53 4.52 4.48 4.47 4.47 4.46 4.46 4.46 4.45 3.98 3.96 3.96 3.56 3.25 3.23 3.00 1.99 1.34 1.34 1.33
H
H
H3C
N
O
N
H
N
O
O
O
N
O
H
N
O
O
Ph
0.89
6.06
2.34
0.31
1.00
4.70
4.85
2.12
0.96
1.95
2.39
1.61
1.97
1.24
1.41
1.24
1.70
3.52
8.0 7.5 7.0 6.5 6.0 5.5 5.0 4.5 4.0 3.5 3.0 2.5 2.0 1.5 1.0 0.5
f1 (ppm)

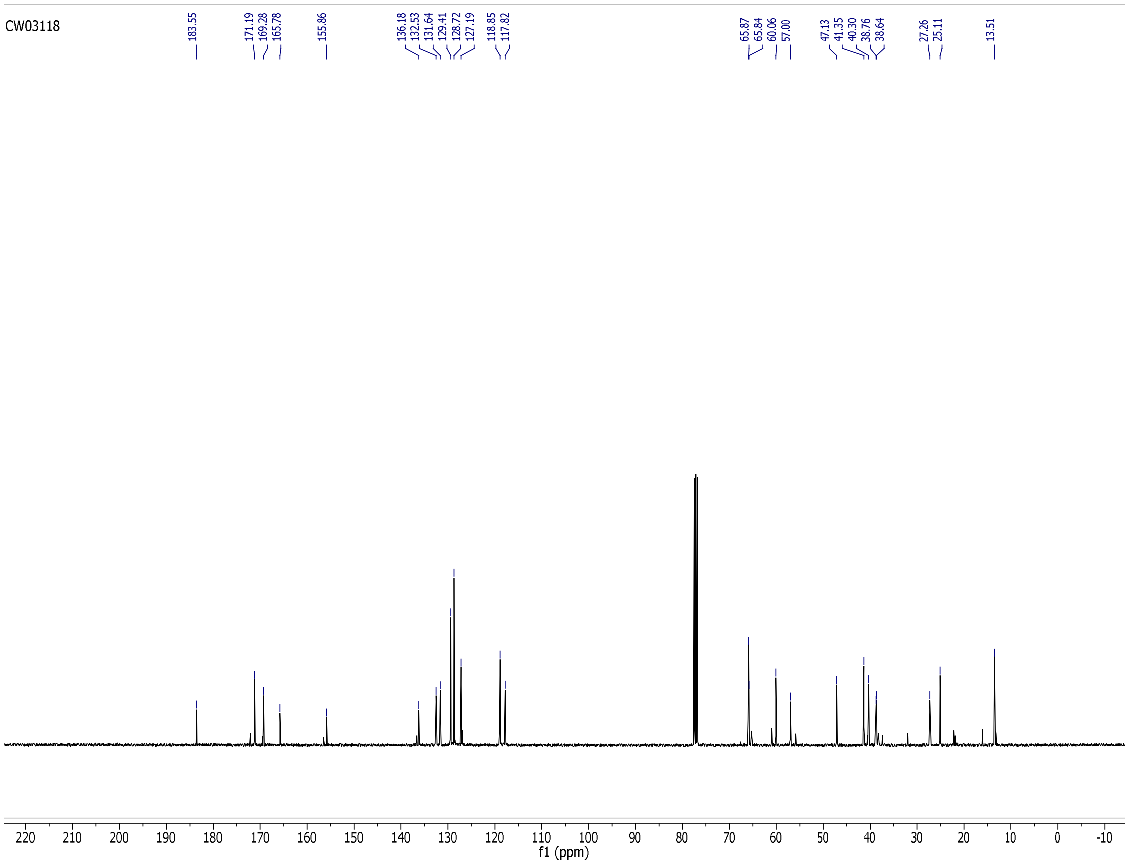
CW03118
183.55
171.19
169.28
165.78
155.86
136.18
132.53
131.64
129.41
128.72
127.19
118.85
117.82
65.87
65.84
60.06
57.00
47.13
41.35
40.30
38.76
38.64
27.26
25.11
13.51
220 210 200 190 180 170 160 150 140 130 120 110 100 90 80 70 60 50 40 30 20 10 0 -10
f1 (ppm)

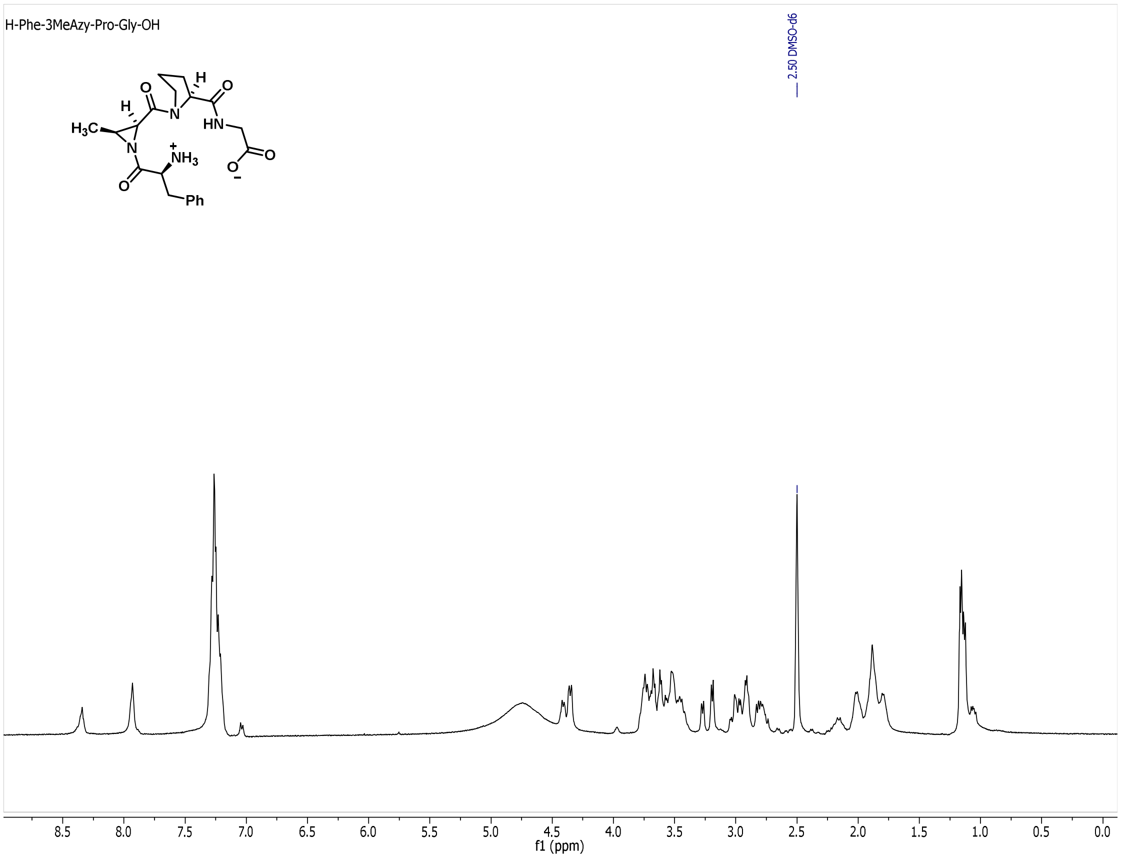
H-Phe-3MeAzy-Pro-Gly-OH
2.50 DMSO-d6
H
H
H_3C
N
N
O
O
HN
$\overset{+}{N}H_3$
O
O
Ph
8.5 8.0 7.5 7.0 6.5 6.0 5.5 5.0 4.5 4.0 3.5 3.0 2.5 2.0 1.5 1.0 0.5 0.0
f1 (ppm)

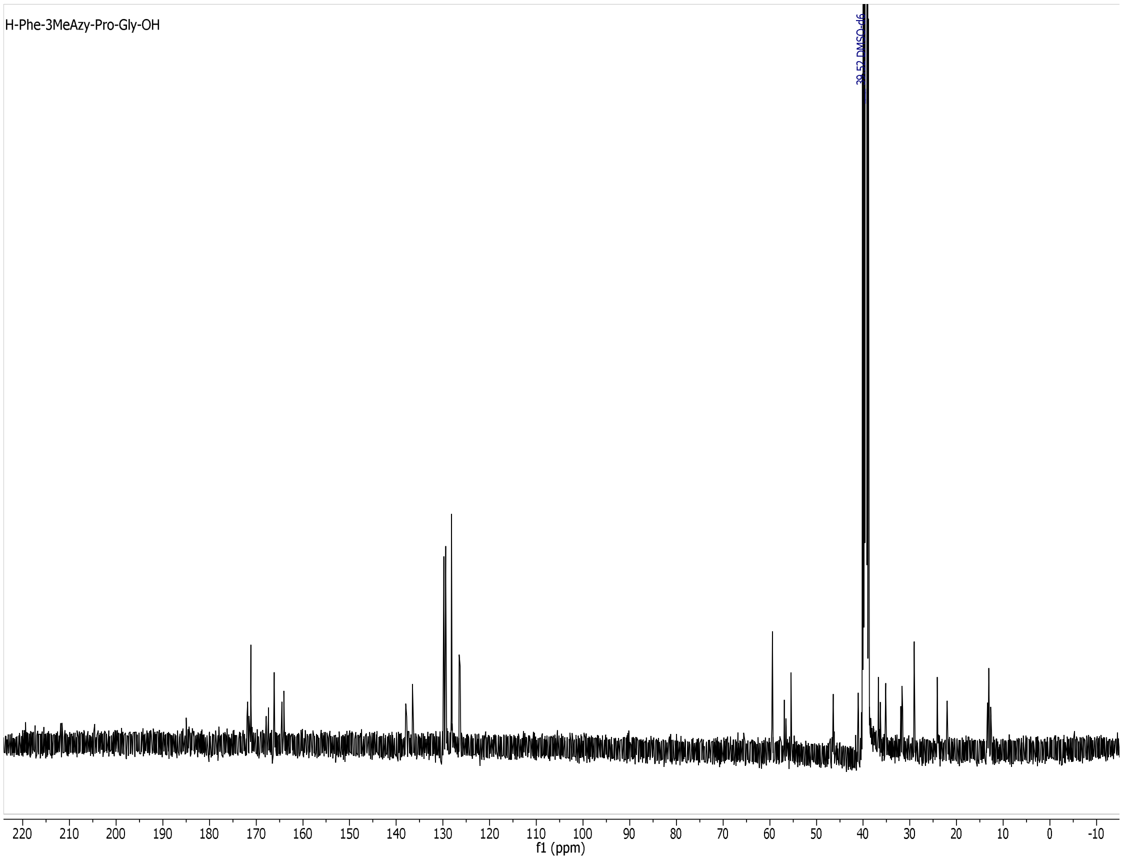
H-Phe-3MeAzy-Pro-Gly-OH
39.52 DMSO-d6
220 210 200 190 180 170 160 150 140 130 120 110 100 90 80 70 60 50 40 30 20 10 0 -10
f1 (ppm)

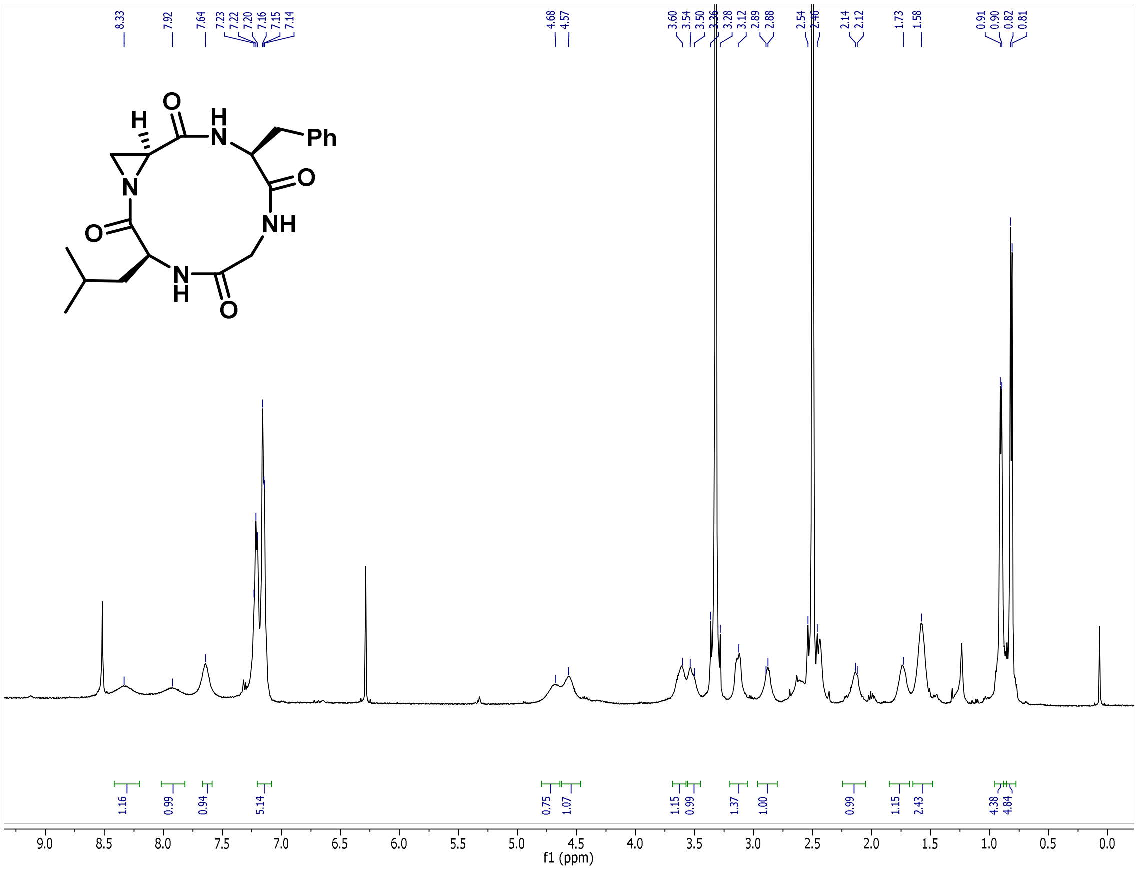
8.33
7.92
7.64
7.23
7.22
7.20
7.16
7.15
7.14
4.68
4.57
3.60
3.54
3.50
3.28
3.12
2.89
2.88
2.54
2.46
2.14
2.12
1.73
1.58
0.91
0.90
0.82
0.81
H
O
H
N
Ph
N
O
O
NH
N
H
O
1.16
0.99
0.94
5.14
0.75
1.07
1.15
0.99
1.37
1.00
0.99
1.15
2.43
4.38
4.84
9.0 8.5 8.0 7.5 7.0 6.5 6.0 5.5 5.0 4.5 4.0 3.5 3.0 2.5 2.0 1.5 1.0 0.5 0.0
f1 (ppm)

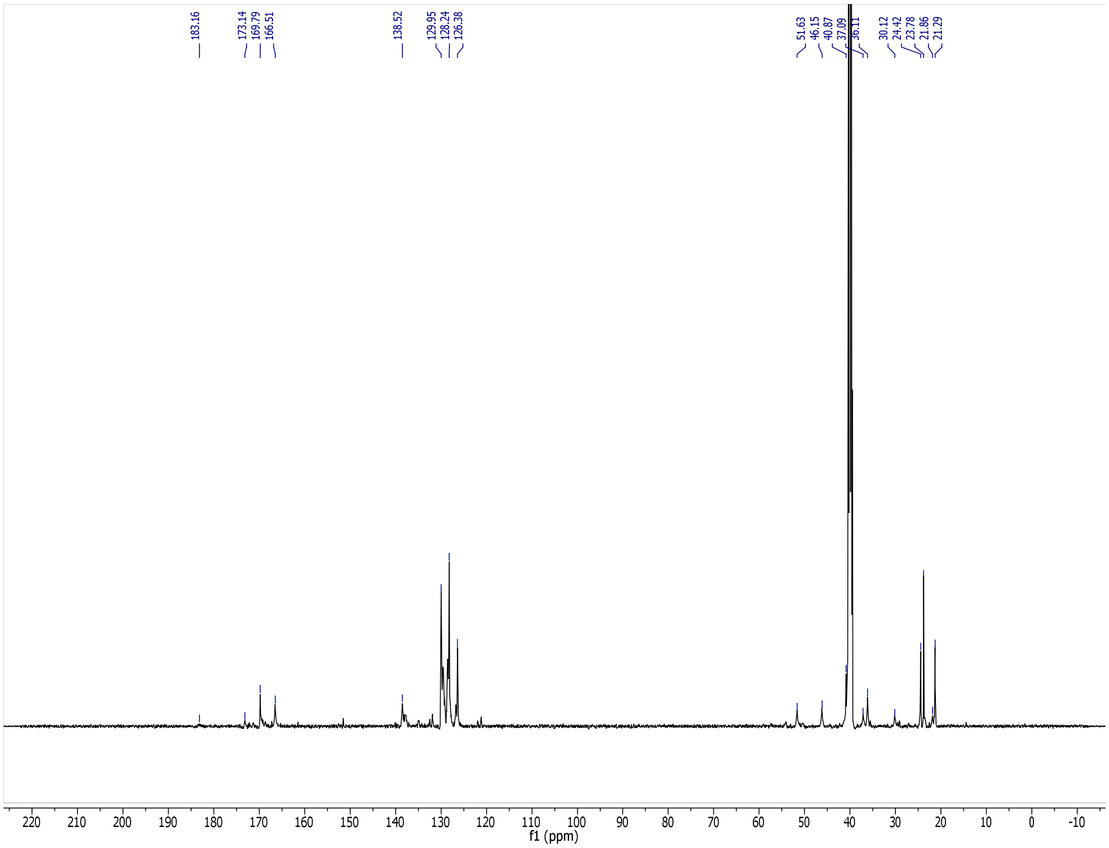
183.16
173.14
169.79
166.51
138.52
129.95
128.24
126.38
51.63
46.15
40.87
37.09
36.11
30.12
24.42
23.78
21.86
21.29
220 210 200 190 180 170 160 150 140 130 120 110 100 90 80 70 60 50 40 30 20 10 0 -10
f1 (ppm)

COSY spectrum

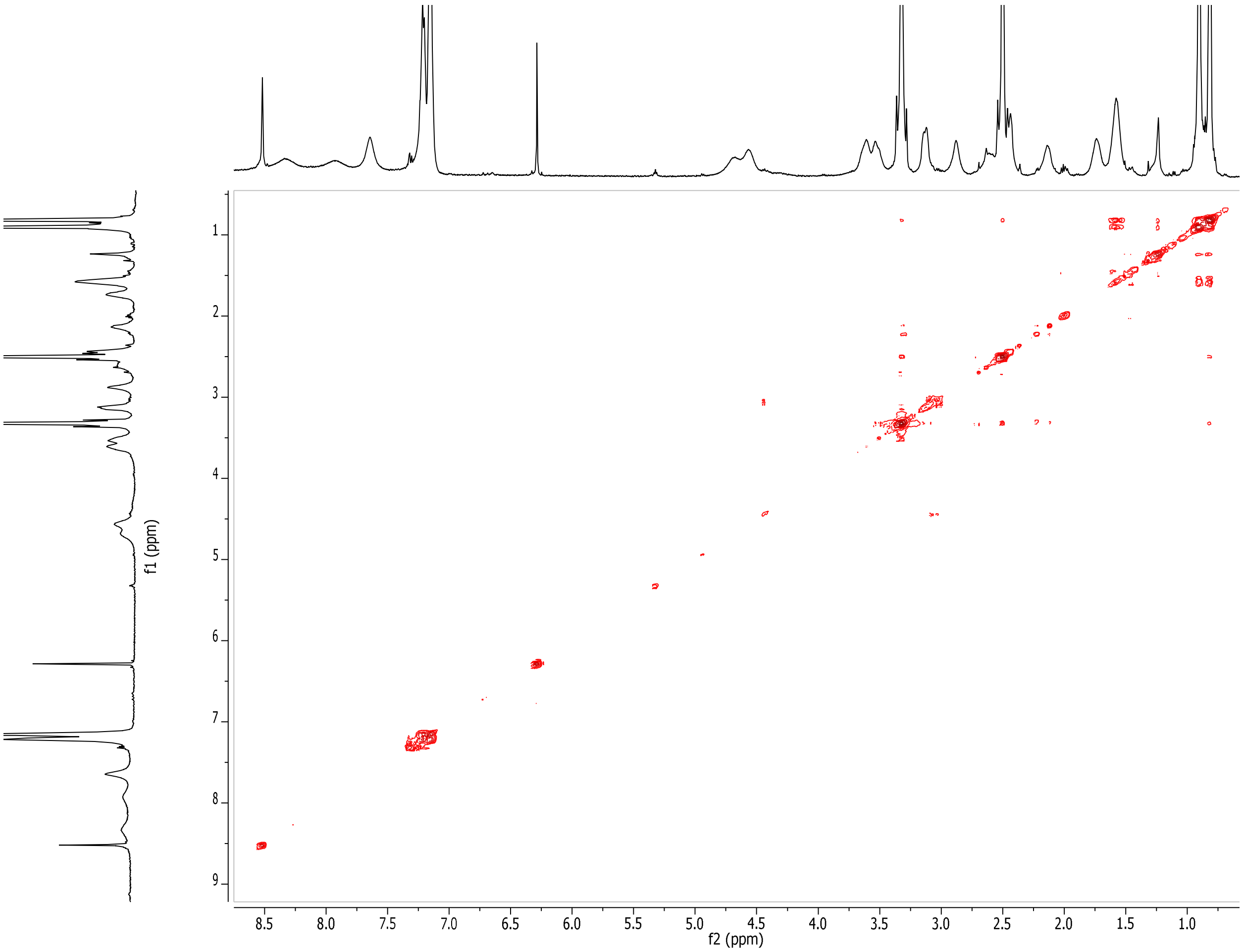

CW07163-ppt-wash

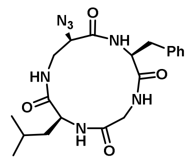

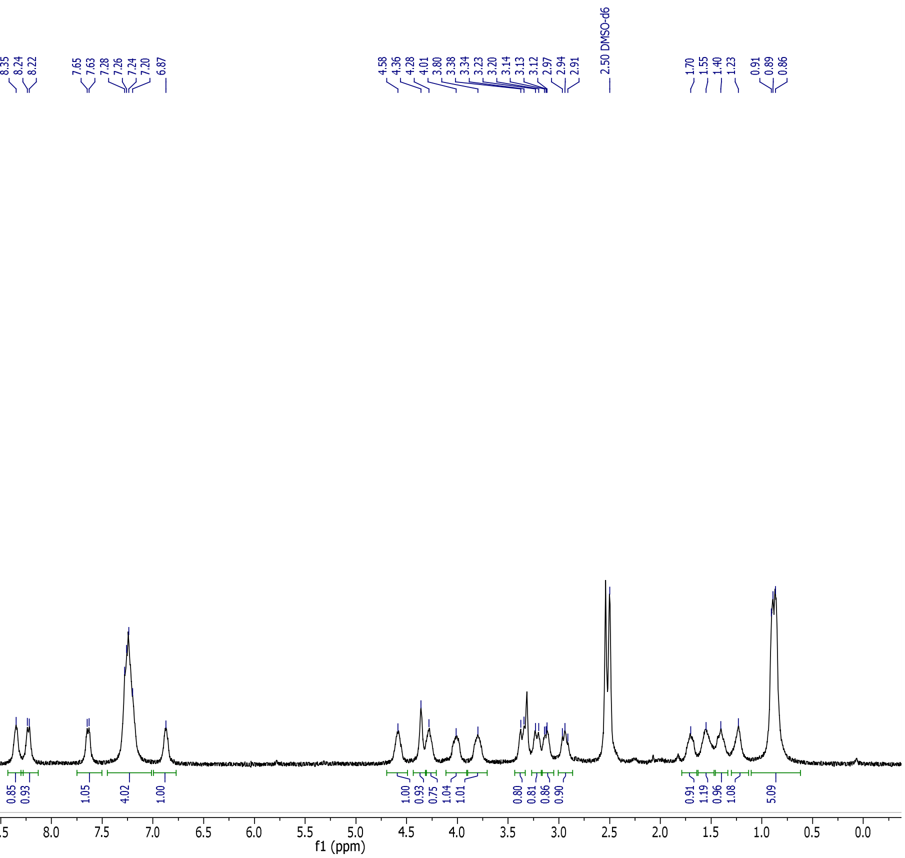

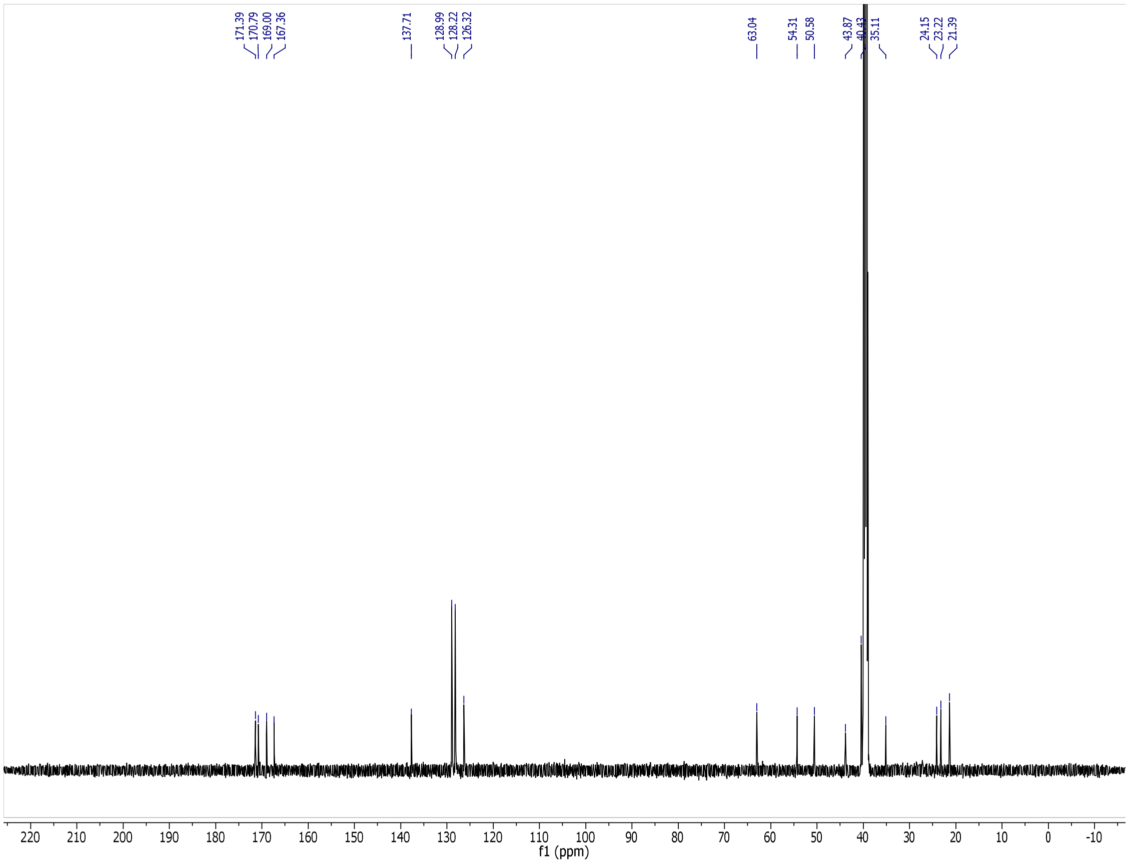

COSY spectrum

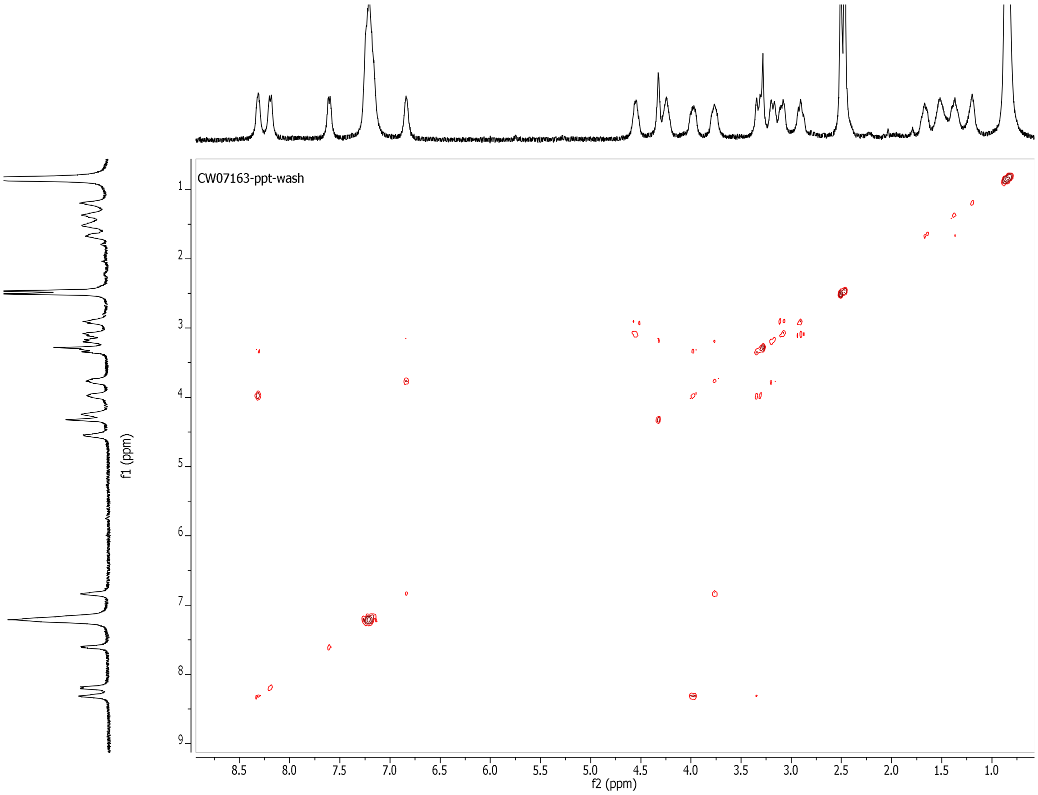

TOCSY spectrum

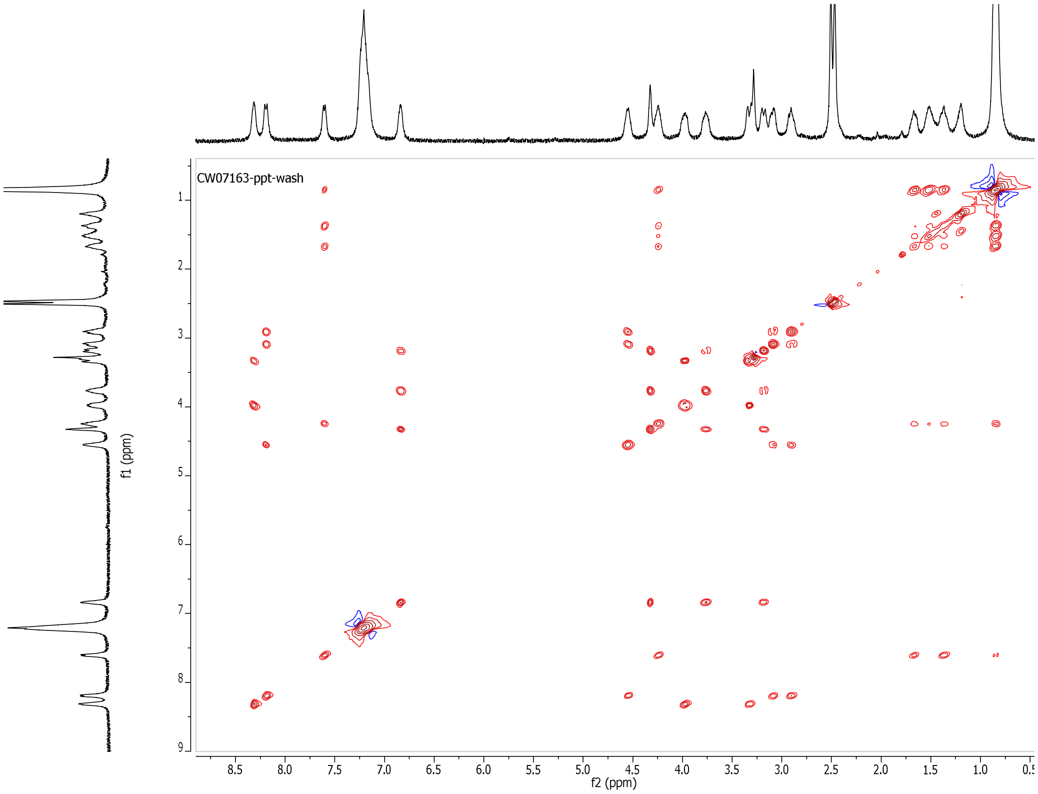

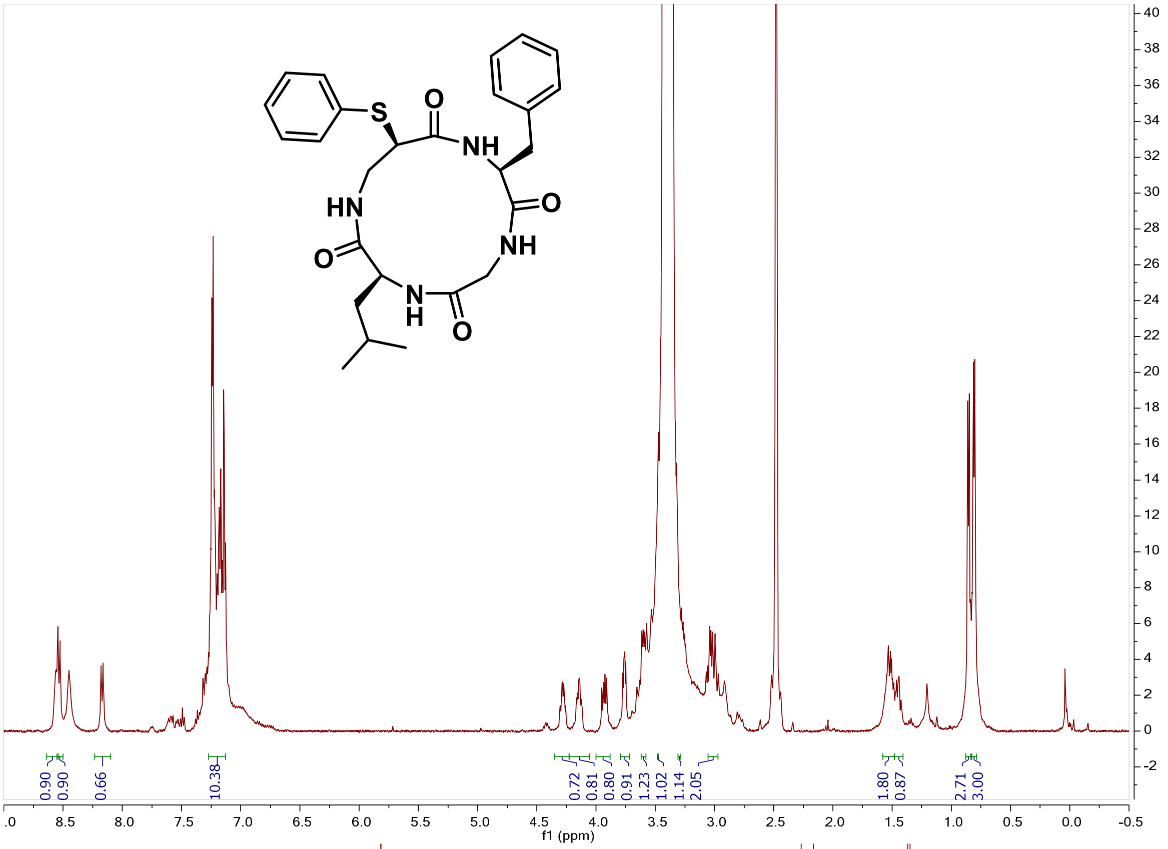
S
O
NH
HN
O
NH
O
N
H
O
0.90
0.90
0.66
10.38
0.72
0.81
0.80
0.91
1.23
1.02
1.14
2.05
1.80
0.87
2.71
3.00
f1 (ppm)

^{1}H-^{1}H COSY

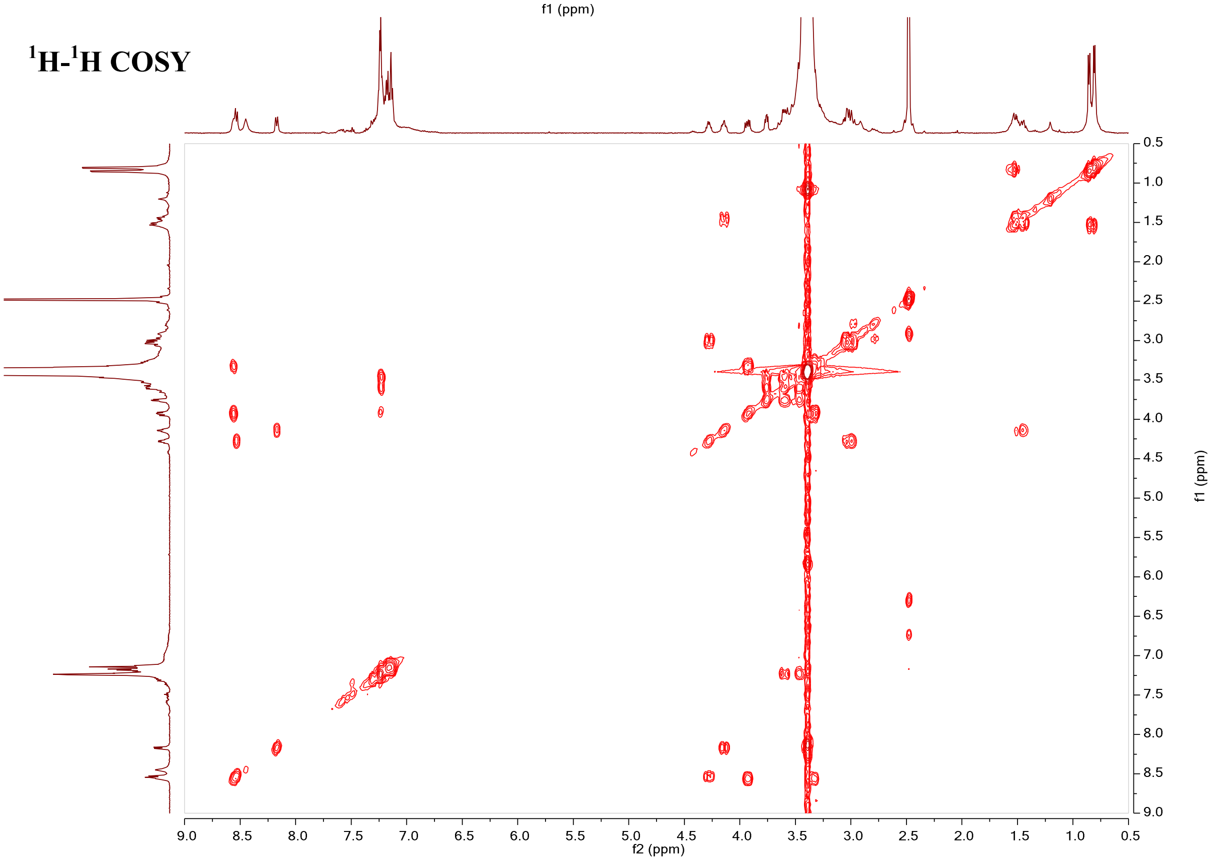
f2 (ppm)
f1 (ppm)

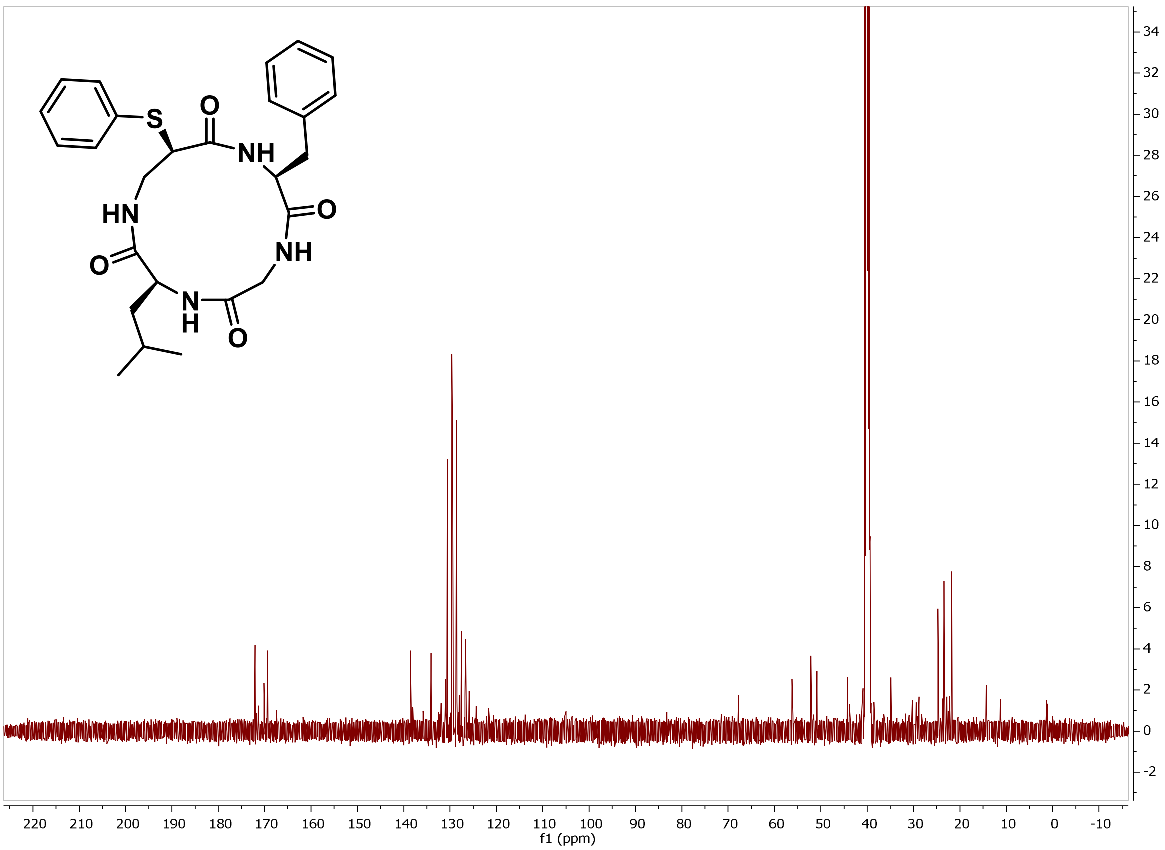

S
O
NH
HN
O
NH
O
N
H
O
f1 (ppm)

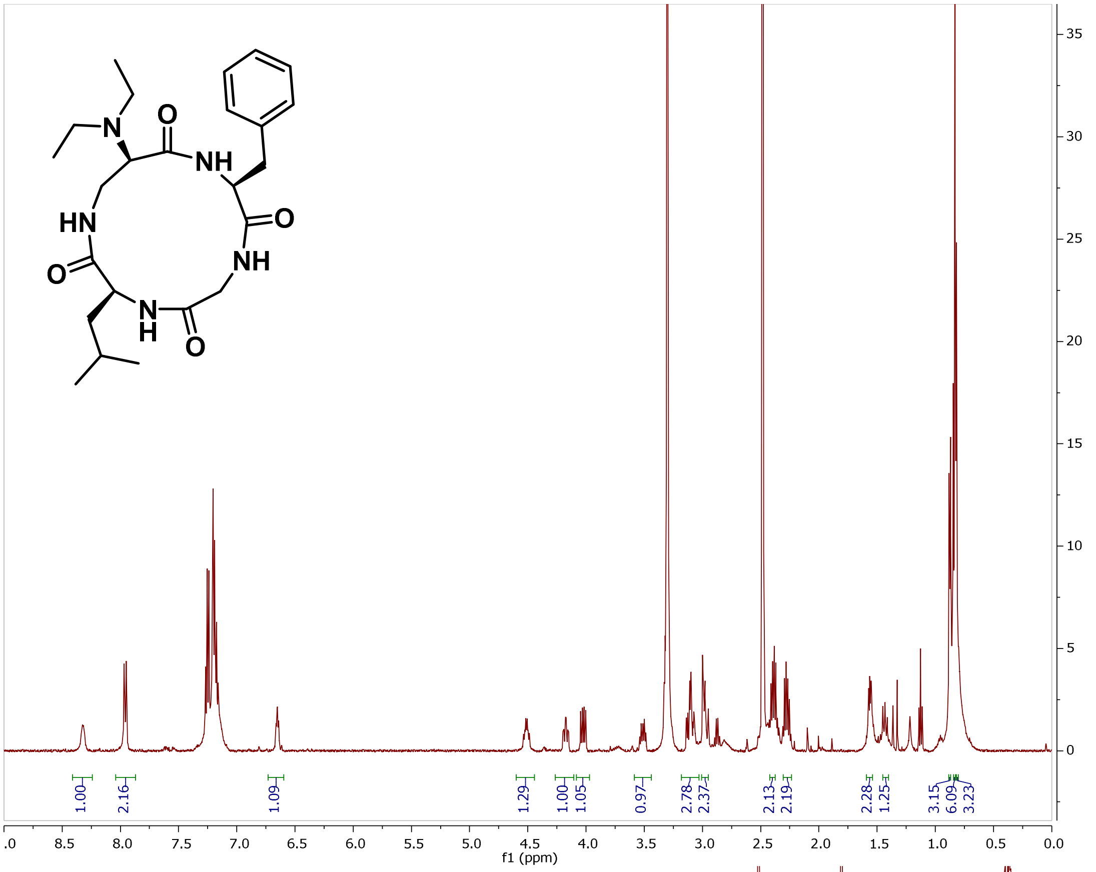
NH
O
N
HN
O
NH
O
N
H
O
1.00
2.16
1.09
1.29
1.00
1.05
0.97
2.78
2.37
2.13
2.19
2.28
1.25
3.15
6.09
3.23
f1 (ppm)

^{1}H-^{1}H COSY

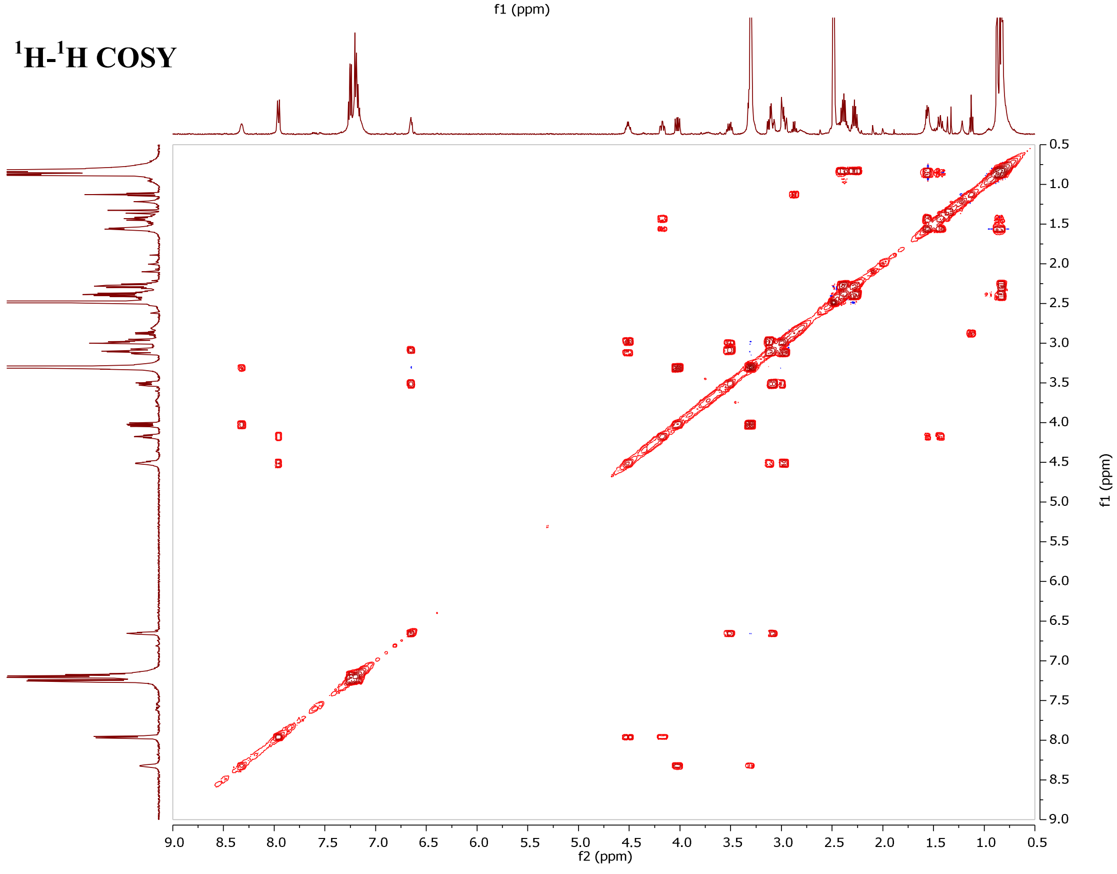
f2 (ppm)
f1 (ppm)

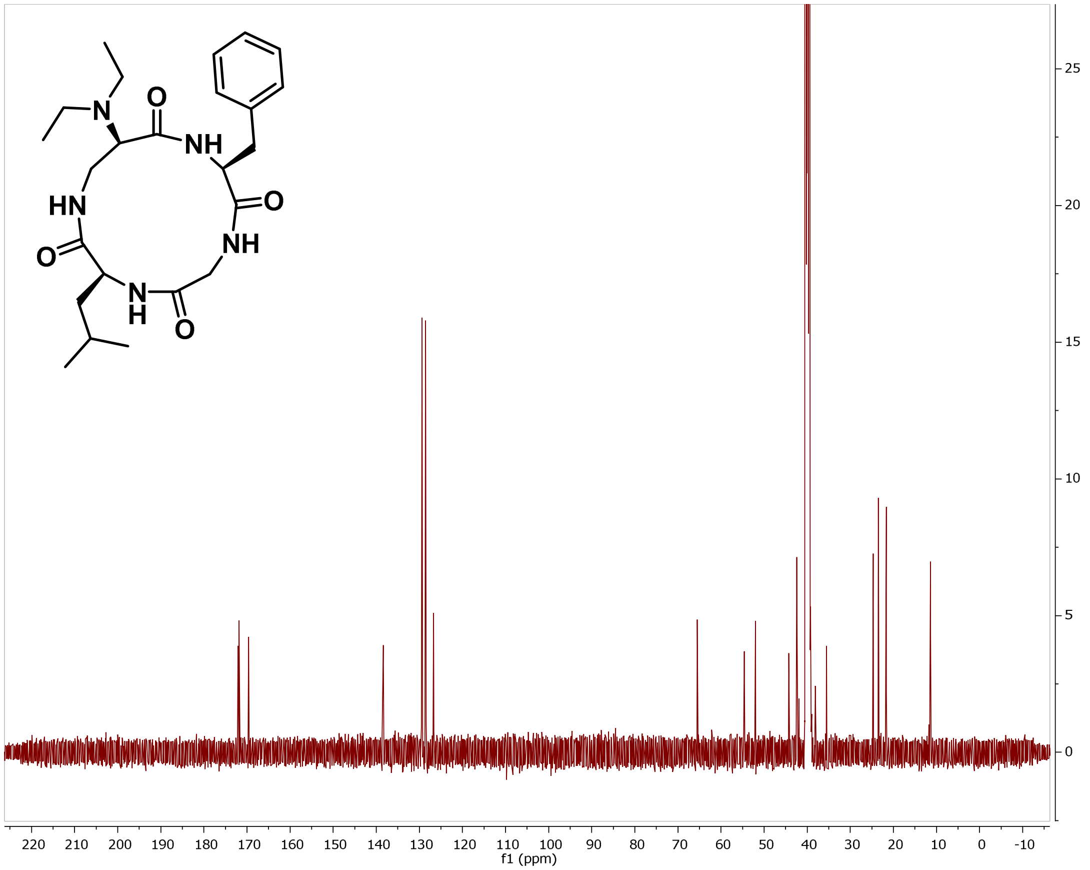

O
NH
O
NH
N
HN
O
N
H
O
25
20
15
10
5
0
220 210 200 190 180 170 160 150 140 130 120 110 100 90 80 70 60 50 40 30 20 10 0 -10
f1 (ppm)

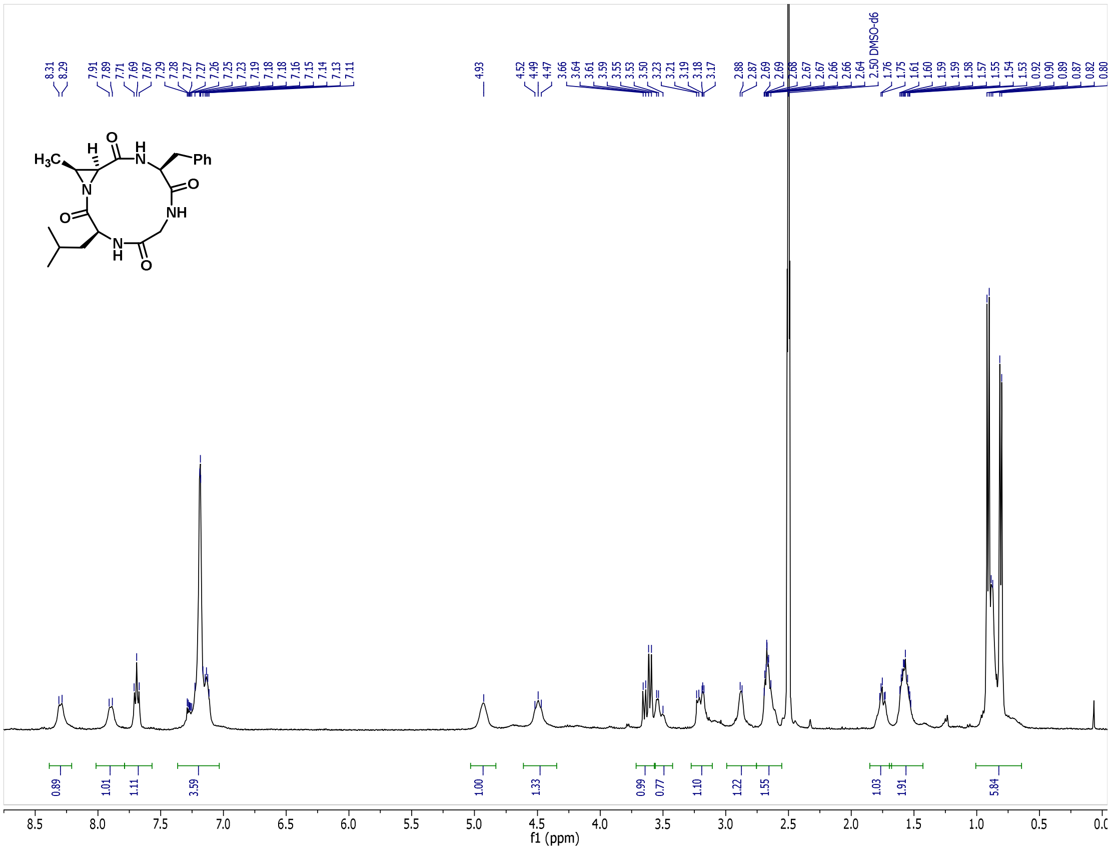
8.31 8.29 7.91 7.89 7.71 7.69 7.67 7.29 7.28 7.27 7.27 7.26 7.25 7.23 7.19 7.18 7.18 7.16 7.15 7.14 7.13 7.11
4.93
4.52 4.49 4.47 3.66 3.64 3.61 3.59 3.55 3.53 3.50 3.23 3.21 3.19 3.18 3.17
2.88 2.87 2.69 2.69 2.68 2.67 2.67 2.66 2.66 2.64 2.50 DMSO-d6 1.76 1.75 1.61 1.60 1.59 1.59 1.58 1.57 1.55 1.54 1.53 0.92 0.90 0.89 0.87 0.82 0.80
H3C
H
O
N
Ph
NH
0.89 1.01 1.11 3.59 1.00 1.33 0.99 0.77 1.10 1.22 1.55 1.03 1.91 5.84
f1 (ppm)

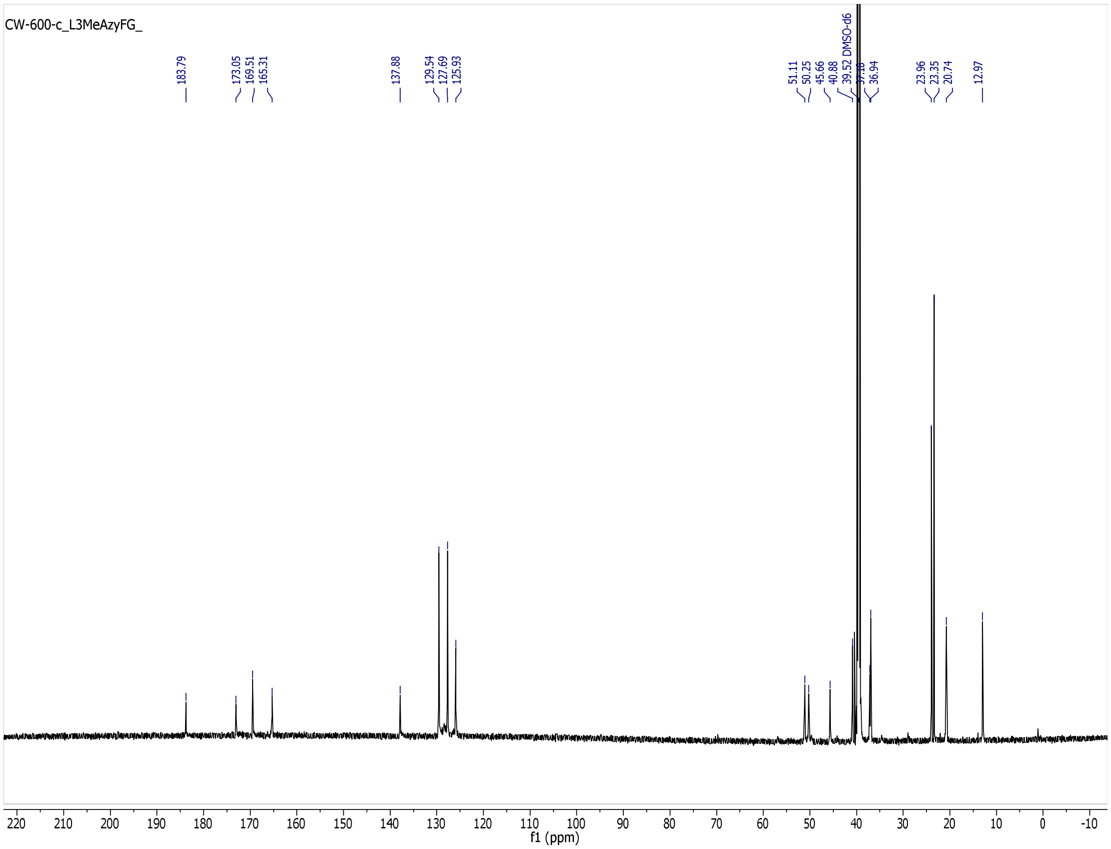
CW-600-c_L3MeAzyFG_
183.79
173.05 169.51 165.31
137.88
129.54 127.69 125.93
51.11 50.25 45.66 40.88 39.52 DMSO-d6 36.94
23.96 23.35 20.74
12.97
f1 (ppm)

Compound 2.28 – COSY spectrum

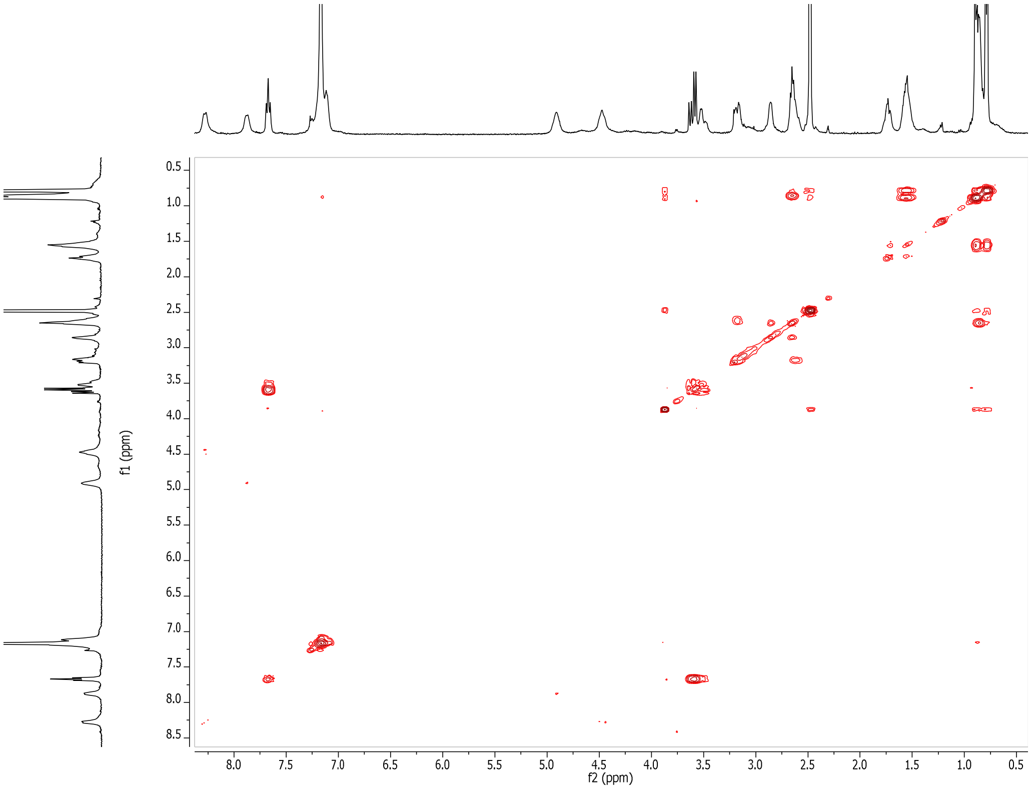

Compound 2.28 – TOCSY spectrum

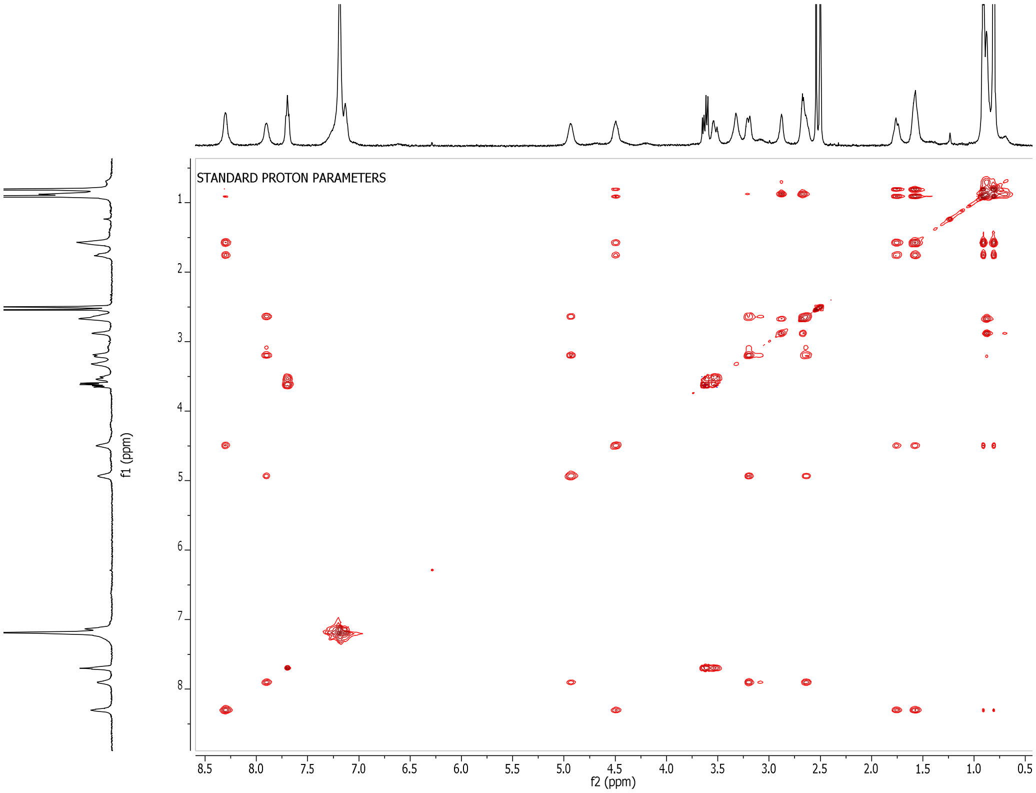

Compound 2.28 – NOESY spectrum

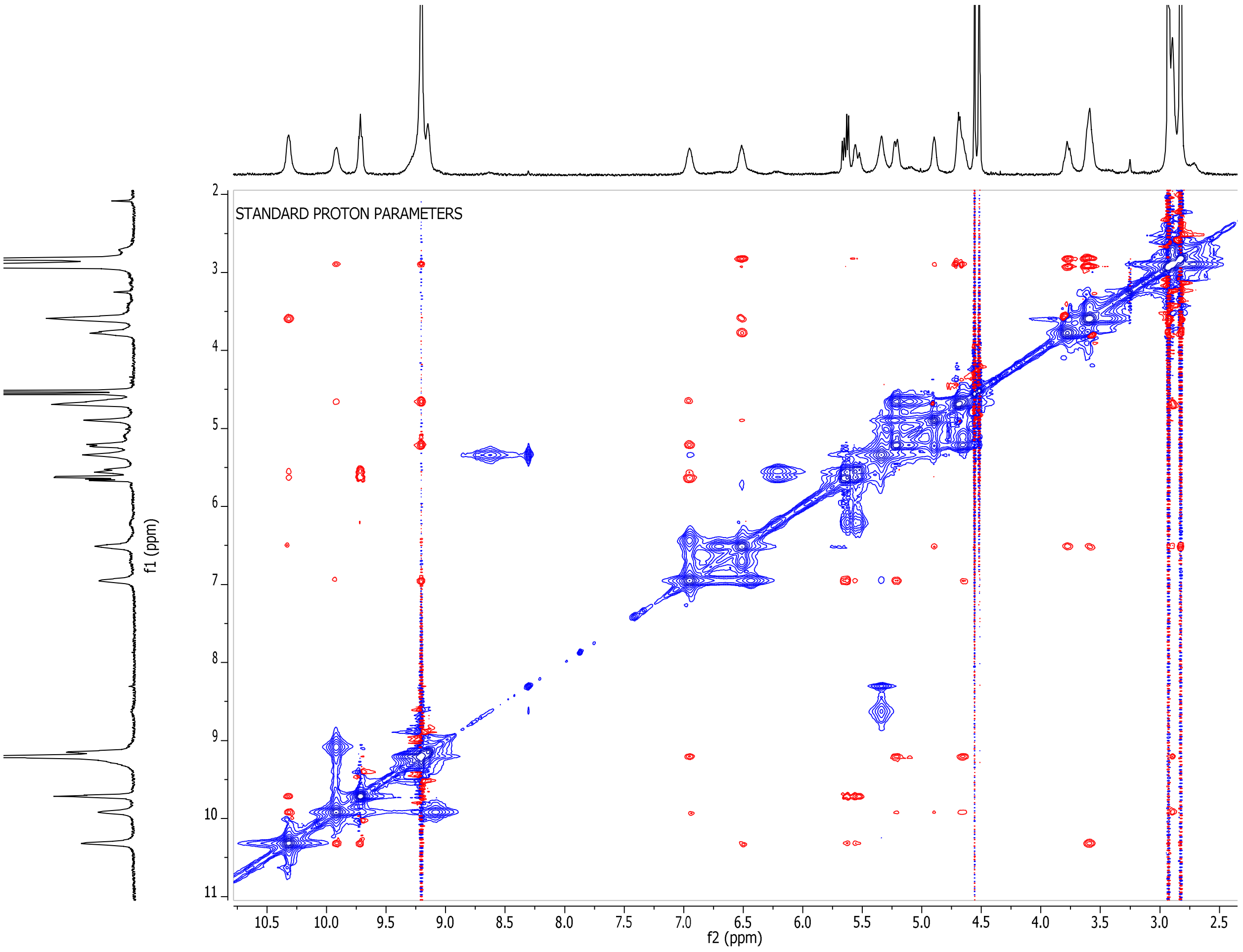

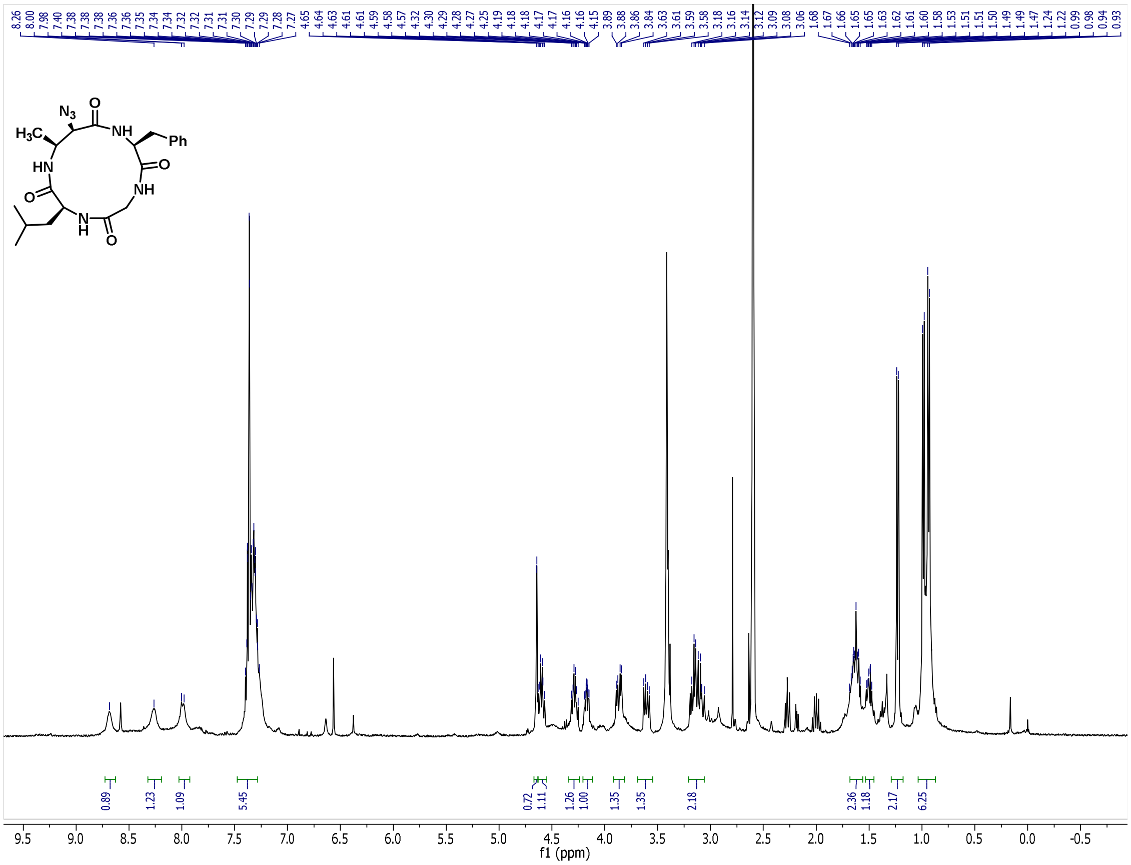
8.26 8.00 7.98 7.40 7.38 7.38 7.38 7.36 7.36 7.35 7.34 7.34 7.32 7.32 7.31 7.31 7.30 7.29 7.29 7.29 7.28 7.27 4.65 4.64 4.63 4.61 4.61 4.59 4.58 4.57 4.32 4.30 4.29 4.28 4.27 4.25 4.19 4.18 4.18 4.17 4.17 4.16 4.16 4.15 3.89 3.88 3.86 3.84 3.63 3.61 3.59 3.58 3.18 3.16 3.14 3.12 3.09 3.08 3.06 1.68 1.67 1.66 1.65 1.65 1.63 1.62 1.61 1.60 1.58 1.53 1.51 1.51 1.50 1.49 1.49 1.47 1.24 1.22 0.99 0.98 0.94 0.93
N_3
O
H_3C
NH
Ph
HN
O
NH
O
N
H
O
0.89 1.23 1.09 5.45 0.72 1.11 1.26 1.00 1.35 1.35 2.18 2.36 1.18 2.17 6.25
9.5 9.0 8.5 8.0 7.5 7.0 6.5 6.0 5.5 5.0 4.5 4.0 3.5 3.0 2.5 2.0 1.5 1.0 0.5 0.0 -0.5
f1 (ppm)

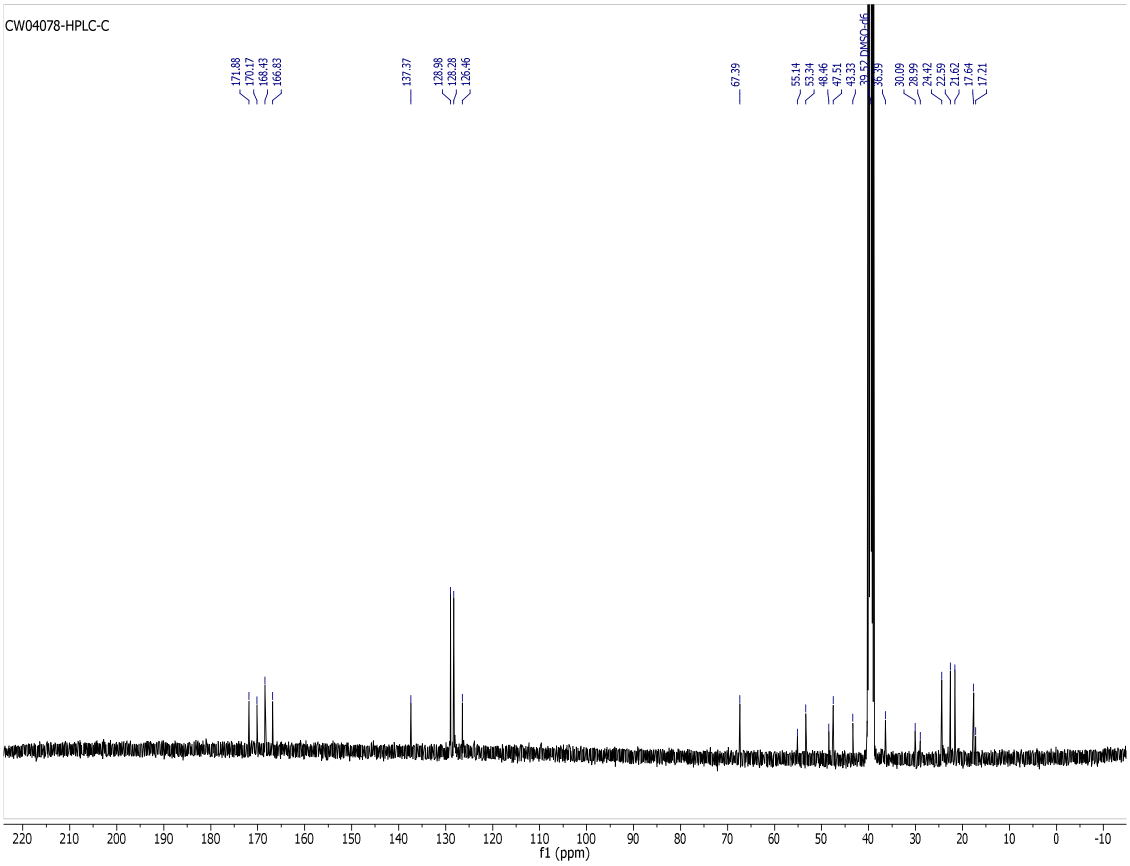
CW04078-HPLC-C
171.88 170.17 168.43 166.83 137.37 128.98 128.28 126.46 67.39 55.14 53.34 48.46 47.51 43.33 39.52 DMSO-d6 36.39 30.09 28.99 24.42 22.59 21.62 17.64 17.21
220 210 200 190 180 170 160 150 140 130 120 110 100 90 80 70 60 50 40 30 20 10 0 -10
f1 (ppm)

COSY spectrum

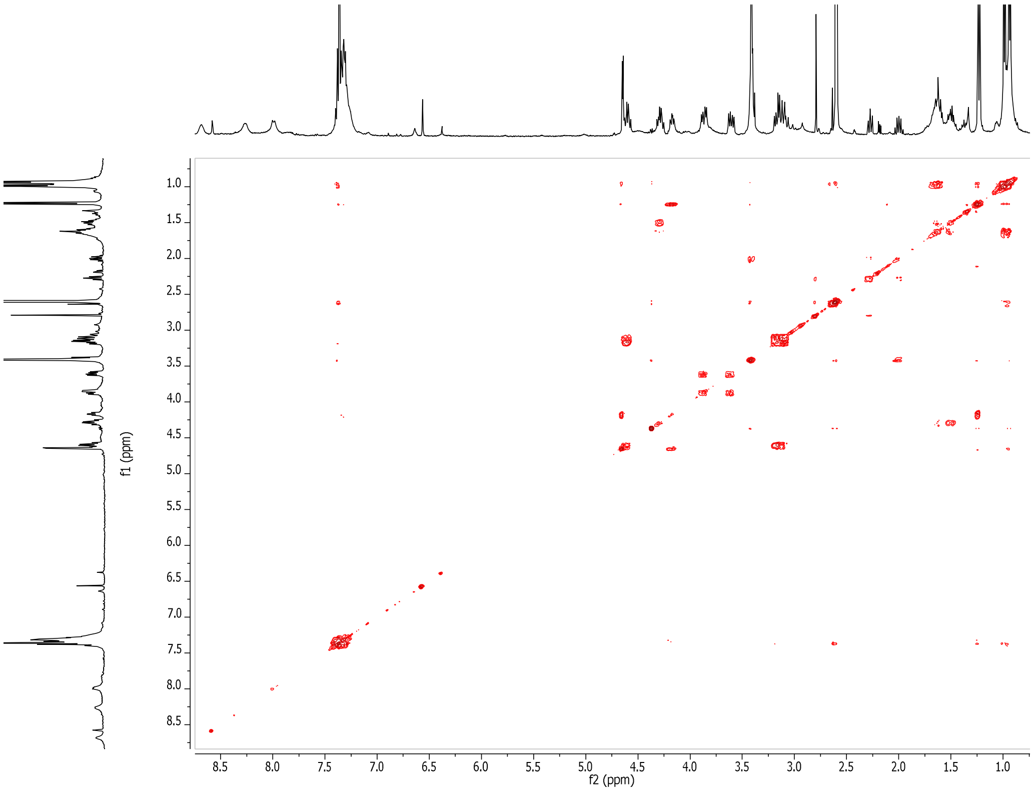

TOCSY spectrum

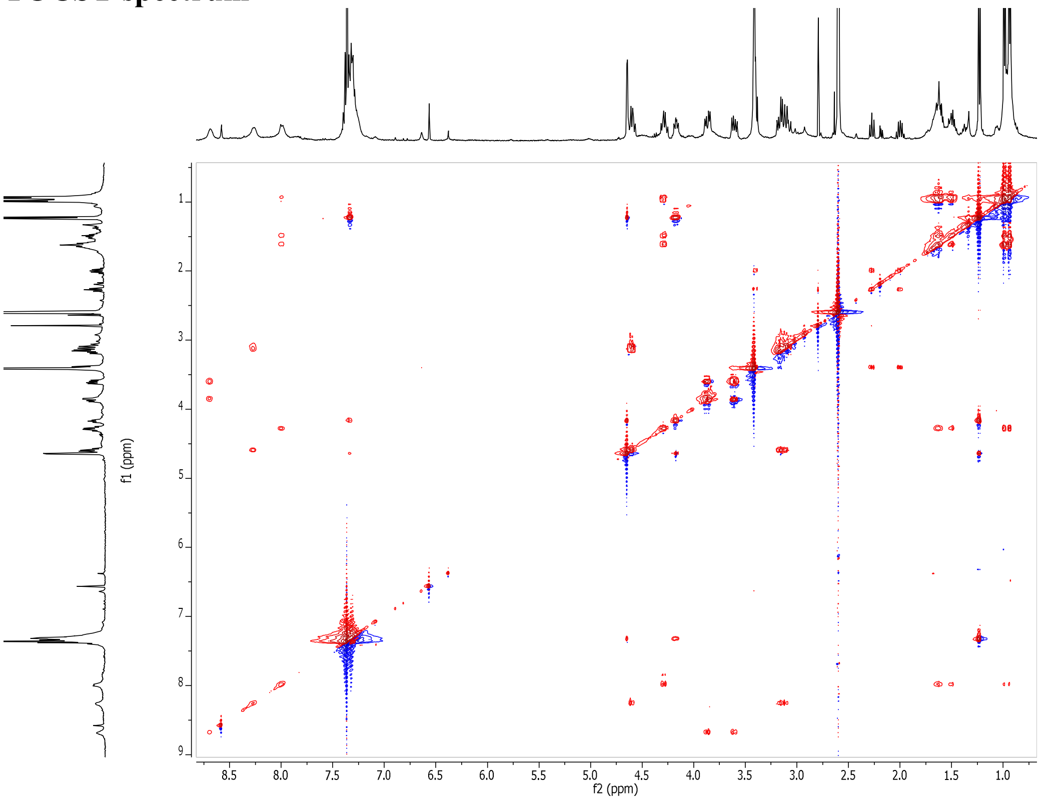

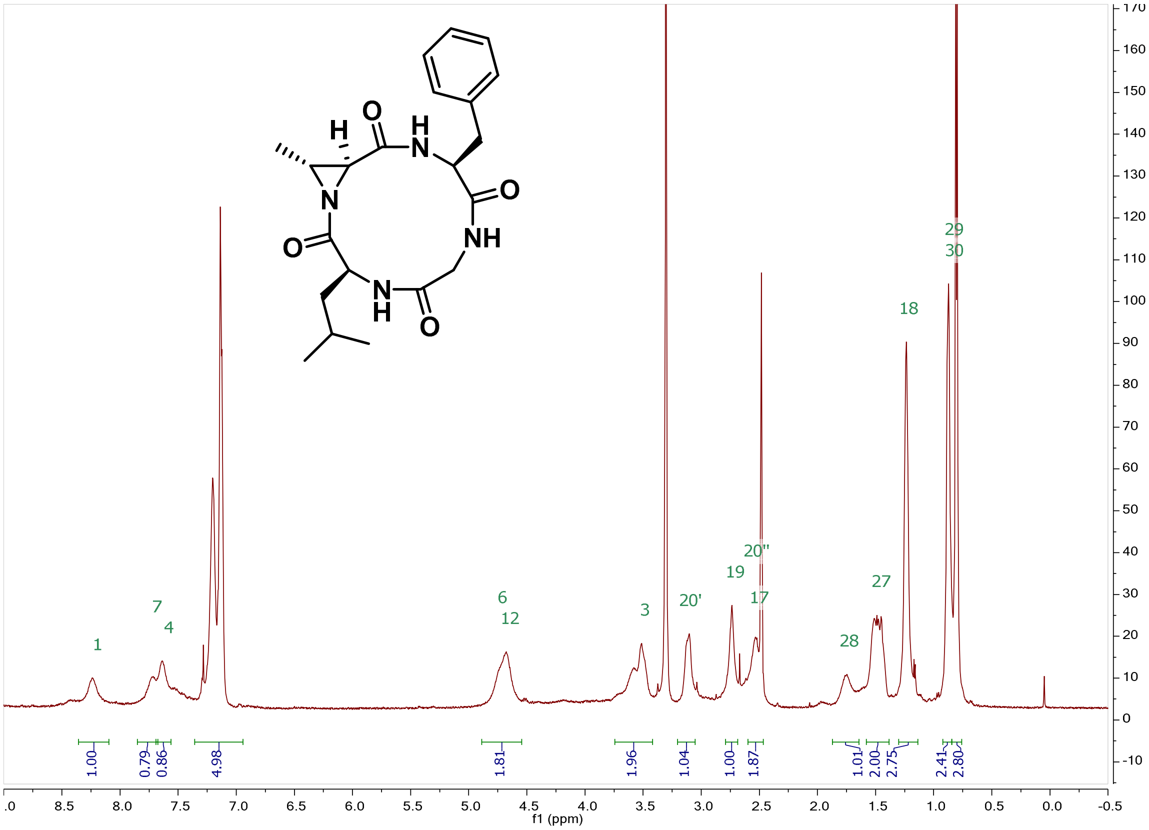

1
7
4
6
12
3
20'
19
20''
17
28
27
18
29
30
1.00
0.79
0.86
4.98
1.81
1.96
1.04
1.00
1.87
1.01
2.00
2.75
2.41
2.80
f1 (ppm)

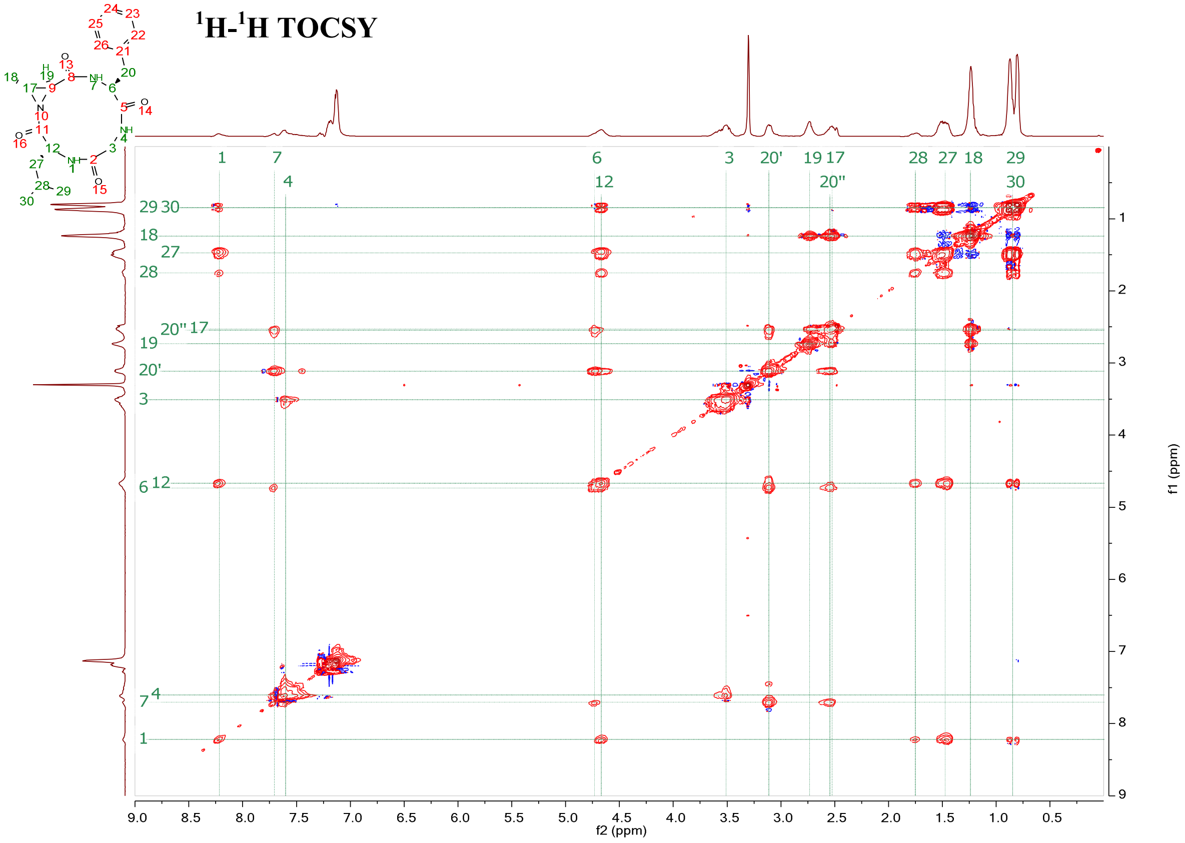

^{1}H-^{1}H TOCSY
f2 (ppm)
f1 (ppm)

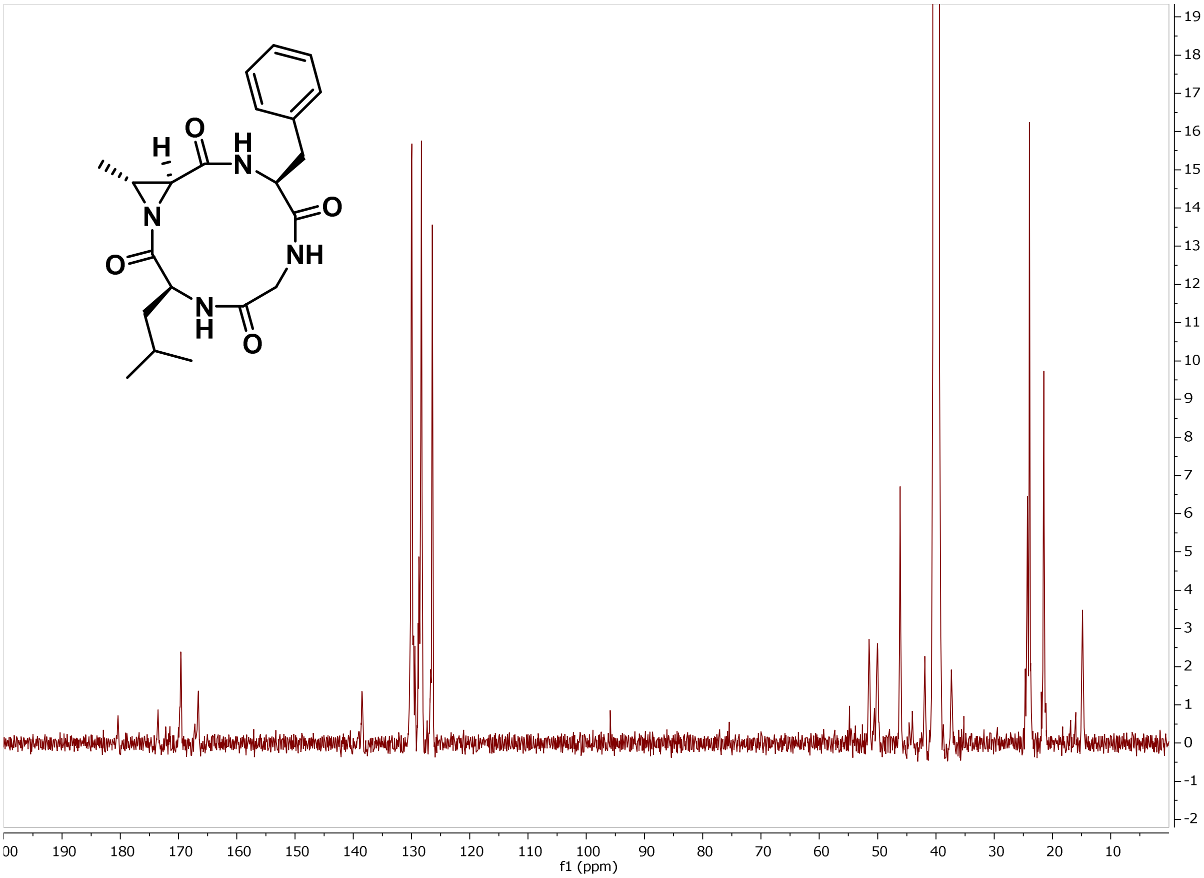

f1 (ppm)

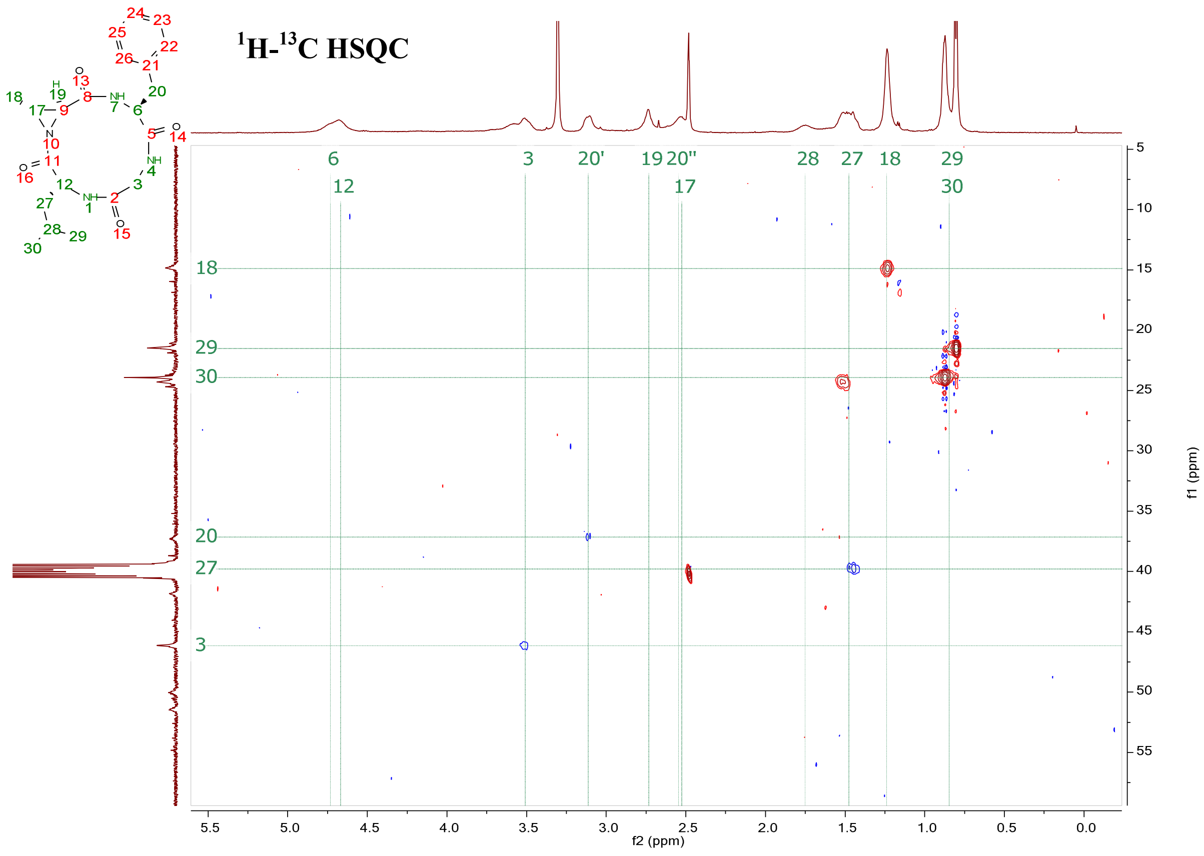

1H-13C HSQC
6
12
3
20'
19
20"
17
28
27
18
29
30
f2 (ppm)
f1 (ppm)

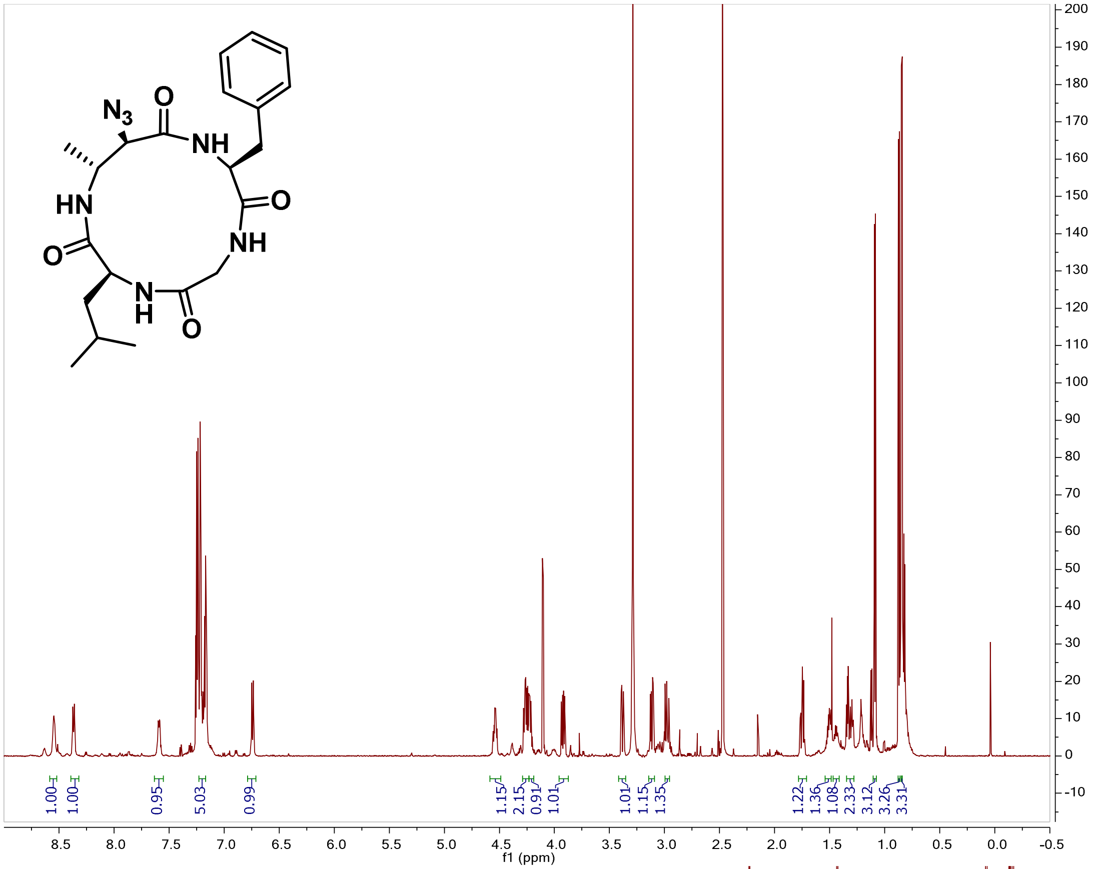

^{1}H-^{1}H TOCSY

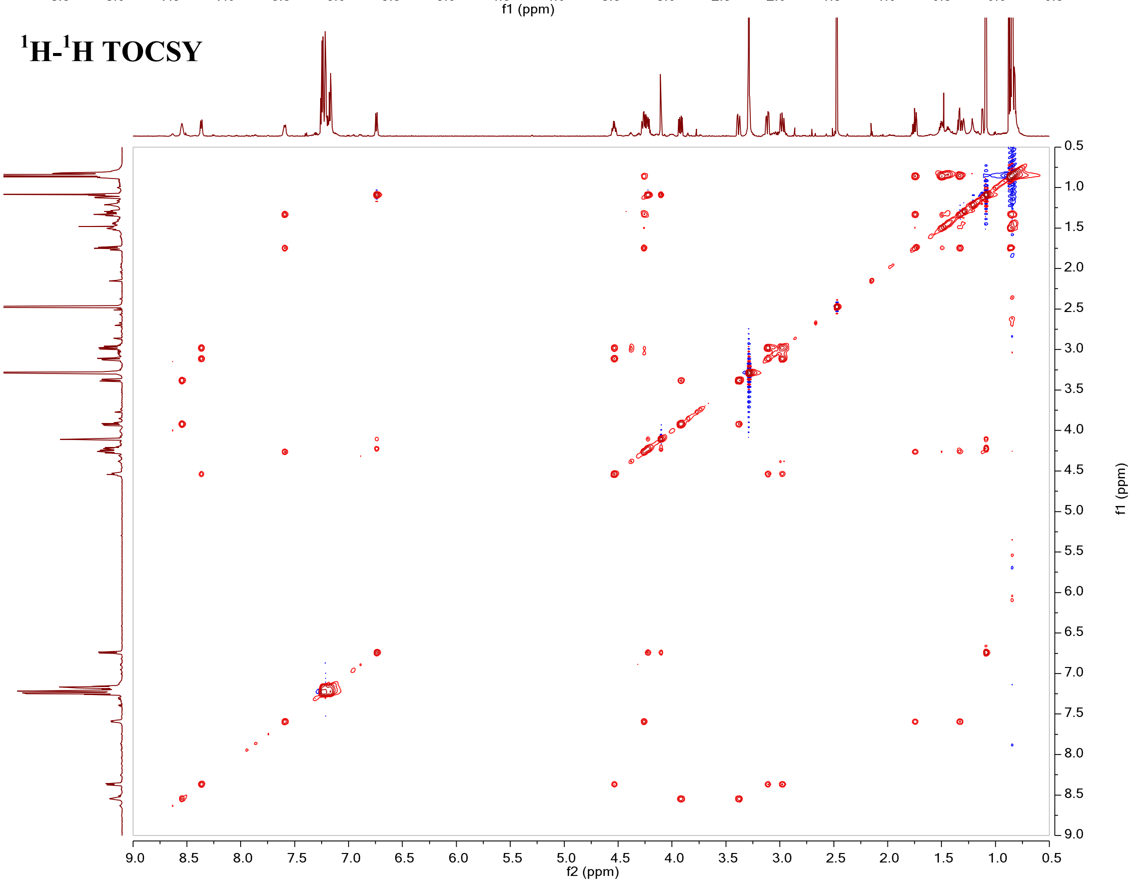

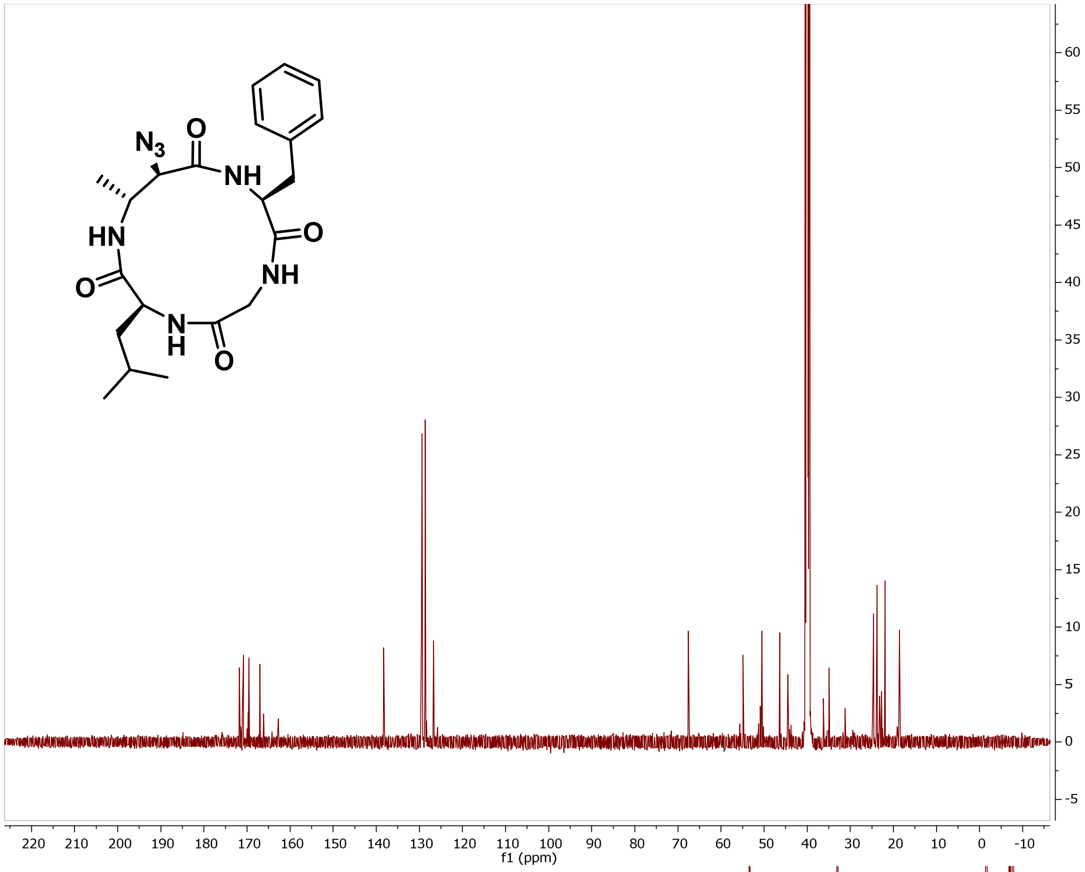

^{1}H-^{13}C HSQC

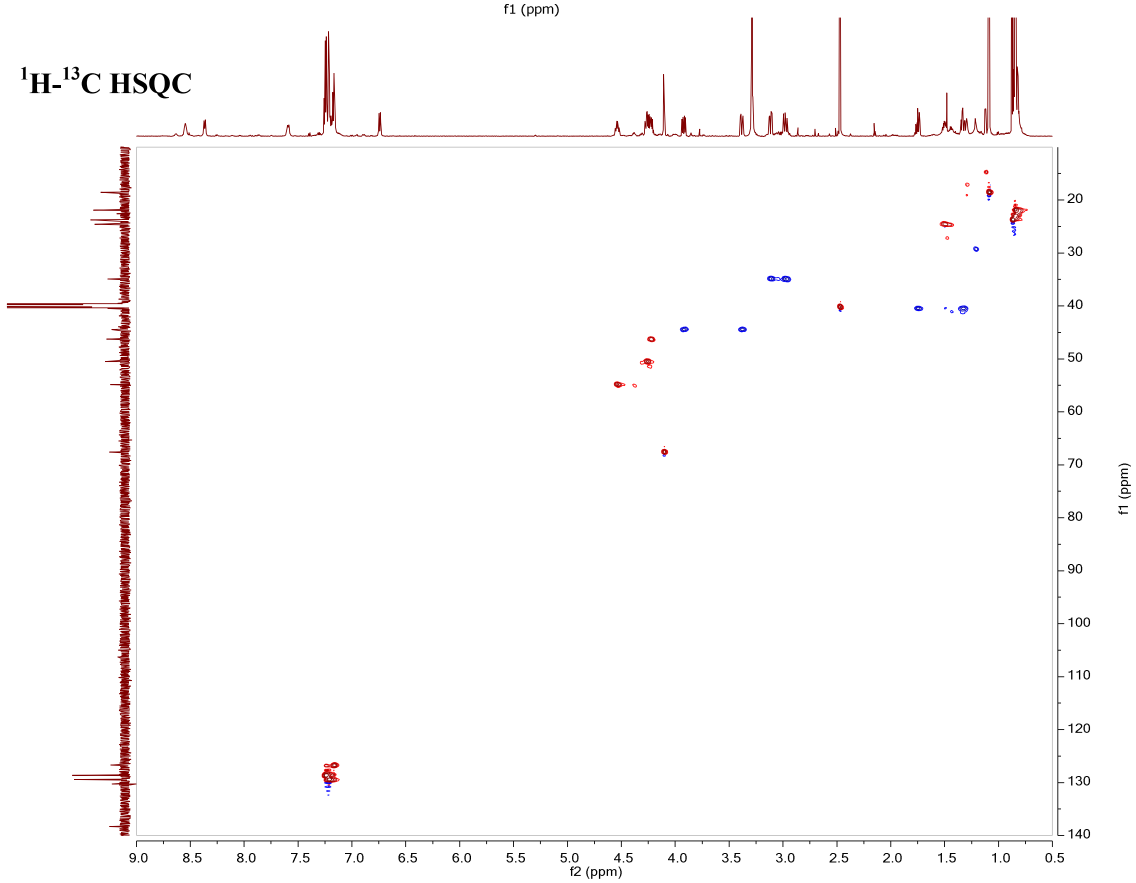

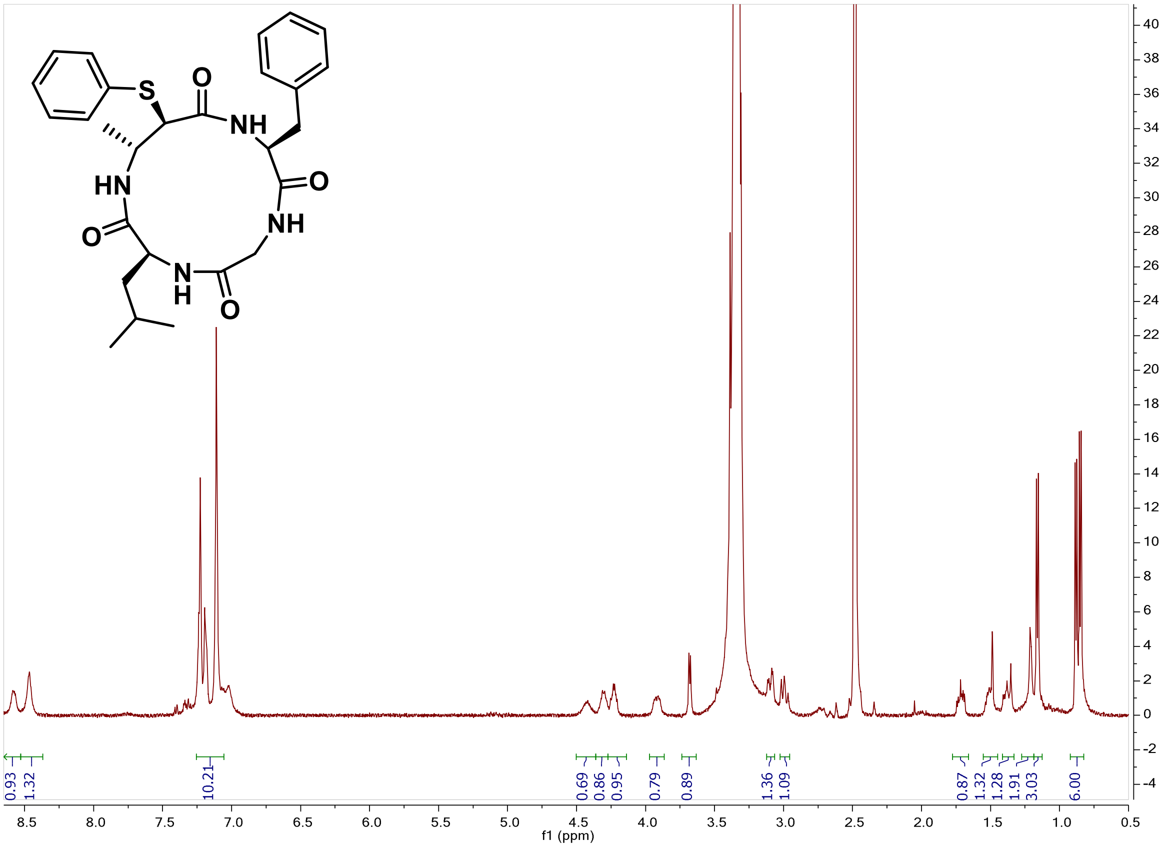

[1]H-^{1}H TOCSY

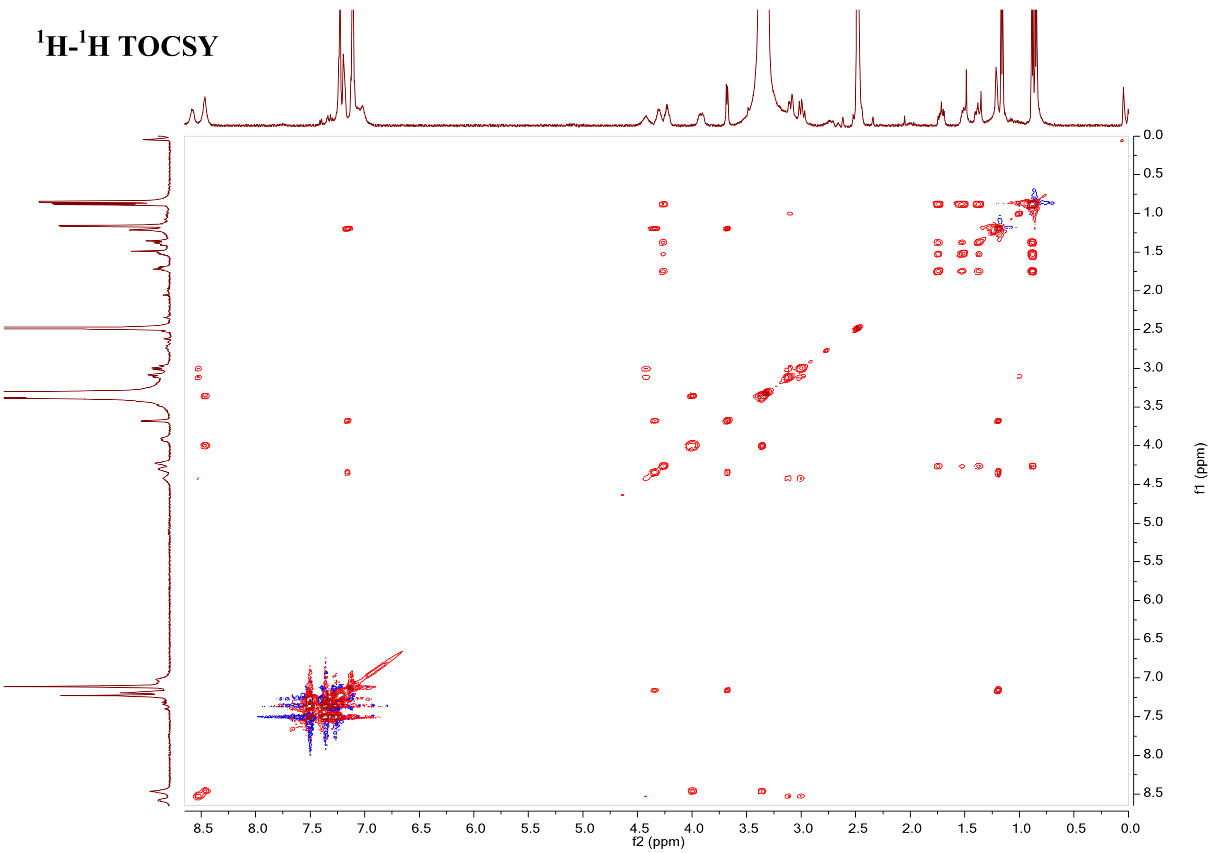

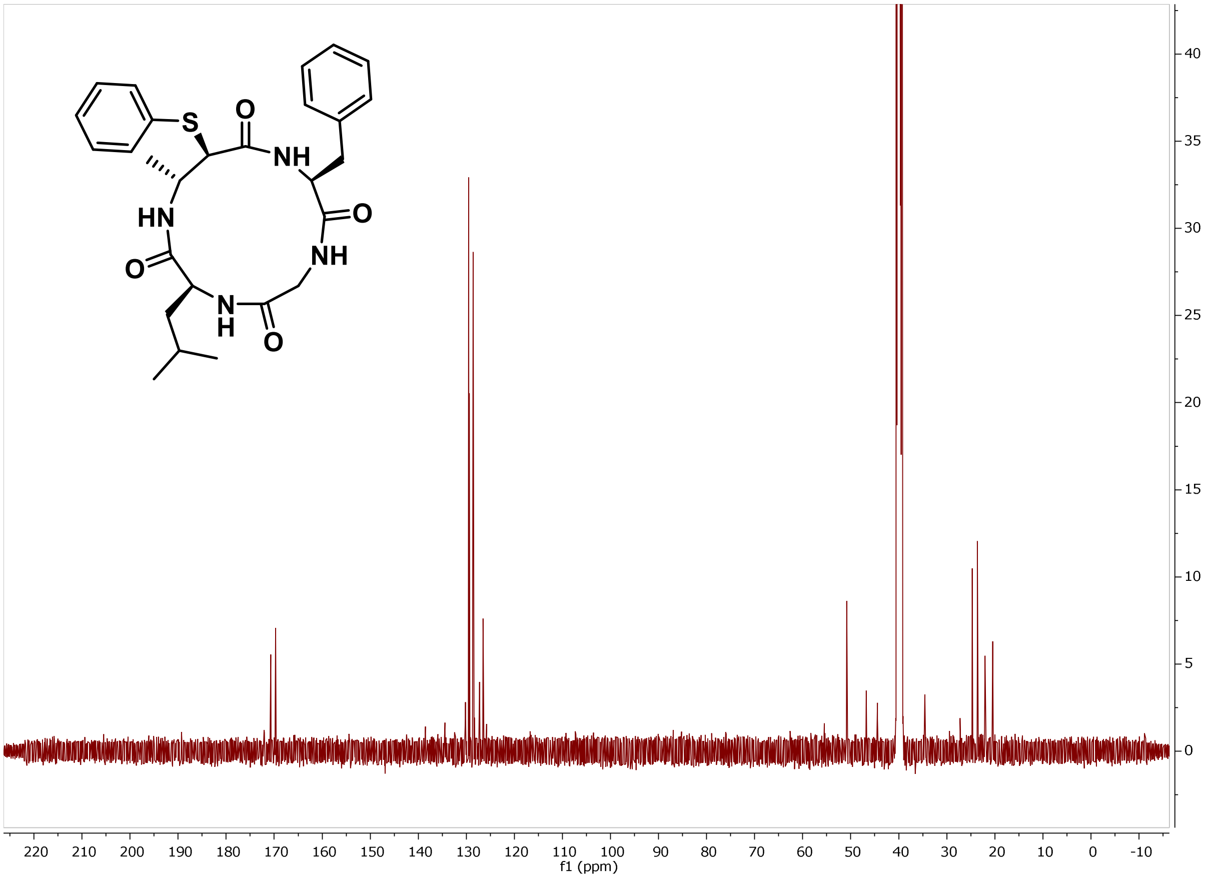
S
O
NH
O
HN
O
NH
N
H
O
220 210 200 190 180 170 160 150 140 130 120 110 100 90 80 70 60 50 40 30 20 10 0 -10
f1 (ppm)
40 35 30 25 20 15 10 5 0

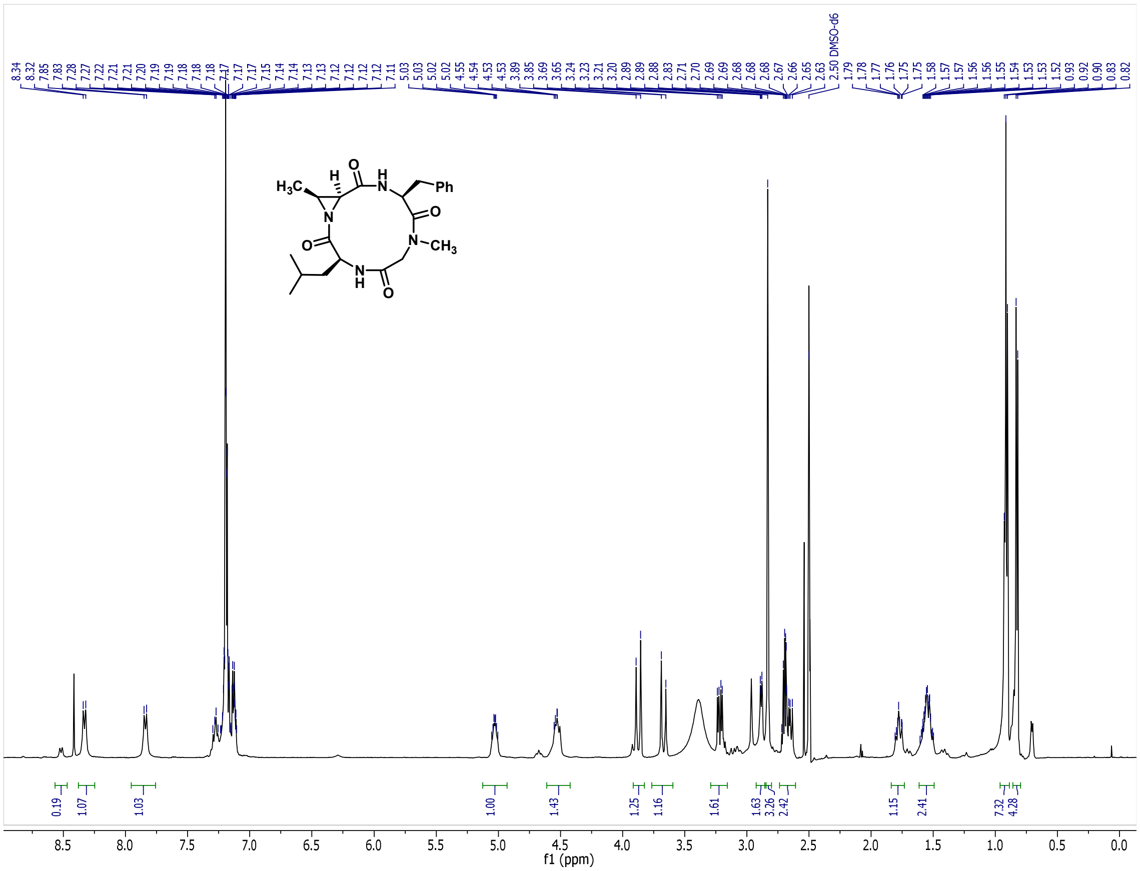
8.34 8.32 7.85 7.83 7.28 7.27 7.22 7.21 7.21 7.20 7.19 7.19 7.18 7.18 7.18 7.17 7.17 7.15 7.14 7.14 7.13 7.13 7.12 7.12 7.12 7.11 5.03 5.03 5.02 5.02 4.55 4.54 4.53 4.53 3.89 3.85 3.69 3.65 3.24 3.23 3.21 3.20 2.89 2.89 2.88 2.83 2.71 2.70 2.69 2.69 2.68 2.68 2.67 2.66 2.65 2.63
2.50 DMSO-d6
1.79 1.78 1.77 1.76 1.75 1.75 1.58 1.57 1.57 1.56 1.56 1.55 1.54 1.53 1.53 1.52 0.93 0.92 0.90 0.83 0.82
H
H₃C
O
N
H
Ph
O
N
N
CH₃
O
N
H
O
0.19 1.07 1.03 1.00 1.43 1.25 1.16 1.61 1.63 3.26 2.42 1.15 2.41 7.32 4.28
8.5 8.0 7.5 7.0 6.5 6.0 5.5 5.0 4.5 4.0 3.5 3.0 2.5 2.0 1.5 1.0 0.5 0.0
f1 (ppm)

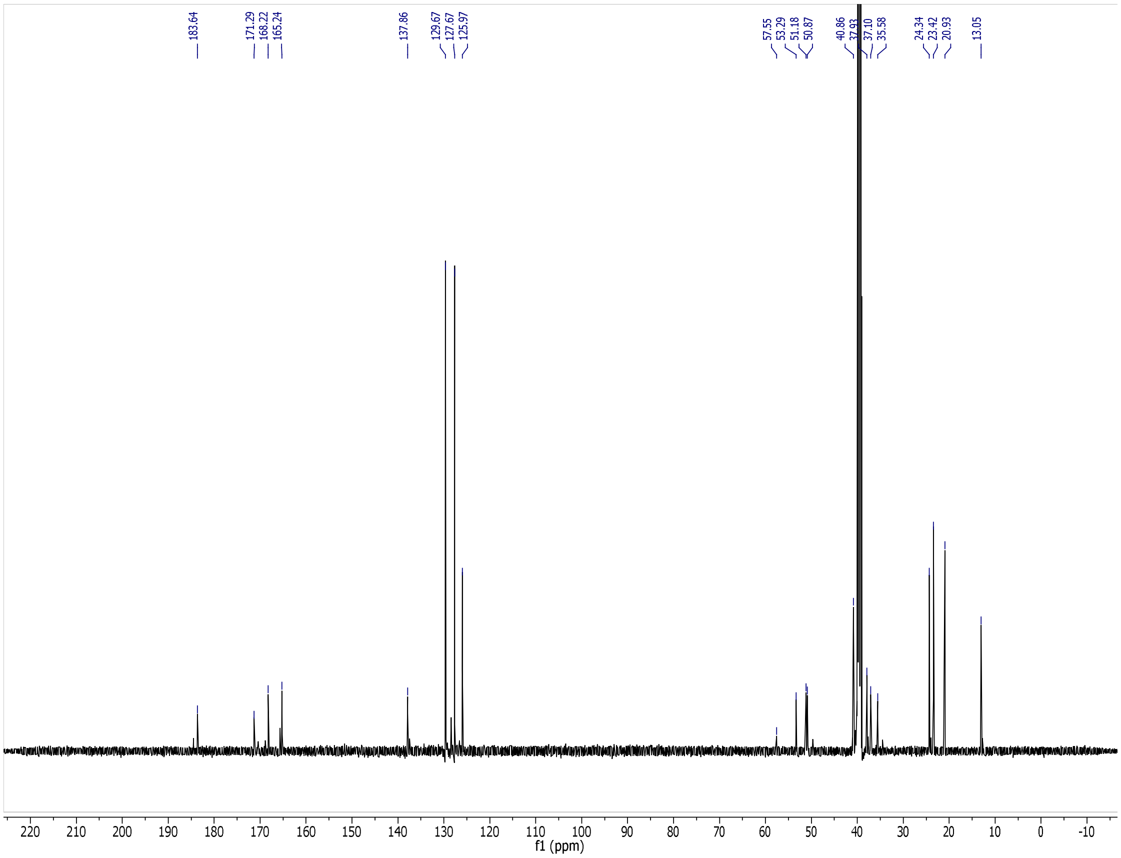
183.64
171.29 168.22 165.24
137.86
129.67 127.67 125.97
57.55 53.29 51.18 50.87
40.86 37.93 37.10 35.58
24.34 23.42 20.93
13.05
220 210 200 190 180 170 160 150 140 130 120 110 100 90 80 70 60 50 40 30 20 10 0 -10
f1 (ppm)

COSY spectrum

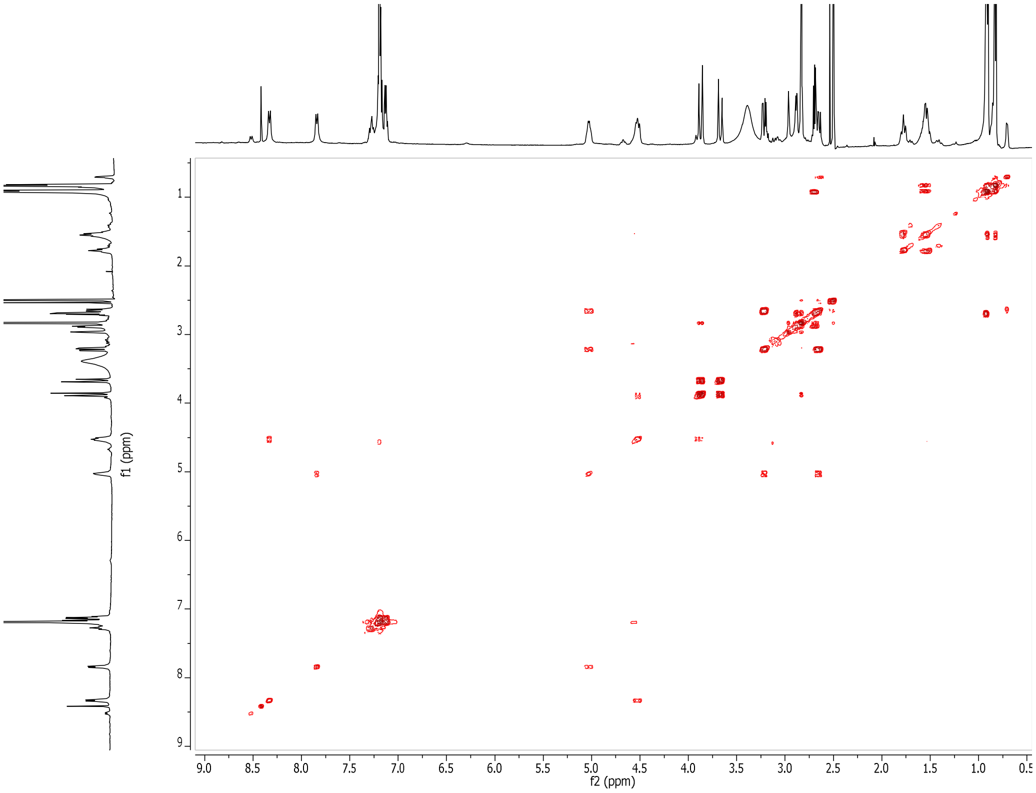

TOCSY spectrum

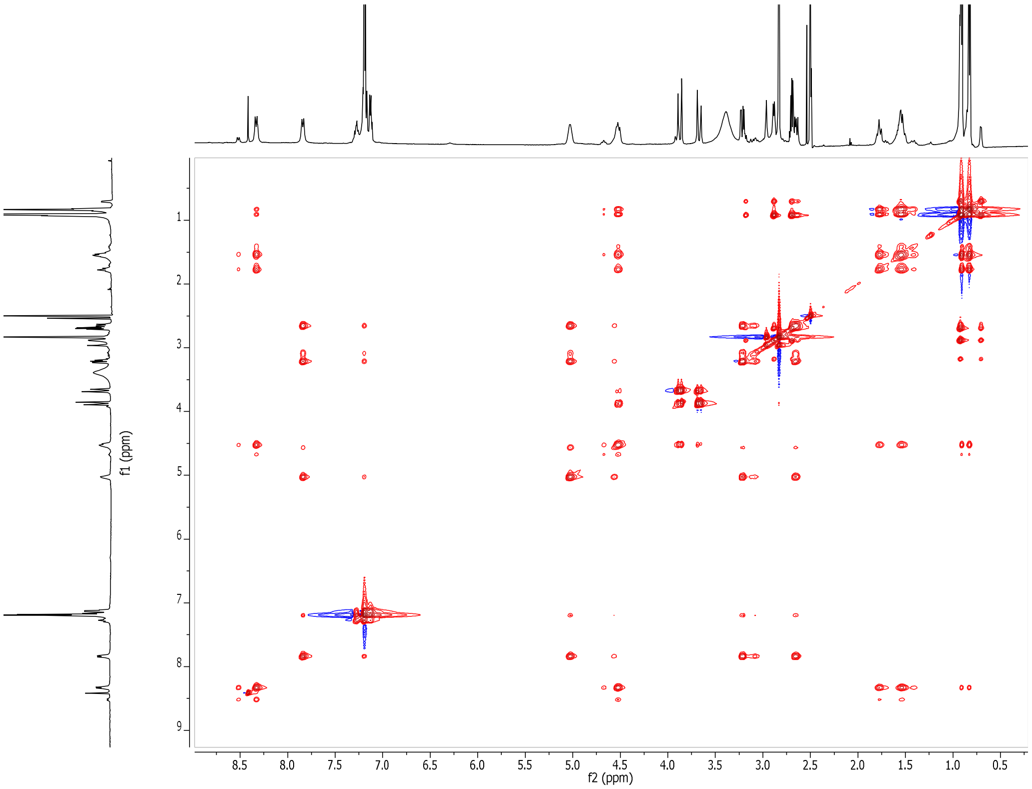

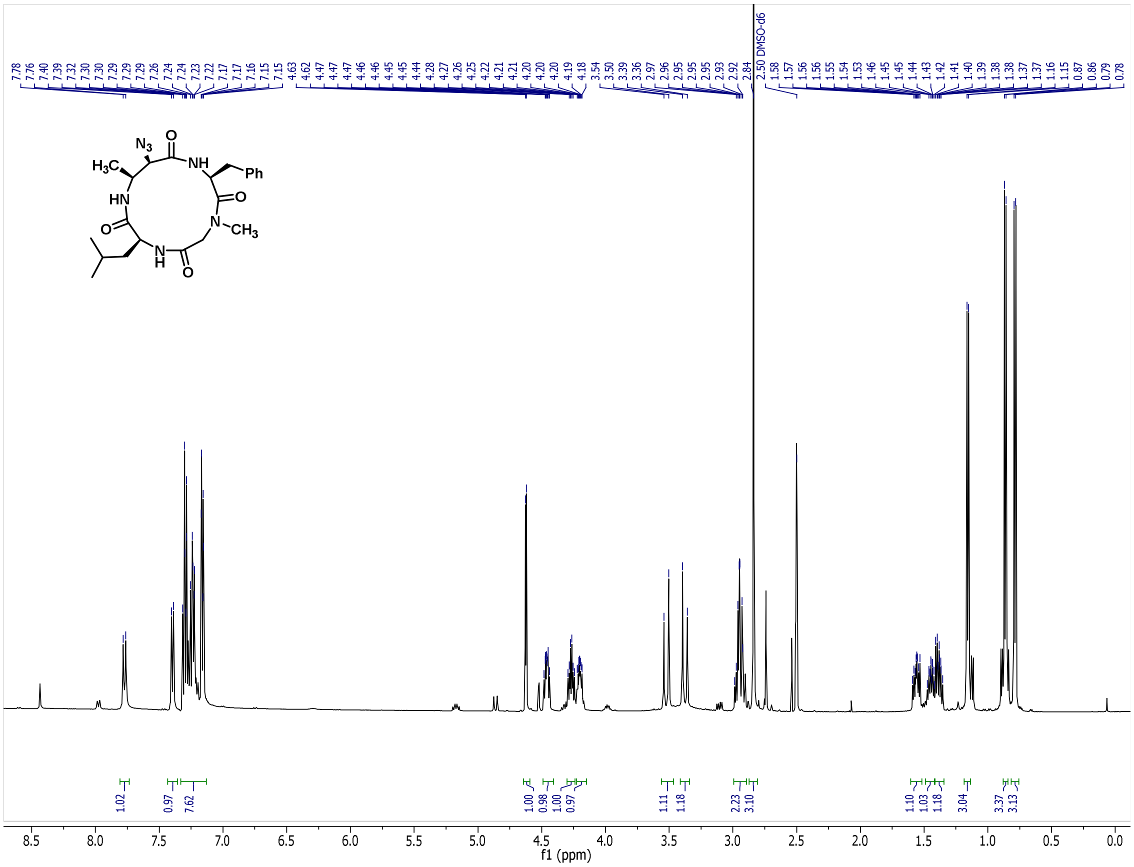
7.78 7.76 7.40 7.39 7.32 7.30 7.30 7.29 7.29 7.29 7.26 7.24 7.24 7.23 7.22 7.17 7.17 7.16 7.15 7.15 4.63 4.62 4.47 4.47 4.47 4.46 4.45 4.45 4.44 4.28 4.27 4.26 4.25 4.22 4.21 4.21 4.20 4.20 4.20 4.19 4.18 3.54 3.50 3.39 3.36 2.97 2.96 2.95 2.95 2.95 2.93 2.92 2.84
2.50 DMSO-d6
1.58 1.57 1.56 1.56 1.55 1.54 1.53 1.46 1.45 1.45 1.44 1.43 1.42 1.41 1.40 1.39 1.38 1.38 1.37 1.37 1.16 1.15 0.87 0.86 0.79 0.78
N3
O
H3C
NH
Ph
HN
O
N
CH3
O
N
H
O
1.02 0.97 7.62 1.00 0.98 1.00 0.97 1.11 1.18 2.23 3.10 1.10 1.03 1.18 3.04 3.37 3.13
8.5 8.0 7.5 7.0 6.5 6.0 5.5 5.0 4.5 4.0 3.5 3.0 2.5 2.0 1.5 1.0 0.5 0.0
f1 (ppm)

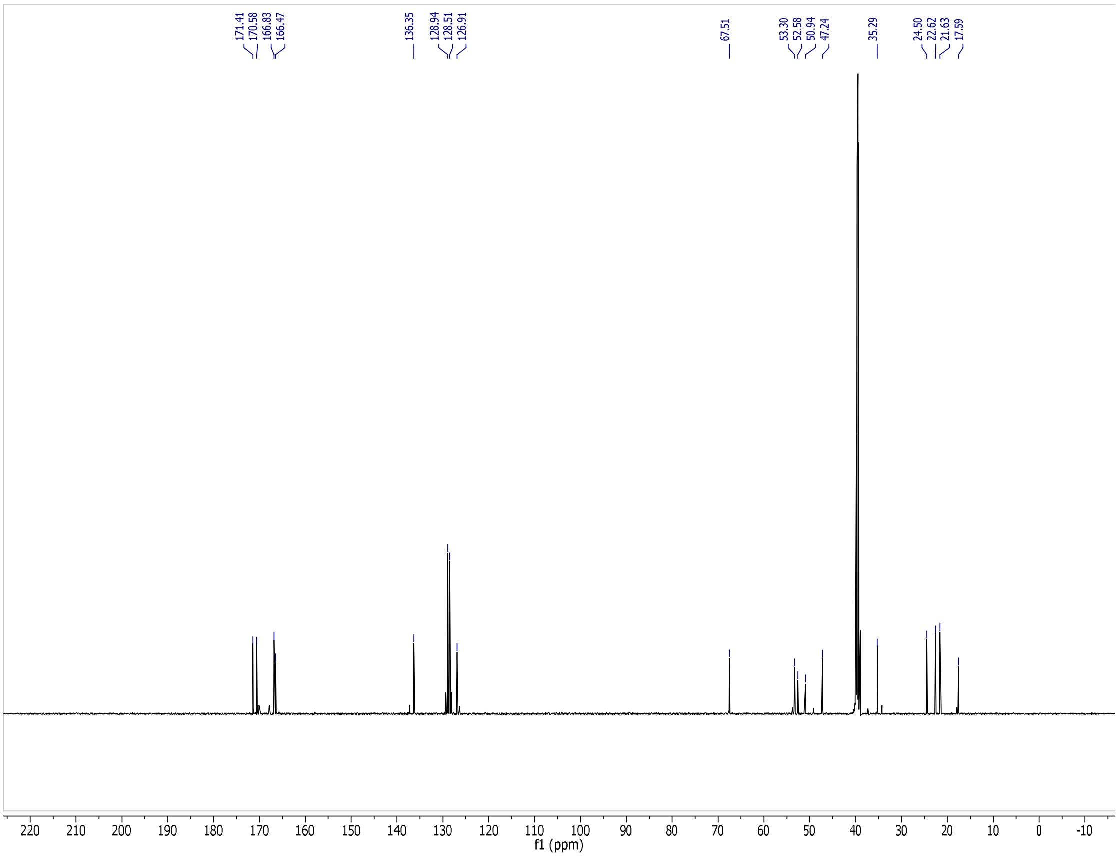
171.41 170.58 166.83 166.47
136.35 128.94 128.51 126.91
67.51
53.30 52.58 50.94 47.24
35.29
24.50 22.62 21.63 17.59
220 210 200 190 180 170 160 150 140 130 120 110 100 90 80 70 60 50 40 30 20 10 0 -10
f1 (ppm)

COSY spectrum

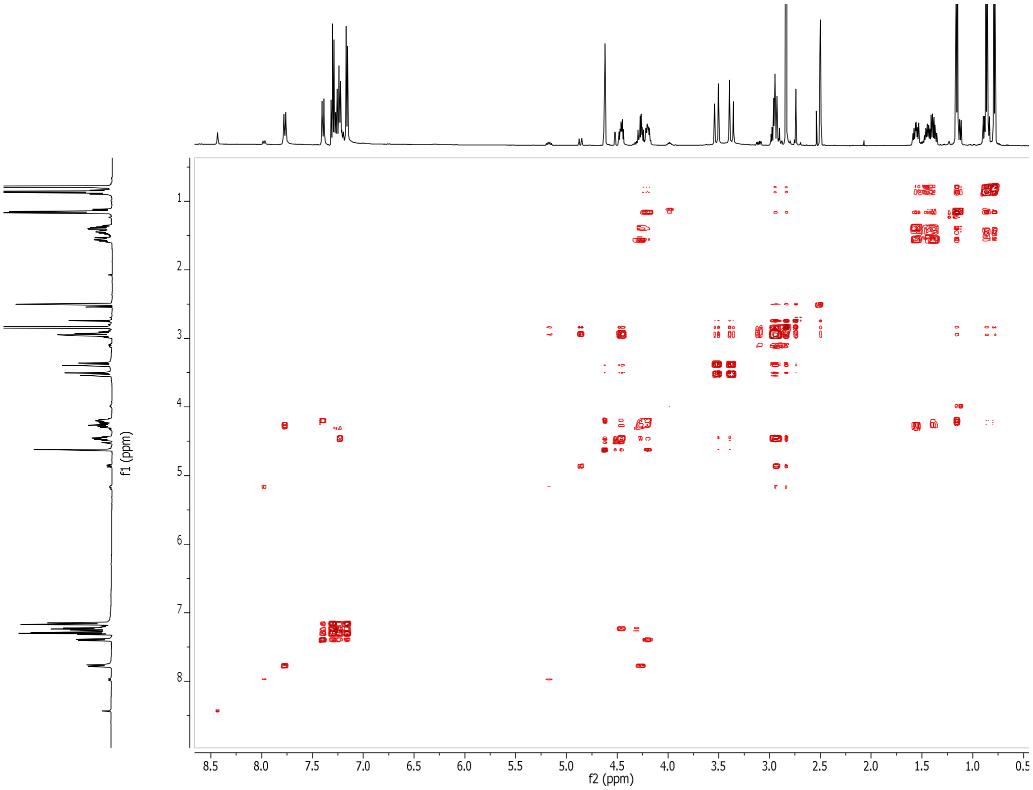

TOCSY spectrum

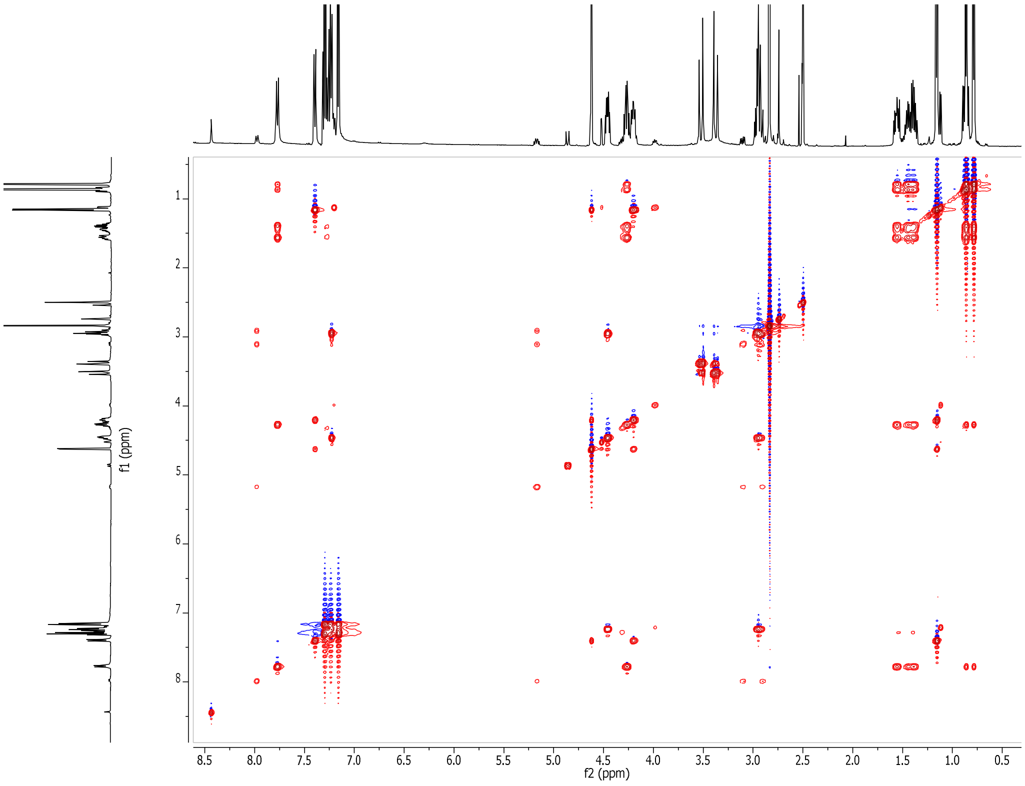

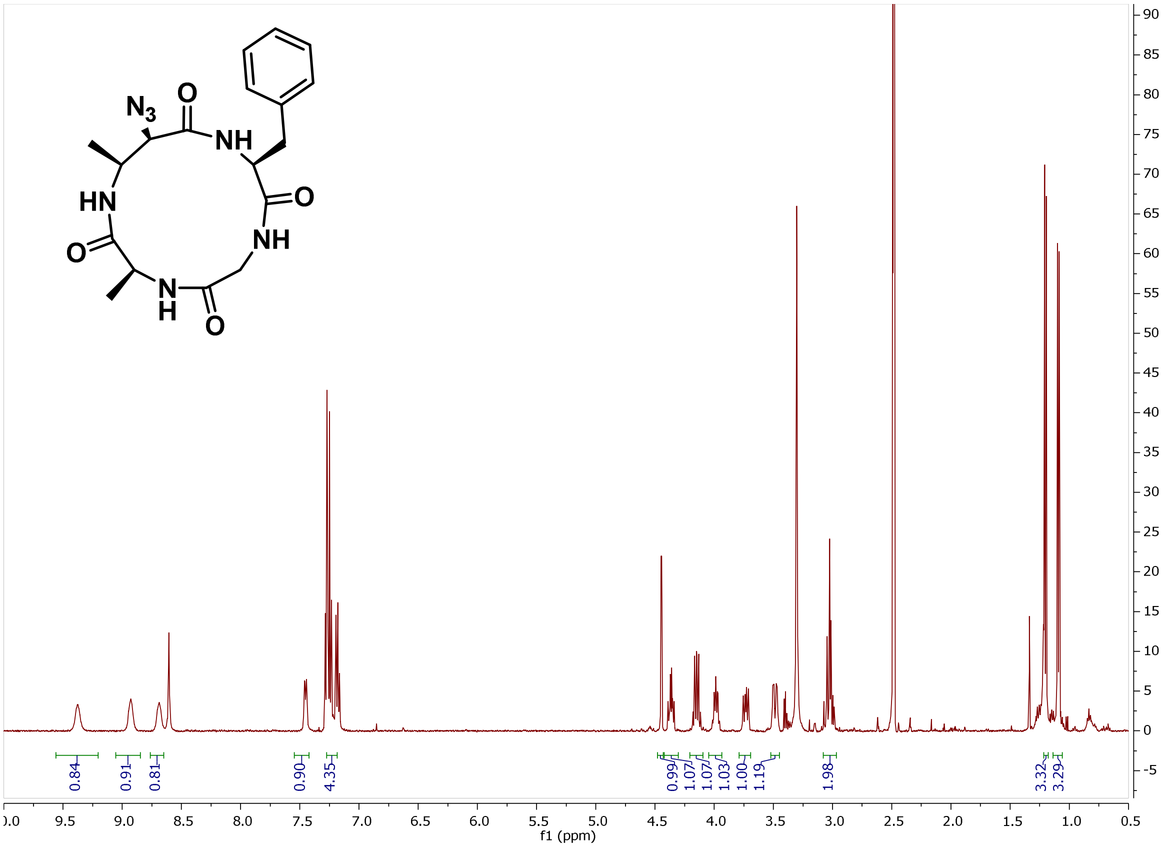

N_3
O
NH
O
HN
NH
O
N
H
O
0.84
0.91
0.81
0.90
4.35
0.99
1.07
1.07
1.03
1.00
1.19
1.98
3.32
3.29
f1 (ppm)

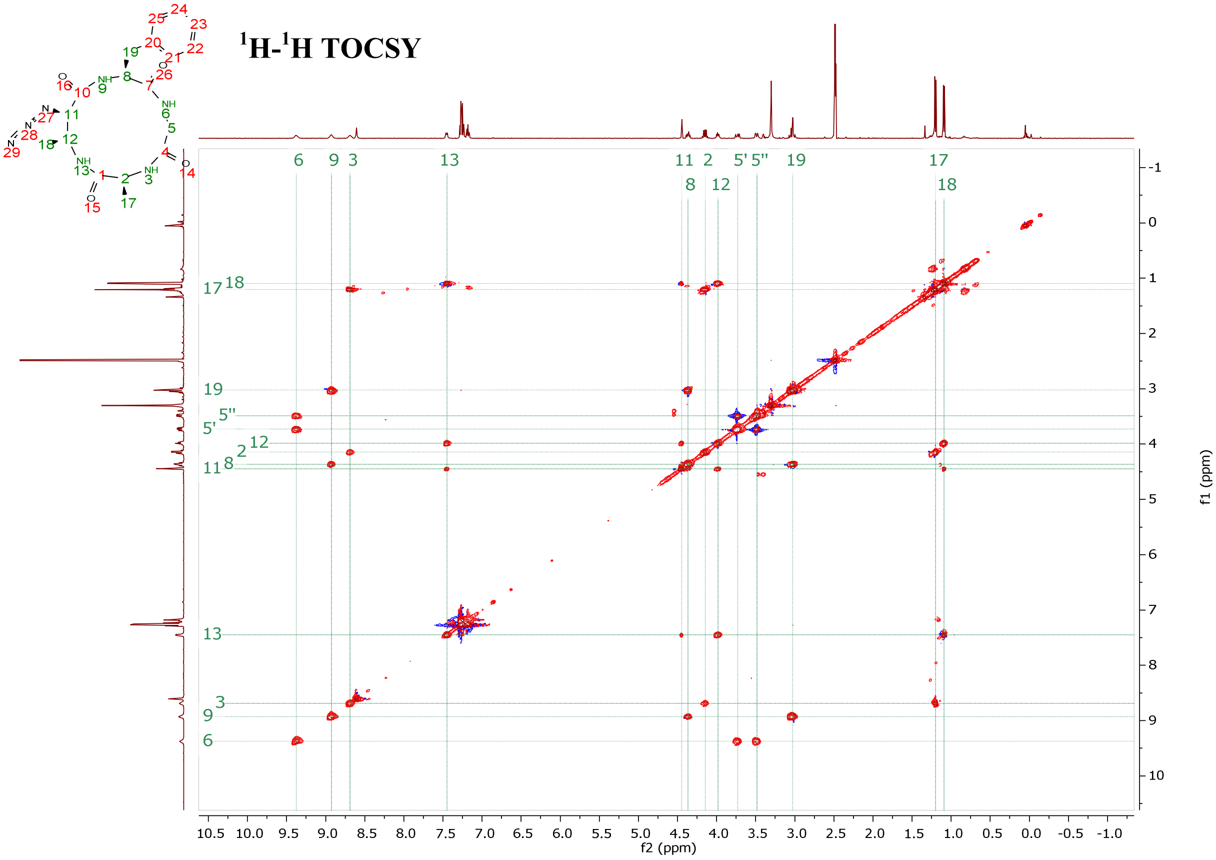

f2 (ppm)
f1 (ppm)

^{1}H-^{1}H TOCSY

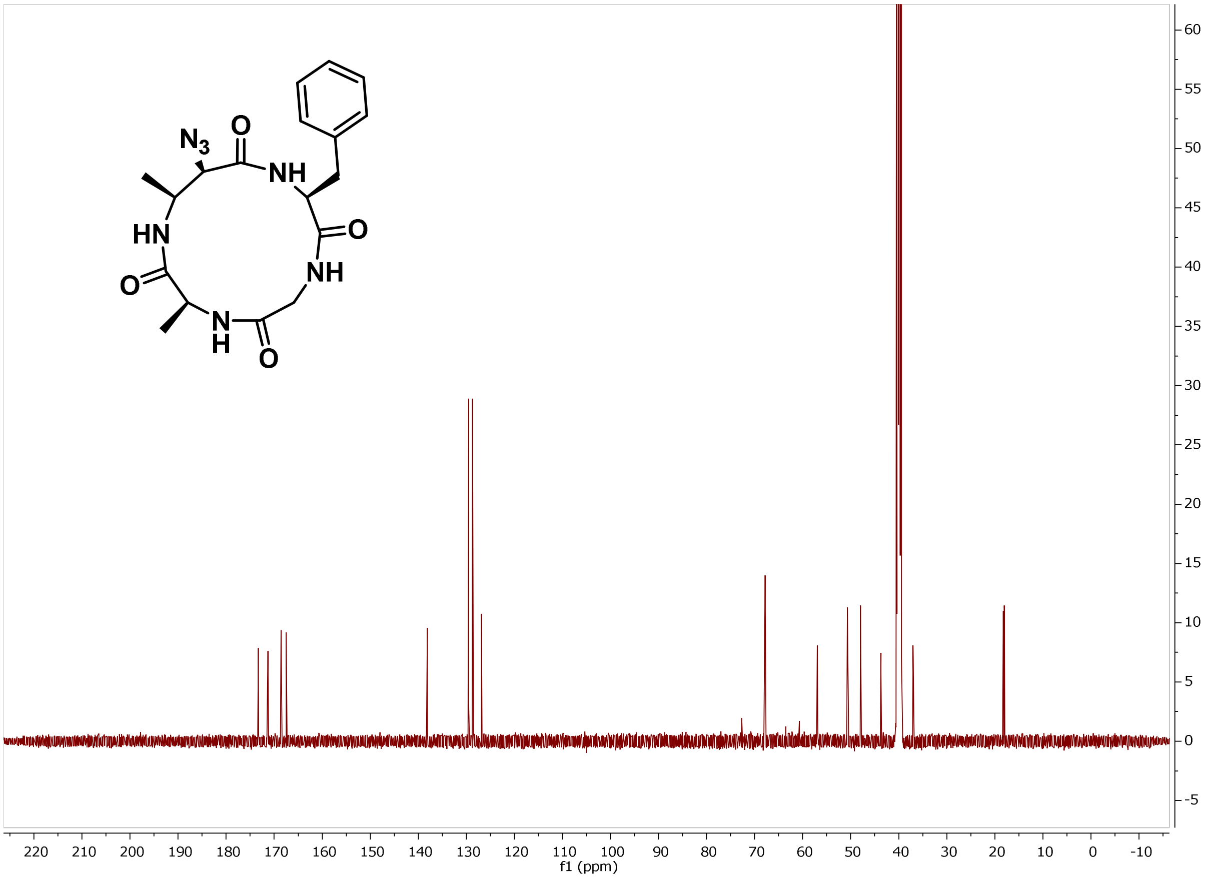
N3
O
NH
HN
O
NH
N
H
O
60
55
50
45
40
35
30
25
20
15
10
5
0
-5
220
210
200
190
180
170
160
150
140
130
120
110
100
90
80
70
60
50
40
30
20
10
0
-10
f1 (ppm)

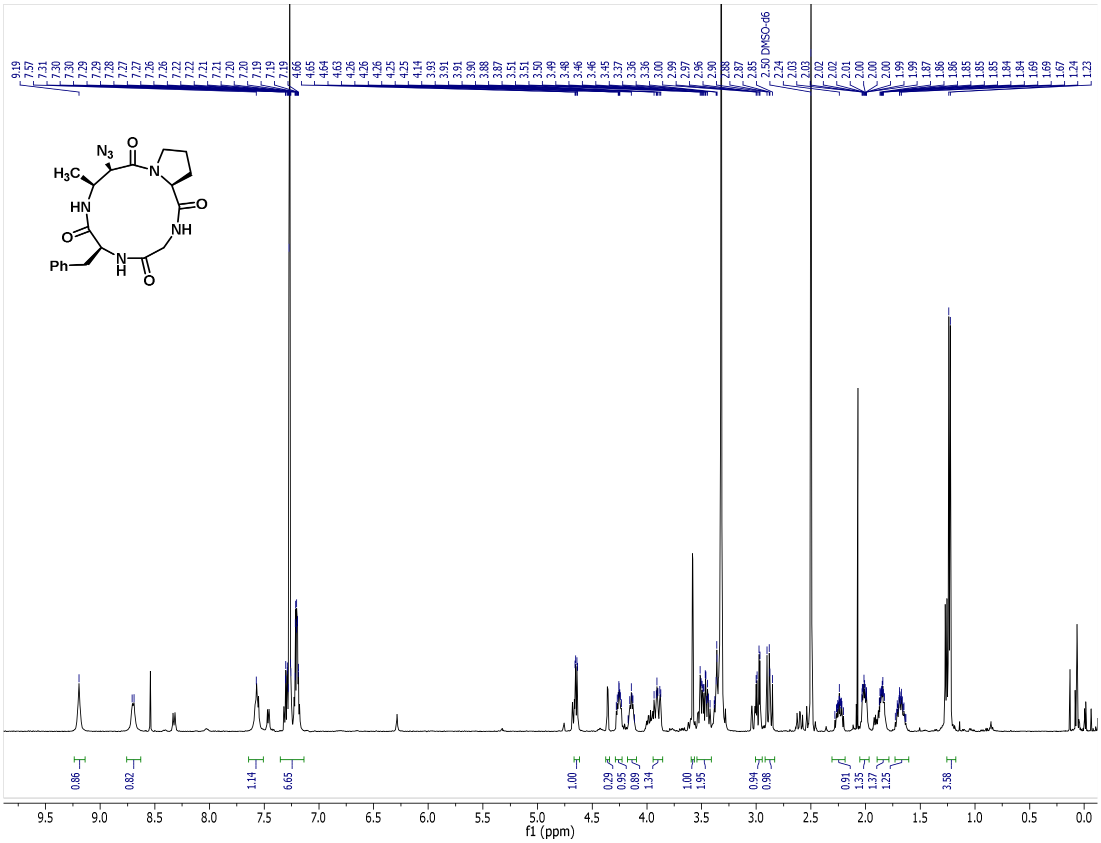
N3
O
N
H3C
HN
O
NH
O
Ph
N
H
O
9.19 7.57 7.31 7.30 7.30 7.29 7.29 7.28 7.27 7.27 7.26 7.26 7.22 7.22 7.21 7.21 7.20 7.20 7.19 7.19 7.19 7.19 4.66 4.65 4.64 4.63 4.26 4.26 4.26 4.25 4.25 4.14 3.93 3.91 3.91 3.90 3.88 3.87 3.51 3.51 3.50 3.49 3.48 3.46 3.46 3.45 3.37 3.36 3.36 3.00 2.99 2.97 2.96 2.90 2.88 2.87 2.85 2.50 DMSO-d6 2.24 2.03 2.03 2.02 2.02 2.01 2.00 2.00 2.00 1.99 1.99 1.87 1.86 1.86 1.85 1.85 1.85 1.84 1.69 1.69 1.67 1.24 1.23
0.86 0.82 1.14 6.65 1.00 0.29 0.95 0.89 1.34 1.00 1.95 0.94 0.98 0.91 1.35 1.37 1.25 3.58
9.5 9.0 8.5 8.0 7.5 7.0 6.5 6.0 5.5 5.0 4.5 4.0 3.5 3.0 2.5 2.0 1.5 1.0 0.5 0.0
f1 (ppm)

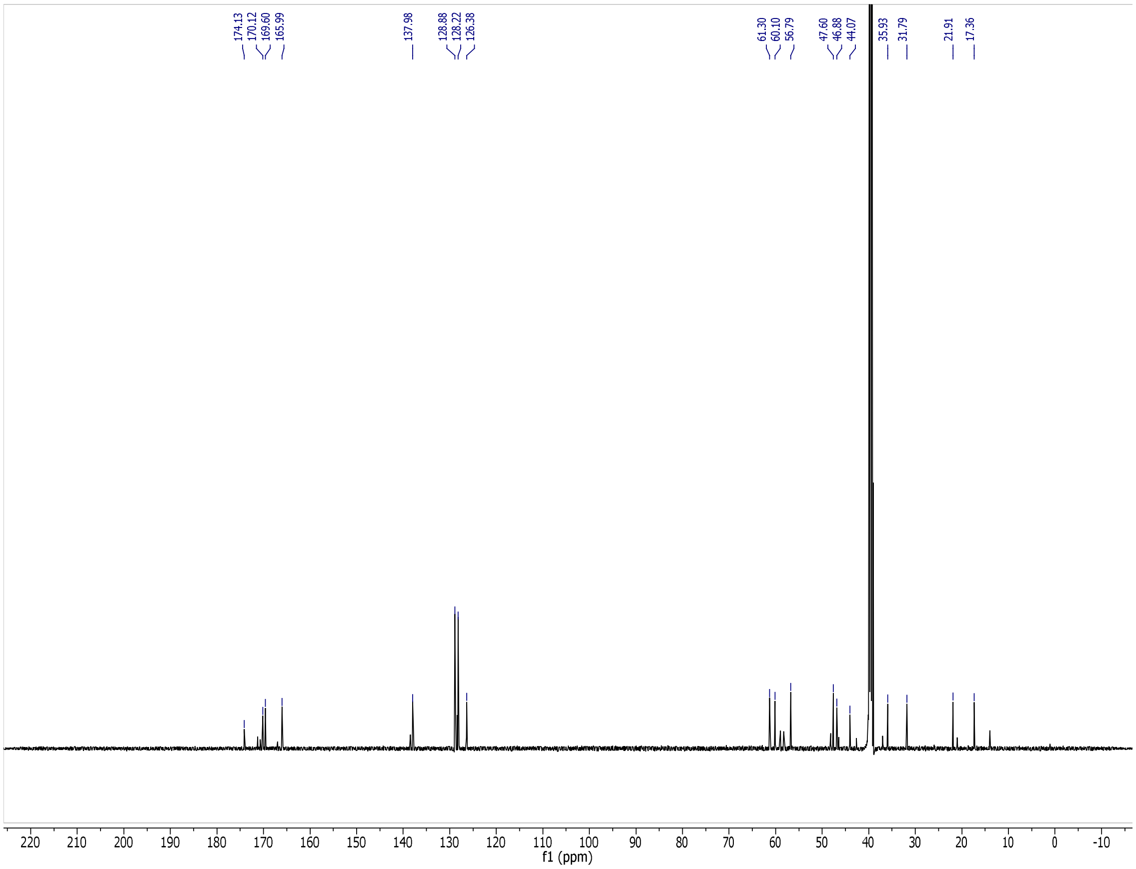
174.13 170.12 169.60 165.99 137.98 128.88 128.22 126.38 61.30 60.10 56.79 47.60 46.88 44.07 35.93 31.79 21.91 17.36
220 210 200 190 180 170 160 150 140 130 120 110 100 90 80 70 60 50 40 30 20 10 0 -10
f1 (ppm)

COSY spectrum

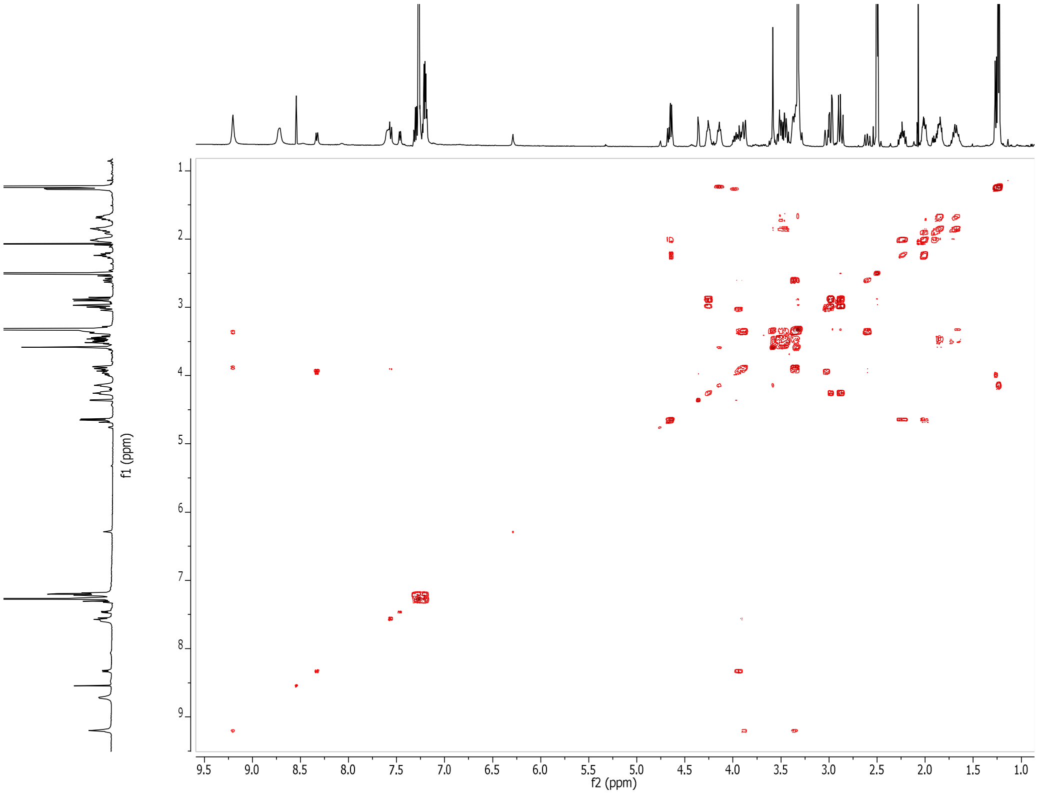

TOCSY spectrum

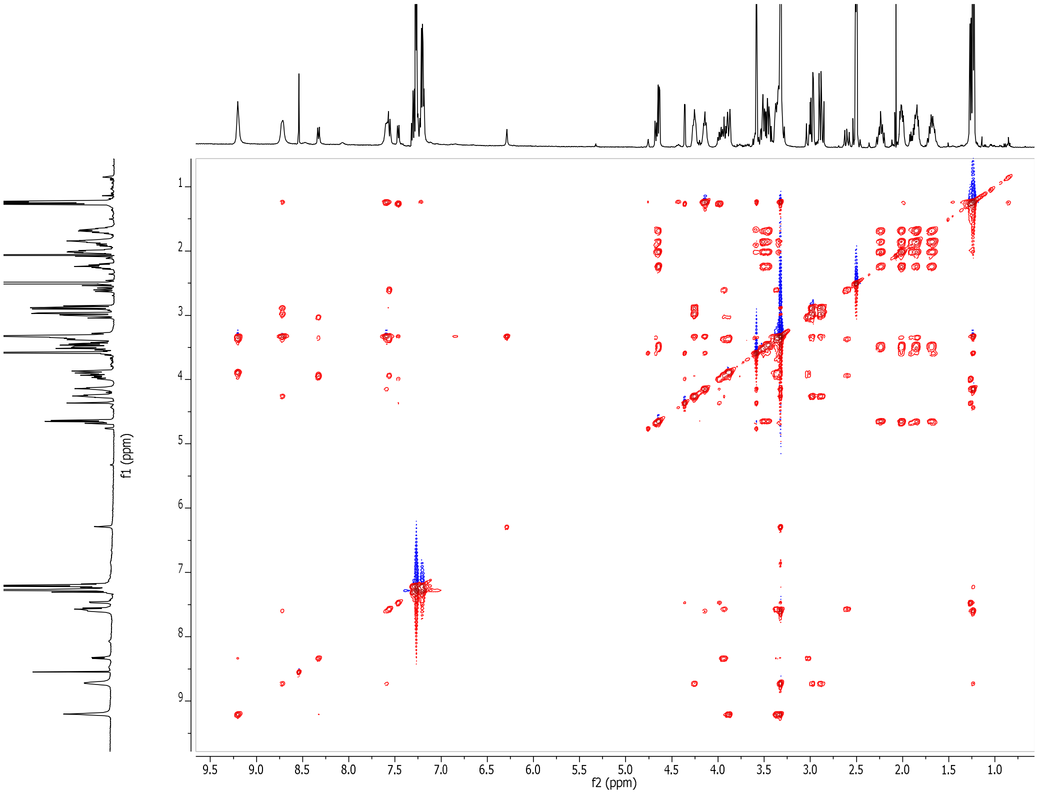

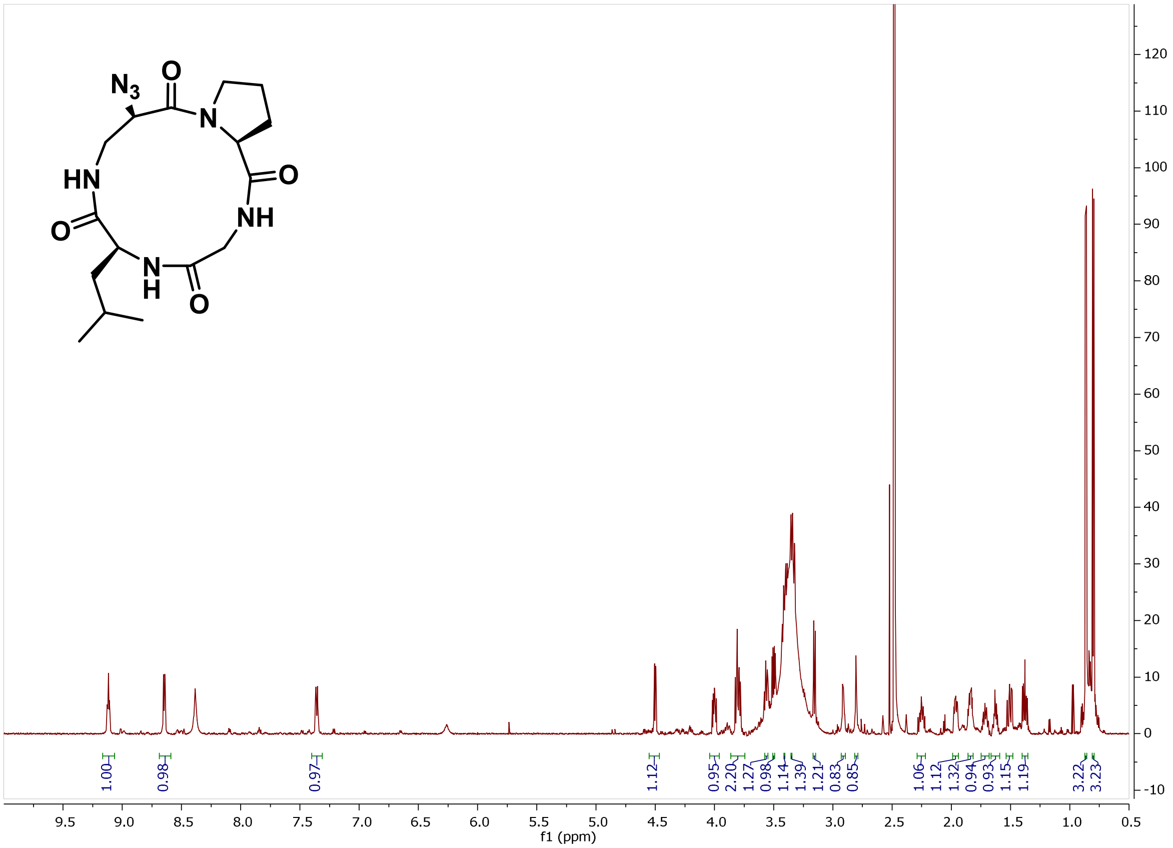
N3
O
N
HN
O
NH
O
N
H
O
1.00
0.98
0.97
1.12
0.95
2.20
1.27
0.98
1.14
1.39
1.21
0.83
0.85
1.06
1.12
1.32
0.94
0.93
1.15
1.19
3.22
3.23
f1 (ppm)

^{1}H-^{1}H TOCSY

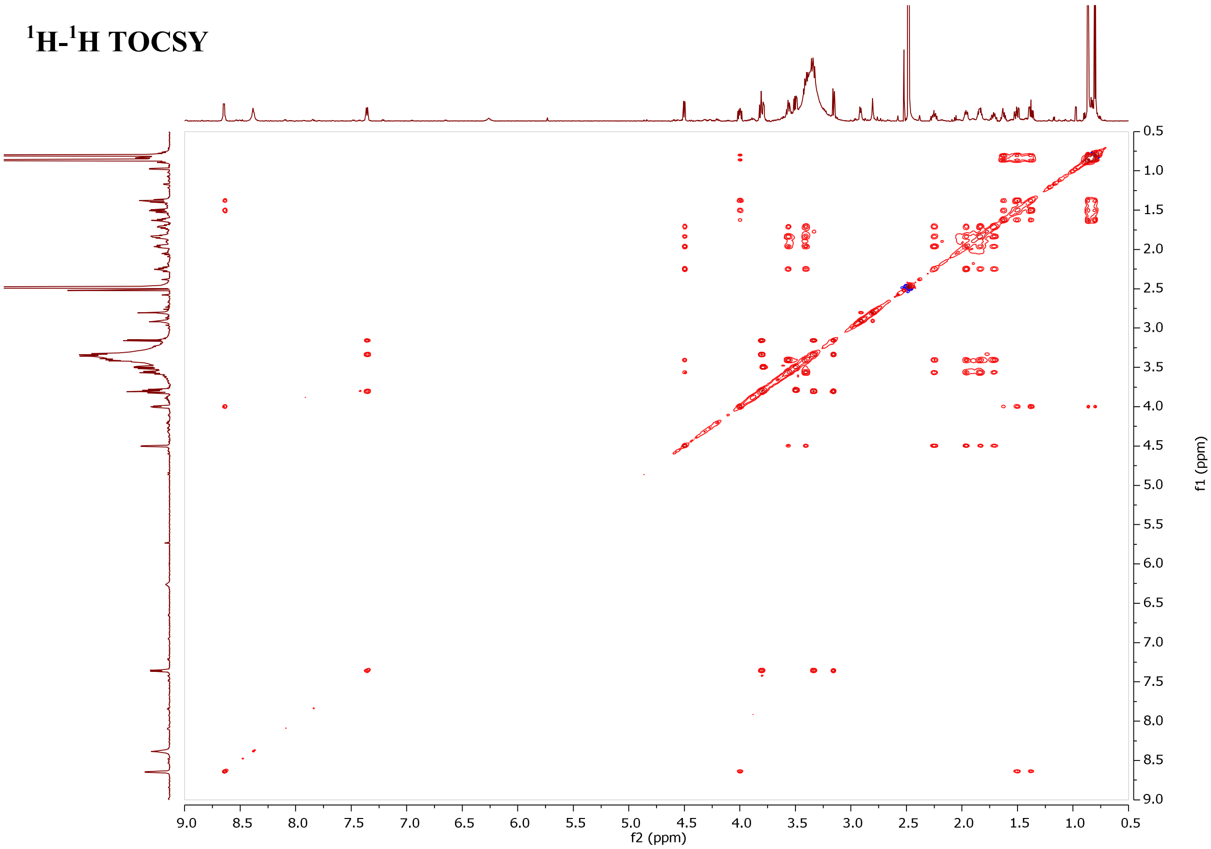
f2 (ppm)
f1 (ppm)

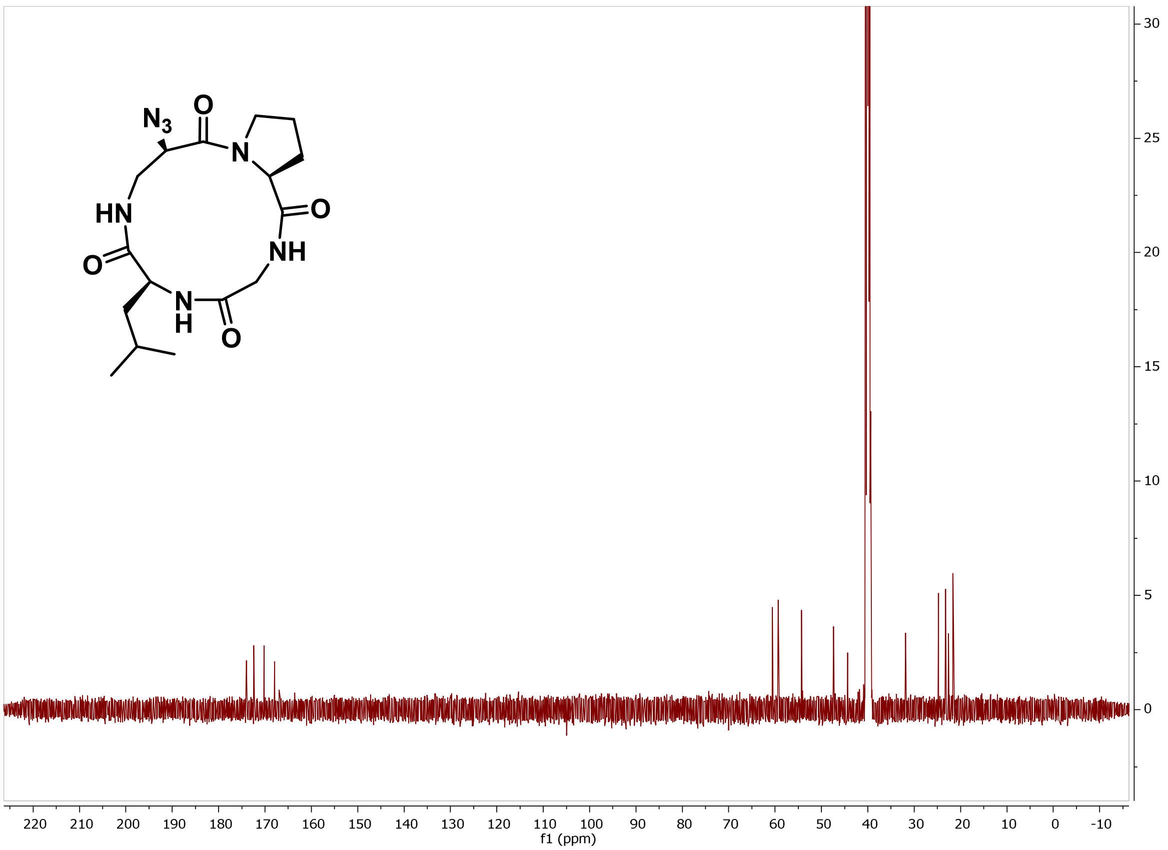

^{1}H-^{13}C HSQC

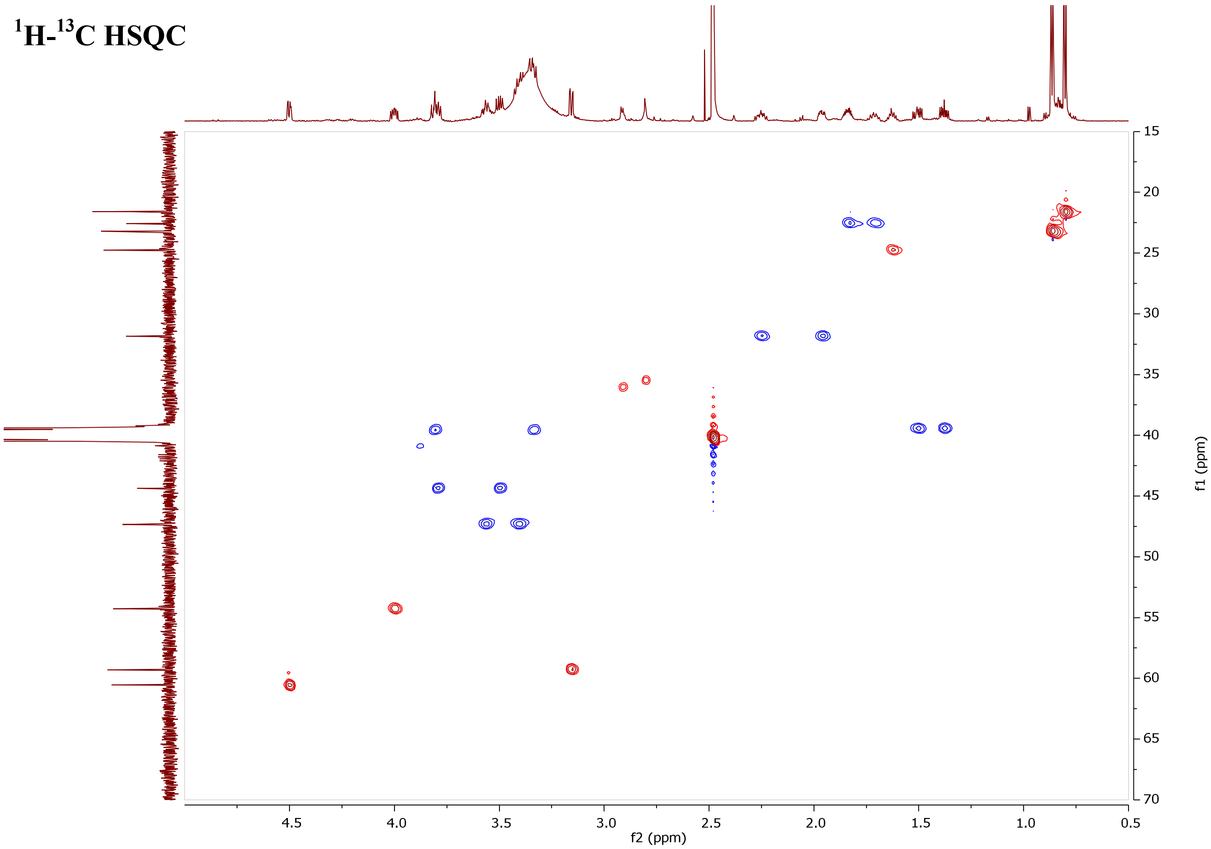

5. Exchange Spectroscopy (EXSY) to Quantify Conformational Exchange

Exchange spectroscopy (EXSY) is an NMR experiment from which experimental intensities of the NMR peaks obtained can be used to calculate the magnetization exchange rates of the exchange equilibrium between multiple conformations. This technique requires that the signals of each different species are sufficiently resolved, i.e., undergo slow chemical exchange in the chemical shift time scale. As EXSY NMR measures the extent of magnetization transfer from one proton to another, it is essentially a NOESY experiment with a different mixing time. Specifically, two different NOESY experiments need to be obtained per signal – one is acquired at a certain mixing time (determined by T1 inversion recovery experiments), and the other is acquired at a mixing time of 0 ms. As there is no magnetization transfer observed in the 0 ms mixing time experiment, this allows for the baseline in the absence of any magnetization transfer processes to be accounted for in the calculations. In addition, for exchange between two signals (corresponding to two different conformations, e.g. signal A and B), these two experiments must be performed for each signal (ie., irradiate A to observe magnetization transfer to B, and vice versa). This means that a total of four NOESY experiments must be performed.

These two pairs of 1D NOESY experiments were performed, where each pair involves selective irradiation of H_{maj} to H_{min} (designated "forward" direction) and H_{min} to H_{maj} (designated "reverse" direction). This first pair of experiments is run at a certain mixing time that must be large enough for magnetization transfer to occur. The second pair of experiments is run with a 0 ms mixing time and is used as a reference. With the spectra in hand, the integrations of all peaks can be obtained using NMR processing software, and the values can be input into the EXSYCALC program (Mestrelab Research SL) to obtain rate constants for the forward and reverse directions, k_1 and k_{-1}. These rate constants can be used to calculate the barriers of conformational interconversion $\Delta G_1^‡$ and $\Delta G_{-1}^‡$ using the rearranged Eyring equation, and the difference between them will provide the ΔΔG between the major and minor conformations, which can be used to calculate K *via* ΔG = –RT ln K. The value for K can be used to compare with the integrations for the major and minor conformations, respectively, obtained by ^{1}H NMR, as a self-check for consistency.

For quantitative analysis of the kinetics of conformational interconversion of **7a** in DMSO-d_6, T1 inversion recovery and 1D NOESY experiments were performed on **5** using an Agilent DD2-500 spectraometer equipped with an Agilent OneNMR probe. For the 1D NOESY experiments, two experiments per peak were run, one with 0 ms mixing time and another with 500 ms mixing time, with 64 scans each. A pair of ^{1}H signals (corresponding to C**H**–N_3, δ = 4.60 (major conformer), 4.49 (minor conformer) ppm) was chosen for their resolution from neighbouring signals, facilitating selective irradiation of the desired nuclei only. The resulting integrations obtained from the 1D NOESY experiments were analyzed using the EXSYCALC program (the input matrix is summarized in the table above) to yield the kinetic parameters k_{ct} = 0.029 s^{-1} and k_{tc} = 0.395 s^{-1}. These values were used to calculate $\Delta G_{ct}^‡$ and $\Delta G^‡_{tc}$ using the Eyring equation, and as a consistency check, the difference between them was used to calculate ΔΔG and K, giving a ratio of 1 : 0.07 (major : minor conformation), which is consistent with ^{1}H NMR integrations.

	**A**	**B**		**Diagonal Peak**
**A**	2646.99	36.00	**A**	3863.31
**B**	47.05	224.50	**B**	369.08

1D NOESY Spectra: Irradiation of δ = 4.60 ppm at 500 ms (teal) and 0 ms mixing time (maroon)

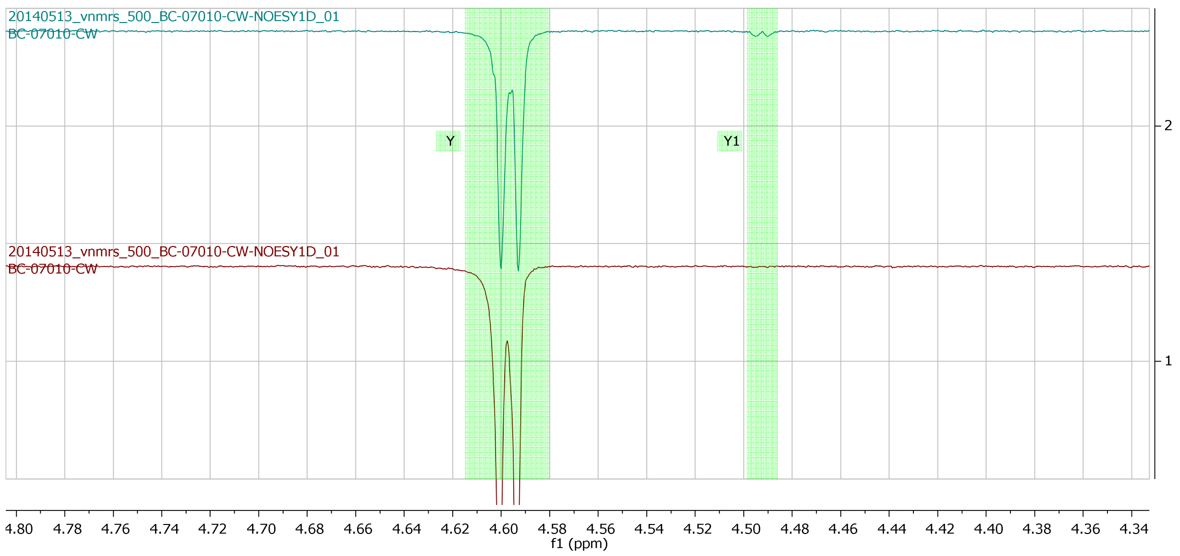

1D NOESY Spectra: Irradiation of δ = 4.49 ppm at 500 ms (teal) and 0 ms mixing time (maroon)

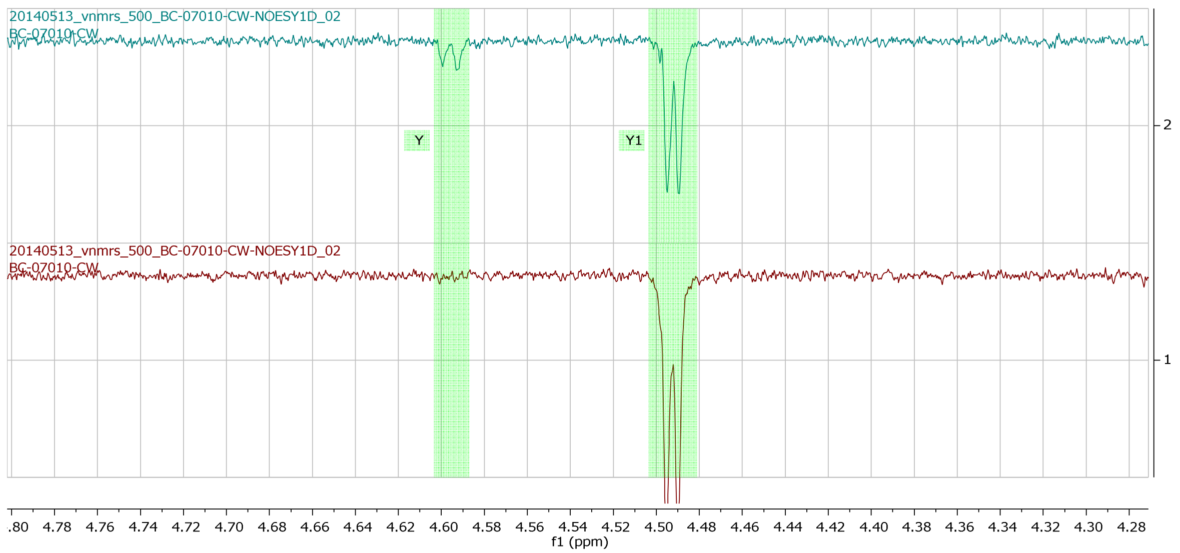

Supplement: Supplementary file 1 [file SC-007-C6SC01687A-s001.pdf]
